# Studies in Hawaiian Diptera III: New Distributional Records for Canacidae and a New Endemic Species of *Procanace*

**DOI:** 10.3897/BDJ.4.e5611

**Published:** 2016-04-08

**Authors:** Patrick M O'Grady, Nina Pak

**Affiliations:** ‡ UC Berkeley, Berkeley, United States of America

**Keywords:** Hawaii, Diptera, Canacidae, Procanace

## Abstract

The distributions of Hawaiian Canacidae, comprising nearly 800 individual collection events, are reviewed and a total of four new island records are reported. These include *Canaceoides angulatus* from Kahoolawae and *Procanace bifurcata* from Molokai and Maui, and *Procanace constricta* from Oahu. A new species from Kauai, *Procanace hardyi* O'Grady and Pak, is described. This species is closely related to *P. constricta* from Oahu, Maui, Molokai and Hawaii and shares a similar constriction of the abdomen between tergites four and five but differs in the configuration of the seventh abdominal tergite. Detailed distribution maps for all species are included.

## Introduction

Canacidae, or the beach flies, surf flies and surge flies, is a relatively small family of acalyptrate Diptera primarily found throughout coastal regions of the world. A number of catalogs and revisions have been published in the past 20 years. Traditionally, this group contained only members of the family Canacidae, *sensu stricto.* The Mathis (1992) catalog of this family consisted of 113 species in 12 genera and recognized three subfamilies (Canacinae, Nocticanacinae, Zaleinae). Mathis and Munari (1996) transferred Zaleinae to Tethinidae when they published their tethinid catalog because this subfamily was considered intermediate between the two families. According to their definition, Tethinidae included 126 species in 14 genera arranged into five subfamilies (Apetaeninae, Horaismopterinae, Pelomyiinae, Tethininae, Zaleinae). McAlpine (2007) significantly altered the concept of this family when he included the family Tethinidae as part of a broader Canacidae *sensu lato.* Finally, Munari and Mathis (2010) updated the previous catalogs and now recognize six subfamilies comprising a total of 307 species in 27 genera within the Canacidae.

Five canacid genera are present in Hawaii (Nishida 2002​), most of which are represented by only one or two species and were introduced by human activities in the past 100 years. Two genera within the subfamily Tethininae are present in Hawaii, *Dasyrhicnoessa* and *Tethina*. These were recently reviewed and updated by Munari and Evenhuis (2011) and the distributional records will not be repeated here. The genus *Dasyrhicnoessa* contains a total of 28 species, 22 of which are found in the Pacific and four of which are present in Hawaii (Evenhuis 2012, Hardy and Delfinado 1980, Munari and Mathis 2010, Munari and Evenhuis 2011). *Dasyrhicnoessa insularis* was first collected in Hawaii in 1923 and is widespread in Polynesia, Micronesia, Papua New Guinea, Central and South America and the Afrotropics (Evenhuis 2012, Hardy and Delfinado 1980). Hardy and Delfinado (1980) were unable to place the second species, *D. vockerothi*, in the literature and described it as a new species from Hawaii. Subsequent collections have found this species on islands throughout the Pacific and Indian Oceans, as well as in Japan and Australia (Evenhuis 2012). Finally, Munari and Evenhuis (2011) reported two additional species from Hawaii, *D. clandestina* and *D. fulva*. The genus *Tethina* is large, with 78 so far described species worldwide (Munari and Mathis 2010). Ten species are known from the Pacific, including three species from Hawaii. Hardy and Delfinado (1980) listed *T. variseta* from the Hawaiian Islands, a species that has since been synonymized with *T. willistoni* (Munari and Mathis 2010). Two other species, *T. albula* (Evenhuis 2012, Munari and Mathis 2010) and *T. pallipes* (Munari and Evenhuis 2011) have also been reported in Hawaii.

A single genus of the subfamily Pelomyiinae, *Pelomyia*, is found in Hawaii. *Pelomyia* contains a total of 29 species, mostly found in the Nearctic and Neotropical regions. Hardy and Delfinado (1980) described *P. steyskali* from material collected in Hawaii and the west Coast of North America. This species has since been synonymized with *P. occidentalis*, a widespread taxon that is known from Nearctic, Neotropical and Palearctic regions (Evenhuis 2012, Hardy and Delfinado 1980, Munari and Mathis 2010).

The remaining two canacid genera present in Hawaii, *Canaceoides* Cresson and *Procanace* Hendel, are both in the subfamily Canacinae. These two genera can be found in a range of habitats, from the coastal aquatic environments to high elevation freshwater streams. They are most likely the result of at least two separate colonization events (Wirth 1969). The genus *Canaceoides* was last revised by Wirth (1969) and contains 9 species, all of which are either found on Pacific Islands or are distributed along the west coast of North and South America. Two species are recorded from Hawaii, *C. angulatus* and *C. hawaiiensis* (Table 1). Wirth (1969) suggested that the widespread distribution of *C. angulatus*, which is found throughout the Hawaiian Archipelago and Peru, Mexico and the Galapagos Islands (Evenhuis 2012), is evidence for this taxon arriving in Hawaii prior to human contact. He proposed, however, that this was more recently than *C. hawaiiensis*, a species found exclusively in the younger Hawaiian Islands. The biogeographic history and evolutionary relationship of these two species is unclear. The male genitalia of *C. angulatus* is quite distinct for the genus and its sister relationships are not known. Likewise, Wirth (1969) states that *C. hawaiiensis* “has no exact counterpart on the American coasts.” It is possible that these two taxa represent two distinct colonizations of Hawaii. Alternatively, these two species could represent a single colonization of Hawaii, with *C. hawaiiensis* being a recently divergent sibling species of *C. angulatus*. Phylogenetic analyses will need to be undertaken to differentiate between these two hypotheses. Aside from the early collections made by D.E. Hardy, W.W. Wirth and others, there are a number of surveys conducted by D. Polhemus and R. Englund for the Bernice P. Bishop Museum in the early 1990s (e.g., Polhemus 1992, Polhemus 1993).

The genus *Procanace* contains 30 species, found in the Palearctic, Nearctic, Australasian/Oceanian and Afrotropical regions. The Oceanian taxa include a small radiation of eight described species present in Hawaii (Table 1). Of these taxa, seven are endemic and one, *P. williamsi*, is known from Oahu and Japan (Evenhuis 2012). It is possible that *P. williamsi* was introduced to Hawaii in the 1940s and has since failed to establish in Hawaii, a common phenomenon among adventive species (Kolar and Lodge 2001). Two individuals are reported by Wirth, one taken at a light on Oahu and another taken in a plane. Additional collections will be required in order to determine the status of *P. williamsi*.

The endemic *Procanace* species are found on all the high Hawaiian Islands. There is one widespread species, *P. bifurcata*, found across the islands. The remainder of the species are found either on the "old" islands of Oahu and Kauai in the northwest and the "young" islands of the Maui Nui complex (Maui, Molokai, Lanai, Kahoolawe) and Hawaii in the southeast (Table 1​). For example, *P. hardyi*, *P. nigroviridis*, *P. quadrisetosa*, and *P. wirthi* are present on the older islands in the archipelago. These are mostly single island endemics, although *P. wirthi* is found on both Oahu and Kauai. In contrast, *P. acuminata*, *P. confusa*, and *P. constricta* are found on Maui Nui and Hawaii. These species are not single island endemics and can be found on multiple islands of the Maui Nui group and/or on Hawaii. This difference in distribution pattern may be because of the recent connections between Maui, Molokai, Lanai and Kahoolawae (Price and Elliott-Fisk 2004​). Alternately, it might be because the Big Island of Hawaii is less than 500,000 years old and speciation has yet to take place between founder populations on Maui Nui and descendant populations on Hawaii.

The endemic Hawaiian *Procanace* are notable within the genus and the family for their adaptation to freshwater, rather than saltwater, habitats. Hardy and Delfinado (1980) listed seven described endemics (*P. acuminata*, *P.bifurcata, P. confusa, P. constricta, P. nigroviridis, P. quadrisetosa, P. wirthi*) and one additional species from the island of Hawaii that they did not describe. Here we describe a new species from Kauai, *Procanace hardyi* O'Grady and Pak, bringing the total Hawaiian *Procanace* to nine (eight endemic and one adventive) described and one underscribed species.

## Materials and methods

### Literature Review

We have reviewed the literature for the endemic, indigenous, and adventive Canacidae species present in the Hawaiian islands. We have attempted to include infomation on both the taxonomic history of the species and all occurrences of these taxa present in the literature, including the original descriptions, subsequent revisions, additional descriptive notes, range expansions and new island records and catalogs. We list the primary type, paratypes and other material examined, whether they were physically examined by us or taken from a database. We include all records from the EMEC and the BPBM. Additional material deposited in the National Museum of Natural History, Smithsonian Institution (USNM) and the UHM are also presented here. All museum abbreviations conform to Evenhuis (2014).

### Collection Methods

All recently collected material was obtained from general sweeping of streams and sea shores. Samples were preserved in 95% ethanol (ETOH) and transported to UC Berkeley for identification and subsequent molecular work. The key and descriptions in (Hardy and Delfinado 1980) were used to identify taxa to species. Vouchers for all species were preserved in 95% ETOH and are deposited in the EMEC.

## Taxon treatments

### Procanace hardyi

O'Grady and Pak sp. n.

urn:lsid:zoobank.org:act:8FF8BBA3-45A9-465A-962B-8AC994B7652F

#### Materials

- **Type status:** Holotype. **Occurrence:** recordedBy: PM O'Grady, RT Lapoint, GM Bennett, B Ort, NA Pantoja; individualCount: 1; sex: female; **Taxon:** kingdom: Animalia; phylum: Arthropoda; class: Insecta; order: Diptera; family: Canacidae; genus: Procanace; specificEpithet: Procanacehardyi; **Location:** islandGroup: Hawaiian Islands; island: Kauai; country: United States; stateProvince: Hawaii; county: Kauai; verbatimLocality: Kokee Stream near Canyon Trail; verbatimLatitude: 22° 6'18.56"N; verbatimLongitude: 159°39'41.12"W; **Identification:** identifiedBy: PM O'Grady; dateIdentified: 7 Oct 2014; **Event:** samplingProtocol: sweeping over running water; eventDate: 10.i.2010; **Record Level:** collectionID: 588.7; institutionCode: EMEC
- **Type status:** Paratype. **Occurrence:** recordedBy: DA Polhemus; individualCount: 1; sex: 1 male; lifeStage: adult; **Taxon:** kingdom: Animalia; phylum: Arthropoda; class: Insecta; order: Diptera; family: Canacidae; genus: Procanace; specificEpithet: Procanacehardyi; **Location:** islandGroup: Hawaiian Islands; island: Kauai; country: United States; stateProvince: Hawaii; verbatimLocality: Waiahuakua Stream at Kalalau Trail, on wet rocks; verbatimElevation: 400 ft.; **Identification:** identifiedBy: PM O'Grady; dateIdentified: 1.2016; **Event:** eventDate: 30.iii.1993; **Record Level:** collectionID: CL8133; institutionCode: BPBM
- **Type status:** Paratype. **Occurrence:** recordedBy: DA Polhemus; individualCount: 3; sex: females; lifeStage: adult; **Taxon:** kingdom: Animalia; phylum: Arthropoda; class: Insecta; order: Diptera; family: Canacidae; genus: Procanace; specificEpithet: Procanacehardyi; **Location:** islandGroup: Hawaiian Islands; island: Kauai; country: United States; stateProvince: Hawaii; verbatimLocality: Kaapoko Stream, tributary to Hanalei River, on wet rocks,; verbatimElevation: 1200ft.; **Identification:** identifiedBy: PM O'Grady; dateIdentified: 2.2016; **Event:** eventDate: 3.xi.1993; **Record Level:** collectionID: CL8233; institutionCode: BPBM
- **Type status:** Paratype. **Occurrence:** recordedBy: DA Polhemus; individualCount: 2; sex: 1 male, 1 female; lifeStage: adult; **Taxon:** kingdom: Animalia; phylum: Arthropoda; class: Insecta; order: Diptera; family: Canacidae; genus: Procanace; specificEpithet: Procanacehardyi; **Location:** islandGroup: Hawaiian Islands; island: Kauai; country: United States; stateProvince: Hawaii; verbatimLocality: Kaapoko Stream, trib. to Hanalei River; verbatimElevation: 1200 ft.; **Identification:** identifiedBy: PM O'Grady; dateIdentified: 1.2016; **Event:** samplingProtocol: in malaise trap; eventDate: 3.xi.1994; **Record Level:** collectionID: CL8233; institutionCode: BPBM
- **Type status:** Paratype. **Occurrence:** recordedBy: RA England; individualCount: 6; sex: 5 females, 1 male; lifeStage: adult; **Taxon:** kingdom: Animalia; phylum: Arthropoda; class: Insecta; order: Diptera; family: Canacidae; genus: Procanace; specificEpithet: Procanacehardyi; **Location:** islandGroup: Hawaiian Islands; island: Kauai; country: United States; stateProvince: Hawaii; verbatimLocality: Koiae Stream, 150m down from dam; verbatimElevation: 3500 ft; **Identification:** identifiedBy: PM O'Grady; dateIdentified: i.2016; **Event:** samplingProtocol: 20 net swings; eventDate: 1.viii.1997; **Record Level:** institutionCode: BPBM
- **Type status:** Paratype. **Occurrence:** recordedBy: RA Englund; individualCount: 14; sex: 2 males, 12 females; lifeStage: adult; **Taxon:** kingdom: Animalia; phylum: Arthropoda; class: Insecta; order: Diptera; family: Canacidae; genus: Procanace; specificEpithet: Procanacehardyi; **Location:** islandGroup: Hawaiian Islands; island: Kauai; country: United States; stateProvince: Hawaii; verbatimLocality: Kauai, Koiae Stream, cascade riffles; verbatimElevation: 3680 ft.; **Identification:** identifiedBy: PM O'Grady; dateIdentified: i.2016; **Event:** samplingProtocol: 10 net swings; eventDate: 3.viii.1997; **Record Level:** institutionCode: BPBM
- **Type status:** Paratype. **Occurrence:** recordedBy: RA Englund; individualCount: 27; sex: 9 females, 18 males; lifeStage: adult; **Taxon:** kingdom: Animalia; phylum: Arthropoda; class: Insecta; order: Diptera; family: Canacidae; genus: Procanace; specificEpithet: Procanacehardyi; **Location:** islandGroup: Hawaiian Islands; island: Kauai; country: United States; stateProvince: Hawaii; verbatimLocality: Kauai, Kokee, Waialae Stream, rocks in stream; verbatimElevation: 3400 ft.; **Identification:** identifiedBy: PM O'Grady; dateIdentified: i.2016; **Event:** eventDate: 5-6.i.1999; **Record Level:** institutionCode: BPBM
- **Type status:** Paratype. **Occurrence:** recordedBy: D Preston; individualCount: 52; sex: 29 females, 23 males; lifeStage: adult; **Taxon:** kingdom: Animalia; phylum: Arthropoda; class: Insecta; order: Diptera; family: Canacidae; genus: Procanace; specificEpithet: Procanacehardyi; **Location:** islandGroup: Hawaiian Islands; island: Kauai; country: United States; stateProvince: Hawaii; verbatimLocality: Kokee, Waialae Stream, rocks in stream; verbatimElevation: 3400 ft.; **Identification:** identifiedBy: PM O'Grady; dateIdentified: i.2016; **Event:** samplingProtocol: 20 net swings; eventDate: 5-6.i.1999; **Record Level:** institutionCode: BPBM
- **Type status:** Paratype. **Occurrence:** recordedBy: D Preston; individualCount: 44; sex: 24 females, 20 males; lifeStage: adult; **Taxon:** kingdom: Animalia; phylum: Arthropoda; class: Insecta; order: Diptera; family: Canacidae; genus: Procanace; specificEpithet: Procanacehardyi; **Location:** islandGroup: Hawaiian Islands; island: Kauai; country: United States; stateProvince: Hawaii; verbatimLocality: Kokee, Waialae Stream, rocks in stream; verbatimElevation: 3400 ft.; **Identification:** identifiedBy: PM O'Grady; dateIdentified: i.2016; **Event:** samplingProtocol: 30 net swings; eventDate: 5-6.i.1999; **Record Level:** institutionCode: BPBM
- **Type status:** Paratype. **Occurrence:** recordedBy: D Polhemus; individualCount: 101; sex: 49 females, 52 males; lifeStage: adult; **Taxon:** kingdom: Animalia; phylum: Arthropoda; class: Insecta; order: Diptera; family: Canacidae; genus: Procanace; specificEpithet: Procanacehardyi; **Location:** islandGroup: Hawaiian Islands; island: Kauai; country: United States; stateProvince: Hawaii; verbatimLocality: Kokee, Waialae Stream, first fall below Waialae cabin; **Identification:** identifiedBy: PM O'Grady; dateIdentified: 1.2016; **Event:** samplingProtocol: 25 net swings (#1); eventDate: 5-6.i.1999; **Record Level:** institutionCode: BPBM
- **Type status:** Paratype. **Occurrence:** recordedBy: D Polhemus; individualCount: 41; sex: 23 females, 18 males; lifeStage: adult; **Taxon:** kingdom: Animalia; phylum: Arthropoda; class: Insecta; order: Diptera; family: Canacidae; genus: Procanace; specificEpithet: Procanacehardyi; **Location:** islandGroup: Hawaiian Islands; island: Kauai; country: United States; stateProvince: Hawaii; verbatimLocality: Kokee, Waialae Stream, first fall below Waialae cabin; verbatimElevation: 3500 ft.; **Identification:** identifiedBy: PM O'Grady; dateIdentified: i.2016; **Event:** samplingProtocol: 25 net swings (#2); eventDate: 5-6.i.1999; **Record Level:** institutionCode: BPBM
- **Type status:** Paratype. **Occurrence:** recordedBy: D Preston; individualCount: 101; sex: 46 females, 55 males; lifeStage: adult; **Taxon:** kingdom: Animalia; phylum: Arthropoda; class: Insecta; order: Diptera; family: Canacidae; genus: Procanace; specificEpithet: Procanacehardyi; **Location:** islandGroup: Hawaiian Islands; island: Kauai; country: United States; stateProvince: Hawaii; verbatimLocality: Kokee, Waialae Stream, rocks in stream; verbatimElevation: 3400 ft.; **Identification:** identifiedBy: PM O'Grady; dateIdentified: 1.2016; **Event:** samplingProtocol: 25 net swings (#3); eventDate: 5-6.i.1999; **Record Level:** institutionCode: BPBM
- **Type status:** Paratype. **Occurrence:** recordedBy: RA Englund; individualCount: 5; sex: 4 females, 1 male; lifeStage: adult; **Taxon:** kingdom: Animalia; phylum: Arthropoda; class: Insecta; order: Diptera; family: Canacidae; genus: Procanace; specificEpithet: Procanacehardyi; **Location:** islandGroup: Hawaiian Islands; island: Kauai; country: United States; stateProvince: Hawaii; verbatimLocality: Hanakapai Stream, 200m from ocean,; verbatimElevation: 5m; **Identification:** identifiedBy: PM O'Grady; dateIdentified: i.2016; **Event:** eventDate: 2.i.2002; **Record Level:** institutionCode: BPBM
- **Type status:** Paratype. **Occurrence:** recordedBy: PM O'Grady, RT Lapoint, GM Bennett, B Ort, NA Pantoja; individualCount: 3; sex: female; **Taxon:** kingdom: Animalia; phylum: Arthropoda; class: Insecta; order: Diptera; family: Canacidae; genus: Procanace; specificEpithet: Procanacehardyi; **Location:** islandGroup: Hawaiian Islands; island: Kauai; country: United States; stateProvince: Hawaii; county: Kauai; verbatimLocality: Kokee Stream near Canyon Trail; verbatimLatitude: 22° 6'18.56"N; verbatimLongitude: 159°39'41.12"W; **Identification:** identifiedBy: PM O'Grady; dateIdentified: 7 Oct 2014; **Event:** samplingProtocol: sweeping over running water; eventDate: 10.i.2010; **Record Level:** collectionID: 588.7; institutionCode: EMEC
- **Type status:** Paratype. **Occurrence:** recordedBy: PM O'Grady, KR Goodman, H Machado; individualCount: 1; sex: female; **Taxon:** kingdom: Animalia; phylum: Arthropoda; class: Insecta; order: Diptera; family: Canacidae; genus: Procanace; specificEpithet: Procanacehardyi; **Location:** islandGroup: Hawaiian Islands; island: Kauai; country: United States; stateProvince: Hawaii; county: Kauai; verbatimLocality: Kawaikoi Stream at Sugi Grove; verbatimLatitude: 22° 7'53.12"N; verbatimLongitude: 159°37'18.12"W; **Identification:** identifiedBy: PM O'Grady; dateIdentified: 7 Oct 2014; **Event:** eventDate: 19.ix.2011; **Record Level:** collectionID: 705.7; institutionCode: EMEC
- **Type status:** Other material. **Occurrence:** recordedBy: DE Hardy; individualCount: 4; sex: 4 females; lifeStage: adult; **Taxon:** kingdom: Amimalia; phylum: Arthropoda; class: Insecta; order: Diptera; family: Canacidae; genus: Procanace; specificEpithet: Procanacehardyi; **Location:** islandGroup: Hawaiian Islands; island: Kauai; country: United States; stateProvince: Hawaii; county: Kauai; verbatimLocality: Kalalau Valley; **Identification:** identifiedBy: PM O'Grady; dateIdentified: 22 Jan 2016; **Event:** eventDate: viii.1953; **Record Level:** institutionCode: USNM
- **Type status:** Other material. **Occurrence:** recordedBy: EH Bryan, Jr.; sex: 1 male; lifeStage: adult; **Taxon:** kingdom: Amimalia; phylum: Arthropoda; class: Insecta; order: Diptera; family: Canacidae; genus: Procanace; specificEpithet: Procanacehardyi; **Location:** islandGroup: Hawaiian Islands; island: Kauai; country: United States; stateProvince: Hawaii; county: Kauai; verbatimLocality: Awaawapuhi; **Identification:** identifiedBy: PM O'Grady; dateIdentified: 22 Jan 2016; **Event:** eventDate: viii.1953; **Record Level:** institutionCode: USNM
- **Type status:** Other material. **Occurrence:** recordedBy: DE Hardy; individualCount: 1; sex: 1 male; lifeStage: adult; **Taxon:** kingdom: Amimalia; phylum: Arthropoda; class: Insecta; order: Diptera; family: Canacidae; genus: Procanace; specificEpithet: Procanacehardyi; **Location:** islandGroup: Hawaiian Islands; island: Kauai; country: United States; stateProvince: Hawaii; county: Kauai; verbatimLocality: Kalalau Valley; **Identification:** identifiedBy: PM O'Grady; dateIdentified: 22 Jan 2016; **Event:** eventDate: viii.1953; **Record Level:** institutionCode: USNM
- **Type status:** Other material. **Occurrence:** recordedBy: DA Polhemus; individualCount: 1; sex: female; **Taxon:** kingdom: Amimalia; phylum: Arthropoda; class: Insecta; order: Diptera; family: Canacidae; genus: Procanace; specificEpithet: Procanacehardyi; **Location:** islandGroup: Hawaiian Islands; island: Kauai; country: United States; stateProvince: Hawaii; county: Kauai; verbatimLocality: Kawaikoi Stream at Sugi Grove, Grove Campground; verbatimElevation: 1050; minimumElevationInMeters: 787; **Identification:** identifiedBy: PM O'Grady; dateIdentified: 22 Jan 2016; **Event:** eventDate: 08.xi.1990; **Record Level:** institutionCode: USNM
- **Type status:** Other material. **Occurrence:** recordedBy: DA Polhemus; individualCount: 11; sex: 7 males, 4 females; **Taxon:** kingdom: Amimalia; phylum: Arthropoda; class: Insecta; order: Diptera; family: Canacidae; genus: Procanace; specificEpithet: Procanacehardyi; **Location:** islandGroup: Hawaiian Islands; island: Kauai; country: United States; stateProvince: Hawaii; county: Kauai; verbatimLocality: Makaleha Stream, at Makaleha Springs; **Identification:** identifiedBy: PM O'Grady; dateIdentified: 22 Jan 2016; **Event:** eventDate: 08.xi.1990; **Record Level:** institutionCode: USNM

#### Description

**Figure 1.**
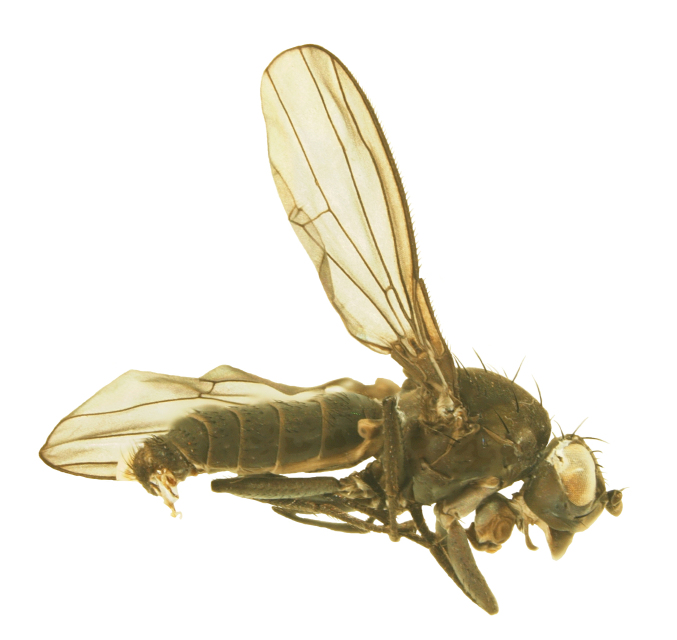
Habitus of a paratype male of *P. hardyi* in lateral view.

**Figure 2.**
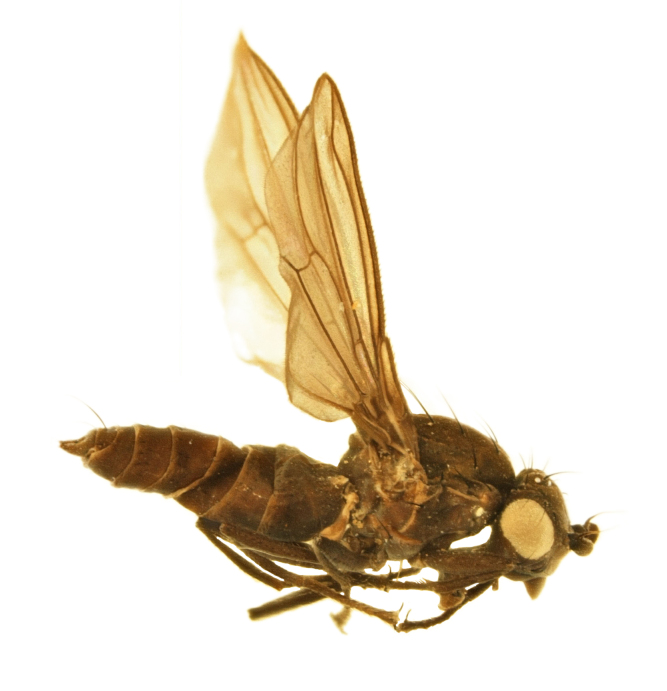
Habitus of the holotype female of *P. hardyi* in lateral view.

**Figure 3.**
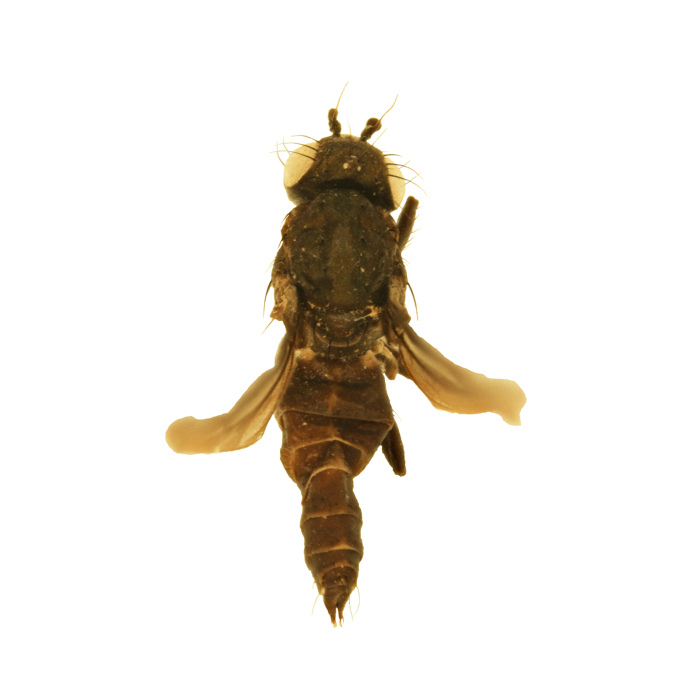
Habitus of the holotype female of *P. hardyi* in dorsal view.

**Figure 4.**
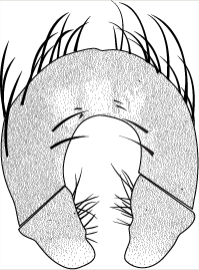
External male genitalia of *P. hardyi*. The narrowed, lightened tips of the surstyli suggest that this species is most closely related to *P. constricta*. Refer to figure 162e from Hardy and Delfinado (1980).

**Figure 5.**
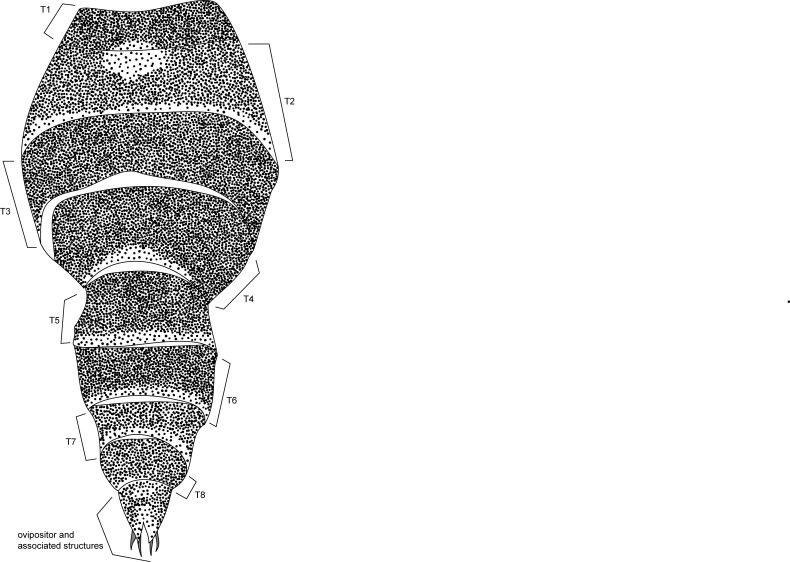
Abdomen of *P. hardyi* female, showing constriction between tergites 4 and 5, the telescoping form of tergite 8 and the ovipositor and associated structures.

**MALE, FEMALE. Head.** Ground color dull black (Figs 1, 2, 3). Frons and fronto-orbital plates covered with indistinct golden pollen. Frons lacking setae. Three subequal lateroclinate fronto-orbital setae present. Ocellar triangle subshining black; ocelli whitish gray; two strong proclinate ocellar setae. Postocellar setae absent. Inner and outer vertical setae strong, subequal. Antennae, face, carina, gena and clypeus subshining, gray pollinose. Arista pubescent, inserted at base of third segment. Gena wide, about 3/4 width of eye, with three setae inserted along anterior margin of eye. Anterior genal seta weak, about 1/3 length of two strong, upward-directed posterior genal setae. Eye dark red, ovoid in shape, about two times longer than high. **Thorax.** Mesonotum dull black, indistinctly golden pollinose. Four pairs of dorsocentral setae present; anterior pair inserted well forward of suture; posteriormost are inserted slightly laterally relative to the others. One strong humeral seta. Scutellum subshining black, with two convergent anterior and two convergent posterior scutellar setae. **Legs.** Entirely black. **Wings.** Smoky dark gray, with even dark infuscation on entire surface of blade. Halteres brown. **Abdomen.** Subshining dark brown to black, with slightly lighter areas on posterior margins of each seqment. Epandriium and surstyli of males very similar to P. constricta. Apices of surstyli narrowed, not straight-sided (Figs 1, 4). Surstyli and central region of epandrium lightened. Females with a strong constriction between tergites four and five (Figs 2, 3, 5). Seventh tergite greatly expanded along ventrolateral margins, extending beyond the apices of the cerci. Eighth tergite with two strong dorsal setae along midline of seventh tergite and two subequal but finer setae on lateral margins of segment. Ovipositor strongly bifurcate in a distinct V shape. Ovipositor about two times longer than wide, with each half bearing three strong leaf-like spines at apex and numerous finer setae subapically.

#### Diagnosis

**Figure 6.**
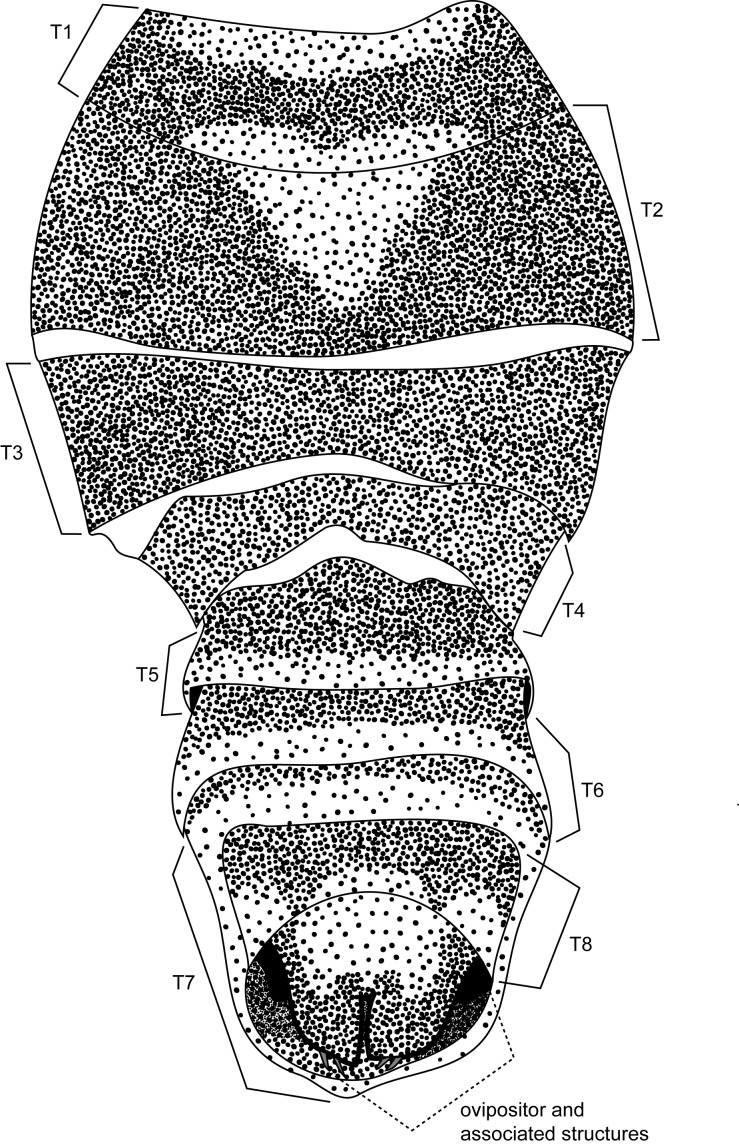
Abdomen of *P. constricta* female, showing constriction between tergites 4 and 5, the lateral extension of tergite 8 beyond the apex of the ovipositor, and the ovipositor and associated structures. The specimen imaged is from collection 205268.

This species is most closely related to *Procanace constricta* from Maui, Molokai and Hawaii. It is differentiated most readily by the shape of tergite seven. In *P. constricta* tergite seven is greatly expanded on the lateroventral margins and extends to the apices of the cerci (Fig. 6​). The seventh tergite of *P. hardyi* (Fig. 5) is not expanded on the lateroventral margins and has a telescoping form typical of the other members of this genus. The ovipositor of *P. hardyi* is long and narrow, about two times longer than wide, with distinct setae at the apices. The width and length of the cerci in *P. constricta* is roughly equal and the setae at the apices of the cerci are short and inconspicuous.

#### Etymology

It is a pleasure to dedicate this species to the memory of Dr. D. Elmo Hardy.

#### Distribution

**Figure 7.**
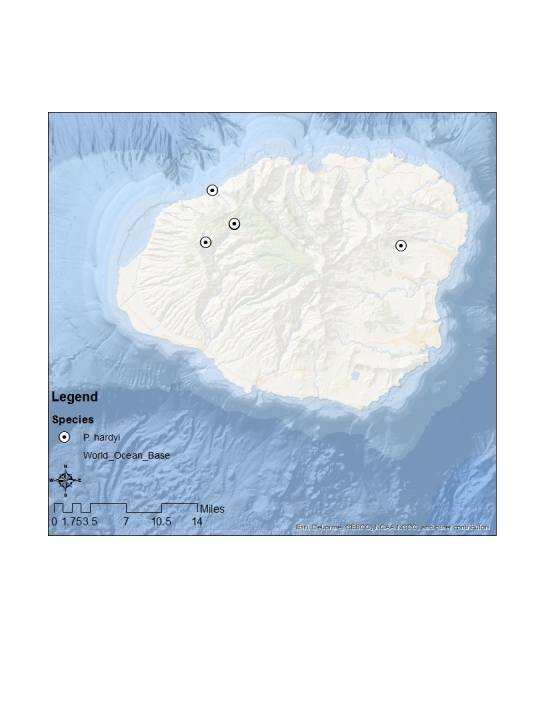
Distribution of *P. hardyi* (Kauai).

This species is endemic to Kauai (Fig. 7).

## Checklists

### Distributional Analyses of Hawaiian Canacinae (Diptera: Canacidae)

#### Canaceoides angulatus

Wirth, 1969

##### Materials

- **Type status:** Holotype. **Occurrence:** recordedBy: WW Wirth; individualCount: 1; sex: male; lifeStage: adult; **Taxon:** kingdom: Animalia; phylum: Arthropoda; class: Insecta; order: Diptera; family: Canacidae; genus: Canaceoides; specificEpithet: Canaceoidesangulatus; scientificNameAuthorship: Wirth, 1969; **Location:** islandGroup: Hawaiian Islands; island: Oahu; verbatimLocality: Waimea, on intertidal rocks; **Identification:** identifiedBy: DE Hardy & MD Delfinado; dateIdentified: 1980; **Event:** verbatimEventDate: 31.i.1946; **Record Level:** institutionCode: USNM
- **Type status:** Paratype. **Occurrence:** recordedBy: EP VanDuzee; individualCount: 1; sex: male; lifeStage: adult; **Taxon:** kingdom: Animalia; phylum: Arthropoda; class: Insecta; order: Diptera; family: Canacidae; genus: Canaceoides; specificEpithet: Canaceoidesangulatus; scientificNameAuthorship: Wirth, 1969; **Location:** waterBody: Gulf of California; island: Isla San Esteban; country: Mexico; verbatimLocality: no location given; **Identification:** identifiedBy: DE Hardy & MD Delfinado; dateIdentified: 1980; **Event:** verbatimEventDate: 20.iv.1920; **Record Level:** institutionCode: CAS, ANSP
- **Type status:** Paratype. **Occurrence:** recordedBy: EP VanDuzee; individualCount: 3; sex: female; lifeStage: adult; **Taxon:** kingdom: Animalia; phylum: Arthropoda; class: Insecta; order: Diptera; family: Canacidae; genus: Canaceoides; specificEpithet: Canaceoidesangulatus; scientificNameAuthorship: Wirth, 1969; **Location:** waterBody: Gulf of California; island: Isla San Esteban; country: Mexico; verbatimLocality: no location given; **Identification:** identifiedBy: DE Hardy & MD Delfinado; dateIdentified: 1980; **Event:** verbatimEventDate: 20.iv.1920; **Record Level:** institutionCode: CAS, ANSP
- **Type status:** Paratype. **Occurrence:** recordedBy: EH Bryan; individualCount: 1; sex: female; lifeStage: adult; **Taxon:** kingdom: Animalia; phylum: Arthropoda; class: Insecta; order: Diptera; family: Canacidae; genus: Canaceoides; specificEpithet: Canaceoidesangulatus; scientificNameAuthorship: Wirth, 1969; **Location:** islandGroup: Hawaiian Islands; island: Oahu; verbatimLocality: Wawamalu Beach near Koko Crater; **Identification:** identifiedBy: DE Hardy & MD Delfinado; dateIdentified: 1980; **Event:** verbatimEventDate: 17.xii.1922
- **Type status:** Paratype. **Occurrence:** recordedBy: C. Grant; individualCount: 1; sex: male; lifeStage: adult; **Taxon:** kingdom: Animalia; phylum: Arthropoda; class: Insecta; order: Diptera; family: Canacidae; genus: Canaceoides; specificEpithet: Canaceoidesangulatus; scientificNameAuthorship: Wirth, 1969; **Location:** islandGroup: Hawaiian Islands; island: Lisianski; verbatimLocality: no location given; **Identification:** identifiedBy: DE Hardy & MD Delfinado; dateIdentified: 1980; **Event:** verbatimEventDate: 19.v.1923; **Record Level:** institutionCode: ANSP
- **Type status:** Paratype. **Occurrence:** recordedBy: C. Grant; individualCount: 1; sex: female; lifeStage: adult; **Taxon:** kingdom: Animalia; phylum: Arthropoda; class: Insecta; order: Diptera; family: Canacidae; genus: Canaceoides; specificEpithet: Canaceoidesangulatus; scientificNameAuthorship: Wirth, 1969; **Location:** islandGroup: Hawaiian Islands; island: Lisianski; verbatimLocality: no location given; **Identification:** identifiedBy: DE Hardy & MD Delfinado; dateIdentified: 1980; **Event:** verbatimEventDate: 19.v.1923; **Record Level:** institutionCode: ANSP
- **Type status:** Paratype. **Occurrence:** recordedBy: EH Bryan; individualCount: 3; sex: female; lifeStage: adult; **Taxon:** kingdom: Animalia; phylum: Arthropoda; class: Insecta; order: Diptera; family: Canacidae; genus: Canaceoides; specificEpithet: Canaceoidesangulatus; scientificNameAuthorship: Wirth, 1969; **Location:** islandGroup: Hawaiian Islands; island: Oahu; verbatimLocality: Koko Head; **Identification:** identifiedBy: DE Hardy & MD Delfinado; dateIdentified: 1980; **Event:** verbatimEventDate: 23.vii.1923
- **Type status:** Paratype. **Occurrence:** recordedBy: WW Wirth; individualCount: 2; sex: female; lifeStage: adult; **Taxon:** kingdom: Animalia; phylum: Arthropoda; class: Insecta; order: Diptera; family: Canacidae; genus: Canaceoides; specificEpithet: Canaceoidesangulatus; scientificNameAuthorship: Wirth, 1969; **Location:** islandGroup: Hawaiian Islands; island: Hawaii; verbatimLocality: Hilo; **Identification:** identifiedBy: DE Hardy & MD Delfinado; dateIdentified: 1980; **Event:** verbatimEventDate: xii.1945
- **Type status:** Paratype. **Occurrence:** recordedBy: WW Wirth; individualCount: 1; sex: female; lifeStage: adult; **Taxon:** kingdom: Animalia; phylum: Arthropoda; class: Insecta; order: Diptera; family: Canacidae; genus: Canaceoides; specificEpithet: Canaceoidesangulatus; scientificNameAuthorship: Wirth, 1969; **Location:** islandGroup: Hawaiian Islands; island: Oahu; verbatimLocality: Lanikai; **Identification:** identifiedBy: DE Hardy & MD Delfinado; dateIdentified: 1980; **Event:** verbatimEventDate: 29.xii.1945
- **Type status:** Paratype. **Occurrence:** recordedBy: WW Wirth; individualCount: 2; sex: male; lifeStage: adult; **Taxon:** kingdom: Animalia; phylum: Arthropoda; class: Insecta; order: Diptera; family: Canacidae; genus: Canaceoides; specificEpithet: Canaceoidesangulatus; scientificNameAuthorship: Wirth, 1969; **Location:** islandGroup: Hawaiian Islands; island: Oahu; verbatimLocality: Lanikai; **Identification:** identifiedBy: DE Hardy & MD Delfinado; dateIdentified: 1980; **Event:** verbatimEventDate: 29.xii.1945
- **Type status:** Paratype. **Occurrence:** recordedBy: WW Wirth; lifeStage: adult; **Taxon:** kingdom: Animalia; phylum: Arthropoda; class: Insecta; order: Diptera; family: Canacidae; genus: Canaceoides; specificEpithet: Canaceoidesangulatus; scientificNameAuthorship: Wirth, 1969; **Location:** islandGroup: Hawaiian Islands; island: Oahu; verbatimLocality: Waimea, on intertidal rocks; **Identification:** identifiedBy: DE Hardy & MD Delfinado; dateIdentified: 1980; **Event:** verbatimEventDate: 31.i.1946
- **Type status:** Paratype. **Occurrence:** recordedBy: WW Wirth; individualCount: 7; sex: male; lifeStage: adult; **Taxon:** kingdom: Animalia; phylum: Arthropoda; class: Insecta; order: Diptera; family: Canacidae; genus: Canaceoides; specificEpithet: Canaceoidesangulatus; scientificNameAuthorship: Wirth, 1969; **Location:** islandGroup: Hawaiian Islands; island: Oahu; verbatimLocality: Waimea, on intertidal rocks; **Identification:** identifiedBy: DE Hardy & MD Delfinado; dateIdentified: 1980; **Event:** verbatimEventDate: 31.i.1946
- **Type status:** Paratype. **Occurrence:** recordedBy: WW Wirth; individualCount: 12; sex: female; lifeStage: adult; **Taxon:** kingdom: Animalia; phylum: Arthropoda; class: Insecta; order: Diptera; family: Canacidae; genus: Canaceoides; specificEpithet: Canaceoidesangulatus; scientificNameAuthorship: Wirth, 1969; **Location:** islandGroup: Hawaiian Islands; island: Oahu; verbatimLocality: Waimea, on intertidal rocks; **Identification:** identifiedBy: DE Hardy & MD Delfinado; dateIdentified: 1980; **Event:** verbatimEventDate: 31.i.1946
- **Type status:** Paratype. **Occurrence:** recordedBy: WW Wirth; individualCount: 1; sex: male; lifeStage: adult; **Taxon:** kingdom: Animalia; phylum: Arthropoda; class: Insecta; order: Diptera; family: Canacidae; genus: Canaceoides; specificEpithet: Canaceoidesangulatus; scientificNameAuthorship: Wirth, 1969; **Location:** islandGroup: Hawaiian Islands; island: Oahu; verbatimLocality: Sand Island, light trap; **Identification:** identifiedBy: DE Hardy & MD Delfinado; dateIdentified: 1980; **Event:** verbatimEventDate: 22.v.1946
- **Type status:** Paratype. **Occurrence:** recordedBy: WW Wirth; individualCount: 2; sex: female; lifeStage: adult; **Taxon:** kingdom: Animalia; phylum: Arthropoda; class: Insecta; order: Diptera; family: Canacidae; genus: Canaceoides; specificEpithet: Canaceoidesangulatus; scientificNameAuthorship: Wirth, 1969; **Location:** islandGroup: Hawaiian Islands; island: Oahu; verbatimLocality: Koko Head; **Identification:** identifiedBy: DE Hardy & MD Delfinado; dateIdentified: 1980; **Event:** verbatimEventDate: 25.vi.1946
- **Type status:** Paratype. **Occurrence:** recordedBy: WW Wirth; individualCount: 1; sex: male; lifeStage: adult; **Taxon:** kingdom: Animalia; phylum: Arthropoda; class: Insecta; order: Diptera; family: Canacidae; genus: Canaceoides; specificEpithet: Canaceoidesangulatus; scientificNameAuthorship: Wirth, 1969; **Location:** islandGroup: Hawaiian Islands; island: Oahu; verbatimLocality: Rabbit Island; **Identification:** identifiedBy: DE Hardy & MD Delfinado; dateIdentified: 1980; **Event:** verbatimEventDate: 30.viii.1946
- **Type status:** Paratype. **Occurrence:** recordedBy: WW Wirth; individualCount: 2; sex: female; lifeStage: adult; **Taxon:** kingdom: Animalia; phylum: Arthropoda; class: Insecta; order: Diptera; family: Canacidae; genus: Canaceoides; specificEpithet: Canaceoidesangulatus; scientificNameAuthorship: Wirth, 1969; **Location:** islandGroup: Hawaiian Islands; island: Oahu; verbatimLocality: Rabbit Island; **Identification:** identifiedBy: DE Hardy & MD Delfinado; dateIdentified: 1980; **Event:** verbatimEventDate: 30.viii.1946
- **Type status:** Paratype. **Occurrence:** recordedBy: WW Wirth; individualCount: 1; sex: female; lifeStage: adult; **Taxon:** kingdom: Animalia; phylum: Arthropoda; class: Insecta; order: Diptera; family: Canacidae; genus: Canaceoides; specificEpithet: Canaceoidesangulatus; scientificNameAuthorship: Wirth, 1969; **Location:** islandGroup: Hawaiian Islands; island: Kauai; verbatimLocality: Kilauea; **Identification:** identifiedBy: DE Hardy & MD Delfinado; dateIdentified: 1980; **Event:** verbatimEventDate: 8.ix.1946
- **Type status:** Paratype. **Occurrence:** recordedBy: WW Wirth; individualCount: 1; sex: male; lifeStage: adult; **Taxon:** kingdom: Animalia; phylum: Arthropoda; class: Insecta; order: Diptera; family: Canacidae; genus: Canaceoides; specificEpithet: Canaceoidesangulatus; scientificNameAuthorship: Wirth, 1969; **Location:** islandGroup: Hawaiian Islands; island: Kauai; verbatimLocality: Nawiliwili; **Identification:** identifiedBy: DE Hardy & MD Delfinado; dateIdentified: 1980; **Event:** verbatimEventDate: 9.ix.1946
- **Type status:** Paratype. **Occurrence:** recordedBy: WW Wirth; individualCount: 2; sex: female; lifeStage: adult; **Taxon:** kingdom: Animalia; phylum: Arthropoda; class: Insecta; order: Diptera; family: Canacidae; genus: Canaceoides; specificEpithet: Canaceoidesangulatus; scientificNameAuthorship: Wirth, 1969; **Location:** islandGroup: Hawaiian Islands; island: Kauai; verbatimLocality: Nawiliwili; **Identification:** identifiedBy: DE Hardy & MD Delfinado; dateIdentified: 1980; **Event:** verbatimEventDate: 9.ix.1946
- **Type status:** Paratype. **Occurrence:** recordedBy: DE Hardy; individualCount: 28; sex: female; lifeStage: adult; **Taxon:** kingdom: Animalia; phylum: Arthropoda; class: Insecta; order: Diptera; family: Canacidae; genus: Canaceoides; specificEpithet: Canaceoidesangulatus; scientificNameAuthorship: Wirth, 1969; **Location:** islandGroup: Hawaiian Islands; island: Molokai; verbatimLocality: Waialua; **Identification:** identifiedBy: DE Hardy & MD Delfinado; dateIdentified: 1980; **Event:** verbatimEventDate: vii.1952; **Record Level:** institutionCode: UHM
- **Type status:** Paratype. **Occurrence:** recordedBy: DE Hardy; individualCount: 30; sex: male; lifeStage: adult; **Taxon:** kingdom: Animalia; phylum: Arthropoda; class: Insecta; order: Diptera; family: Canacidae; genus: Canaceoides; specificEpithet: Canaceoidesangulatus; scientificNameAuthorship: Wirth, 1969; **Location:** islandGroup: Hawaiian Islands; island: Molokai; verbatimLocality: Waialua; **Identification:** identifiedBy: DE Hardy & MD Delfinado; dateIdentified: 1980; **Event:** verbatimEventDate: vii.1952; **Record Level:** institutionCode: UHM
- **Type status:** Paratype. **Occurrence:** recordedBy: PH Arnauld; individualCount: 12; sex: male; lifeStage: adult; **Taxon:** kingdom: Animalia; phylum: Arthropoda; class: Insecta; order: Diptera; family: Canacidae; genus: Canaceoides; specificEpithet: Canaceoidesangulatus; scientificNameAuthorship: Wirth, 1969; **Location:** waterBody: Gulf of California; island: Isla Cerralvo; country: Mexico; verbatimLocality: Gordas Point; **Identification:** identifiedBy: DE Hardy & MD Delfinado; dateIdentified: 1980; **Event:** verbatimEventDate: 20.iii.1953; eventRemarks: Sefton-Orca Expedition; **Record Level:** institutionCode: CAS
- **Type status:** Paratype. **Occurrence:** recordedBy: PH Arnauld; individualCount: 13; sex: female; lifeStage: adult; **Taxon:** kingdom: Animalia; phylum: Arthropoda; class: Insecta; order: Diptera; family: Canacidae; genus: Canaceoides; specificEpithet: Canaceoidesangulatus; scientificNameAuthorship: Wirth, 1969; **Location:** waterBody: Gulf of California; island: Isla Cerralvo; country: Mexico; verbatimLocality: Gordas Point; **Identification:** identifiedBy: DE Hardy & MD Delfinado; dateIdentified: 1980; **Event:** verbatimEventDate: 20.iii.1953; eventRemarks: Sefton-Orca Expedition; **Record Level:** institutionCode: CAS
- **Type status:** Paratype. **Occurrence:** recordedBy: PH Arnauld; individualCount: 2; sex: male; lifeStage: adult; **Taxon:** kingdom: Animalia; phylum: Arthropoda; class: Insecta; order: Diptera; family: Canacidae; genus: Canaceoides; specificEpithet: Canaceoidesangulatus; scientificNameAuthorship: Wirth, 1969; **Location:** waterBody: Gulf of California; island: Isla Partida; country: Mexico; verbatimLocality: no location given; **Identification:** identifiedBy: DE Hardy & MD Delfinado; dateIdentified: 1980; **Event:** verbatimEventDate: 23.iii.1953; eventRemarks: Sefton-Orca Expedition; **Record Level:** institutionCode: CAS
- **Type status:** Paratype. **Occurrence:** recordedBy: PH Arnauld; individualCount: 11; sex: female; lifeStage: adult; **Taxon:** kingdom: Animalia; phylum: Arthropoda; class: Insecta; order: Diptera; family: Canacidae; genus: Canaceoides; specificEpithet: Canaceoidesangulatus; scientificNameAuthorship: Wirth, 1969; **Location:** waterBody: Gulf of California; island: Isla Partida; country: Mexico; verbatimLocality: no location given; **Identification:** identifiedBy: DE Hardy & MD Delfinado; dateIdentified: 1980; **Event:** verbatimEventDate: 23.iii.1953; eventRemarks: Sefton-Orca Expedition; **Record Level:** institutionCode: CAS
- **Type status:** Paratype. **Occurrence:** recordedBy: PH Arnauld; individualCount: 20; sex: female; lifeStage: adult; **Taxon:** kingdom: Animalia; phylum: Arthropoda; class: Insecta; order: Diptera; family: Canacidae; genus: Canaceoides; specificEpithet: Canaceoidesangulatus; scientificNameAuthorship: Wirth, 1969; **Location:** waterBody: Gulf of California; island: Isla San Francisco; country: Mexico; verbatimLocality: no location given; **Identification:** identifiedBy: DE Hardy & MD Delfinado; dateIdentified: 1980; **Event:** verbatimEventDate: 24.iii.1953; eventRemarks: Sefton-Orca Expedition; **Record Level:** institutionCode: CAS
- **Type status:** Paratype. **Occurrence:** recordedBy: PH Arnauld; individualCount: 34; sex: male; lifeStage: adult; **Taxon:** kingdom: Animalia; phylum: Arthropoda; class: Insecta; order: Diptera; family: Canacidae; genus: Canaceoides; specificEpithet: Canaceoidesangulatus; scientificNameAuthorship: Wirth, 1969; **Location:** waterBody: Gulf of California; island: Isla San Francisco; country: Mexico; verbatimLocality: no location given; **Identification:** identifiedBy: DE Hardy & MD Delfinado; dateIdentified: 1980; **Event:** verbatimEventDate: 24.iii.1953; eventRemarks: Sefton-Orca Expedition; **Record Level:** institutionCode: CAS
- **Type status:** Paratype. **Occurrence:** recordedBy: PH Arnauld; individualCount: 3; sex: female; lifeStage: adult; **Taxon:** kingdom: Animalia; phylum: Arthropoda; class: Insecta; order: Diptera; family: Canacidae; genus: Canaceoides; specificEpithet: Canaceoidesangulatus; scientificNameAuthorship: Wirth, 1969; **Location:** country: Mexico; stateProvince: Baja California Sur; verbatimLocality: Agua Verde Bay; **Identification:** identifiedBy: DE Hardy & MD Delfinado; dateIdentified: 1980; **Event:** verbatimEventDate: 26.iii.1953; eventRemarks: Sefton-Orca Expedition; **Record Level:** institutionCode: CAS
- **Type status:** Paratype. **Occurrence:** recordedBy: PH Arnauld; individualCount: 1; sex: male; lifeStage: adult; **Taxon:** kingdom: Animalia; phylum: Arthropoda; class: Insecta; order: Diptera; family: Canacidae; genus: Canaceoides; specificEpithet: Canaceoidesangulatus; scientificNameAuthorship: Wirth, 1969; **Location:** country: Mexico; stateProvince: Baja California Sur; verbatimLocality: Agua Verde Bay; **Identification:** identifiedBy: DE Hardy & MD Delfinado; dateIdentified: 1980; **Event:** verbatimEventDate: 26.iii.1953; eventRemarks: Sefton-Orca Expedition; **Record Level:** institutionCode: CAS
- **Type status:** Paratype. **Occurrence:** recordedBy: PH Arnauld; individualCount: 8; sex: male; lifeStage: adult; **Taxon:** kingdom: Animalia; phylum: Arthropoda; class: Insecta; order: Diptera; family: Canacidae; genus: Canaceoides; specificEpithet: Canaceoidesangulatus; scientificNameAuthorship: Wirth, 1969; **Location:** waterBody: Gulf of California; island: Isla Ildefonso; country: Mexico; verbatimLocality: no location given; **Identification:** identifiedBy: DE Hardy & MD Delfinado; dateIdentified: 1980; **Event:** verbatimEventDate: 30.iii.1953; eventRemarks: Sefton-Orca Expedition; **Record Level:** institutionCode: CAS
- **Type status:** Paratype. **Occurrence:** recordedBy: PH Arnauld; individualCount: 10; sex: female; lifeStage: adult; **Taxon:** kingdom: Animalia; phylum: Arthropoda; class: Insecta; order: Diptera; family: Canacidae; genus: Canaceoides; specificEpithet: Canaceoidesangulatus; scientificNameAuthorship: Wirth, 1969; **Location:** waterBody: Gulf of California; island: Isla Ildefonso; country: Mexico; verbatimLocality: no location given; **Identification:** identifiedBy: DE Hardy & MD Delfinado; dateIdentified: 1980; **Event:** verbatimEventDate: 30.iii.1953; eventRemarks: Sefton-Orca Expedition; **Record Level:** institutionCode: CAS
- **Type status:** Paratype. **Occurrence:** recordedBy: PH Arnauld; individualCount: 34; sex: female; lifeStage: adult; **Taxon:** kingdom: Animalia; phylum: Arthropoda; class: Insecta; order: Diptera; family: Canacidae; genus: Canaceoides; specificEpithet: Canaceoidesangulatus; scientificNameAuthorship: Wirth, 1969; **Location:** country: Mexico; stateProvince: Baja California Norte; verbatimLocality: San Felipe; **Identification:** identifiedBy: DE Hardy & MD Delfinado; dateIdentified: 1980; **Event:** verbatimEventDate: 19.ii.1954; **Record Level:** institutionCode: CAS
- **Type status:** Paratype. **Occurrence:** recordedBy: PH Arnauld; individualCount: 19; sex: male; lifeStage: adult; **Taxon:** kingdom: Animalia; phylum: Arthropoda; class: Insecta; order: Diptera; family: Canacidae; genus: Canaceoides; specificEpithet: Canaceoidesangulatus; scientificNameAuthorship: Wirth, 1969; **Location:** country: Mexico; stateProvince: Baja California Norte; verbatimLocality: San Felipe; **Identification:** identifiedBy: DE Hardy & MD Delfinado; dateIdentified: 1980; **Event:** verbatimEventDate: 19.ii.1954; **Record Level:** institutionCode: CAS
- **Type status:** Paratype. **Occurrence:** recordedBy: EI Schlinger, ES Ross; individualCount: 12; sex: male; lifeStage: adult; **Taxon:** kingdom: Animalia; phylum: Arthropoda; class: Insecta; order: Diptera; family: Canacidae; genus: Canaceoides; specificEpithet: Canaceoidesangulatus; scientificNameAuthorship: Wirth, 1969; **Location:** country: Peru; verbatimLocality: Lima, 3km northwest Canete; **Identification:** identifiedBy: DE Hardy & MD Delfinado; dateIdentified: 1980; **Event:** verbatimEventDate: 13.ix.1954; **Record Level:** institutionCode: CAS
- **Type status:** Paratype. **Occurrence:** recordedBy: EI Schlinger, ES Ross; individualCount: 10; sex: female; lifeStage: adult; **Taxon:** kingdom: Animalia; phylum: Arthropoda; class: Insecta; order: Diptera; family: Canacidae; genus: Canaceoides; specificEpithet: Canaceoidesangulatus; scientificNameAuthorship: Wirth, 1969; **Location:** country: Peru; verbatimLocality: Lima, 3km northwest Canete; **Identification:** identifiedBy: DE Hardy & MD Delfinado; dateIdentified: 1980; **Event:** verbatimEventDate: 13.ix.1954; **Record Level:** institutionCode: CAS
- **Type status:** Paratype. **Occurrence:** recordedBy: DE Hardy; individualCount: 2; sex: male; lifeStage: adult; **Taxon:** kingdom: Animalia; phylum: Arthropoda; class: Insecta; order: Diptera; family: Canacidae; genus: Canaceoides; specificEpithet: Canaceoidesangulatus; scientificNameAuthorship: Wirth, 1969; **Location:** islandGroup: Hawaiian Islands; island: Oahu; verbatimLocality: Hauula; **Identification:** identifiedBy: DE Hardy & MD Delfinado; dateIdentified: 1980; **Event:** verbatimEventDate: 4.vii.1955
- **Type status:** Paratype. **Occurrence:** recordedBy: no collector given; individualCount: 1; sex: male; lifeStage: adult; **Taxon:** kingdom: Animalia; phylum: Arthropoda; class: Insecta; order: Diptera; family: Canacidae; genus: Canaceoides; specificEpithet: Canaceoidesangulatus; scientificNameAuthorship: Wirth, 1969; **Location:** islandGroup: Hawaiian Islands; island: Midway; verbatimLocality: at light; **Identification:** identifiedBy: DE Hardy & MD Delfinado; dateIdentified: 1980; **Event:** verbatimEventDate: 12.xi.1959
- **Type status:** Paratype. **Occurrence:** recordedBy: PH Arnauld, E Ross, Rentz; individualCount: 11; sex: female; lifeStage: adult; **Taxon:** kingdom: Animalia; phylum: Arthropoda; class: Insecta; order: Diptera; family: Canacidae; genus: Canaceoides; specificEpithet: Canaceoidesangulatus; scientificNameAuthorship: Wirth, 1969; **Location:** country: Mexico; stateProvince: Sonora; verbatimLocality: San Carlos Bay; **Identification:** identifiedBy: DE Hardy & MD Delfinado; dateIdentified: 1980; **Event:** verbatimEventDate: 10.viii.1960; **Record Level:** institutionCode: CAS
- **Type status:** Paratype. **Occurrence:** recordedBy: PH Arnauld, E Ross, Rentz; individualCount: 9; sex: male; lifeStage: adult; **Taxon:** kingdom: Animalia; phylum: Arthropoda; class: Insecta; order: Diptera; family: Canacidae; genus: Canaceoides; specificEpithet: Canaceoidesangulatus; scientificNameAuthorship: Wirth, 1969; **Location:** country: Mexico; stateProvince: Sonora; verbatimLocality: San Carlos Bay; **Identification:** identifiedBy: DE Hardy & MD Delfinado; dateIdentified: 1980; **Event:** verbatimEventDate: 10.viii.1960; **Record Level:** institutionCode: CAS
- **Type status:** Paratype. **Occurrence:** recordedBy: GD Butler; individualCount: 1; sex: female; lifeStage: adult; **Taxon:** kingdom: Animalia; phylum: Arthropoda; class: Insecta; order: Diptera; family: Canacidae; genus: Canaceoides; specificEpithet: Canaceoidesangulatus; scientificNameAuthorship: Wirth, 1969; **Location:** islandGroup: Hawaiian Islands; island: Laysan; verbatimLocality: no location given; **Identification:** identifiedBy: DE Hardy & MD Delfinado; dateIdentified: 1980; **Event:** verbatimEventDate: ix.1961; **Record Level:** institutionCode: UHM
- **Type status:** Paratype. **Occurrence:** recordedBy: Ryckman, Ryckman, Christianson; individualCount: 10; sex: male; lifeStage: adult; **Taxon:** kingdom: Animalia; phylum: Arthropoda; class: Insecta; order: Diptera; family: Canacidae; genus: Canaceoides; specificEpithet: Canaceoidesangulatus; scientificNameAuthorship: Wirth, 1969; **Location:** waterBody: Gulf of California; island: Isla Salsipuedes; country: Mexico; verbatimLocality: no location given; **Identification:** identifiedBy: DE Hardy & MD Delfinado; dateIdentified: 1980; **Event:** verbatimEventDate: 20.v.1962
- **Type status:** Paratype. **Occurrence:** recordedBy: Ryckman, Ryckman, Christianson; individualCount: 6; sex: female; lifeStage: adult; **Taxon:** kingdom: Animalia; phylum: Arthropoda; class: Insecta; order: Diptera; family: Canacidae; genus: Canaceoides; specificEpithet: Canaceoidesangulatus; scientificNameAuthorship: Wirth, 1969; **Location:** waterBody: Gulf of California; island: Isla Salsipuedes; country: Mexico; verbatimLocality: no location given; **Identification:** identifiedBy: DE Hardy & MD Delfinado; dateIdentified: 1980; **Event:** verbatimEventDate: 20.v.1962
- **Type status:** Paratype. **Occurrence:** recordedBy: DQ Cavagnaro, RO Schuster; individualCount: 1; sex: female; lifeStage: adult; **Taxon:** kingdom: Animalia; phylum: Arthropoda; class: Insecta; order: Diptera; family: Canacidae; genus: Canaceoides; specificEpithet: Canaceoidesangulatus; scientificNameAuthorship: Wirth, 1969; **Location:** islandGroup: Galapagos Archipelago; island: Isla Santa Cruz; country: Ecuador; verbatimLocality: Academy Bay; **Identification:** identifiedBy: DE Hardy & MD Delfinado; dateIdentified: 1980; **Event:** verbatimEventDate: 24.i.1964; **Record Level:** institutionCode: CAS
- **Type status:** Paratype. **Occurrence:** recordedBy: T Pappenfuss; individualCount: 11; sex: female; lifeStage: adult; **Taxon:** kingdom: Animalia; phylum: Arthropoda; class: Insecta; order: Diptera; family: Canacidae; genus: Canaceoides; specificEpithet: Canaceoidesangulatus; scientificNameAuthorship: Wirth, 1969; **Location:** islandGroup: Galapagos Archipelago; island: Isla Santa Fe; country: Ecuador; verbatimLocality: no location given; **Identification:** identifiedBy: DE Hardy & MD Delfinado; dateIdentified: 1980; **Event:** verbatimEventDate: 5.ii.1964; **Record Level:** institutionCode: CAS
- **Type status:** Paratype. **Occurrence:** recordedBy: PD Ashlock; individualCount: 1; sex: male; lifeStage: adult; **Taxon:** kingdom: Animalia; phylum: Arthropoda; class: Insecta; order: Diptera; family: Canacidae; genus: Canaceoides; specificEpithet: Canaceoidesangulatus; scientificNameAuthorship: Wirth, 1969; **Location:** islandGroup: Galapagos Archipelago; island: Isla Santa Cruz; country: Ecuador; verbatimLocality: Academy Bay, at light; **Identification:** identifiedBy: DE Hardy & MD Delfinado; dateIdentified: 1980; **Event:** verbatimEventDate: 17.ii.1964; **Record Level:** institutionCode: BPBM
- **Type status:** Paratype. **Occurrence:** recordedBy: PD Ashlock; individualCount: 1; sex: female; lifeStage: adult; **Taxon:** kingdom: Animalia; phylum: Arthropoda; class: Insecta; order: Diptera; family: Canacidae; genus: Canaceoides; specificEpithet: Canaceoidesangulatus; scientificNameAuthorship: Wirth, 1969; **Location:** islandGroup: Galapagos Archipelago; island: Isla Santa Cruz; country: Ecuador; verbatimLocality: Academy Bay, at light; **Identification:** identifiedBy: DE Hardy & MD Delfinado; dateIdentified: 1980; **Event:** verbatimEventDate: 17.ii.1964; **Record Level:** institutionCode: BPBM
- **Type status:** Paratype. **Occurrence:** recordedBy: DQ Cavagnaro; individualCount: 22; sex: male; lifeStage: adult; **Taxon:** kingdom: Animalia; phylum: Arthropoda; class: Insecta; order: Diptera; family: Canacidae; genus: Canaceoides; specificEpithet: Canaceoidesangulatus; scientificNameAuthorship: Wirth, 1969; **Location:** islandGroup: Galapagos Archipelago; island: Isla Pinta; country: Ecuador; verbatimLocality: southeast coast; **Identification:** identifiedBy: DE Hardy & MD Delfinado; dateIdentified: 1980; **Event:** verbatimEventDate: 25.v.1964; **Record Level:** institutionCode: CAS
- **Type status:** Paratype. **Occurrence:** recordedBy: DQ Cavagnaro; individualCount: 24; sex: female; lifeStage: adult; **Taxon:** kingdom: Animalia; phylum: Arthropoda; class: Insecta; order: Diptera; family: Canacidae; genus: Canaceoides; specificEpithet: Canaceoidesangulatus; scientificNameAuthorship: Wirth, 1969; **Location:** islandGroup: Galapagos Archipelago; island: Isla Pinta; country: Ecuador; verbatimLocality: southeast coast; **Identification:** identifiedBy: DE Hardy & MD Delfinado; dateIdentified: 1980; **Event:** verbatimEventDate: 25.v.1964; **Record Level:** institutionCode: CAS
- **Type status:** Paratype. **Occurrence:** recordedBy: JR Vockeroth; individualCount: 47; sex: female; lifeStage: adult; **Taxon:** kingdom: Animalia; phylum: Arthropoda; class: Insecta; order: Diptera; family: Canacidae; genus: Canaceoides; specificEpithet: Canaceoidesangulatus; scientificNameAuthorship: Wirth, 1969; **Location:** islandGroup: Hawaiian Islands; island: Oahu; verbatimLocality: Honolulu, wave swept rocks; **Identification:** identifiedBy: DE Hardy & MD Delfinado; dateIdentified: 1980; **Event:** verbatimEventDate: 25.ix.1966 and 20.xi.1966; **Record Level:** institutionCode: CNC
- **Type status:** Paratype. **Occurrence:** recordedBy: JR Vockeroth; individualCount: 40; sex: male; lifeStage: adult; **Taxon:** kingdom: Animalia; phylum: Arthropoda; class: Insecta; order: Diptera; family: Canacidae; genus: Canaceoides; specificEpithet: Canaceoidesangulatus; scientificNameAuthorship: Wirth, 1969; **Location:** islandGroup: Hawaiian Islands; island: Oahu; verbatimLocality: Honolulu, wave swept rocks; **Identification:** identifiedBy: DE Hardy & MD Delfinado; dateIdentified: 1980; **Event:** verbatimEventDate: 25.ix.1966 and 20.xi.1966; **Record Level:** institutionCode: CNC
- **Type status:** Paratype. **Occurrence:** recordedBy: W. Voss; individualCount: 21; sex: male; lifeStage: adult; **Taxon:** kingdom: Animalia; phylum: Arthropoda; class: Insecta; order: Diptera; family: Canacidae; genus: Canaceoides; specificEpithet: Canaceoidesangulatus; scientificNameAuthorship: Wirth, 1969; **Location:** islandGroup: Hawaiian Islands; island: Hawaii; verbatimLocality: Kailua-Kona; **Identification:** identifiedBy: DE Hardy & MD Delfinado; dateIdentified: 1980; **Event:** verbatimEventDate: 2.x.1966; **Record Level:** institutionCode: CNC
- **Type status:** Paratype. **Occurrence:** recordedBy: W. Voss; individualCount: 31; sex: female; lifeStage: adult; **Taxon:** kingdom: Animalia; phylum: Arthropoda; class: Insecta; order: Diptera; family: Canacidae; genus: Canaceoides; specificEpithet: Canaceoidesangulatus; scientificNameAuthorship: Wirth, 1969; **Location:** islandGroup: Hawaiian Islands; island: Hawaii; verbatimLocality: Kailua-Kona; **Identification:** identifiedBy: DE Hardy & MD Delfinado; dateIdentified: 1980; **Event:** verbatimEventDate: 2.x.1966; **Record Level:** institutionCode: CNC
- **Type status:** Paratype. **Occurrence:** recordedBy: T. Saigusa; individualCount: 2; sex: female; lifeStage: adult; **Taxon:** kingdom: Animalia; phylum: Arthropoda; class: Insecta; order: Diptera; family: Canacidae; genus: Canaceoides; specificEpithet: Canaceoidesangulatus; scientificNameAuthorship: Wirth, 1969; **Location:** islandGroup: Hawaiian Islands; island: Maui; verbatimLocality: Honokowai; **Identification:** identifiedBy: DE Hardy & MD Delfinado; dateIdentified: 1980; **Event:** verbatimEventDate: 28.x.1966; **Record Level:** institutionCode: CNC
- **Type status:** Paratype. **Occurrence:** recordedBy: T. Saigusa; individualCount: 1; sex: male; lifeStage: adult; **Taxon:** kingdom: Animalia; phylum: Arthropoda; class: Insecta; order: Diptera; family: Canacidae; genus: Canaceoides; specificEpithet: Canaceoidesangulatus; scientificNameAuthorship: Wirth, 1969; **Location:** islandGroup: Hawaiian Islands; island: Maui; verbatimLocality: Kihei; **Identification:** identifiedBy: DE Hardy & MD Delfinado; dateIdentified: 1980; **Event:** verbatimEventDate: 28.x.1966; **Record Level:** institutionCode: CNC
- **Type status:** Paratype. **Occurrence:** recordedBy: T. Saigusa; individualCount: 1; sex: female; lifeStage: adult; **Taxon:** kingdom: Animalia; phylum: Arthropoda; class: Insecta; order: Diptera; family: Canacidae; genus: Canaceoides; specificEpithet: Canaceoidesangulatus; scientificNameAuthorship: Wirth, 1969; **Location:** islandGroup: Hawaiian Islands; island: Maui; verbatimLocality: Kihei; **Identification:** identifiedBy: DE Hardy & MD Delfinado; dateIdentified: 1980; **Event:** verbatimEventDate: 28.x.1966; **Record Level:** institutionCode: CNC
- **Type status:** Paratype. **Occurrence:** recordedBy: T. Saigusa; individualCount: 2; sex: male; lifeStage: adult; **Taxon:** kingdom: Animalia; phylum: Arthropoda; class: Insecta; order: Diptera; family: Canacidae; genus: Canaceoides; specificEpithet: Canaceoidesangulatus; scientificNameAuthorship: Wirth, 1969; **Location:** islandGroup: Hawaiian Islands; island: Maui; verbatimLocality: Honokowai; **Identification:** identifiedBy: DE Hardy & MD Delfinado; dateIdentified: 1980; **Event:** verbatimEventDate: 28.x.1966; **Record Level:** institutionCode: CNC
- **Type status:** Paratype. **Occurrence:** recordedBy: T. Saigusa; individualCount: 2; sex: female; lifeStage: adult; **Taxon:** kingdom: Animalia; phylum: Arthropoda; class: Insecta; order: Diptera; family: Canacidae; genus: Canaceoides; specificEpithet: Canaceoidesangulatus; scientificNameAuthorship: Wirth, 1969; **Location:** islandGroup: Hawaiian Islands; island: Maui; verbatimLocality: McGregor Point; **Identification:** identifiedBy: DE Hardy & MD Delfinado; dateIdentified: 1980; **Event:** verbatimEventDate: 28.x.1966; **Record Level:** institutionCode: CNC
- **Type status:** Paratype. **Occurrence:** recordedBy: JR Vockeroth; individualCount: 5; sex: female; lifeStage: adult; **Taxon:** kingdom: Animalia; phylum: Arthropoda; class: Insecta; order: Diptera; family: Canacidae; genus: Canaceoides; specificEpithet: Canaceoidesangulatus; scientificNameAuthorship: Wirth, 1969; **Location:** islandGroup: Hawaiian Islands; island: Hawaii; verbatimLocality: Hilo; **Identification:** identifiedBy: DE Hardy & MD Delfinado; dateIdentified: 1980; **Event:** verbatimEventDate: 23.iii.1967; **Record Level:** institutionCode: CNC
- **Type status:** Paratype. **Occurrence:** recordedBy: JR Vockeroth; individualCount: 3; sex: male; lifeStage: adult; **Taxon:** kingdom: Animalia; phylum: Arthropoda; class: Insecta; order: Diptera; family: Canacidae; genus: Canaceoides; specificEpithet: Canaceoidesangulatus; scientificNameAuthorship: Wirth, 1969; **Location:** islandGroup: Hawaiian Islands; island: Hawaii; verbatimLocality: Hilo; **Identification:** identifiedBy: DE Hardy & MD Delfinado; dateIdentified: 1980; **Event:** verbatimEventDate: 23.iii.1967; **Record Level:** institutionCode: CNC
- **Type status:** Paratype. **Occurrence:** recordedBy: JR Vockeroth; individualCount: 10; sex: female; lifeStage: adult; **Taxon:** kingdom: Animalia; phylum: Arthropoda; class: Insecta; order: Diptera; family: Canacidae; genus: Canaceoides; specificEpithet: Canaceoidesangulatus; scientificNameAuthorship: Wirth, 1969; **Location:** islandGroup: Hawaiian Islands; island: Hawaii; verbatimLocality: Kalapana Park; **Identification:** identifiedBy: DE Hardy & MD Delfinado; dateIdentified: 1980; **Event:** verbatimEventDate: 24.iii.1967; **Record Level:** institutionCode: CNC
- **Type status:** Paratype. **Occurrence:** recordedBy: JR Vockeroth; individualCount: 10; sex: male; lifeStage: adult; **Taxon:** kingdom: Animalia; phylum: Arthropoda; class: Insecta; order: Diptera; family: Canacidae; genus: Canaceoides; specificEpithet: Canaceoidesangulatus; scientificNameAuthorship: Wirth, 1969; **Location:** islandGroup: Hawaiian Islands; island: Hawaii; verbatimLocality: Kalapana Park; **Identification:** identifiedBy: DE Hardy & MD Delfinado; dateIdentified: 1980; **Event:** verbatimEventDate: 24.iii.1967; **Record Level:** institutionCode: CNC
- **Type status:** Other material. **Occurrence:** catalogNumber: 2006004911; recordedBy: Terry; lifeStage: adult; **Taxon:** kingdom: Animalia; phylum: Arthropoda; class: Insecta; order: Diptera; family: Canacidae; genus: Canaceoides; specificEpithet: Canaceoidesangulatus; scientificNameAuthorship: Wirth, 1969; **Location:** islandGroup: Hawaiian Islands; island: Oahu; verbatimLocality: Leahi, at shore; **Identification:** identifiedBy: Cresson; **Event:** verbatimEventDate: 26.ii.1911; **Record Level:** institutionCode: BPBM
- **Type status:** Other material. **Occurrence:** catalogNumber: 2006004873; recordedBy: EH Bryan, Jr.; lifeStage: adult; **Taxon:** kingdom: Animalia; phylum: Arthropoda; class: Insecta; order: Diptera; family: Canacidae; genus: Canaceoides; specificEpithet: Canaceoidesangulatus; scientificNameAuthorship: Wirth, 1969; **Location:** islandGroup: Hawaiian Islands; island: Oahu; verbatimLocality: Koko Head; **Identification:** identifiedBy: Cresson; **Event:** verbatimEventDate: 23.vii.1922; **Record Level:** institutionCode: BPBM
- **Type status:** Other material. **Occurrence:** catalogNumber: 2006004878; recordedBy: EH Bryan, Jr.; lifeStage: adult; **Taxon:** kingdom: Animalia; phylum: Arthropoda; class: Insecta; order: Diptera; family: Canacidae; genus: Canaceoides; specificEpithet: Canaceoidesangulatus; scientificNameAuthorship: Wirth, 1969; **Location:** islandGroup: Hawaiian Islands; island: Oahu; verbatimLocality: Koko Head; **Identification:** identifiedBy: Cresson; **Event:** verbatimEventDate: 23.vii.1922; **Record Level:** institutionCode: BPBM
- **Type status:** Other material. **Occurrence:** catalogNumber: 2006004879; recordedBy: EH Bryan, Jr.; lifeStage: adult; **Taxon:** kingdom: Animalia; phylum: Arthropoda; class: Insecta; order: Diptera; family: Canacidae; genus: Canaceoides; specificEpithet: Canaceoidesangulatus; scientificNameAuthorship: Wirth, 1969; **Location:** islandGroup: Hawaiian Islands; island: Oahu; verbatimLocality: Koko Head; **Identification:** identifiedBy: Cresson; **Event:** verbatimEventDate: 23.vii.1922; **Record Level:** institutionCode: BPBM
- **Type status:** Other material. **Occurrence:** catalogNumber: 2006004880; recordedBy: EH Bryan, Jr.; lifeStage: adult; **Taxon:** kingdom: Animalia; phylum: Arthropoda; class: Insecta; order: Diptera; family: Canacidae; genus: Canaceoides; specificEpithet: Canaceoidesangulatus; scientificNameAuthorship: Wirth, 1969; **Location:** islandGroup: Hawaiian Islands; island: Oahu; verbatimLocality: Koko Head; **Identification:** identifiedBy: Cresson; **Event:** verbatimEventDate: 23.vii.1922; **Record Level:** institutionCode: BPBM
- **Type status:** Other material. **Occurrence:** catalogNumber: 2006004881; recordedBy: EH Bryan, Jr.; lifeStage: adult; **Taxon:** kingdom: Animalia; phylum: Arthropoda; class: Insecta; order: Diptera; family: Canacidae; genus: Canaceoides; specificEpithet: Canaceoidesangulatus; scientificNameAuthorship: Wirth, 1969; **Location:** islandGroup: Hawaiian Islands; island: Oahu; verbatimLocality: Koko Head; **Identification:** identifiedBy: Cresson; **Event:** verbatimEventDate: 23.vii.1922; **Record Level:** institutionCode: BPBM
- **Type status:** Other material. **Occurrence:** catalogNumber: 2006004882; recordedBy: EH Bryan, Jr.; lifeStage: adult; **Taxon:** kingdom: Animalia; phylum: Arthropoda; class: Insecta; order: Diptera; family: Canacidae; genus: Canaceoides; specificEpithet: Canaceoidesangulatus; scientificNameAuthorship: Wirth, 1969; **Location:** islandGroup: Hawaiian Islands; island: Oahu; verbatimLocality: Koko Head; **Identification:** identifiedBy: Cresson; **Event:** verbatimEventDate: 23.vii.1922; **Record Level:** institutionCode: BPBM
- **Type status:** Other material. **Occurrence:** catalogNumber: 2006004888; recordedBy: EH Bryan, Jr.; lifeStage: adult; **Taxon:** kingdom: Animalia; phylum: Arthropoda; class: Insecta; order: Diptera; family: Canacidae; genus: Canaceoides; specificEpithet: Canaceoidesangulatus; scientificNameAuthorship: Wirth, 1969; **Location:** islandGroup: Hawaiian Islands; island: Oahu; verbatimLocality: Koko Head; **Identification:** identifiedBy: Cresson; **Event:** verbatimEventDate: 23.vii.1922; **Record Level:** institutionCode: BPBM
- **Type status:** Other material. **Occurrence:** catalogNumber: 2006004895; recordedBy: EH Bryan, Jr.; lifeStage: adult; **Taxon:** kingdom: Animalia; phylum: Arthropoda; class: Insecta; order: Diptera; family: Canacidae; genus: Canaceoides; specificEpithet: Canaceoidesangulatus; scientificNameAuthorship: Wirth, 1969; **Location:** islandGroup: Hawaiian Islands; island: Oahu; verbatimLocality: Wawamalu Beach, near Koko Head; **Identification:** identifiedBy: Cresson; **Event:** verbatimEventDate: 17.xii.1922; **Record Level:** institutionCode: BPBM
- **Type status:** Other material. **Occurrence:** catalogNumber: 2006004896; recordedBy: EH Bryan, Jr.; lifeStage: adult; **Taxon:** kingdom: Animalia; phylum: Arthropoda; class: Insecta; order: Diptera; family: Canacidae; genus: Canaceoides; specificEpithet: Canaceoidesangulatus; scientificNameAuthorship: Wirth, 1969; **Location:** islandGroup: Hawaiian Islands; island: Oahu; verbatimLocality: Wawamalu Beach, near Koko Head; **Identification:** identifiedBy: Cresson; **Event:** verbatimEventDate: 17.xii.1922; **Record Level:** institutionCode: BPBM
- **Type status:** Other material. **Occurrence:** catalogNumber: 2006004897; recordedBy: EH Bryan, Jr.; lifeStage: adult; **Taxon:** kingdom: Animalia; phylum: Arthropoda; class: Insecta; order: Diptera; family: Canacidae; genus: Canaceoides; specificEpithet: Canaceoidesangulatus; scientificNameAuthorship: Wirth, 1969; **Location:** islandGroup: Hawaiian Islands; island: Oahu; verbatimLocality: Wawamalu Beach, near Koko Head; **Identification:** identifiedBy: Cresson; **Event:** verbatimEventDate: 17.xii.1922; **Record Level:** institutionCode: BPBM
- **Type status:** Other material. **Occurrence:** catalogNumber: 2006004889; recordedBy: EH Bryan, Jr.; lifeStage: adult; **Taxon:** kingdom: Animalia; phylum: Arthropoda; class: Insecta; order: Diptera; family: Canacidae; genus: Canaceoides; specificEpithet: Canaceoidesangulatus; scientificNameAuthorship: Wirth, 1969; **Location:** islandGroup: Hawaiian Islands; island: Oahu; verbatimLocality: Wawamalu Beach, near Koko Head; **Identification:** identifiedBy: Cresson; **Event:** verbatimEventDate: 17.xii.1922; **Record Level:** institutionCode: BPBM
- **Type status:** Other material. **Occurrence:** catalogNumber: 2006004890; recordedBy: EH Bryan, Jr.; lifeStage: adult; **Taxon:** kingdom: Animalia; phylum: Arthropoda; class: Insecta; order: Diptera; family: Canacidae; genus: Canaceoides; specificEpithet: Canaceoidesangulatus; scientificNameAuthorship: Wirth, 1969; **Location:** islandGroup: Hawaiian Islands; island: Oahu; verbatimLocality: Wawamalu Beach, near Koko Head; **Identification:** identifiedBy: Cresson; **Event:** verbatimEventDate: 17.xii.1922; **Record Level:** institutionCode: BPBM
- **Type status:** Other material. **Occurrence:** catalogNumber: 2006004891; recordedBy: EH Bryan, Jr.; lifeStage: adult; **Taxon:** kingdom: Animalia; phylum: Arthropoda; class: Insecta; order: Diptera; family: Canacidae; genus: Canaceoides; specificEpithet: Canaceoidesangulatus; scientificNameAuthorship: Wirth, 1969; **Location:** islandGroup: Hawaiian Islands; island: Oahu; verbatimLocality: Wawamalu Beach, near Koko Head; **Identification:** identifiedBy: Cresson; **Event:** verbatimEventDate: 17.xii.1922; **Record Level:** institutionCode: BPBM
- **Type status:** Other material. **Occurrence:** catalogNumber: 2006004892; recordedBy: EH Bryan, Jr.; lifeStage: adult; **Taxon:** kingdom: Animalia; phylum: Arthropoda; class: Insecta; order: Diptera; family: Canacidae; genus: Canaceoides; specificEpithet: Canaceoidesangulatus; scientificNameAuthorship: Wirth, 1969; **Location:** islandGroup: Hawaiian Islands; island: Oahu; verbatimLocality: Wawamalu Beach, near Koko Head; **Identification:** identifiedBy: Cresson; **Event:** verbatimEventDate: 17.xii.1922; **Record Level:** institutionCode: BPBM
- **Type status:** Other material. **Occurrence:** catalogNumber: 2006004893; recordedBy: EH Bryan, Jr.; lifeStage: adult; **Taxon:** kingdom: Animalia; phylum: Arthropoda; class: Insecta; order: Diptera; family: Canacidae; genus: Canaceoides; specificEpithet: Canaceoidesangulatus; scientificNameAuthorship: Wirth, 1969; **Location:** islandGroup: Hawaiian Islands; island: Oahu; verbatimLocality: Wawamalu Beach, near Koko Head; **Identification:** identifiedBy: Cresson; **Event:** verbatimEventDate: 17.xii.1922; **Record Level:** institutionCode: BPBM
- **Type status:** Other material. **Occurrence:** catalogNumber: 2006004894; recordedBy: EH Bryan, Jr.; lifeStage: adult; **Taxon:** kingdom: Animalia; phylum: Arthropoda; class: Insecta; order: Diptera; family: Canacidae; genus: Canaceoides; specificEpithet: Canaceoidesangulatus; scientificNameAuthorship: Wirth, 1969; **Location:** islandGroup: Hawaiian Islands; island: Oahu; verbatimLocality: Wawamalu Beach, near Koko Head; **Identification:** identifiedBy: Cresson; **Event:** verbatimEventDate: 17.xii.1922; **Record Level:** institutionCode: BPBM
- **Type status:** Other material. **Occurrence:** catalogNumber: 2006004656; recordedBy: C Grant; lifeStage: adult; **Taxon:** kingdom: Animalia; phylum: Arthropoda; class: Insecta; order: Diptera; family: Canacidae; genus: Canaceoides; specificEpithet: Canaceoidesangulatus; scientificNameAuthorship: Wirth, 1969; **Location:** islandGroup: Hawaiian Islands; island: Lisianski; **Event:** verbatimEventDate: 19.v.1923; **Record Level:** institutionCode: BPBM
- **Type status:** Other material. **Occurrence:** catalogNumber: 2008008252; recordedBy: C Grant; lifeStage: adult; **Taxon:** kingdom: Animalia; phylum: Arthropoda; class: Insecta; order: Diptera; family: Canacidae; genus: Canaceoides; specificEpithet: Canaceoidesangulatus; scientificNameAuthorship: Wirth, 1969; **Location:** islandGroup: Hawaiian Islands; island: Lisianski; **Identification:** identifiedBy: J Straxanac; dateIdentified: 1991; **Event:** verbatimEventDate: 19.v.1923; **Record Level:** institutionCode: BPBM
- **Type status:** Other material. **Occurrence:** catalogNumber: 2006004855; recordedBy: EH Bryan, Jr.; lifeStage: adult; **Taxon:** kingdom: Animalia; phylum: Arthropoda; class: Insecta; order: Diptera; family: Canacidae; genus: Canaceoides; specificEpithet: Canaceoidesangulatus; scientificNameAuthorship: Wirth, 1969; **Location:** islandGroup: Hawaiian Islands; island: Oahu; verbatimLocality: Rabbit Island; **Identification:** identifiedBy: MD Delfinado; **Event:** verbatimEventDate: 26.viii.1934; **Record Level:** institutionCode: BPBM
- **Type status:** Other material. **Occurrence:** recordedBy: MS Adachi; individualCount: 1; lifeStage: adult; **Taxon:** kingdom: Animalia; phylum: Arthropoda; class: Insecta; order: Diptera; family: Canacidae; genus: Canaceoides; specificEpithet: Canaceoidesangulatus; scientificNameAuthorship: Wirth, 1969; **Location:** islandGroup: Hawaiian Islands; island: Oahu; verbatimLocality: Waikiki; **Event:** verbatimEventDate: 16.iv.1950; **Record Level:** institutionCode: UHM
- **Type status:** Other material. **Occurrence:** recordedBy: NLH Krauss; individualCount: 8; lifeStage: adult; **Taxon:** kingdom: Animalia; phylum: Arthropoda; class: Insecta; order: Diptera; family: Canacidae; genus: Canaceoides; specificEpithet: Canaceoidesangulatus; scientificNameAuthorship: Wirth, 1969; **Location:** islandGroup: Hawaiian Islands; island: Hawaii; verbatimLocality: Kailua; **Event:** verbatimEventDate: xii.1950; **Record Level:** institutionCode: UHM
- **Type status:** Other material. **Occurrence:** recordedBy: HA Bess; individualCount: 9; lifeStage: adult; **Taxon:** kingdom: Animalia; phylum: Arthropoda; class: Insecta; order: Diptera; family: Canacidae; genus: Canaceoides; specificEpithet: Canaceoidesangulatus; scientificNameAuthorship: Wirth, 1969; **Location:** islandGroup: Hawaiian Islands; island: Hawaii; verbatimLocality: Honaunau; **Event:** verbatimEventDate: x.1951; **Record Level:** institutionCode: UHM
- **Type status:** Other material. **Occurrence:** recordedBy: MS Adachi; individualCount: 1; lifeStage: adult; **Taxon:** kingdom: Animalia; phylum: Arthropoda; class: Insecta; order: Diptera; family: Canacidae; genus: Canaceoides; specificEpithet: Canaceoidesangulatus; scientificNameAuthorship: Wirth, 1969; **Location:** islandGroup: Hawaiian Islands; island: Oahu; verbatimLocality: Ala Wai Canal; **Event:** verbatimEventDate: vi.1952; **Record Level:** institutionCode: UHM
- **Type status:** Other material. **Occurrence:** recordedBy: DE Hardy; individualCount: 2; lifeStage: adult; **Taxon:** kingdom: Animalia; phylum: Arthropoda; class: Insecta; order: Diptera; family: Canacidae; genus: Canaceoides; specificEpithet: Canaceoidesangulatus; scientificNameAuthorship: Wirth, 1969; **Location:** islandGroup: Hawaiian Islands; island: Oahu; verbatimLocality: Honolulu; **Event:** verbatimEventDate: iii.1953; **Record Level:** institutionCode: UHM
- **Type status:** Other material. **Occurrence:** recordedBy: M Tamashiro; individualCount: 3; lifeStage: adult; **Taxon:** kingdom: Animalia; phylum: Arthropoda; class: Insecta; order: Diptera; family: Canacidae; genus: Canaceoides; specificEpithet: Canaceoidesangulatus; scientificNameAuthorship: Wirth, 1969; **Location:** islandGroup: Hawaiian Islands; island: Molokai; verbatimLocality: Waialua Beach; **Event:** verbatimEventDate: vii.1953; **Record Level:** institutionCode: UHM
- **Type status:** Other material. **Occurrence:** catalogNumber: 2006004924; recordedBy: JL Gressitt; lifeStage: adult; **Taxon:** kingdom: Animalia; phylum: Arthropoda; class: Insecta; order: Diptera; family: Canacidae; genus: Canaceoides; specificEpithet: Canaceoidesangulatus; scientificNameAuthorship: Wirth, 1969; **Location:** islandGroup: Hawaiian Islands; island: Oahu; verbatimLocality: Mokumanu I.; **Event:** verbatimEventDate: 23.vi.1954; **Record Level:** institutionCode: BPBM
- **Type status:** Other material. **Occurrence:** catalogNumber: 2006004765; recordedBy: EH Bryan, Jr.; lifeStage: adult; **Taxon:** kingdom: Animalia; phylum: Arthropoda; class: Insecta; order: Diptera; family: Canacidae; genus: Canaceoides; specificEpithet: Canaceoidesangulatus; scientificNameAuthorship: Wirth, 1969; **Location:** islandGroup: Hawaiian Islands; island: Hawaii; verbatimLocality: Kona, near Puuhonua Point; **Event:** verbatimEventDate: 23.i.1957; **Record Level:** institutionCode: BPBM
- **Type status:** Other material. **Occurrence:** catalogNumber: 2006004771; recordedBy: EH Bryan, Jr.; lifeStage: adult; **Taxon:** kingdom: Animalia; phylum: Arthropoda; class: Insecta; order: Diptera; family: Canacidae; genus: Canaceoides; specificEpithet: Canaceoidesangulatus; scientificNameAuthorship: Wirth, 1969; **Location:** islandGroup: Hawaiian Islands; island: Hawaii; verbatimLocality: Kona, near Puuhonua Point; **Event:** verbatimEventDate: 23.i.1957; **Record Level:** institutionCode: BPBM
- **Type status:** Other material. **Occurrence:** recordedBy: Y Kondoh; individualCount: 1; lifeStage: adult; **Taxon:** kingdom: Animalia; phylum: Arthropoda; class: Insecta; order: Diptera; family: Canacidae; genus: Canaceoides; specificEpithet: Canaceoidesangulatus; scientificNameAuthorship: Wirth, 1969; **Location:** islandGroup: Hawaiian Islands; island: Oahu; verbatimLocality: Waianae; **Event:** verbatimEventDate: 1958; **Record Level:** institutionCode: UHM
- **Type status:** Other material. **Occurrence:** catalogNumber: 2006004702; recordedBy: Y Kondo; lifeStage: adult; **Taxon:** kingdom: Animalia; phylum: Arthropoda; class: Insecta; order: Diptera; family: Canacidae; genus: Canaceoides; specificEpithet: Canaceoidesangulatus; scientificNameAuthorship: Wirth, 1969; **Location:** islandGroup: Hawaiian Islands; island: Hawaii; verbatimLocality: Kona, Kahaluu; **Event:** verbatimEventDate: August 1958; **Record Level:** institutionCode: BPBM
- **Type status:** Other material. **Occurrence:** catalogNumber: 2006004649; recordedBy: JW Beardsley; lifeStage: adult; **Taxon:** kingdom: Animalia; phylum: Arthropoda; class: Insecta; order: Diptera; family: Canacidae; genus: Canaceoides; specificEpithet: Canaceoidesangulatus; scientificNameAuthorship: Wirth, 1969; **Location:** islandGroup: Hawaiian Islands; island: Lisianski; **Event:** verbatimEventDate: 18.ix.1964; **Record Level:** institutionCode: BPBM
- **Type status:** Other material. **Occurrence:** recordedBy: JA Tenorio; individualCount: 13; lifeStage: adult; **Taxon:** kingdom: Animalia; phylum: Arthropoda; class: Insecta; order: Diptera; family: Canacidae; genus: Canaceoides; specificEpithet: Canaceoidesangulatus; scientificNameAuthorship: Wirth, 1969; **Location:** islandGroup: Hawaiian Islands; island: Oahu; verbatimLocality: Waianae Beach Park; **Event:** verbatimEventDate: 11.vi.1967; **Record Level:** institutionCode: UHM
- **Type status:** Other material. **Occurrence:** recordedBy: JA Tenorio; individualCount: 3; lifeStage: adult; **Taxon:** kingdom: Animalia; phylum: Arthropoda; class: Insecta; order: Diptera; family: Canacidae; genus: Canaceoides; specificEpithet: Canaceoidesangulatus; scientificNameAuthorship: Wirth, 1969; **Location:** islandGroup: Hawaiian Islands; island: Kauai; verbatimLocality: Nawiliwili Dock; **Event:** verbatimEventDate: 14.vii.1968; **Record Level:** institutionCode: UHM
- **Type status:** Other material. **Occurrence:** recordedBy: OM Barber; individualCount: 48; lifeStage: adult; **Taxon:** kingdom: Animalia; phylum: Arthropoda; class: Insecta; order: Diptera; family: Canacidae; genus: Canaceoides; specificEpithet: Canaceoidesangulatus; scientificNameAuthorship: Wirth, 1969; **Location:** islandGroup: Hawaiian Islands; island: Kauai; verbatimLocality: Kapaa Beach Park, Kapaa; **Event:** verbatimEventDate: 13.viii.1968; **Record Level:** institutionCode: UHM
- **Type status:** Other material. **Occurrence:** recordedBy: JA Tenorio; individualCount: 7; lifeStage: adult; **Taxon:** kingdom: Animalia; phylum: Arthropoda; class: Insecta; order: Diptera; family: Canacidae; genus: Canaceoides; specificEpithet: Canaceoidesangulatus; scientificNameAuthorship: Wirth, 1969; **Location:** islandGroup: Hawaiian Islands; island: Hawaii; verbatimLocality: Naalehu Beach Park; **Event:** verbatimEventDate: 15.viii.1968; **Record Level:** institutionCode: UHM
- **Type status:** Other material. **Occurrence:** recordedBy: JA Tenorio, JM Tenorio; individualCount: 10; lifeStage: adult; **Taxon:** kingdom: Animalia; phylum: Arthropoda; class: Insecta; order: Diptera; family: Canacidae; genus: Canaceoides; specificEpithet: Canaceoidesangulatus; scientificNameAuthorship: Wirth, 1969; **Location:** islandGroup: Hawaiian Islands; island: Hawaii; verbatimLocality: Kalapana; **Event:** verbatimEventDate: 29.xi.1968; **Record Level:** institutionCode: UHM
- **Type status:** Other material. **Occurrence:** catalogNumber: 2006004658; recordedBy: JL Gressitt; lifeStage: adult; **Taxon:** kingdom: Animalia; phylum: Arthropoda; class: Insecta; order: Diptera; family: Canacidae; genus: Canaceoides; specificEpithet: Canaceoidesangulatus; scientificNameAuthorship: Wirth, 1969; **Location:** islandGroup: Hawaiian Islands; island: Kauai; verbatimLocality: Barking Sands; **Event:** verbatimEventDate: 02.iv.1969; **Record Level:** institutionCode: BPBM
- **Type status:** Other material. **Occurrence:** recordedBy: L Teremoto, L Uyenishi; individualCount: 26; lifeStage: adult; **Taxon:** kingdom: Animalia; phylum: Arthropoda; class: Insecta; order: Diptera; family: Canacidae; genus: Canaceoides; specificEpithet: Canaceoidesangulatus; scientificNameAuthorship: Wirth, 1969; **Location:** islandGroup: Hawaiian Islands; island: Kauai; verbatimLocality: Wailua Beach; **Event:** verbatimEventDate: 4.iv.1970; **Record Level:** institutionCode: UHM
- **Type status:** Other material. **Occurrence:** catalogNumber: 2006004912; recordedBy: D Devaney; lifeStage: adult; **Taxon:** kingdom: Animalia; phylum: Arthropoda; class: Insecta; order: Diptera; family: Canacidae; genus: Canaceoides; specificEpithet: Canaceoidesangulatus; scientificNameAuthorship: Wirth, 1969; **Location:** islandGroup: Hawaiian Islands; island: Maui; verbatimLocality: Kahului, Power Plant Discharge #1; **Identification:** identifiedBy: JA Tenorio; **Event:** verbatimEventDate: 08.viii.1973; **Record Level:** institutionCode: BPBM
- **Type status:** Other material. **Occurrence:** catalogNumber: 2006008014; recordedBy: GM Nishida; lifeStage: adult; **Taxon:** kingdom: Animalia; phylum: Arthropoda; class: Insecta; order: Diptera; family: Canacidae; genus: Canaceoides; specificEpithet: Canaceoidesangulatus; scientificNameAuthorship: Wirth, 1969; **Location:** islandGroup: Hawaiian Islands; island: Kahoolawe; verbatimLocality: Hakioawa Point, to 2km SW; minimumElevationInMeters: 0; maximumElevationInMeters: 66; **Identification:** identifiedBy: NL Evenhuis; dateIdentified: 1984; **Event:** verbatimEventDate: 07.xi.1979; **Record Level:** institutionCode: BPBM
- **Type status:** Other material. **Occurrence:** catalogNumber: 2008008253; recordedBy: J Strazanac; lifeStage: adult; **Taxon:** kingdom: Animalia; phylum: Arthropoda; class: Insecta; order: Diptera; family: Canacidae; genus: Canaceoides; specificEpithet: Canaceoidesangulatus; scientificNameAuthorship: Wirth, 1969; **Location:** islandGroup: Hawaiian Islands; island: Nihoa; verbatimLocality: Miller Valley; minimumElevationInMeters: 10; **Event:** verbatimEventDate: 24.vi.1990; **Record Level:** institutionCode: BPBM
- **Type status:** Other material. **Occurrence:** catalogNumber: 2008000617; recordedBy: GA Samuelson; lifeStage: adult; **Taxon:** kingdom: Animalia; phylum: Arthropoda; class: Insecta; order: Diptera; family: Canacidae; genus: Canaceoides; specificEpithet: Canaceoidesangulatus; scientificNameAuthorship: Wirth, 1969; **Location:** islandGroup: Hawaiian Islands; island: Oahu; verbatimLocality: Blaisdell Park, Waimalu Stream; **Identification:** identifiedBy: K Arakaki; **Event:** verbatimEventDate: 28.v.1998; eventRemarks: Pearl Harbor Survey; **Record Level:** institutionCode: BPBM
- **Type status:** Other material. **Occurrence:** catalogNumber: 2008000594; recordedBy: K Arakaki, GA Samuelson, K Kami; lifeStage: adult; **Taxon:** kingdom: Animalia; phylum: Arthropoda; class: Insecta; order: Diptera; family: Canacidae; genus: Canaceoides; specificEpithet: Canaceoidesangulatus; scientificNameAuthorship: Wirth, 1969; **Location:** islandGroup: Hawaiian Islands; island: Oahu; verbatimLocality: Iroquois Point, Okiokiolepe Pond, sheltered cove; **Identification:** identifiedBy: K Arakaki; **Event:** verbatimEventDate: 23.vi.1998; eventRemarks: Pearl Harbor Survey; **Record Level:** institutionCode: BPBM
- **Type status:** Other material. **Occurrence:** catalogNumber: 2008000595; recordedBy: K Arakaki, GA Samuelson, K Kami; lifeStage: adult; **Taxon:** kingdom: Animalia; phylum: Arthropoda; class: Insecta; order: Diptera; family: Canacidae; genus: Canaceoides; specificEpithet: Canaceoidesangulatus; scientificNameAuthorship: Wirth, 1969; **Location:** islandGroup: Hawaiian Islands; island: Oahu; verbatimLocality: Iroquois Point, at Pearl Harbor entrance; **Identification:** identifiedBy: K Arakaki; **Event:** verbatimEventDate: 23.vi.1998; eventRemarks: Pearl Harbor Survey; **Record Level:** institutionCode: BPBM
- **Type status:** Other material. **Occurrence:** catalogNumber: 2008000596; recordedBy: K Arakaki, GA Samuelson, K Kami; lifeStage: adult; **Taxon:** kingdom: Animalia; phylum: Arthropoda; class: Insecta; order: Diptera; family: Canacidae; genus: Canaceoides; specificEpithet: Canaceoidesangulatus; scientificNameAuthorship: Wirth, 1969; **Location:** islandGroup: Hawaiian Islands; island: Oahu; verbatimLocality: Iroquois Point, at Pearl Harbor entrance; **Identification:** identifiedBy: K Arakaki; **Event:** verbatimEventDate: 23.vi.1998; eventRemarks: Pearl Harbor Survey; **Record Level:** institutionCode: BPBM
- **Type status:** Other material. **Occurrence:** catalogNumber: 2008000597; recordedBy: K Arakaki, GA Samuelson, K Kami; lifeStage: adult; **Taxon:** kingdom: Animalia; phylum: Arthropoda; class: Insecta; order: Diptera; family: Canacidae; genus: Canaceoides; specificEpithet: Canaceoidesangulatus; scientificNameAuthorship: Wirth, 1969; **Location:** islandGroup: Hawaiian Islands; island: Oahu; verbatimLocality: Iroquois Point, at Pearl Harbor entrance; **Identification:** identifiedBy: K Arakaki; **Event:** verbatimEventDate: 23.vi.1998; eventRemarks: Pearl Harbor Survey; **Record Level:** institutionCode: BPBM
- **Type status:** Other material. **Occurrence:** catalogNumber: 2008000598; recordedBy: K Arakaki, GA Samuelson, K Kami; lifeStage: adult; **Taxon:** kingdom: Animalia; phylum: Arthropoda; class: Insecta; order: Diptera; family: Canacidae; genus: Canaceoides; specificEpithet: Canaceoidesangulatus; scientificNameAuthorship: Wirth, 1969; **Location:** islandGroup: Hawaiian Islands; island: Oahu; verbatimLocality: Iroquois Point, edge of fish pond; **Identification:** identifiedBy: K Arakaki; **Event:** verbatimEventDate: 23.vi.1998; eventRemarks: Pearl Harbor Survey; **Record Level:** institutionCode: BPBM
- **Type status:** Other material. **Occurrence:** catalogNumber: 2008000599; recordedBy: K Arakaki, GA Samuelson, K Kami; lifeStage: adult; **Taxon:** kingdom: Animalia; phylum: Arthropoda; class: Insecta; order: Diptera; family: Canacidae; genus: Canaceoides; specificEpithet: Canaceoidesangulatus; scientificNameAuthorship: Wirth, 1969; **Location:** islandGroup: Hawaiian Islands; island: Oahu; verbatimLocality: Iroquois Point, at Pearl Harbor entrance; **Identification:** identifiedBy: K Arakaki; **Event:** verbatimEventDate: 23.vi.1998; eventRemarks: Pearl Harbor Survey; **Record Level:** institutionCode: BPBM
- **Type status:** Other material. **Occurrence:** catalogNumber: 2008000600; recordedBy: K Arakaki, GA Samuelson, K Kami; lifeStage: adult; **Taxon:** kingdom: Animalia; phylum: Arthropoda; class: Insecta; order: Diptera; family: Canacidae; genus: Canaceoides; specificEpithet: Canaceoidesangulatus; scientificNameAuthorship: Wirth, 1969; **Location:** islandGroup: Hawaiian Islands; island: Oahu; verbatimLocality: Iroquois Point, edge of fish pond; **Identification:** identifiedBy: K Arakaki; **Event:** verbatimEventDate: 23.vi.1998; eventRemarks: Pearl Harbor Survey; **Record Level:** institutionCode: BPBM
- **Type status:** Other material. **Occurrence:** catalogNumber: 2008000601; recordedBy: K Arakaki, GA Samuelson, K Kami; lifeStage: adult; **Taxon:** kingdom: Animalia; phylum: Arthropoda; class: Insecta; order: Diptera; family: Canacidae; genus: Canaceoides; specificEpithet: Canaceoidesangulatus; scientificNameAuthorship: Wirth, 1969; **Location:** islandGroup: Hawaiian Islands; island: Oahu; verbatimLocality: Iroquois Point, at Pearl Harbor entrance; **Identification:** identifiedBy: K Arakaki; **Event:** verbatimEventDate: 23.vi.1998; eventRemarks: Pearl Harbor Survey; **Record Level:** institutionCode: BPBM
- **Type status:** Other material. **Occurrence:** catalogNumber: 2008000602; recordedBy: K Arakaki, GA Samuelson, K Kami; lifeStage: adult; **Taxon:** kingdom: Animalia; phylum: Arthropoda; class: Insecta; order: Diptera; family: Canacidae; genus: Canaceoides; specificEpithet: Canaceoidesangulatus; scientificNameAuthorship: Wirth, 1969; **Location:** islandGroup: Hawaiian Islands; island: Oahu; verbatimLocality: Iroquois Point, at Pearl Harbor entrance; **Identification:** identifiedBy: K Arakaki; **Event:** verbatimEventDate: 23.vi.1998; eventRemarks: Pearl Harbor Survey; **Record Level:** institutionCode: BPBM
- **Type status:** Other material. **Occurrence:** catalogNumber: 2008000603; recordedBy: K Arakaki, GA Samuelson, K Kami; lifeStage: adult; **Taxon:** kingdom: Animalia; phylum: Arthropoda; class: Insecta; order: Diptera; family: Canacidae; genus: Canaceoides; specificEpithet: Canaceoidesangulatus; scientificNameAuthorship: Wirth, 1969; **Location:** islandGroup: Hawaiian Islands; island: Oahu; verbatimLocality: Iroquois Point, at Pearl Harbor entrance; **Identification:** identifiedBy: K Arakaki; **Event:** verbatimEventDate: 23.vi.1998; eventRemarks: Pearl Harbor Survey; **Record Level:** institutionCode: BPBM
- **Type status:** Other material. **Occurrence:** catalogNumber: 2008000619; recordedBy: K Arakaki, K Kami; lifeStage: adult; **Taxon:** kingdom: Animalia; phylum: Arthropoda; class: Insecta; order: Diptera; family: Canacidae; genus: Canaceoides; specificEpithet: Canaceoidesangulatus; scientificNameAuthorship: Wirth, 1969; **Location:** islandGroup: Hawaiian Islands; island: Oahu; verbatimLocality: Aiea Bay, rocky shoreline; **Identification:** identifiedBy: K Arakaki; **Event:** verbatimEventDate: 24.vi.1998; eventRemarks: Pearl Harbor Survey; **Record Level:** institutionCode: BPBM
- **Type status:** Other material. **Occurrence:** catalogNumber: 2008000620; recordedBy: K Arakaki, K Kami; lifeStage: adult; **Taxon:** kingdom: Animalia; phylum: Arthropoda; class: Insecta; order: Diptera; family: Canacidae; genus: Canaceoides; specificEpithet: Canaceoidesangulatus; scientificNameAuthorship: Wirth, 1969; **Location:** islandGroup: Hawaiian Islands; island: Oahu; verbatimLocality: Aiea Bay, rocky shoreline; **Identification:** identifiedBy: K Arakaki; **Event:** verbatimEventDate: 24.vi.1998; eventRemarks: Pearl Harbor Survey; **Record Level:** institutionCode: BPBM
- **Type status:** Other material. **Occurrence:** catalogNumber: 2008000621; recordedBy: K Arakaki, K Kami; lifeStage: adult; **Taxon:** kingdom: Animalia; phylum: Arthropoda; class: Insecta; order: Diptera; family: Canacidae; genus: Canaceoides; specificEpithet: Canaceoidesangulatus; scientificNameAuthorship: Wirth, 1969; **Location:** islandGroup: Hawaiian Islands; island: Oahu; verbatimLocality: Aiea Bay, stream mouth; **Identification:** identifiedBy: K Arakaki; **Event:** verbatimEventDate: 24.vi.1998; eventRemarks: Pearl Harbor Survey; **Record Level:** institutionCode: BPBM
- **Type status:** Other material. **Occurrence:** catalogNumber: 2008000606; recordedBy: K Arakaki, K Kami; lifeStage: adult; **Taxon:** kingdom: Animalia; phylum: Arthropoda; class: Insecta; order: Diptera; family: Canacidae; genus: Canaceoides; specificEpithet: Canaceoidesangulatus; scientificNameAuthorship: Wirth, 1969; **Location:** islandGroup: Hawaiian Islands; island: Oahu; verbatimLocality: Waiau Hawaiian Electric Power Plant, open rocky shoreline at ewa end; minimumElevationInMeters: 0; **Identification:** identifiedBy: K Arakaki; **Event:** verbatimEventDate: 30.vi.1998; eventRemarks: Pearl Harbor Survey; **Record Level:** institutionCode: BPBM
- **Type status:** Other material. **Occurrence:** catalogNumber: 2008000607; recordedBy: K Arakaki, K Kami; lifeStage: adult; **Taxon:** kingdom: Animalia; phylum: Arthropoda; class: Insecta; order: Diptera; family: Canacidae; genus: Canaceoides; specificEpithet: Canaceoidesangulatus; scientificNameAuthorship: Wirth, 1969; **Location:** islandGroup: Hawaiian Islands; island: Oahu; verbatimLocality: Waiau Hawaiian Electric Power Plant, open rocky shoreline at ewa end; minimumElevationInMeters: 0; **Identification:** identifiedBy: K Arakaki; **Event:** verbatimEventDate: 30.vi.1998; eventRemarks: Pearl Harbor Survey; **Record Level:** institutionCode: BPBM
- **Type status:** Other material. **Occurrence:** catalogNumber: 2008000608; recordedBy: K Arakaki, K Kami; lifeStage: adult; **Taxon:** kingdom: Animalia; phylum: Arthropoda; class: Insecta; order: Diptera; family: Canacidae; genus: Canaceoides; specificEpithet: Canaceoidesangulatus; scientificNameAuthorship: Wirth, 1969; **Location:** islandGroup: Hawaiian Islands; island: Oahu; verbatimLocality: Waiau Hawaiian Electric Power Plant, open rocky shoreline at ewa end; minimumElevationInMeters: 0; **Identification:** identifiedBy: K Arakaki; **Event:** verbatimEventDate: 30.vi.1998; eventRemarks: Pearl Harbor Survey; **Record Level:** institutionCode: BPBM
- **Type status:** Other material. **Occurrence:** catalogNumber: 2008000609; recordedBy: K Arakaki, K Kami; lifeStage: adult; **Taxon:** kingdom: Animalia; phylum: Arthropoda; class: Insecta; order: Diptera; family: Canacidae; genus: Canaceoides; specificEpithet: Canaceoidesangulatus; scientificNameAuthorship: Wirth, 1969; **Location:** islandGroup: Hawaiian Islands; island: Oahu; verbatimLocality: Waiau Hawaiian Electric Power Plant, open rocky shoreline at ewa end; minimumElevationInMeters: 0; **Identification:** identifiedBy: K Arakaki; **Event:** verbatimEventDate: 30.vi.1998; eventRemarks: Pearl Harbor Survey; **Record Level:** institutionCode: BPBM
- **Type status:** Other material. **Occurrence:** catalogNumber: 2008000610; recordedBy: K Arakaki, K Kami; sex: female; lifeStage: adult; **Taxon:** kingdom: Animalia; phylum: Arthropoda; class: Insecta; order: Diptera; family: Canacidae; genus: Canaceoides; specificEpithet: Canaceoidesangulatus; scientificNameAuthorship: Wirth, 1969; **Location:** islandGroup: Hawaiian Islands; island: Oahu; verbatimLocality: Waiau Hawaiian Electric Power Plant, open rocky shoreline at ewa end; minimumElevationInMeters: 0; **Identification:** identifiedBy: K Arakaki; **Event:** verbatimEventDate: 30.vi.1998; eventRemarks: Pearl Harbor Survey; **Record Level:** institutionCode: BPBM
- **Type status:** Other material. **Occurrence:** catalogNumber: 2008000611; recordedBy: K Arakaki, K Kami; lifeStage: adult; **Taxon:** kingdom: Animalia; phylum: Arthropoda; class: Insecta; order: Diptera; family: Canacidae; genus: Canaceoides; specificEpithet: Canaceoidesangulatus; scientificNameAuthorship: Wirth, 1969; **Location:** islandGroup: Hawaiian Islands; island: Oahu; verbatimLocality: Waiau Hawaiian Electric Power Plant, open rocky shoreline at ewa end; minimumElevationInMeters: 0; **Identification:** identifiedBy: K Arakaki; **Event:** verbatimEventDate: 30.vi.1998; eventRemarks: Pearl Harbor Survey; **Record Level:** institutionCode: BPBM
- **Type status:** Other material. **Occurrence:** catalogNumber: 2008000612; recordedBy: K Arakaki, K Kami; lifeStage: adult; **Taxon:** kingdom: Animalia; phylum: Arthropoda; class: Insecta; order: Diptera; family: Canacidae; genus: Canaceoides; specificEpithet: Canaceoidesangulatus; scientificNameAuthorship: Wirth, 1969; **Location:** islandGroup: Hawaiian Islands; island: Oahu; verbatimLocality: Waiau Hawaiian Electric Power Plant, open rocky shoreline at ewa end; minimumElevationInMeters: 0; **Identification:** identifiedBy: K Arakaki; **Event:** verbatimEventDate: 30.vi.1998; eventRemarks: Pearl Harbor Survey; **Record Level:** institutionCode: BPBM
- **Type status:** Other material. **Occurrence:** catalogNumber: 2008000613; recordedBy: K Arakaki, K Kami; lifeStage: adult; **Taxon:** kingdom: Animalia; phylum: Arthropoda; class: Insecta; order: Diptera; family: Canacidae; genus: Canaceoides; specificEpithet: Canaceoidesangulatus; scientificNameAuthorship: Wirth, 1969; **Location:** islandGroup: Hawaiian Islands; island: Oahu; verbatimLocality: Waiau Hawaiian Electric Power Plant, open rocky shoreline at ewa end; minimumElevationInMeters: 0; **Identification:** identifiedBy: K Arakaki; **Event:** verbatimEventDate: 30.vi.1998; eventRemarks: Pearl Harbor Survey; **Record Level:** institutionCode: BPBM
- **Type status:** Other material. **Occurrence:** catalogNumber: 2008000614; recordedBy: K Arakaki, K Kami; lifeStage: adult; **Taxon:** kingdom: Animalia; phylum: Arthropoda; class: Insecta; order: Diptera; family: Canacidae; genus: Canaceoides; specificEpithet: Canaceoidesangulatus; scientificNameAuthorship: Wirth, 1969; **Location:** islandGroup: Hawaiian Islands; island: Oahu; verbatimLocality: Waiau Hawaiian Electric Power Plant, open rocky shoreline at ewa end; minimumElevationInMeters: 0; **Identification:** identifiedBy: K Arakaki; **Event:** verbatimEventDate: 30.vi.1998; eventRemarks: Pearl Harbor Survey; **Record Level:** institutionCode: BPBM
- **Type status:** Other material. **Occurrence:** catalogNumber: 2008000615; recordedBy: K Arakaki, K Kami; lifeStage: adult; **Taxon:** kingdom: Animalia; phylum: Arthropoda; class: Insecta; order: Diptera; family: Canacidae; genus: Canaceoides; specificEpithet: Canaceoidesangulatus; scientificNameAuthorship: Wirth, 1969; **Location:** islandGroup: Hawaiian Islands; island: Oahu; verbatimLocality: Waiau Hawaiian Electric Power Plant, open rocky shoreline at ewa end; minimumElevationInMeters: 0; **Identification:** identifiedBy: K Arakaki; **Event:** verbatimEventDate: 30.vi.1998; eventRemarks: Pearl Harbor Survey; **Record Level:** institutionCode: BPBM
- **Type status:** Other material. **Occurrence:** catalogNumber: 2008000616; recordedBy: K Arakaki, K Kami; lifeStage: adult; **Taxon:** kingdom: Animalia; phylum: Arthropoda; class: Insecta; order: Diptera; family: Canacidae; genus: Canaceoides; specificEpithet: Canaceoidesangulatus; scientificNameAuthorship: Wirth, 1969; **Location:** islandGroup: Hawaiian Islands; island: Oahu; verbatimLocality: Waiau Hawaiian Electric Power Plant, open rocky shoreline at ewa end; minimumElevationInMeters: 0; **Identification:** identifiedBy: K Arakaki; **Event:** verbatimEventDate: 30.vi.1998; eventRemarks: Pearl Harbor Survey; **Record Level:** institutionCode: BPBM
- **Type status:** Other material. **Occurrence:** catalogNumber: 2008000618; recordedBy: K Arakaki, K Kami; lifeStage: adult; **Taxon:** kingdom: Animalia; phylum: Arthropoda; class: Insecta; order: Diptera; family: Canacidae; genus: Canaceoides; specificEpithet: Canaceoidesangulatus; scientificNameAuthorship: Wirth, 1969; **Location:** islandGroup: Hawaiian Islands; island: Oahu; verbatimLocality: Waiau Hawaiian Electric Power Plant, open rocky shoreline at ewa end; minimumElevationInMeters: 0; **Identification:** identifiedBy: K Arakaki; **Event:** verbatimEventDate: 30.vi.1998; eventRemarks: Pearl Harbor Survey; **Record Level:** institutionCode: BPBM
- **Type status:** Other material. **Occurrence:** catalogNumber: 2008000622; recordedBy: K Arakaki, K Kami; lifeStage: adult; **Taxon:** kingdom: Animalia; phylum: Arthropoda; class: Insecta; order: Diptera; family: Canacidae; genus: Canaceoides; specificEpithet: Canaceoidesangulatus; scientificNameAuthorship: Wirth, 1969; **Location:** islandGroup: Hawaiian Islands; island: Oahu; verbatimLocality: E'o Canal, Ted Makalena Golf Course, at shore line; **Identification:** identifiedBy: K Arakaki; **Event:** verbatimEventDate: 27.vii.1998; eventRemarks: Pearl Harbor Survey; **Record Level:** institutionCode: BPBM
- **Type status:** Other material. **Occurrence:** catalogNumber: 2008000623; recordedBy: K Arakaki, K Kami; lifeStage: adult; **Taxon:** kingdom: Animalia; phylum: Arthropoda; class: Insecta; order: Diptera; family: Canacidae; genus: Canaceoides; specificEpithet: Canaceoidesangulatus; scientificNameAuthorship: Wirth, 1969; **Location:** islandGroup: Hawaiian Islands; island: Oahu; verbatimLocality: E'o Canal, Ted Makalena Golf Course, at shore line; **Identification:** identifiedBy: K Arakaki; **Event:** verbatimEventDate: 27.vii.1998; eventRemarks: Pearl Harbor Survey; **Record Level:** institutionCode: BPBM
- **Type status:** Other material. **Occurrence:** catalogNumber: 2008000624; recordedBy: K Arakaki, K Kami; lifeStage: adult; **Taxon:** kingdom: Animalia; phylum: Arthropoda; class: Insecta; order: Diptera; family: Canacidae; genus: Canaceoides; specificEpithet: Canaceoidesangulatus; scientificNameAuthorship: Wirth, 1969; **Location:** islandGroup: Hawaiian Islands; island: Oahu; verbatimLocality: E'o Canal, Ted Makalena Golf Course, at shore line; **Identification:** identifiedBy: K Arakaki; **Event:** verbatimEventDate: 27.vii.1998; eventRemarks: Pearl Harbor Survey; **Record Level:** institutionCode: BPBM
- **Type status:** Other material. **Occurrence:** catalogNumber: 2008000625; recordedBy: K Arakaki, K Kami; lifeStage: adult; **Taxon:** kingdom: Animalia; phylum: Arthropoda; class: Insecta; order: Diptera; family: Canacidae; genus: Canaceoides; specificEpithet: Canaceoidesangulatus; scientificNameAuthorship: Wirth, 1969; **Location:** islandGroup: Hawaiian Islands; island: Oahu; verbatimLocality: E'o Canal, Ted Makalena Golf Course, at shore line; **Identification:** identifiedBy: K Arakaki; **Event:** verbatimEventDate: 27.vii.1998; eventRemarks: Pearl Harbor Survey; **Record Level:** institutionCode: BPBM
- **Type status:** Other material. **Occurrence:** catalogNumber: 2006016244; recordedBy: PT Oboyski; lifeStage: adult; **Taxon:** kingdom: Animalia; phylum: Arthropoda; class: Insecta; order: Diptera; family: Canacidae; genus: Canaceoides; specificEpithet: Canaceoidesangulatus; scientificNameAuthorship: Wirth, 1969; **Location:** islandGroup: Hawaiian Islands; island: Nihoa; **Identification:** identifiedBy: PT Oboyski; dateIdentified: 2005; **Event:** verbatimEventDate: 13 Aug 2005; **Record Level:** institutionCode: BPBM
- **Type status:** Other material. **Occurrence:** catalogNumber: 2006016243; recordedBy: PT Oboyski; lifeStage: adult; **Taxon:** kingdom: Animalia; phylum: Arthropoda; class: Insecta; order: Diptera; family: Canacidae; genus: Canaceoides; specificEpithet: Canaceoidesangulatus; scientificNameAuthorship: Wirth, 1969; **Location:** islandGroup: Hawaiian Islands; island: Nihoa; **Identification:** identifiedBy: PT Oboyski; dateIdentified: 2005; **Event:** verbatimEventDate: 13 Aug 2005; **Record Level:** institutionCode: BPBM
- **Type status:** Other material. **Occurrence:** recordedBy: RT Lapoint; lifeStage: adult; **Taxon:** kingdom: Animalia; phylum: Arthropoda; class: Insecta; order: Diptera; family: Canacidae; genus: Canaceoides; specificEpithet: Canaceoidesangulatus; scientificNameAuthorship: Wirth, 1969; **Location:** islandGroup: Hawaiian Islands; island: Hawaii; verbatimLocality: Kolekole Beach Park; **Identification:** identifiedBy: PM O'Grady; dateIdentified: 2014; **Event:** verbatimEventDate: 31.vii.2009; **Record Level:** institutionCode: EMEC; collectionCode: 205642
- **Type status:** Other material. **Occurrence:** recordedBy: PM O'Grady; lifeStage: adult; **Taxon:** kingdom: Animalia; phylum: Arthropoda; class: Insecta; order: Diptera; family: Canacidae; genus: Canaceoides; specificEpithet: Canaceoidesangulatus; scientificNameAuthorship: Wirth, 1969; **Location:** islandGroup: Hawaiian Islands; island: Kauai; verbatimLocality: Kikiaola, Small Boat Harbor; **Identification:** identifiedBy: PM O'Grady; dateIdentified: 2014; **Event:** verbatimEventDate: 5.i.2010; **Record Level:** institutionCode: EMEC; collectionCode: 205640
- **Type status:** Other material. **Occurrence:** recordedBy: NL Evenhuis; lifeStage: adult; **Taxon:** kingdom: Animalia; phylum: Arthropoda; class: Insecta; order: Diptera; family: Canacidae; genus: Canaceoides; specificEpithet: Canaceoidesangulatus; scientificNameAuthorship: Wirth, 1969; **Location:** islandGroup: Hawaiian Islands; island: Hawaii; verbatimLocality: Punaluu Beach Park; **Identification:** identifiedBy: PM O'Grady; dateIdentified: 2014; **Event:** verbatimEventDate: 26.vi.2010; **Record Level:** institutionCode: EMEC; collectionCode: 205519
- **Type status:** Other material. **Occurrence:** recordedBy: NL Evenhuis; lifeStage: adult; **Taxon:** kingdom: Animalia; phylum: Arthropoda; class: Insecta; order: Diptera; family: Canacidae; genus: Canaceoides; specificEpithet: Canaceoidesangulatus; scientificNameAuthorship: Wirth, 1969; **Location:** islandGroup: Hawaiian Islands; island: Hawaii; verbatimLocality: Ninole, Kauwale Fish Pond; **Identification:** identifiedBy: PM O'Grady; dateIdentified: 2014; **Event:** verbatimEventDate: 26.vi.2010; **Record Level:** institutionCode: EMEC; collectionCode: 205646
- **Type status:** Other material. **Occurrence:** recordedBy: NL Evenhuis, G Bennett, KR Goodman, BS Ort, S Wang; lifeStage: adult; **Taxon:** kingdom: Animalia; phylum: Arthropoda; class: Insecta; order: Diptera; family: Canacidae; genus: Canaceoides; specificEpithet: Canaceoidesangulatus; scientificNameAuthorship: Wirth, 1969; **Location:** islandGroup: Hawaiian Islands; island: Molokai; verbatimLocality: Halawa Bay Park; **Identification:** identifiedBy: PM O'Grady; dateIdentified: 2014; **Event:** verbatimEventDate: 12.i.2011; **Record Level:** institutionCode: EMEC
- **Type status:** Other material. **Occurrence:** recordedBy: NL Evenhuis; lifeStage: adult; **Taxon:** kingdom: Animalia; phylum: Arthropoda; class: Insecta; order: Diptera; family: Canacidae; genus: Canaceoides; specificEpithet: Canaceoidesangulatus; scientificNameAuthorship: Wirth, 1969; **Location:** islandGroup: Hawaiian Islands; island: Molokai; verbatimLocality: Waialua beach rocks; **Identification:** identifiedBy: PM O'Grady; dateIdentified: 2014; **Event:** verbatimEventDate: 12.i.2011; **Record Level:** institutionCode: EMEC; collectionCode: 205534
- **Type status:** Other material. **Occurrence:** recordedBy: KR Goodman; lifeStage: adult; **Taxon:** kingdom: Animalia; phylum: Arthropoda; class: Insecta; order: Diptera; family: Canacidae; genus: Canaceoides; specificEpithet: Canaceoidesangulatus; scientificNameAuthorship: Wirth, 1969; **Location:** islandGroup: Hawaiian Islands; **Identification:** identifiedBy: PM O'Grady; dateIdentified: 2014; **Event:** verbatimEventDate: 12.i.2011; **Record Level:** institutionCode: EMEC; collectionCode: 205638
- **Type status:** Other material. **Occurrence:** recordedBy: KR Goodman; lifeStage: adult; **Taxon:** kingdom: Animalia; phylum: Arthropoda; class: Insecta; order: Diptera; family: Canacidae; genus: Canaceoides; specificEpithet: Canaceoidesangulatus; scientificNameAuthorship: Wirth, 1969; **Location:** islandGroup: Hawaiian Islands; island: Molokai; verbatimLocality: Hale O Ono; **Identification:** identifiedBy: PM O'Grady; dateIdentified: 2014; **Event:** verbatimEventDate: 12.i.2011; **Record Level:** institutionCode: EMEC; collectionCode: 205644
- **Type status:** Other material. **Occurrence:** recordedBy: KR Goodman; lifeStage: adult; **Taxon:** kingdom: Animalia; phylum: Arthropoda; class: Insecta; order: Diptera; family: Canacidae; genus: Canaceoides; specificEpithet: Canaceoidesangulatus; scientificNameAuthorship: Wirth, 1969; **Location:** islandGroup: Hawaiian Islands; island: Molokai; verbatimLocality: Beach rocks near mile 21.25; **Identification:** identifiedBy: PM O'Grady; dateIdentified: 2014; **Event:** verbatimEventDate: 12.i.2011; **Record Level:** institutionCode: EMEC; collectionCode: 205645
- **Type status:** Other material. **Occurrence:** recordedBy: no collector given; lifeStage: adult; **Taxon:** kingdom: Animalia; phylum: Arthropoda; class: Insecta; order: Diptera; family: Canacidae; genus: Canaceoides; specificEpithet: Canaceoidesangulatus; scientificNameAuthorship: Wirth, 1969; **Location:** islandGroup: Hawaiian Islands; island: Molokai; verbatimLocality: Moomoni; **Identification:** identifiedBy: PM O'Grady; dateIdentified: 2014; **Event:** verbatimEventDate: 13.i.2011; **Record Level:** institutionCode: EMEC; collectionCode: 205637
- **Type status:** Other material. **Occurrence:** recordedBy: KR Goodman; lifeStage: adult; **Taxon:** kingdom: Animalia; phylum: Arthropoda; class: Insecta; order: Diptera; family: Canacidae; genus: Canaceoides; specificEpithet: Canaceoidesangulatus; scientificNameAuthorship: Wirth, 1969; **Location:** islandGroup: Hawaiian Islands; island: Molokai; verbatimLocality: Moomoni; **Identification:** identifiedBy: PM O'Grady; dateIdentified: 2014; **Event:** verbatimEventDate: 13.i.2011; **Record Level:** institutionCode: EMEC; collectionCode: 205639
- **Type status:** Other material. **Occurrence:** recordedBy: NL Evenhuis; lifeStage: adult; **Taxon:** kingdom: Animalia; phylum: Arthropoda; class: Insecta; order: Diptera; family: Canacidae; genus: Canaceoides; specificEpithet: Canaceoidesangulatus; scientificNameAuthorship: Wirth, 1969; **Location:** islandGroup: Hawaiian Islands; island: Molokai; verbatimLocality: Moomoni; **Identification:** identifiedBy: PM O'Grady; dateIdentified: 2014; **Event:** verbatimEventDate: 15.i.2011; **Record Level:** institutionCode: EMEC; collectionCode: 205520

##### Ecological interactions

###### Native status

adventive

##### Distribution

**Figure 8.**
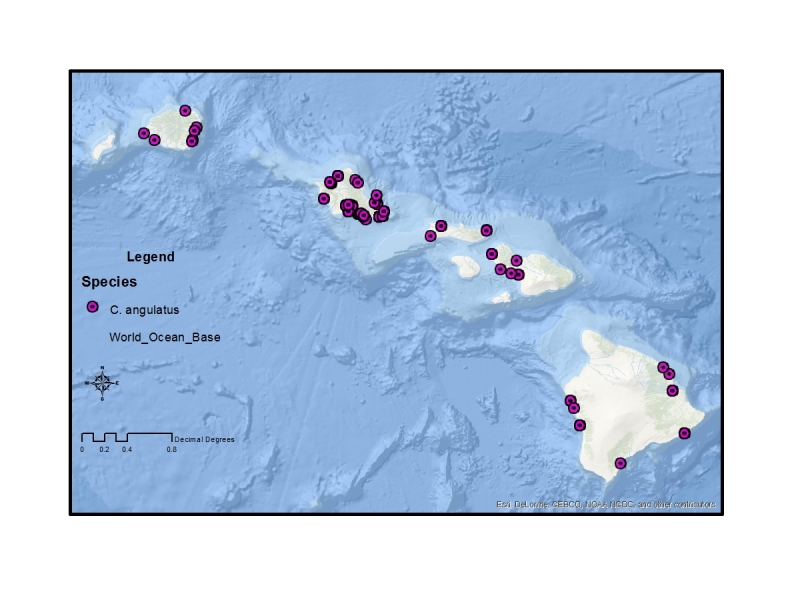
Distribution of *C. angulatus* in the main Hawaiian Islands. This species is also found in the Northwest Hawaiian Islands (Laysan, Lisianski, Midway, Nihoa).

HAWAIIAN ISLANDS: Midway, Laysan, Lisianski, Nihoa, Kauai, Oahu, Molokai, Kahoolawae, Maui, Hawaii (Fig. 8); MEXICO: Baja California Norte,Baja California Sur, Sonora; ECUADOR: Galapagos Islands; PERU

##### Notes

Cresson (1926), [description of *Canace nudata*]; Williams (1938), as *C. nudata*, [ecology; eggs, larvae, puparium]; Wirth (1951), as *C. nudata*, [family revision; male genitalia (ventral and lateral)]; Wirth (1969), [original description; male genitalia, female terminalia, ninth sternum, spermatheca, distribution map (partial)]; Hardy and Delfinado (1980), [redescription and revision of Hawaiian taxa; head (front and lateral), female terminalia (dorsal and ventral), spermathecae, wing, surstylus; cephalopharyngael skeleton (larval and pupal, puparium, third instar larvae]; Mathis (1992), [World Catalog]; Nishida (2002), [Hawaiian Arthropod Checklist]; Evenhuis and Eldredge (2004), [Natural History of Nihoa and Necker Islands].

#### Canaceoides hawaiiensis

Wirth, 1969

##### Materials

- **Type status:** Holotype. **Occurrence:** recordedBy: DE Hardy; individualCount: 1; sex: male; lifeStage: adult; **Taxon:** kingdom: Animalia; phylum: Arthropoda; class: Insecta; order: Diptera; family: Canacidae; genus: Canaceoides; specificEpithet: Canaceoideshawaiiensis; scientificNameAuthorship: Wirth, 1969; **Location:** islandGroup: Hawaiian Islands; island: Maui; verbatimLocality: Hana; **Identification:** identifiedBy: DE Hardy & MD Delfinado; dateIdentified: 1980; **Event:** verbatimEventDate: 20.vi.1967; **Record Level:** institutionCode: BPBM
- **Type status:** Paratype. **Occurrence:** recordedBy: EH Bryan; individualCount: 1; sex: male; lifeStage: adult; **Taxon:** kingdom: Animalia; phylum: Arthropoda; class: Insecta; order: Diptera; family: Canacidae; genus: Canaceoides; specificEpithet: Canaceoideshawaiiensis; scientificNameAuthorship: Wirth, 1969; **Location:** islandGroup: Hawaiian Islands; island: Nihoa; verbatimLocality: ex pools of salt water; **Identification:** identifiedBy: DE Hardy & MD Delfinado; dateIdentified: 1980; **Event:** verbatimEventDate: 12.vi.1922; **Record Level:** institutionCode: ANSP
- **Type status:** Paratype. **Occurrence:** recordedBy: EH Bryan; individualCount: 1; sex: female; lifeStage: adult; **Taxon:** kingdom: Animalia; phylum: Arthropoda; class: Insecta; order: Diptera; family: Canacidae; genus: Canaceoides; specificEpithet: Canaceoideshawaiiensis; scientificNameAuthorship: Wirth, 1969; **Location:** islandGroup: Hawaiian Islands; island: Nihoa; verbatimLocality: ex pools of salt water; **Identification:** identifiedBy: DE Hardy & MD Delfinado; dateIdentified: 1980; **Event:** verbatimEventDate: 12.vi.1922; **Record Level:** institutionCode: ANSP
- **Type status:** Paratype. **Occurrence:** recordedBy: EH Bryan; individualCount: 3; sex: male; lifeStage: adult; **Taxon:** kingdom: Animalia; phylum: Arthropoda; class: Insecta; order: Diptera; family: Canacidae; genus: Canaceoides; specificEpithet: Canaceoideshawaiiensis; scientificNameAuthorship: Wirth, 1969; **Location:** islandGroup: Hawaiian Islands; island: Oahu; verbatimLocality: Wawamalu Beach near Koko Crater; **Identification:** identifiedBy: DE Hardy & MD Delfinado; dateIdentified: 1980; **Event:** verbatimEventDate: 17.xii.1922; **Record Level:** institutionCode: ANSP
- **Type status:** Paratype. **Occurrence:** recordedBy: Y Tanada; individualCount: 2; sex: male; lifeStage: adult; **Taxon:** kingdom: Animalia; phylum: Arthropoda; class: Insecta; order: Diptera; family: Canacidae; genus: Canaceoides; specificEpithet: Canaceoideshawaiiensis; scientificNameAuthorship: Wirth, 1969; **Location:** islandGroup: Hawaiian Islands; island: Molokai; verbatimLocality: Mapulehu, Kupeke Pond; **Identification:** identifiedBy: DE Hardy & MD Delfinado; dateIdentified: 1980; **Event:** verbatimEventDate: 28.viii.1944
- **Type status:** Paratype. **Occurrence:** recordedBy: Y Tanada; individualCount: 3; sex: male; lifeStage: adult; **Taxon:** kingdom: Animalia; phylum: Arthropoda; class: Insecta; order: Diptera; family: Canacidae; genus: Canaceoides; specificEpithet: Canaceoideshawaiiensis; scientificNameAuthorship: Wirth, 1969; **Location:** islandGroup: Hawaiian Islands; island: Molokai; verbatimLocality: Niaupala; **Identification:** identifiedBy: DE Hardy & MD Delfinado; dateIdentified: 1980; **Event:** verbatimEventDate: 28.xii.1944
- **Type status:** Paratype. **Occurrence:** recordedBy: Y Tanada; individualCount: 4; sex: female; lifeStage: adult; **Taxon:** kingdom: Animalia; phylum: Arthropoda; class: Insecta; order: Diptera; family: Canacidae; genus: Canaceoides; specificEpithet: Canaceoideshawaiiensis; scientificNameAuthorship: Wirth, 1969; **Location:** islandGroup: Hawaiian Islands; island: Molokai; verbatimLocality: Niaupala; **Identification:** identifiedBy: DE Hardy & MD Delfinado; dateIdentified: 1980; **Event:** verbatimEventDate: 28.xii.1944
- **Type status:** Paratype. **Occurrence:** recordedBy: WW Wirth; individualCount: 2; sex: male; lifeStage: adult; **Taxon:** kingdom: Animalia; phylum: Arthropoda; class: Insecta; order: Diptera; family: Canacidae; genus: Canaceoides; specificEpithet: Canaceoideshawaiiensis; scientificNameAuthorship: Wirth, 1969; **Location:** islandGroup: Hawaiian Islands; island: Oahu; verbatimLocality: Hanauma Bay; **Identification:** identifiedBy: DE Hardy & MD Delfinado; dateIdentified: 1980; **Event:** verbatimEventDate: 4.i.1946
- **Type status:** Paratype. **Occurrence:** recordedBy: WW Wirth; individualCount: 1; sex: female; lifeStage: adult; **Taxon:** kingdom: Animalia; phylum: Arthropoda; class: Insecta; order: Diptera; family: Canacidae; genus: Canaceoides; specificEpithet: Canaceoideshawaiiensis; scientificNameAuthorship: Wirth, 1969; **Location:** islandGroup: Hawaiian Islands; island: Oahu; verbatimLocality: Maile; **Identification:** identifiedBy: DE Hardy & MD Delfinado; dateIdentified: 1980; **Event:** verbatimEventDate: 8.i.1946
- **Type status:** Paratype. **Occurrence:** recordedBy: WW Wirth; individualCount: 1; sex: male; lifeStage: adult; **Taxon:** kingdom: Animalia; phylum: Arthropoda; class: Insecta; order: Diptera; family: Canacidae; genus: Canaceoides; specificEpithet: Canaceoideshawaiiensis; scientificNameAuthorship: Wirth, 1969; **Location:** islandGroup: Hawaiian Islands; island: Kauai; verbatimLocality: Nawiliwili; **Identification:** identifiedBy: DE Hardy & MD Delfinado; dateIdentified: 1980; **Event:** verbatimEventDate: 9.ix.1946
- **Type status:** Paratype. **Occurrence:** recordedBy: MS Adachi; individualCount: 2; sex: male; lifeStage: adult; **Taxon:** kingdom: Animalia; phylum: Arthropoda; class: Insecta; order: Diptera; family: Canacidae; genus: Canaceoides; specificEpithet: Canaceoideshawaiiensis; scientificNameAuthorship: Wirth, 1969; **Location:** islandGroup: Hawaiian Islands; island: Oahu; verbatimLocality: Hanauma Bay; **Identification:** identifiedBy: DE Hardy & MD Delfinado; dateIdentified: 1980; **Event:** verbatimEventDate: 4.viii.1950
- **Type status:** Paratype. **Occurrence:** recordedBy: MS Adachi; individualCount: 2; sex: female; lifeStage: adult; **Taxon:** kingdom: Animalia; phylum: Arthropoda; class: Insecta; order: Diptera; family: Canacidae; genus: Canaceoides; specificEpithet: Canaceoideshawaiiensis; scientificNameAuthorship: Wirth, 1969; **Location:** islandGroup: Hawaiian Islands; island: Oahu; verbatimLocality: Hanauma Bay; **Identification:** identifiedBy: DE Hardy & MD Delfinado; dateIdentified: 1980; **Event:** verbatimEventDate: 4.viii.1950
- **Type status:** Paratype. **Occurrence:** recordedBy: MS Adachi; individualCount: 2; sex: female; lifeStage: adult; **Taxon:** kingdom: Animalia; phylum: Arthropoda; class: Insecta; order: Diptera; family: Canacidae; genus: Canaceoides; specificEpithet: Canaceoideshawaiiensis; scientificNameAuthorship: Wirth, 1969; **Location:** islandGroup: Hawaiian Islands; island: Oahu; verbatimLocality: Waimanalo; **Identification:** identifiedBy: DE Hardy & MD Delfinado; dateIdentified: 1980; **Event:** verbatimEventDate: 4.viii.1951; **Record Level:** institutionCode: UHM
- **Type status:** Paratype. **Occurrence:** recordedBy: HA Bess; individualCount: 13; sex: male; lifeStage: adult; **Taxon:** kingdom: Animalia; phylum: Arthropoda; class: Insecta; order: Diptera; family: Canacidae; genus: Canaceoides; specificEpithet: Canaceoideshawaiiensis; scientificNameAuthorship: Wirth, 1969; **Location:** islandGroup: Hawaiian Islands; island: Hawaii; verbatimLocality: Honaunau, Kona; **Identification:** identifiedBy: DE Hardy & MD Delfinado; dateIdentified: 1980; **Event:** verbatimEventDate: x.1951; **Record Level:** institutionCode: UHM
- **Type status:** Paratype. **Occurrence:** recordedBy: HA Bess; individualCount: 3; sex: female; lifeStage: adult; **Taxon:** kingdom: Animalia; phylum: Arthropoda; class: Insecta; order: Diptera; family: Canacidae; genus: Canaceoides; specificEpithet: Canaceoideshawaiiensis; scientificNameAuthorship: Wirth, 1969; **Location:** islandGroup: Hawaiian Islands; island: Hawaii; verbatimLocality: Honaunau, Kona; **Identification:** identifiedBy: DE Hardy & MD Delfinado; dateIdentified: 1980; **Event:** verbatimEventDate: x.1951; **Record Level:** institutionCode: UHM
- **Type status:** Paratype. **Occurrence:** recordedBy: DE Hardy; individualCount: 2; sex: male; lifeStage: adult; **Taxon:** kingdom: Animalia; phylum: Arthropoda; class: Insecta; order: Diptera; family: Canacidae; genus: Canaceoides; specificEpithet: Canaceoideshawaiiensis; scientificNameAuthorship: Wirth, 1969; **Location:** islandGroup: Hawaiian Islands; island: Oahu; verbatimLocality: Koko Head; **Identification:** identifiedBy: DE Hardy & MD Delfinado; dateIdentified: 1980; **Event:** verbatimEventDate: vi.1955
- **Type status:** Paratype. **Occurrence:** recordedBy: Y Kondo; individualCount: 5; sex: male; lifeStage: adult; **Taxon:** kingdom: Animalia; phylum: Arthropoda; class: Insecta; order: Diptera; family: Canacidae; genus: Canaceoides; specificEpithet: Canaceoideshawaiiensis; scientificNameAuthorship: Wirth, 1969; **Location:** islandGroup: Hawaiian Islands; island: Oahu; verbatimLocality: Waianae; **Identification:** identifiedBy: DE Hardy & MD Delfinado; dateIdentified: 1980; **Event:** verbatimEventDate: v.1958; **Record Level:** institutionCode: UHM
- **Type status:** Paratype. **Occurrence:** recordedBy: Y Kondo; individualCount: 4; sex: female; lifeStage: adult; **Taxon:** kingdom: Animalia; phylum: Arthropoda; class: Insecta; order: Diptera; family: Canacidae; genus: Canaceoides; specificEpithet: Canaceoideshawaiiensis; scientificNameAuthorship: Wirth, 1969; **Location:** islandGroup: Hawaiian Islands; island: Oahu; verbatimLocality: Waianae; **Identification:** identifiedBy: DE Hardy & MD Delfinado; dateIdentified: 1980; **Event:** verbatimEventDate: v.1958
- **Type status:** Paratype. **Occurrence:** recordedBy: NLH Krauss; individualCount: 1; sex: male; lifeStage: adult; **Taxon:** kingdom: Animalia; phylum: Arthropoda; class: Insecta; order: Diptera; family: Canacidae; genus: Canaceoides; specificEpithet: Canaceoideshawaiiensis; scientificNameAuthorship: Wirth, 1969; **Location:** islandGroup: Hawaiian Islands; island: Hawaii; verbatimLocality: on lava at edge of sea; **Identification:** identifiedBy: DE Hardy & MD Delfinado; dateIdentified: 1980; **Event:** verbatimEventDate: 10.ii.1965
- **Type status:** Paratype. **Occurrence:** recordedBy: NLH Krauss; individualCount: 3; sex: female; lifeStage: adult; **Taxon:** kingdom: Animalia; phylum: Arthropoda; class: Insecta; order: Diptera; family: Canacidae; genus: Canaceoides; specificEpithet: Canaceoideshawaiiensis; scientificNameAuthorship: Wirth, 1969; **Location:** islandGroup: Hawaiian Islands; island: Hawaii; verbatimLocality: on lava at edge of sea; **Identification:** identifiedBy: DE Hardy & MD Delfinado; dateIdentified: 1980; **Event:** verbatimEventDate: 10.ii.1965
- **Type status:** Paratype. **Occurrence:** recordedBy: no collector given; individualCount: 4; sex: male; lifeStage: adult; **Taxon:** kingdom: Animalia; phylum: Arthropoda; class: Insecta; order: Diptera; family: Canacidae; genus: Canaceoides; specificEpithet: Canaceoideshawaiiensis; scientificNameAuthorship: Wirth, 1969; **Location:** islandGroup: Hawaiian Islands; island: Oahu; verbatimLocality: Sunset Beach, light trap; **Identification:** identifiedBy: DE Hardy & MD Delfinado; dateIdentified: 1980; **Event:** verbatimEventDate: 24.iii.1966; **Record Level:** institutionCode: UHM
- **Type status:** Paratype. **Occurrence:** recordedBy: no collector given; individualCount: 2; sex: female; lifeStage: adult; **Taxon:** kingdom: Animalia; phylum: Arthropoda; class: Insecta; order: Diptera; family: Canacidae; genus: Canaceoides; specificEpithet: Canaceoideshawaiiensis; scientificNameAuthorship: Wirth, 1969; **Location:** islandGroup: Hawaiian Islands; island: Oahu; verbatimLocality: Sunset Beach, light trap; **Identification:** identifiedBy: DE Hardy & MD Delfinado; dateIdentified: 1980; **Event:** verbatimEventDate: 24.iii.1966; **Record Level:** institutionCode: UHM
- **Type status:** Paratype. **Occurrence:** recordedBy: JR Vockeroth; individualCount: 29; sex: male; lifeStage: adult; **Taxon:** kingdom: Animalia; phylum: Arthropoda; class: Insecta; order: Diptera; family: Canacidae; genus: Canaceoides; specificEpithet: Canaceoideshawaiiensis; scientificNameAuthorship: Wirth, 1969; **Location:** islandGroup: Hawaiian Islands; island: Oahu; verbatimLocality: Honolulu, wave swept rocks; **Identification:** identifiedBy: DE Hardy & MD Delfinado; dateIdentified: 1980; **Event:** verbatimEventDate: 24.viii.1966
- **Type status:** Paratype. **Occurrence:** recordedBy: JR Vockeroth; individualCount: 30; sex: female; lifeStage: adult; **Taxon:** kingdom: Animalia; phylum: Arthropoda; class: Insecta; order: Diptera; family: Canacidae; genus: Canaceoides; specificEpithet: Canaceoideshawaiiensis; scientificNameAuthorship: Wirth, 1969; **Location:** islandGroup: Hawaiian Islands; island: Oahu; verbatimLocality: Honolulu, wave swept rocks; **Identification:** identifiedBy: DE Hardy & MD Delfinado; dateIdentified: 1980; **Event:** verbatimEventDate: 24.viii.1966
- **Type status:** Paratype. **Occurrence:** recordedBy: T. Saigusa; individualCount: 5; sex: male; lifeStage: adult; **Taxon:** kingdom: Animalia; phylum: Arthropoda; class: Insecta; order: Diptera; family: Canacidae; genus: Canaceoides; specificEpithet: Canaceoideshawaiiensis; scientificNameAuthorship: Wirth, 1969; **Location:** islandGroup: Hawaiian Islands; island: Maui; verbatimLocality: Kaanapali; **Identification:** identifiedBy: DE Hardy & MD Delfinado; dateIdentified: 1980; **Event:** verbatimEventDate: 28.x.1966; **Record Level:** institutionCode: CNC
- **Type status:** Paratype. **Occurrence:** recordedBy: T. Saigusa; individualCount: 2; sex: female; lifeStage: adult; **Taxon:** kingdom: Animalia; phylum: Arthropoda; class: Insecta; order: Diptera; family: Canacidae; genus: Canaceoides; specificEpithet: Canaceoideshawaiiensis; scientificNameAuthorship: Wirth, 1969; **Location:** islandGroup: Hawaiian Islands; island: Maui; verbatimLocality: Kaanapali; **Identification:** identifiedBy: DE Hardy & MD Delfinado; dateIdentified: 1980; **Event:** verbatimEventDate: 28.x.1966; **Record Level:** institutionCode: CNC
- **Type status:** Paratype. **Occurrence:** recordedBy: JR Vockeroth; individualCount: 1; sex: male; lifeStage: adult; **Taxon:** kingdom: Animalia; phylum: Arthropoda; class: Insecta; order: Diptera; family: Canacidae; genus: Canaceoides; specificEpithet: Canaceoideshawaiiensis; scientificNameAuthorship: Wirth, 1969; **Location:** islandGroup: Hawaiian Islands; island: Hawaii; verbatimLocality: Kalapana Park; **Identification:** identifiedBy: DE Hardy & MD Delfinado; dateIdentified: 1980; **Event:** verbatimEventDate: 24.iii.1967; **Record Level:** institutionCode: CNC
- **Type status:** Paratype. **Occurrence:** recordedBy: DE Hardy; individualCount: 3; sex: female; lifeStage: adult; **Taxon:** kingdom: Animalia; phylum: Arthropoda; class: Insecta; order: Diptera; family: Canacidae; genus: Canaceoides; specificEpithet: Canaceoideshawaiiensis; scientificNameAuthorship: Wirth, 1969; **Location:** islandGroup: Hawaiian Islands; island: Maui; verbatimLocality: Hana; **Identification:** identifiedBy: DE Hardy & MD Delfinado; dateIdentified: 1980; **Event:** verbatimEventDate: 20.vi.1967; **Record Level:** institutionCode: BPBM
- **Type status:** Paratype. **Occurrence:** recordedBy: DE Hardy; individualCount: 21; sex: male; lifeStage: adult; **Taxon:** kingdom: Animalia; phylum: Arthropoda; class: Insecta; order: Diptera; family: Canacidae; genus: Canaceoides; specificEpithet: Canaceoideshawaiiensis; scientificNameAuthorship: Wirth, 1969; **Location:** islandGroup: Hawaiian Islands; island: Maui; verbatimLocality: Hana; **Identification:** identifiedBy: DE Hardy & MD Delfinado; dateIdentified: 1980; **Event:** verbatimEventDate: 20.vi.1967; **Record Level:** institutionCode: BPBM
- **Type status:** Other material. **Occurrence:** catalogNumber: 2008008278; recordedBy: EH Bryan, Jr.; lifeStage: adult; **Taxon:** kingdom: Animalia; phylum: Arthropoda; class: Insecta; order: Diptera; family: Canacidae; genus: Canaceoides; specificEpithet: Canaceoideshawaiiensis; scientificNameAuthorship: Wirth, 1969; **Location:** islandGroup: Hawaiian Islands; island: Nihoa; **Identification:** identifiedBy: J Straxanac; dateIdentified: 1991; **Event:** verbatimEventDate: 12.vi.1923; **Record Level:** institutionCode: BPBM
- **Type status:** Other material. **Occurrence:** catalogNumber: 2006004951; recordedBy: HA Bess; lifeStage: adult; **Taxon:** kingdom: Animalia; phylum: Arthropoda; class: Insecta; order: Diptera; family: Canacidae; genus: Canaceoides; specificEpithet: Canaceoideshawaiiensis; scientificNameAuthorship: Wirth, 1969; **Location:** islandGroup: Hawaiian Islands; island: Hawaii; verbatimLocality: Honaunau; **Identification:** identifiedBy: WW Wirth; dateIdentified: 1969; **Event:** verbatimEventDate: October 1951; **Record Level:** institutionCode: BPBM
- **Type status:** Other material. **Occurrence:** catalogNumber: 2006004948; recordedBy: HA Bess; lifeStage: adult; **Taxon:** kingdom: Animalia; phylum: Arthropoda; class: Insecta; order: Diptera; family: Canacidae; genus: Canaceoides; specificEpithet: Canaceoideshawaiiensis; scientificNameAuthorship: Wirth, 1969; **Location:** islandGroup: Hawaiian Islands; island: Hawaii; verbatimLocality: Honaunau; **Identification:** identifiedBy: WW Wirth; dateIdentified: 1969; **Event:** verbatimEventDate: October 1951; **Record Level:** institutionCode: BPBM
- **Type status:** Other material. **Occurrence:** catalogNumber: 2006004950; recordedBy: HA Bess; lifeStage: adult; **Taxon:** kingdom: Animalia; phylum: Arthropoda; class: Insecta; order: Diptera; family: Canacidae; genus: Canaceoides; specificEpithet: Canaceoideshawaiiensis; scientificNameAuthorship: Wirth, 1969; **Location:** islandGroup: Hawaiian Islands; island: Hawaii; verbatimLocality: Honaunau; **Identification:** identifiedBy: WW Wirth; dateIdentified: 1969; **Event:** verbatimEventDate: x.1951; **Record Level:** institutionCode: BPBM
- **Type status:** Other material. **Occurrence:** catalogNumber: 2006004949; recordedBy: HA Bess; lifeStage: adult; **Taxon:** kingdom: Animalia; phylum: Arthropoda; class: Insecta; order: Diptera; family: Canacidae; genus: Canaceoides; specificEpithet: Canaceoideshawaiiensis; scientificNameAuthorship: Wirth, 1969; **Location:** islandGroup: Hawaiian Islands; island: Hawaii; verbatimLocality: Honaunau; **Identification:** identifiedBy: WW Wirth; dateIdentified: 1969; **Event:** verbatimEventDate: x.1951; **Record Level:** institutionCode: BPBM
- **Type status:** Other material. **Occurrence:** catalogNumber: 2006004946; recordedBy: Y Kondo; lifeStage: adult; **Taxon:** kingdom: Animalia; phylum: Arthropoda; class: Insecta; order: Diptera; family: Canacidae; genus: Canaceoides; specificEpithet: Canaceoideshawaiiensis; scientificNameAuthorship: Wirth, 1969; **Location:** islandGroup: Hawaiian Islands; island: Oahu; verbatimLocality: Waianae; **Identification:** identifiedBy: WW Wirth; dateIdentified: 1969; **Event:** verbatimEventDate: May 1958; **Record Level:** institutionCode: BPBM
- **Type status:** Other material. **Occurrence:** catalogNumber: 2006004947; recordedBy: Y Kondo; lifeStage: adult; **Taxon:** kingdom: Animalia; phylum: Arthropoda; class: Insecta; order: Diptera; family: Canacidae; genus: Canaceoides; specificEpithet: Canaceoideshawaiiensis; scientificNameAuthorship: Wirth, 1969; **Location:** islandGroup: Hawaiian Islands; island: Oahu; verbatimLocality: Waianae; **Identification:** identifiedBy: WW Wirth; dateIdentified: 1969; **Event:** verbatimEventDate: May 1958; **Record Level:** institutionCode: BPBM
- **Type status:** Other material. **Occurrence:** catalogNumber: 2008008266; recordedBy: WC Gagne; lifeStage: adult; **Taxon:** kingdom: Animalia; phylum: Arthropoda; class: Insecta; order: Diptera; family: Canacidae; genus: Canaceoides; specificEpithet: Canaceoideshawaiiensis; scientificNameAuthorship: Wirth, 1969; **Location:** islandGroup: Hawaiian Islands; island: Nihoa; verbatimLocality: on coast; **Identification:** identifiedBy: DE Hardy; **Event:** verbatimEventDate: 20.iv.1983; **Record Level:** institutionCode: BPBM
- **Type status:** Other material. **Occurrence:** catalogNumber: 2008008267; recordedBy: WC Gagne; lifeStage: adult; **Taxon:** kingdom: Animalia; phylum: Arthropoda; class: Insecta; order: Diptera; family: Canacidae; genus: Canaceoides; specificEpithet: Canaceoideshawaiiensis; scientificNameAuthorship: Wirth, 1969; **Location:** islandGroup: Hawaiian Islands; island: Nihoa; verbatimLocality: on coast; **Identification:** identifiedBy: DE Hardy; **Event:** verbatimEventDate: 20.iv.1983; **Record Level:** institutionCode: BPBM
- **Type status:** Other material. **Occurrence:** catalogNumber: 2008008268; recordedBy: WC Gagne; lifeStage: adult; **Taxon:** kingdom: Animalia; phylum: Arthropoda; class: Insecta; order: Diptera; family: Canacidae; genus: Canaceoides; specificEpithet: Canaceoideshawaiiensis; scientificNameAuthorship: Wirth, 1969; **Location:** islandGroup: Hawaiian Islands; island: Nihoa; verbatimLocality: on coast; **Identification:** identifiedBy: DE Hardy; **Event:** verbatimEventDate: 20.iv.1983; **Record Level:** institutionCode: BPBM
- **Type status:** Other material. **Occurrence:** catalogNumber: 2008008269; recordedBy: WC Gagne; lifeStage: adult; **Taxon:** kingdom: Animalia; phylum: Arthropoda; class: Insecta; order: Diptera; family: Canacidae; genus: Canaceoides; specificEpithet: Canaceoideshawaiiensis; scientificNameAuthorship: Wirth, 1969; **Location:** islandGroup: Hawaiian Islands; island: Nihoa; verbatimLocality: on coast; **Identification:** identifiedBy: DE Hardy; **Event:** verbatimEventDate: 20.iv.1983; **Record Level:** institutionCode: BPBM
- **Type status:** Other material. **Occurrence:** catalogNumber: 2008008263; recordedBy: WC Gagne; lifeStage: adult; **Taxon:** kingdom: Animalia; phylum: Arthropoda; class: Insecta; order: Diptera; family: Canacidae; genus: Canaceoides; specificEpithet: Canaceoideshawaiiensis; scientificNameAuthorship: Wirth, 1969; **Location:** islandGroup: Hawaiian Islands; island: Nihoa; verbatimLocality: on coast; **Identification:** identifiedBy: DE Hardy; **Event:** verbatimEventDate: 20.iv.1983; **Record Level:** institutionCode: BPBM
- **Type status:** Other material. **Occurrence:** catalogNumber: 2008008276; recordedBy: WC Gagne; lifeStage: adult; **Taxon:** kingdom: Animalia; phylum: Arthropoda; class: Insecta; order: Diptera; family: Canacidae; genus: Canaceoides; specificEpithet: Canaceoideshawaiiensis; scientificNameAuthorship: Wirth, 1969; **Location:** islandGroup: Hawaiian Islands; island: Nihoa; verbatimLocality: on coast; **Identification:** identifiedBy: DE Hardy; **Event:** verbatimEventDate: 20.iv.1983; **Record Level:** institutionCode: BPBM
- **Type status:** Other material. **Occurrence:** catalogNumber: 2008008257; recordedBy: WC Gagne; lifeStage: adult; **Taxon:** kingdom: Animalia; phylum: Arthropoda; class: Insecta; order: Diptera; family: Canacidae; genus: Canaceoides; specificEpithet: Canaceoideshawaiiensis; scientificNameAuthorship: Wirth, 1969; **Location:** islandGroup: Hawaiian Islands; island: Nihoa; verbatimLocality: on coast; **Identification:** identifiedBy: DE Hardy; **Event:** verbatimEventDate: 20.iv.1983; **Record Level:** institutionCode: BPBM
- **Type status:** Other material. **Occurrence:** catalogNumber: 2008008254; recordedBy: WC Gagne; lifeStage: adult; **Taxon:** kingdom: Animalia; phylum: Arthropoda; class: Insecta; order: Diptera; family: Canacidae; genus: Canaceoides; specificEpithet: Canaceoideshawaiiensis; scientificNameAuthorship: Wirth, 1969; **Location:** islandGroup: Hawaiian Islands; island: Nihoa; verbatimLocality: on coast; **Identification:** identifiedBy: DE Hardy; **Event:** verbatimEventDate: 20.iv.1983; **Record Level:** institutionCode: BPBM
- **Type status:** Other material. **Occurrence:** catalogNumber: 2008008265; recordedBy: WC Gagne; lifeStage: adult; **Taxon:** kingdom: Animalia; phylum: Arthropoda; class: Insecta; order: Diptera; family: Canacidae; genus: Canaceoides; specificEpithet: Canaceoideshawaiiensis; scientificNameAuthorship: Wirth, 1969; **Location:** islandGroup: Hawaiian Islands; island: Nihoa; verbatimLocality: on coast; **Identification:** identifiedBy: DE Hardy; **Event:** verbatimEventDate: 20.iv.1983; **Record Level:** institutionCode: BPBM
- **Type status:** Other material. **Occurrence:** catalogNumber: 2008008256; recordedBy: WC Gagne; lifeStage: adult; **Taxon:** kingdom: Animalia; phylum: Arthropoda; class: Insecta; order: Diptera; family: Canacidae; genus: Canaceoides; specificEpithet: Canaceoideshawaiiensis; scientificNameAuthorship: Wirth, 1969; **Location:** islandGroup: Hawaiian Islands; island: Nihoa; verbatimLocality: on coast; **Identification:** identifiedBy: DE Hardy; **Event:** verbatimEventDate: 20.iv.1983; **Record Level:** institutionCode: BPBM
- **Type status:** Other material. **Occurrence:** catalogNumber: 2008008259; recordedBy: WC Gagne; lifeStage: adult; **Taxon:** kingdom: Animalia; phylum: Arthropoda; class: Insecta; order: Diptera; family: Canacidae; genus: Canaceoides; specificEpithet: Canaceoideshawaiiensis; scientificNameAuthorship: Wirth, 1969; **Location:** islandGroup: Hawaiian Islands; island: Nihoa; verbatimLocality: on coast; **Identification:** identifiedBy: DE Hardy; **Event:** verbatimEventDate: 20.iv.1983; **Record Level:** institutionCode: BPBM
- **Type status:** Other material. **Occurrence:** catalogNumber: 2008008264; recordedBy: WC Gagne; lifeStage: adult; **Taxon:** kingdom: Animalia; phylum: Arthropoda; class: Insecta; order: Diptera; family: Canacidae; genus: Canaceoides; specificEpithet: Canaceoideshawaiiensis; scientificNameAuthorship: Wirth, 1969; **Location:** islandGroup: Hawaiian Islands; island: Nihoa; verbatimLocality: on coast; **Identification:** identifiedBy: DE Hardy; **Event:** verbatimEventDate: 20.iv.1983; **Record Level:** institutionCode: BPBM
- **Type status:** Other material. **Occurrence:** catalogNumber: 2008008260; recordedBy: WC Gagne; lifeStage: adult; **Taxon:** kingdom: Animalia; phylum: Arthropoda; class: Insecta; order: Diptera; family: Canacidae; genus: Canaceoides; specificEpithet: Canaceoideshawaiiensis; scientificNameAuthorship: Wirth, 1969; **Location:** islandGroup: Hawaiian Islands; island: Nihoa; verbatimLocality: on coast; **Identification:** identifiedBy: DE Hardy; **Event:** verbatimEventDate: 20.iv.1983; **Record Level:** institutionCode: BPBM
- **Type status:** Other material. **Occurrence:** catalogNumber: 2008008261; recordedBy: WC Gagne; lifeStage: adult; **Taxon:** kingdom: Animalia; phylum: Arthropoda; class: Insecta; order: Diptera; family: Canacidae; genus: Canaceoides; specificEpithet: Canaceoideshawaiiensis; scientificNameAuthorship: Wirth, 1969; **Location:** islandGroup: Hawaiian Islands; island: Nihoa; verbatimLocality: on coast; **Identification:** identifiedBy: DE Hardy; **Event:** verbatimEventDate: 20.iv.1983; **Record Level:** institutionCode: BPBM
- **Type status:** Other material. **Occurrence:** catalogNumber: 2008008262; recordedBy: WC Gagne; lifeStage: adult; **Taxon:** kingdom: Animalia; phylum: Arthropoda; class: Insecta; order: Diptera; family: Canacidae; genus: Canaceoides; specificEpithet: Canaceoideshawaiiensis; scientificNameAuthorship: Wirth, 1969; **Location:** islandGroup: Hawaiian Islands; island: Nihoa; verbatimLocality: on coast; **Identification:** identifiedBy: DE Hardy; **Event:** verbatimEventDate: 20.iv.1983; **Record Level:** institutionCode: BPBM
- **Type status:** Other material. **Occurrence:** catalogNumber: 2008008255; recordedBy: WC Gagne; lifeStage: adult; **Taxon:** kingdom: Animalia; phylum: Arthropoda; class: Insecta; order: Diptera; family: Canacidae; genus: Canaceoides; specificEpithet: Canaceoideshawaiiensis; scientificNameAuthorship: Wirth, 1969; **Location:** islandGroup: Hawaiian Islands; island: Nihoa; verbatimLocality: on coast; **Identification:** identifiedBy: DE Hardy; **Event:** verbatimEventDate: 20.iv.1983; **Record Level:** institutionCode: BPBM
- **Type status:** Other material. **Occurrence:** catalogNumber: 2008008270; recordedBy: J Strazanac; lifeStage: adult; **Taxon:** kingdom: Animalia; phylum: Arthropoda; class: Insecta; order: Diptera; family: Canacidae; genus: Canaceoides; specificEpithet: Canaceoideshawaiiensis; scientificNameAuthorship: Wirth, 1969; **Location:** islandGroup: Hawaiian Islands; island: Nihoa; verbatimLocality: Miller Valley; minimumElevationInMeters: 10; **Identification:** identifiedBy: DE Hardy; **Event:** verbatimEventDate: 25.vi.1990; **Record Level:** institutionCode: BPBM
- **Type status:** Other material. **Occurrence:** catalogNumber: 2008008271; recordedBy: J Strazanac; lifeStage: adult; **Taxon:** kingdom: Animalia; phylum: Arthropoda; class: Insecta; order: Diptera; family: Canacidae; genus: Canaceoides; specificEpithet: Canaceoideshawaiiensis; scientificNameAuthorship: Wirth, 1969; **Location:** islandGroup: Hawaiian Islands; island: Nihoa; verbatimLocality: Miller Valley; minimumElevationInMeters: 10; **Identification:** identifiedBy: DE Hardy; **Event:** verbatimEventDate: 25.vi.1990; **Record Level:** institutionCode: BPBM
- **Type status:** Other material. **Occurrence:** catalogNumber: 2008008272; recordedBy: J Strazanac; lifeStage: adult; **Taxon:** kingdom: Animalia; phylum: Arthropoda; class: Insecta; order: Diptera; family: Canacidae; genus: Canaceoides; specificEpithet: Canaceoideshawaiiensis; scientificNameAuthorship: Wirth, 1969; **Location:** islandGroup: Hawaiian Islands; island: Nihoa; verbatimLocality: Miller Valley; minimumElevationInMeters: 10; **Identification:** identifiedBy: DE Hardy; **Event:** verbatimEventDate: 25.vi.1990; **Record Level:** institutionCode: BPBM
- **Type status:** Other material. **Occurrence:** catalogNumber: 2008008273; recordedBy: J Strazanac; lifeStage: adult; **Taxon:** kingdom: Animalia; phylum: Arthropoda; class: Insecta; order: Diptera; family: Canacidae; genus: Canaceoides; specificEpithet: Canaceoideshawaiiensis; scientificNameAuthorship: Wirth, 1969; **Location:** islandGroup: Hawaiian Islands; island: Nihoa; verbatimLocality: Miller Valley; minimumElevationInMeters: 10; **Identification:** identifiedBy: DE Hardy; **Event:** verbatimEventDate: 25.vi.1990; **Record Level:** institutionCode: BPBM
- **Type status:** Other material. **Occurrence:** catalogNumber: 2008008274; recordedBy: J Strazanac; lifeStage: adult; **Taxon:** kingdom: Animalia; phylum: Arthropoda; class: Insecta; order: Diptera; family: Canacidae; genus: Canaceoides; specificEpithet: Canaceoideshawaiiensis; scientificNameAuthorship: Wirth, 1969; **Location:** islandGroup: Hawaiian Islands; island: Nihoa; verbatimLocality: Miller Valley; minimumElevationInMeters: 10; **Identification:** identifiedBy: DE Hardy; **Event:** verbatimEventDate: 25.vi.1990; **Record Level:** institutionCode: BPBM
- **Type status:** Other material. **Occurrence:** catalogNumber: 2008008275; recordedBy: J Strazanac; lifeStage: adult; **Taxon:** kingdom: Animalia; phylum: Arthropoda; class: Insecta; order: Diptera; family: Canacidae; genus: Canaceoides; specificEpithet: Canaceoideshawaiiensis; scientificNameAuthorship: Wirth, 1969; **Location:** islandGroup: Hawaiian Islands; island: Nihoa; verbatimLocality: Miller Valley; minimumElevationInMeters: 10; **Identification:** identifiedBy: DE Hardy; **Event:** verbatimEventDate: 25.vi.1990; **Record Level:** institutionCode: BPBM
- **Type status:** Other material. **Occurrence:** catalogNumber: 2008008277; recordedBy: J Strazanac; lifeStage: adult; **Taxon:** kingdom: Animalia; phylum: Arthropoda; class: Insecta; order: Diptera; family: Canacidae; genus: Canaceoides; specificEpithet: Canaceoideshawaiiensis; scientificNameAuthorship: Wirth, 1969; **Location:** islandGroup: Hawaiian Islands; island: Nihoa; verbatimLocality: Miller Valley; minimumElevationInMeters: 10; **Identification:** identifiedBy: DE Hardy; **Event:** verbatimEventDate: 25.vi.1990; **Record Level:** institutionCode: BPBM
- **Type status:** Other material. **Occurrence:** catalogNumber: 2008008258; recordedBy: J Strazanac; lifeStage: adult; **Taxon:** kingdom: Animalia; phylum: Arthropoda; class: Insecta; order: Diptera; family: Canacidae; genus: Canaceoides; specificEpithet: Canaceoideshawaiiensis; scientificNameAuthorship: Wirth, 1969; **Location:** islandGroup: Hawaiian Islands; island: Nihoa; verbatimLocality: Miller Valley; minimumElevationInMeters: 10; **Identification:** identifiedBy: DE Hardy; **Event:** verbatimEventDate: 25.vi.1990; **Record Level:** institutionCode: BPBM
- **Type status:** Other material. **Occurrence:** catalogNumber: 2008000626; recordedBy: K Arakaki, GA Samuelson, K Kami; lifeStage: adult; **Taxon:** kingdom: Animalia; phylum: Arthropoda; class: Insecta; order: Diptera; family: Canacidae; genus: Canaceoides; specificEpithet: Canaceoideshawaiiensis; scientificNameAuthorship: Wirth, 1969; **Location:** islandGroup: Hawaiian Islands; island: Oahu; verbatimLocality: Kalauao Ponds, Ewa Stream; minimumElevationInMeters: 0; **Identification:** identifiedBy: K Arakaki; **Event:** verbatimEventDate: 29.vi.1998; eventRemarks: Pearl Harbor Survey; **Record Level:** institutionCode: BPBM
- **Type status:** Other material. **Occurrence:** recordedBy: NL Evenhuis; lifeStage: adult; **Taxon:** kingdom: Animalia; phylum: Arthropoda; class: Insecta; order: Diptera; family: Canacidae; genus: Canaceoides; specificEpithet: Canaceoideshawaiiensis; scientificNameAuthorship: Wirth, 1969; **Location:** islandGroup: Hawaiian Islands; island: Hawaii; verbatimLocality: Ninole, Kauwale Fish Pond; **Identification:** identifiedBy: PM O'Grady; dateIdentified: 2014; **Event:** verbatimEventDate: 26.vi.2010; **Record Level:** institutionCode: EMEC; collectionCode: 205542
- **Type status:** Other material. **Occurrence:** recordedBy: PM O'Grady, B Ort, NA Pantoja; lifeStage: adult; **Taxon:** kingdom: Animalia; phylum: Arthropoda; class: Insecta; order: Diptera; family: Canacidae; genus: Canaceoides; specificEpithet: Canaceoideshawaiiensis; scientificNameAuthorship: Wirth, 1969; **Location:** islandGroup: Hawaiian Islands; island: Hawaii; verbatimLocality: Whittington Beach Park, South Coast, Kau; **Identification:** identifiedBy: PM O'Grady; dateIdentified: 2014; **Event:** verbatimEventDate: 1.viii.2010; **Record Level:** institutionCode: EMEC; collectionCode: 205121
- **Type status:** Other material. **Occurrence:** recordedBy: PM O'Grady, BS Ort, NA Pantoja; lifeStage: adult; **Taxon:** kingdom: Animalia; phylum: Arthropoda; class: Insecta; order: Diptera; family: Canacidae; genus: Canaceoides; specificEpithet: Canaceoideshawaiiensis; scientificNameAuthorship: Wirth, 1969; **Location:** islandGroup: Hawaiian Islands; island: Hawaii; verbatimLocality: Whittington Beach Park, South Coast, Kau, sweeping lava rocks and estuary; **Identification:** identifiedBy: PM O'Grady; dateIdentified: 2014; **Event:** verbatimEventDate: 1.viii.2010; **Record Level:** institutionCode: EMEC; collectionCode: 612.6
- **Type status:** Other material. **Occurrence:** recordedBy: PM O'Grady, BS Ort, RT Lapoint, GM Bennett, NA Pantoja; lifeStage: adult; **Taxon:** kingdom: Animalia; phylum: Arthropoda; class: Insecta; order: Diptera; family: Canacidae; genus: Canaceoides; specificEpithet: Canaceoideshawaiiensis; scientificNameAuthorship: Wirth, 1969; **Location:** islandGroup: Hawaiian Islands; island: Hawaii; verbatimLocality: Alakahi Trail to Onomea Bay, adjacent to Hawaii Botanical Garden, sweeping rocks in surf; **Identification:** identifiedBy: PM O'Grady; dateIdentified: 2014; **Event:** verbatimEventDate: 2.viii.2010; **Record Level:** institutionCode: EMEC; collectionCode: 614.4
- **Type status:** Other material. **Occurrence:** recordedBy: PM O'Grady, BS Ort, RT Lapoint, GM Bennett, NA Pantoja; lifeStage: adult; **Taxon:** kingdom: Animalia; phylum: Arthropoda; class: Insecta; order: Diptera; family: Canacidae; genus: Canaceoides; specificEpithet: Canaceoideshawaiiensis; scientificNameAuthorship: Wirth, 1969; **Location:** islandGroup: Hawaiian Islands; island: Hawaii; verbatimLocality: Kolekole Beach Park, sweeping along ocean coast; **Identification:** identifiedBy: PM O'Grady; dateIdentified: 2014; **Event:** verbatimEventDate: 2.viii.2010; **Record Level:** institutionCode: EMEC; collectionCode: 616.3
- **Type status:** Other material. **Occurrence:** recordedBy: PM O'Grady, RT Lapoint, GM Bennett, NA Pantoja; lifeStage: adult; **Taxon:** kingdom: Animalia; phylum: Arthropoda; class: Insecta; order: Diptera; family: Canacidae; genus: Canaceoides; specificEpithet: Canaceoideshawaiiensis; scientificNameAuthorship: Wirth, 1969; **Location:** islandGroup: Hawaiian Islands; island: Hawaii; verbatimLocality: Alakahi Trail to Onomea Bay, adjacent to Hawaiia Botanical Garden; **Identification:** identifiedBy: PM O'Grady; dateIdentified: 2014; **Event:** verbatimEventDate: 2.viii.2010; **Record Level:** institutionCode: EMEC; collectionCode: 205130
- **Type status:** Other material. **Occurrence:** recordedBy: BS Ort; lifeStage: adult; **Taxon:** kingdom: Animalia; phylum: Arthropoda; class: Insecta; order: Diptera; family: Canacidae; genus: Canaceoides; specificEpithet: Canaceoideshawaiiensis; scientificNameAuthorship: Wirth, 1969; **Location:** islandGroup: Hawaiian Islands; island: Hawaii; verbatimLocality: Alakahi Trail to Onomea Bay, adjacent to Hawaiia Botanical Garden; **Identification:** identifiedBy: PM O'Grady; dateIdentified: 2014; **Event:** verbatimEventDate: 2.viii.2010; **Record Level:** institutionCode: EMEC; collectionCode: 205636
- **Type status:** Other material. **Occurrence:** recordedBy: KR Goodman; lifeStage: adult; **Taxon:** kingdom: Animalia; phylum: Arthropoda; class: Insecta; order: Diptera; family: Canacidae; genus: Canaceoides; specificEpithet: Canaceoideshawaiiensis; scientificNameAuthorship: Wirth, 1969; **Location:** islandGroup: Hawaiian Islands; island: Molokai; verbatimLocality: South West Molokai, Hale O Lono; **Identification:** identifiedBy: PM O'Grady; dateIdentified: 2014; **Event:** verbatimEventDate: 12.i.2011; **Record Level:** institutionCode: EMEC; collectionCode: 205643
- **Type status:** Other material. **Occurrence:** recordedBy: BS Ort; lifeStage: adult; **Taxon:** kingdom: Animalia; phylum: Arthropoda; class: Insecta; order: Diptera; family: Canacidae; genus: Canaceoides; specificEpithet: Canaceoideshawaiiensis; scientificNameAuthorship: Wirth, 1969; **Location:** islandGroup: Hawaiian Islands; island: Oahu; verbatimLocality: Makapuu Point; **Identification:** identifiedBy: PM O'Grady; dateIdentified: 2014; **Event:** verbatimEventDate: 25.v.2011; **Record Level:** institutionCode: EMEC; collectionCode: 205641
- **Type status:** Other material. **Occurrence:** lifeStage: adult; **Taxon:** kingdom: Animalia; phylum: Arthropoda; class: Insecta; order: Diptera; family: Canacidae; genus: Canaceoides; specificEpithet: Canaceoideshawaiiensis; scientificNameAuthorship: Wirth, 1969; **Location:** islandGroup: Hawaiian Islands; island: Hawaii; **Identification:** identifiedBy: PM O'Grady; dateIdentified: 2014; **Record Level:** institutionCode: EMEC; collectionCode: 205725

##### Ecological interactions

###### Native status

endemic

##### Distribution

**Figure 9.**
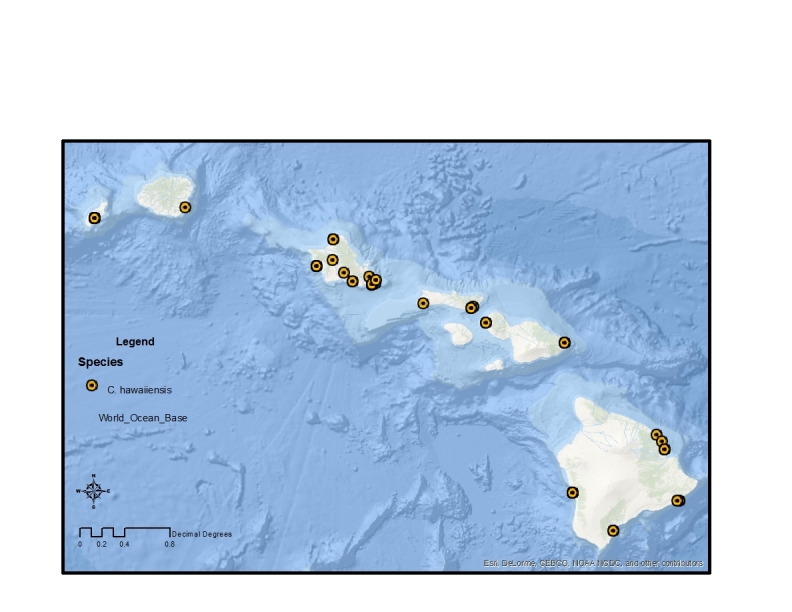
Distribution of *C. hawaiiensis* in the main Hawaiian Islands. This species is also found in the Northwest Hawaiian Islands (Nihoa).

HAWAIIAN ISLANDS: Nihoa, Kauai, Oahu, Molokai, Maui, Hawaii (Fig. 9).

##### Notes

Wirth (1969), [original description; male genitalia, female terminalia, ninth sternum, spermatheca]; Hardy and Delfinado (1980), [redescription and revision of Hawaiian taxa; female terminalia (dorsal and ventral), spermathecae, ninth sternum, epandrium and surstylus]; Mathis (1992), [World Catalog]; Nishida (2002), [Hawaiian Arthropod Checklist]; Evenhuis and Eldredge (2004), [Natural History of Nihoa and Necker Islands].

#### Procanace acuminata

Hardy and Delfinado, 1980

##### Materials

- **Type status:** Holotype. **Occurrence:** recordedBy: MD Delfinado; individualCount: 1; sex: male; lifeStage: adult; **Taxon:** kingdom: Animalia; phylum: Arthropoda; class: Insecta; order: Diptera; family: Canacidae; genus: Procanace; specificEpithet: Procanaceacuminata; scientificNameAuthorship: Hardy & Delfinado, 1980; **Location:** islandGroup: Hawaiian Islands; island: Hawaii; verbatimLocality: Kapue Stream, east slope of Mauna Kea; verbatimElevation: 1000 ft.; **Identification:** identifiedBy: DE Hardy & MD Delfinado; dateIdentified: 1980; **Event:** verbatimEventDate: 29.v.1970; **Record Level:** institutionCode: BPBM
- **Type status:** Paratype. **Occurrence:** recordedBy: DE Hardy; individualCount: 3; lifeStage: adult; **Taxon:** kingdom: Animalia; phylum: Arthropoda; class: Insecta; order: Diptera; family: Canacidae; genus: Procanace; specificEpithet: Procanaceacuminata; scientificNameAuthorship: Hardy & Delfinado, 1980; **Location:** islandGroup: Hawaiian Islands; island: Maui; verbatimLocality: Iao Valley; **Identification:** identifiedBy: DE Hardy & MD Delfinado; dateIdentified: 1980; **Event:** verbatimEventDate: vi.1952; **Record Level:** institutionCode: UHM
- **Type status:** Paratype. **Occurrence:** recordedBy: DE Hardy, JA Tenorio; lifeStage: adult; **Taxon:** kingdom: Animalia; phylum: Arthropoda; class: Insecta; order: Diptera; family: Canacidae; genus: Procanace; specificEpithet: Procanaceacuminata; scientificNameAuthorship: Hardy & Delfinado, 1980; **Location:** islandGroup: Hawaiian Islands; island: Maui; verbatimLocality: Iao Valley, on wet rocks in swift moving stream; **Identification:** identifiedBy: DE Hardy & MD Delfinado; dateIdentified: 1980; **Event:** verbatimEventDate: vi.1952
- **Type status:** Paratype. **Occurrence:** recordedBy: CR Joyce; individualCount: 3; lifeStage: adult; **Taxon:** kingdom: Animalia; phylum: Arthropoda; class: Insecta; order: Diptera; family: Canacidae; genus: Procanace; specificEpithet: Procanaceacuminata; scientificNameAuthorship: Hardy & Delfinado, 1980; **Location:** islandGroup: Hawaiian Islands; island: Maui; verbatimLocality: Wailua; **Identification:** identifiedBy: DE Hardy & MD Delfinado; dateIdentified: 1980; **Event:** verbatimEventDate: vi.1953; **Record Level:** institutionCode: UHM
- **Type status:** Paratype. **Occurrence:** recordedBy: CR Joyce; lifeStage: adult; **Taxon:** kingdom: Animalia; phylum: Arthropoda; class: Insecta; order: Diptera; family: Canacidae; genus: Procanace; specificEpithet: Procanaceacuminata; scientificNameAuthorship: Hardy & Delfinado, 1980; **Location:** islandGroup: Hawaiian Islands; island: Maui; verbatimLocality: Wailua; **Identification:** identifiedBy: DE Hardy & MD Delfinado; dateIdentified: 1980; **Event:** verbatimEventDate: vi.1953
- **Type status:** Paratype. **Occurrence:** recordedBy: JA Tenorio; individualCount: 1; lifeStage: adult; **Taxon:** kingdom: Animalia; phylum: Arthropoda; class: Insecta; order: Diptera; family: Canacidae; genus: Procanace; specificEpithet: Procanaceacuminata; scientificNameAuthorship: Hardy & Delfinado, 1980; **Location:** islandGroup: Hawaiian Islands; island: Maui; verbatimLocality: Iao Stream; **Identification:** identifiedBy: DE Hardy & MD Delfinado; dateIdentified: 1980; **Event:** verbatimEventDate: 13.ix.1968; **Record Level:** institutionCode: UHM
- **Type status:** Paratype. **Occurrence:** recordedBy: JA Tenorio; individualCount: 1; lifeStage: adult; **Taxon:** kingdom: Animalia; phylum: Arthropoda; class: Insecta; order: Diptera; family: Canacidae; genus: Procanace; specificEpithet: Procanaceacuminata; scientificNameAuthorship: Hardy & Delfinado, 1980; **Location:** islandGroup: Hawaiian Islands; island: Hawaii; verbatimLocality: Kawai-iki Stream; **Identification:** identifiedBy: DE Hardy & MD Delfinado; dateIdentified: 1980; **Event:** verbatimEventDate: 27.xii.1969; **Record Level:** institutionCode: UHM
- **Type status:** Paratype. **Occurrence:** recordedBy: JA Tenorio; lifeStage: adult; **Taxon:** kingdom: Animalia; phylum: Arthropoda; class: Insecta; order: Diptera; family: Canacidae; genus: Procanace; specificEpithet: Procanaceacuminata; scientificNameAuthorship: Hardy & Delfinado, 1980; **Location:** islandGroup: Hawaiian Islands; island: Hawaii; verbatimLocality: Kaiwiki Stream; **Identification:** identifiedBy: DE Hardy & MD Delfinado; dateIdentified: 1980; **Event:** verbatimEventDate: 27.xii.1969
- **Type status:** Paratype. **Occurrence:** recordedBy: JA Tenorio; individualCount: 1; lifeStage: adult; **Taxon:** kingdom: Animalia; phylum: Arthropoda; class: Insecta; order: Diptera; family: Canacidae; genus: Procanace; specificEpithet: Procanaceacuminata; scientificNameAuthorship: Hardy & Delfinado, 1980; **Location:** islandGroup: Hawaiian Islands; island: Hawaii; verbatimLocality: Honomu Maili Stream; **Identification:** identifiedBy: DE Hardy & MD Delfinado; dateIdentified: 1980; **Event:** verbatimEventDate: 28.ii.1970; **Record Level:** institutionCode: UHM
- **Type status:** Paratype. **Occurrence:** recordedBy: JA Tenorio; lifeStage: adult; **Taxon:** kingdom: Animalia; phylum: Arthropoda; class: Insecta; order: Diptera; family: Canacidae; genus: Procanace; specificEpithet: Procanaceacuminata; scientificNameAuthorship: Hardy & Delfinado, 1980; **Location:** islandGroup: Hawaiian Islands; island: Hawaii; verbatimLocality: Honomu Maiki Stream; **Identification:** identifiedBy: DE Hardy & MD Delfinado; dateIdentified: 1980; **Event:** verbatimEventDate: 28.ii.1970
- **Type status:** Paratype. **Occurrence:** recordedBy: JA Tenorio; individualCount: 10; lifeStage: adult; **Taxon:** kingdom: Animalia; phylum: Arthropoda; class: Insecta; order: Diptera; family: Canacidae; genus: Procanace; specificEpithet: Procanaceacuminata; scientificNameAuthorship: Hardy & Delfinado, 1980; **Location:** islandGroup: Hawaiian Islands; island: Maui; verbatimLocality: Iao Valley; **Identification:** identifiedBy: DE Hardy & MD Delfinado; dateIdentified: 1980; **Event:** verbatimEventDate: 27.iii.1970; **Record Level:** institutionCode: UHM
- **Type status:** Paratype. **Occurrence:** recordedBy: JA Tenorio; individualCount: 20; lifeStage: adult; **Taxon:** kingdom: Animalia; phylum: Arthropoda; class: Insecta; order: Diptera; family: Canacidae; genus: Procanace; specificEpithet: Procanaceacuminata; scientificNameAuthorship: Hardy & Delfinado, 1980; **Location:** islandGroup: Hawaiian Islands; island: Maui; verbatimLocality: On Stream, Iao Valley; **Identification:** identifiedBy: DE Hardy & MD Delfinado; dateIdentified: 1980; **Event:** verbatimEventDate: 28.iii.1970; **Record Level:** institutionCode: UHM
- **Type status:** Paratype. **Occurrence:** recordedBy: DE Hardy, JA Tenorio; individualCount: 8; sex: 3 males, 5 females; lifeStage: adult; **Taxon:** kingdom: Animalia; phylum: Arthropoda; class: Insecta; order: Diptera; family: Canacidae; genus: Procanace; specificEpithet: Procanaceacuminata; scientificNameAuthorship: Hardy & Delfinado, 1980; **Location:** islandGroup: Hawaiian Islands; island: Maui; verbatimLocality: Iao Valley, on wet rocks in swift moving stream; **Identification:** identifiedBy: DE Hardy & MD Delfinado; dateIdentified: 1980; **Event:** verbatimEventDate: 28.iii.1970; **Record Level:** institutionCode: USNM
- **Type status:** Paratype. **Occurrence:** recordedBy: DE Hardy; individualCount: 3; lifeStage: adult; **Taxon:** kingdom: Animalia; phylum: Arthropoda; class: Insecta; order: Diptera; family: Canacidae; genus: Procanace; specificEpithet: Procanaceacuminata; scientificNameAuthorship: Hardy & Delfinado, 1980; **Location:** islandGroup: Hawaiian Islands; island: Maui; verbatimLocality: Stream in Iao Valley; verbatimElevation: 1375 ft.; **Identification:** identifiedBy: DE Hardy & MD Delfinado; dateIdentified: 1980; **Event:** verbatimEventDate: 31.iii.1970; **Record Level:** institutionCode: UHM
- **Type status:** Paratype. **Occurrence:** recordedBy: DE Hardy; lifeStage: adult; **Taxon:** kingdom: Animalia; phylum: Arthropoda; class: Insecta; order: Diptera; family: Canacidae; genus: Procanace; specificEpithet: Procanaceacuminata; scientificNameAuthorship: Hardy & Delfinado, 1980; **Location:** islandGroup: Hawaiian Islands; island: Maui; verbatimLocality: Makamakaole Valley, on wet rocks along stream; verbatimElevation: 1100 ft.; **Identification:** identifiedBy: DE Hardy & MD Delfinado; dateIdentified: 1980; **Event:** verbatimEventDate: 31.iii.1970
- **Type status:** Paratype. **Occurrence:** recordedBy: JA Tenorio; individualCount: 1; lifeStage: adult; **Taxon:** kingdom: Animalia; phylum: Arthropoda; class: Insecta; order: Diptera; family: Canacidae; genus: Procanace; specificEpithet: Procanaceacuminata; scientificNameAuthorship: Hardy & Delfinado, 1980; **Location:** islandGroup: Hawaiian Islands; island: Hawaii; verbatimLocality: Rainbow Falls Park; **Identification:** identifiedBy: DE Hardy & MD Delfinado; dateIdentified: 1980; **Event:** verbatimEventDate: 28.v.1970; **Record Level:** institutionCode: UHM
- **Type status:** Paratype. **Occurrence:** recordedBy: JA Tenorio; lifeStage: adult; **Taxon:** kingdom: Animalia; phylum: Arthropoda; class: Insecta; order: Diptera; family: Canacidae; genus: Procanace; specificEpithet: Procanaceacuminata; scientificNameAuthorship: Hardy & Delfinado, 1980; **Location:** islandGroup: Hawaiian Islands; island: Hawaii; verbatimLocality: above Rainbow Falls, on rocks in swift stream; **Identification:** identifiedBy: DE Hardy & MD Delfinado; dateIdentified: 1980; **Event:** verbatimEventDate: 28.v.1970
- **Type status:** Paratype. **Occurrence:** recordedBy: MD Delfinado; individualCount: 3; lifeStage: adult; **Taxon:** kingdom: Animalia; phylum: Arthropoda; class: Insecta; order: Diptera; family: Canacidae; genus: Procanace; specificEpithet: Procanaceacuminata; scientificNameAuthorship: Hardy & Delfinado, 1980; **Location:** islandGroup: Hawaiian Islands; island: Hawaii; verbatimLocality: Pahoehoe Stream; **Identification:** identifiedBy: DE Hardy & MD Delfinado; dateIdentified: 1980; **Event:** verbatimEventDate: 29.v.1970; **Record Level:** institutionCode: UHM
- **Type status:** Paratype. **Occurrence:** recordedBy: MD Delfinado; individualCount: 10; lifeStage: adult; **Taxon:** kingdom: Animalia; phylum: Arthropoda; class: Insecta; order: Diptera; family: Canacidae; genus: Procanace; specificEpithet: Procanaceacuminata; scientificNameAuthorship: Hardy & Delfinado, 1980; **Location:** islandGroup: Hawaiian Islands; island: Hawaii; verbatimLocality: Kapue Stream; verbatimElevation: 1000 ft.; **Identification:** identifiedBy: DE Hardy & MD Delfinado; dateIdentified: 1980; **Event:** verbatimEventDate: 29.v.1970; **Record Level:** institutionCode: UHM
- **Type status:** Paratype. **Occurrence:** recordedBy: JA Tenorio; individualCount: 11; lifeStage: adult; **Taxon:** kingdom: Animalia; phylum: Arthropoda; class: Insecta; order: Diptera; family: Canacidae; genus: Procanace; specificEpithet: Procanaceacuminata; scientificNameAuthorship: Hardy & Delfinado, 1980; **Location:** islandGroup: Hawaiian Islands; island: Hawaii; verbatimLocality: Kapue Stream; verbatimElevation: 750 ft.; **Identification:** identifiedBy: DE Hardy & MD Delfinado; dateIdentified: 1980; **Event:** verbatimEventDate: 29.v.1970; **Record Level:** institutionCode: UHM
- **Type status:** Paratype. **Occurrence:** recordedBy: JA Tenorio; individualCount: 11; lifeStage: adult; **Taxon:** kingdom: Animalia; phylum: Arthropoda; class: Insecta; order: Diptera; family: Canacidae; genus: Procanace; specificEpithet: Procanaceacuminata; scientificNameAuthorship: Hardy & Delfinado, 1980; **Location:** islandGroup: Hawaiian Islands; island: Hawaii; verbatimLocality: Pahoehoe Stream; verbatimElevation: 750 ft.; **Identification:** identifiedBy: DE Hardy & MD Delfinado; dateIdentified: 1980; **Event:** verbatimEventDate: 29.v.1970; **Record Level:** institutionCode: UHM
- **Type status:** Paratype. **Occurrence:** recordedBy: MD Delfinado; individualCount: 1; sex: female; lifeStage: adult; **Taxon:** kingdom: Animalia; phylum: Arthropoda; class: Insecta; order: Diptera; family: Canacidae; genus: Procanace; specificEpithet: Procanaceacuminata; scientificNameAuthorship: Hardy & Delfinado, 1980; **Location:** islandGroup: Hawaiian Islands; island: Hawaii; verbatimLocality: Kapue Stream, east slope of Mauna Kea; verbatimElevation: 1000 ft.; **Identification:** identifiedBy: DE Hardy & MD Delfinado; dateIdentified: 1980; **Event:** verbatimEventDate: 29.v.1970; **Record Level:** institutionCode: BPBM
- **Type status:** Paratype. **Occurrence:** recordedBy: MD Delfinado, JA Tenorio; lifeStage: adult; **Taxon:** kingdom: Animalia; phylum: Arthropoda; class: Insecta; order: Diptera; family: Canacidae; genus: Procanace; specificEpithet: Procanaceacuminata; scientificNameAuthorship: Hardy & Delfinado, 1980; **Location:** islandGroup: Hawaiian Islands; island: Hawaii; verbatimLocality: Kapue Stream, east slope of Mauna Kea; verbatimElevation: 1000 ft.; **Identification:** identifiedBy: DE Hardy & MD Delfinado; dateIdentified: 1980; **Event:** verbatimEventDate: 29.v.1970
- **Type status:** Paratype. **Occurrence:** recordedBy: MD Delfinado, JA Tenorio; lifeStage: adult; **Taxon:** kingdom: Animalia; phylum: Arthropoda; class: Insecta; order: Diptera; family: Canacidae; genus: Procanace; specificEpithet: Procanaceacuminata; scientificNameAuthorship: Hardy & Delfinado, 1980; **Location:** islandGroup: Hawaiian Islands; island: Hawaii; verbatimLocality: Pahoehoe, on rocks in swift moving stream; **Identification:** identifiedBy: DE Hardy & MD Delfinado; dateIdentified: 1980; **Event:** verbatimEventDate: 29.v.1970
- **Type status:** Paratype. **Occurrence:** recordedBy: DE Hardy; individualCount: 3; lifeStage: adult; **Taxon:** kingdom: Animalia; phylum: Arthropoda; class: Insecta; order: Diptera; family: Canacidae; genus: Procanace; specificEpithet: Procanaceacuminata; scientificNameAuthorship: Hardy & Delfinado, 1980; **Location:** islandGroup: Hawaiian Islands; island: Maui; verbatimLocality: Iao Stream; verbatimElevation: 800 ft.; **Identification:** identifiedBy: DE Hardy & MD Delfinado; dateIdentified: 1980; **Event:** verbatimEventDate: 2.ix.1970; **Record Level:** institutionCode: UHM
- **Type status:** Other material. **Occurrence:** recordedBy: FX Williams; individualCount: 4; lifeStage: adult; **Taxon:** kingdom: Animalia; phylum: Arthropoda; class: Insecta; order: Diptera; family: Canacidae; genus: Procanace; specificEpithet: Procanaceacuminata; scientificNameAuthorship: Hardy & Delfinado, 1980; **Location:** islandGroup: Hawaiian Islands; island: Molokai; verbatimLocality: Moaula Stream; verbatimElevation: 2000 ft.; **Event:** verbatimEventDate: 28.xi.1933; **Record Level:** institutionCode: UHM
- **Type status:** Other material. **Occurrence:** recordedBy: FX Williams; individualCount: 4; lifeStage: adult; **Taxon:** kingdom: Animalia; phylum: Arthropoda; class: Insecta; order: Diptera; family: Canacidae; genus: Procanace; specificEpithet: Procanaceacuminata; scientificNameAuthorship: Hardy & Delfinado, 1980; **Location:** islandGroup: Hawaiian Islands; island: Molokai; verbatimLocality: Halawa Valley; **Event:** verbatimEventDate: 30.xi.1933; **Record Level:** institutionCode: UHM
- **Type status:** Other material. **Occurrence:** recordedBy: NLH Krauss; individualCount: 1; lifeStage: adult; **Taxon:** kingdom: Animalia; phylum: Arthropoda; class: Insecta; order: Diptera; family: Canacidae; genus: Procanace; specificEpithet: Procanaceacuminata; scientificNameAuthorship: Hardy & Delfinado, 1980; **Location:** islandGroup: Hawaiian Islands; island: Maui; verbatimLocality: Nahiku; **Event:** verbatimEventDate: viii.1952; **Record Level:** institutionCode: UHM
- **Type status:** Other material. **Occurrence:** recordedBy: NLH Krauss; individualCount: 13; lifeStage: adult; **Taxon:** kingdom: Animalia; phylum: Arthropoda; class: Insecta; order: Diptera; family: Canacidae; genus: Procanace; specificEpithet: Procanaceacuminata; scientificNameAuthorship: Hardy & Delfinado, 1980; **Location:** islandGroup: Hawaiian Islands; island: Maui; verbatimLocality: Ulupalakua; **Event:** verbatimEventDate: 8.ii.1958; **Record Level:** institutionCode: UHM
- **Type status:** Other material. **Occurrence:** recordedBy: DE Hardy; individualCount: 72; lifeStage: adult; **Taxon:** kingdom: Animalia; phylum: Arthropoda; class: Insecta; order: Diptera; family: Canacidae; genus: Procanace; specificEpithet: Procanaceacuminata; scientificNameAuthorship: Hardy & Delfinado, 1980; **Location:** islandGroup: Hawaiian Islands; island: Molokai; verbatimLocality: Halawa Valley, Stream below Moalua Falls; **Event:** verbatimEventDate: 16.iii.1970; **Record Level:** institutionCode: UHM
- **Type status:** Other material. **Occurrence:** recordedBy: JA Tenorio; individualCount: 95; lifeStage: adult; **Taxon:** kingdom: Animalia; phylum: Arthropoda; class: Insecta; order: Diptera; family: Canacidae; genus: Procanace; specificEpithet: Procanaceacuminata; scientificNameAuthorship: Hardy & Delfinado, 1980; **Location:** islandGroup: Hawaiian Islands; island: Molokai; verbatimLocality: Waterfalls, Halawa Valley; **Event:** verbatimEventDate: 16.iii.1970; **Record Level:** institutionCode: UHM
- **Type status:** Other material. **Occurrence:** recordedBy: DE Hardy; individualCount: 79; lifeStage: adult; **Taxon:** kingdom: Animalia; phylum: Arthropoda; class: Insecta; order: Diptera; family: Canacidae; genus: Procanace; specificEpithet: Procanaceacuminata; scientificNameAuthorship: Hardy & Delfinado, 1980; **Location:** islandGroup: Hawaiian Islands; island: Molokai; verbatimLocality: Moaula Falls, Halawa Valley; **Event:** verbatimEventDate: 16.iii.1970; **Record Level:** institutionCode: UHM
- **Type status:** Other material. **Occurrence:** recordedBy: DE Hardy; individualCount: 61; lifeStage: adult; **Taxon:** kingdom: Animalia; phylum: Arthropoda; class: Insecta; order: Diptera; family: Canacidae; genus: Procanace; specificEpithet: Procanaceacuminata; scientificNameAuthorship: Hardy & Delfinado, 1980; **Location:** islandGroup: Hawaiian Islands; island: Molokai; verbatimLocality: Halawa Stream; **Event:** verbatimEventDate: 16.iii.1970; **Record Level:** institutionCode: UHM
- **Type status:** Other material. **Occurrence:** recordedBy: JA Tenorio; individualCount: 2; lifeStage: adult; **Taxon:** kingdom: Animalia; phylum: Arthropoda; class: Insecta; order: Diptera; family: Canacidae; genus: Procanace; specificEpithet: Procanaceacuminata; scientificNameAuthorship: Hardy & Delfinado, 1980; **Location:** islandGroup: Hawaiian Islands; island: Molokai; verbatimLocality: Halawa Valley; **Event:** verbatimEventDate: 16.iii.1970; **Record Level:** institutionCode: UHM
- **Type status:** Other material. **Occurrence:** recordedBy: DE Hardy, MD Delfinado, JA Tenorio; lifeStage: adult; **Taxon:** kingdom: Animalia; phylum: Arthropoda; class: Insecta; order: Diptera; family: Canacidae; genus: Procanace; specificEpithet: Procanaceacuminata; scientificNameAuthorship: Hardy & Delfinado, 1980; **Location:** islandGroup: Hawaiian Islands; island: Molokai; verbatimLocality: Moaula Falls, Halawa Valley; **Event:** verbatimEventDate: 16.iii.1970
- **Type status:** Other material. **Occurrence:** catalogNumber: 2006004967; recordedBy: JA Tenorio; lifeStage: adult; **Taxon:** kingdom: Animalia; phylum: Arthropoda; class: Insecta; order: Diptera; family: Canacidae; genus: Procanace; specificEpithet: Procanaceacuminata; scientificNameAuthorship: Hardy & Delfinado, 1980; **Location:** islandGroup: Hawaiian Islands; island: Maui; verbatimLocality: West Maui, Iao Valley, on stream; **Identification:** identifiedBy: DE Hardy & MD Delfinado; dateIdentified: 1980; **Event:** verbatimEventDate: 28.iii.1970; **Record Level:** institutionCode: BPBM
- **Type status:** Other material. **Occurrence:** catalogNumber: 2006004960; recordedBy: JA Tenorio; lifeStage: adult; **Taxon:** kingdom: Animalia; phylum: Arthropoda; class: Insecta; order: Diptera; family: Canacidae; genus: Procanace; specificEpithet: Procanaceacuminata; scientificNameAuthorship: Hardy & Delfinado, 1980; **Location:** islandGroup: Hawaiian Islands; island: Maui; verbatimLocality: West Maui, Iao Valley, on stream; **Identification:** identifiedBy: DE Hardy & MD Delfinado; dateIdentified: 1980; **Event:** verbatimEventDate: 28.iii.1970; **Record Level:** institutionCode: BPBM
- **Type status:** Other material. **Occurrence:** catalogNumber: 2006004962; recordedBy: JA Tenorio; lifeStage: adult; **Taxon:** kingdom: Animalia; phylum: Arthropoda; class: Insecta; order: Diptera; family: Canacidae; genus: Procanace; specificEpithet: Procanaceacuminata; scientificNameAuthorship: Hardy & Delfinado, 1980; **Location:** islandGroup: Hawaiian Islands; island: Maui; verbatimLocality: West Maui, Iao Valley, on stream; **Identification:** identifiedBy: DE Hardy & MD Delfinado; dateIdentified: 1980; **Event:** verbatimEventDate: 28.iii.1970; **Record Level:** institutionCode: BPBM
- **Type status:** Other material. **Occurrence:** catalogNumber: 2006004963; recordedBy: JA Tenorio; lifeStage: adult; **Taxon:** kingdom: Animalia; phylum: Arthropoda; class: Insecta; order: Diptera; family: Canacidae; genus: Procanace; specificEpithet: Procanaceacuminata; scientificNameAuthorship: Hardy & Delfinado, 1980; **Location:** islandGroup: Hawaiian Islands; island: Maui; verbatimLocality: West Maui, Iao Valley, on stream; **Identification:** identifiedBy: DE Hardy & MD Delfinado; dateIdentified: 1980; **Event:** verbatimEventDate: 28.iii.1970; **Record Level:** institutionCode: BPBM
- **Type status:** Other material. **Occurrence:** catalogNumber: 2006004964; recordedBy: JA Tenorio; lifeStage: adult; **Taxon:** kingdom: Animalia; phylum: Arthropoda; class: Insecta; order: Diptera; family: Canacidae; genus: Procanace; specificEpithet: Procanaceacuminata; scientificNameAuthorship: Hardy & Delfinado, 1980; **Location:** islandGroup: Hawaiian Islands; island: Maui; verbatimLocality: West Maui, Iao Valley, on stream; **Identification:** identifiedBy: DE Hardy & MD Delfinado; dateIdentified: 1980; **Event:** verbatimEventDate: 28.iii.1970; **Record Level:** institutionCode: BPBM
- **Type status:** Other material. **Occurrence:** catalogNumber: 2006004965; recordedBy: JA Tenorio; lifeStage: adult; **Taxon:** kingdom: Animalia; phylum: Arthropoda; class: Insecta; order: Diptera; family: Canacidae; genus: Procanace; specificEpithet: Procanaceacuminata; scientificNameAuthorship: Hardy & Delfinado, 1980; **Location:** islandGroup: Hawaiian Islands; island: Maui; verbatimLocality: West Maui, Iao Valley, on stream; **Identification:** identifiedBy: DE Hardy & MD Delfinado; dateIdentified: 1980; **Event:** verbatimEventDate: 28.iii.1970; **Record Level:** institutionCode: BPBM
- **Type status:** Other material. **Occurrence:** catalogNumber: 2006004966; recordedBy: JA Tenorio; lifeStage: adult; **Taxon:** kingdom: Animalia; phylum: Arthropoda; class: Insecta; order: Diptera; family: Canacidae; genus: Procanace; specificEpithet: Procanaceacuminata; scientificNameAuthorship: Hardy & Delfinado, 1980; **Location:** islandGroup: Hawaiian Islands; island: Maui; verbatimLocality: West Maui, Iao Valley, on stream; **Identification:** identifiedBy: DE Hardy & MD Delfinado; dateIdentified: 1980; **Event:** verbatimEventDate: 28.iii.1970; **Record Level:** institutionCode: BPBM
- **Type status:** Other material. **Occurrence:** catalogNumber: 2006004961; recordedBy: JA Tenorio; lifeStage: adult; **Taxon:** kingdom: Animalia; phylum: Arthropoda; class: Insecta; order: Diptera; family: Canacidae; genus: Procanace; specificEpithet: Procanaceacuminata; scientificNameAuthorship: Hardy & Delfinado, 1980; **Location:** islandGroup: Hawaiian Islands; island: Maui; verbatimLocality: West Maui, Iao Valley, on stream; **Identification:** identifiedBy: DE Hardy & MD Delfinado; dateIdentified: 1980; **Event:** verbatimEventDate: 28.iii.1970; **Record Level:** institutionCode: BPBM
- **Type status:** Other material. **Occurrence:** catalogNumber: 2006004959; recordedBy: JA Tenorio; lifeStage: adult; **Taxon:** kingdom: Animalia; phylum: Arthropoda; class: Insecta; order: Diptera; family: Canacidae; genus: Procanace; specificEpithet: Procanaceacuminata; scientificNameAuthorship: Hardy & Delfinado, 1980; **Location:** islandGroup: Hawaiian Islands; island: Maui; verbatimLocality: West Maui, Iao Valley, on stream; **Identification:** identifiedBy: DE Hardy & MD Delfinado; dateIdentified: 1980; **Event:** verbatimEventDate: 28.iii.1970; **Record Level:** institutionCode: BPBM
- **Type status:** Other material. **Occurrence:** catalogNumber: 2006004952; recordedBy: JA Tenorio; lifeStage: adult; **Taxon:** kingdom: Animalia; phylum: Arthropoda; class: Insecta; order: Diptera; family: Canacidae; genus: Procanace; specificEpithet: Procanaceacuminata; scientificNameAuthorship: Hardy & Delfinado, 1980; **Location:** islandGroup: Hawaiian Islands; island: Maui; verbatimLocality: West Maui, Iao Valley, on stream; **Identification:** identifiedBy: DE Hardy & MD Delfinado; dateIdentified: 1980; **Event:** verbatimEventDate: 28.iii.1970; **Record Level:** institutionCode: BPBM
- **Type status:** Other material. **Occurrence:** catalogNumber: 2006004953; recordedBy: JA Tenorio; lifeStage: adult; **Taxon:** kingdom: Animalia; phylum: Arthropoda; class: Insecta; order: Diptera; family: Canacidae; genus: Procanace; specificEpithet: Procanaceacuminata; scientificNameAuthorship: Hardy & Delfinado, 1980; **Location:** islandGroup: Hawaiian Islands; island: Maui; verbatimLocality: West Maui, Iao Valley, on stream; **Identification:** identifiedBy: DE Hardy & MD Delfinado; dateIdentified: 1980; **Event:** verbatimEventDate: 28.iii.1970; **Record Level:** institutionCode: BPBM
- **Type status:** Other material. **Occurrence:** catalogNumber: 2006004968; recordedBy: DE Hardy; lifeStage: adult; **Taxon:** kingdom: Animalia; phylum: Arthropoda; class: Insecta; order: Diptera; family: Canacidae; genus: Procanace; specificEpithet: Procanaceacuminata; scientificNameAuthorship: Hardy & Delfinado, 1980; **Location:** islandGroup: Hawaiian Islands; island: Maui; verbatimLocality: Makamakaole Valley, along stream; minimumElevationInMeters: 1100; **Identification:** identifiedBy: DE Hardy & MD Delfinado; dateIdentified: 1980; **Event:** verbatimEventDate: 31.iii.1970; **Record Level:** institutionCode: BPBM
- **Type status:** Other material. **Occurrence:** recordedBy: MD Delfinado; individualCount: 1; lifeStage: adult; **Taxon:** kingdom: Animalia; phylum: Arthropoda; class: Insecta; order: Diptera; family: Canacidae; genus: Procanace; specificEpithet: Procanaceacuminata; scientificNameAuthorship: Hardy & Delfinado, 1980; **Location:** islandGroup: Hawaiian Islands; island: Hawaii; verbatimLocality: Wailuku Stream, above Rainbow Falls; **Event:** verbatimEventDate: 28.v.1970; **Record Level:** institutionCode: UHM
- **Type status:** Other material. **Occurrence:** recordedBy: MD Delfinado; individualCount: 3; lifeStage: adult; **Taxon:** kingdom: Animalia; phylum: Arthropoda; class: Insecta; order: Diptera; family: Canacidae; genus: Procanace; specificEpithet: Procanaceacuminata; scientificNameAuthorship: Hardy & Delfinado, 1980; **Location:** islandGroup: Hawaiian Islands; island: Hawaii; verbatimLocality: Akaka Park Stream; **Event:** verbatimEventDate: 29.v.1970; **Record Level:** institutionCode: UHM
- **Type status:** Other material. **Occurrence:** recordedBy: JA Tenorio; individualCount: 3; lifeStage: adult; **Taxon:** kingdom: Animalia; phylum: Arthropoda; class: Insecta; order: Diptera; family: Canacidae; genus: Procanace; specificEpithet: Procanaceacuminata; scientificNameAuthorship: Hardy & Delfinado, 1980; **Location:** islandGroup: Hawaiian Islands; island: Hawaii; verbatimLocality: Kaieie Stream; verbatimElevation: 1000 ft.; **Event:** verbatimEventDate: 29.v.1970; **Record Level:** institutionCode: UHM
- **Type status:** Other material. **Occurrence:** recordedBy: MD Delfinado; individualCount: 5; lifeStage: adult; **Taxon:** kingdom: Animalia; phylum: Arthropoda; class: Insecta; order: Diptera; family: Canacidae; genus: Procanace; specificEpithet: Procanaceacuminata; scientificNameAuthorship: Hardy & Delfinado, 1980; **Location:** islandGroup: Hawaiian Islands; island: Hawaii; verbatimLocality: Kaieie Stream; verbatimElevation: 1000 ft.; **Event:** verbatimEventDate: 29.v.1970; **Record Level:** institutionCode: UHM
- **Type status:** Other material. **Occurrence:** recordedBy: M Tamashiro; individualCount: 1; lifeStage: adult; **Taxon:** kingdom: Animalia; phylum: Arthropoda; class: Insecta; order: Diptera; family: Canacidae; genus: Procanace; specificEpithet: Procanaceacuminata; scientificNameAuthorship: Hardy & Delfinado, 1980; **Location:** islandGroup: Hawaiian Islands; island: Hawaii; verbatimLocality: Pahoehoe Stream; verbatimElevation: 1200 ft.; **Event:** verbatimEventDate: 29.v.1970; **Record Level:** institutionCode: UHM
- **Type status:** Other material. **Occurrence:** catalogNumber: 2006004954; recordedBy: JA Tenorio; lifeStage: adult; **Taxon:** kingdom: Animalia; phylum: Arthropoda; class: Insecta; order: Diptera; family: Canacidae; genus: Procanace; specificEpithet: Procanaceacuminata; scientificNameAuthorship: Hardy & Delfinado, 1980; **Location:** islandGroup: Hawaiian Islands; island: Hawaii; verbatimLocality: Kapue Stream; minimumElevationInMeters: 750; **Identification:** identifiedBy: DE Hardy & MD Delfinado; dateIdentified: 1980; **Event:** verbatimEventDate: 29.v.1970; **Record Level:** institutionCode: BPBM
- **Type status:** Other material. **Occurrence:** catalogNumber: 2006004957; recordedBy: JA Tenorio; lifeStage: adult; **Taxon:** kingdom: Animalia; phylum: Arthropoda; class: Insecta; order: Diptera; family: Canacidae; genus: Procanace; specificEpithet: Procanaceacuminata; scientificNameAuthorship: Hardy & Delfinado, 1980; **Location:** islandGroup: Hawaiian Islands; island: Hawaii; verbatimLocality: Kapue Stream; minimumElevationInMeters: 750; **Identification:** identifiedBy: DE Hardy & MD Delfinado; dateIdentified: 1980; **Event:** verbatimEventDate: 29.v.1970; **Record Level:** institutionCode: BPBM
- **Type status:** Other material. **Occurrence:** catalogNumber: 2006004955; recordedBy: JA Tenorio; lifeStage: adult; **Taxon:** kingdom: Animalia; phylum: Arthropoda; class: Insecta; order: Diptera; family: Canacidae; genus: Procanace; specificEpithet: Procanaceacuminata; scientificNameAuthorship: Hardy & Delfinado, 1980; **Location:** islandGroup: Hawaiian Islands; island: Hawaii; verbatimLocality: Kapue Stream; minimumElevationInMeters: 750; **Identification:** identifiedBy: DE Hardy & MD Delfinado; dateIdentified: 1980; **Event:** verbatimEventDate: 29.v.1970; **Record Level:** institutionCode: BPBM
- **Type status:** Other material. **Occurrence:** catalogNumber: 2006004956; recordedBy: JA Tenorio; lifeStage: adult; **Taxon:** kingdom: Animalia; phylum: Arthropoda; class: Insecta; order: Diptera; family: Canacidae; genus: Procanace; specificEpithet: Procanaceacuminata; scientificNameAuthorship: Hardy & Delfinado, 1980; **Location:** islandGroup: Hawaiian Islands; island: Hawaii; verbatimLocality: Kapue Stream; minimumElevationInMeters: 750; **Identification:** identifiedBy: DE Hardy & MD Delfinado; dateIdentified: 1980; **Event:** verbatimEventDate: 29.v.1970; **Record Level:** institutionCode: BPBM
- **Type status:** Other material. **Occurrence:** catalogNumber: 2006004958; recordedBy: MD Delfinado; lifeStage: adult; **Taxon:** kingdom: Animalia; phylum: Arthropoda; class: Insecta; order: Diptera; family: Canacidae; genus: Procanace; specificEpithet: Procanaceacuminata; scientificNameAuthorship: Hardy & Delfinado, 1980; **Location:** islandGroup: Hawaiian Islands; island: Hawaii; verbatimLocality: Kapue Stream; minimumElevationInMeters: 1000; **Identification:** identifiedBy: DE Hardy & MD Delfinado; dateIdentified: 1980; **Event:** verbatimEventDate: 29.v.1970; **Record Level:** institutionCode: BPBM
- **Type status:** Other material. **Occurrence:** recordedBy: J Kjargaard; individualCount: 5; lifeStage: adult; **Taxon:** kingdom: Animalia; phylum: Arthropoda; class: Insecta; order: Diptera; family: Canacidae; genus: Procanace; specificEpithet: Procanaceacuminata; scientificNameAuthorship: Hardy & Delfinado, 1980; **Location:** islandGroup: Hawaiian Islands; island: Molokai; verbatimLocality: Pulena Stream; verbatimElevation: 650 ft.; **Event:** verbatimEventDate: 3.vi.1970; **Record Level:** institutionCode: UHM
- **Type status:** Other material. **Occurrence:** recordedBy: J Kjargaard; individualCount: 1; lifeStage: adult; **Taxon:** kingdom: Animalia; phylum: Arthropoda; class: Insecta; order: Diptera; family: Canacidae; genus: Procanace; specificEpithet: Procanaceacuminata; scientificNameAuthorship: Hardy & Delfinado, 1980; **Location:** islandGroup: Hawaiian Islands; island: Molokai; verbatimLocality: 0.5 miles above Wailau Stream gauge; verbatimElevation: 700 ft.; **Event:** verbatimEventDate: 3.vi.1970; **Record Level:** institutionCode: UHM
- **Type status:** Other material. **Occurrence:** recordedBy: J Kjargaard; individualCount: 1; lifeStage: adult; **Taxon:** kingdom: Animalia; phylum: Arthropoda; class: Insecta; order: Diptera; family: Canacidae; genus: Procanace; specificEpithet: Procanaceacuminata; scientificNameAuthorship: Hardy & Delfinado, 1980; **Location:** islandGroup: Hawaiian Islands; island: Molokai; verbatimLocality: 0.25 miles below Wailau Stream gauge; verbatimElevation: 550 ft.; **Event:** verbatimEventDate: 4.vi.1970; **Record Level:** institutionCode: UHM
- **Type status:** Other material. **Occurrence:** recordedBy: WC Gagne; individualCount: 1; lifeStage: adult; **Taxon:** kingdom: Animalia; phylum: Arthropoda; class: Insecta; order: Diptera; family: Canacidae; genus: Procanace; specificEpithet: Procanaceacuminata; scientificNameAuthorship: Hardy & Delfinado, 1980; **Location:** islandGroup: Hawaiian Islands; island: Hawaii; verbatimLocality: Kohala Mountains, Honopue Stream; verbatimElevation: 2000 ft.; **Event:** verbatimEventDate: 12.vi.1970; **Record Level:** institutionCode: UHM
- **Type status:** Other material. **Occurrence:** recordedBy: MD Delfinado; individualCount: 4; lifeStage: adult; **Taxon:** kingdom: Animalia; phylum: Arthropoda; class: Insecta; order: Diptera; family: Canacidae; genus: Procanace; specificEpithet: Procanaceacuminata; scientificNameAuthorship: Hardy & Delfinado, 1980; **Location:** islandGroup: Hawaiian Islands; island: Maui; verbatimLocality: Seven Sacred Pools; **Event:** verbatimEventDate: 3.viii.1970; **Record Level:** institutionCode: UHM
- **Type status:** Other material. **Occurrence:** recordedBy: JA Tenorio; individualCount: 8; lifeStage: adult; **Taxon:** kingdom: Animalia; phylum: Arthropoda; class: Insecta; order: Diptera; family: Canacidae; genus: Procanace; specificEpithet: Procanaceacuminata; scientificNameAuthorship: Hardy & Delfinado, 1980; **Location:** islandGroup: Hawaiian Islands; island: Hawaii; verbatimLocality: Wailuku River; verbatimElevation: 880 ft.; **Event:** verbatimEventDate: 14.viii.1970; **Record Level:** institutionCode: UHM
- **Type status:** Other material. **Occurrence:** recordedBy: DE Hardy; individualCount: 80; lifeStage: adult; **Taxon:** kingdom: Animalia; phylum: Arthropoda; class: Insecta; order: Diptera; family: Canacidae; genus: Procanace; specificEpithet: Procanaceacuminata; scientificNameAuthorship: Hardy & Delfinado, 1980; **Location:** islandGroup: Hawaiian Islands; island: Maui; verbatimLocality: Kinihapai Stream, Iao Valley; verbatimElevation: 800 ft.; **Event:** verbatimEventDate: 2.ix.1970; **Record Level:** institutionCode: UHM
- **Type status:** Other material. **Occurrence:** recordedBy: DE Hardy; individualCount: 56; lifeStage: adult; **Taxon:** kingdom: Animalia; phylum: Arthropoda; class: Insecta; order: Diptera; family: Canacidae; genus: Procanace; specificEpithet: Procanaceacuminata; scientificNameAuthorship: Hardy & Delfinado, 1980; **Location:** islandGroup: Hawaiian Islands; island: Maui; verbatimLocality: Iao Stream, Iao Valley; verbatimElevation: 800 ft.; **Event:** verbatimEventDate: 2.ix.1970; **Record Level:** institutionCode: UHM
- **Type status:** Other material. **Occurrence:** recordedBy: DE Hardy; individualCount: 8; lifeStage: adult; **Taxon:** kingdom: Animalia; phylum: Arthropoda; class: Insecta; order: Diptera; family: Canacidae; genus: Procanace; specificEpithet: Procanaceacuminata; scientificNameAuthorship: Hardy & Delfinado, 1980; **Location:** islandGroup: Hawaiian Islands; island: Maui; verbatimLocality: Kopiliula Stream, Hana; verbatimElevation: 1200 ft.; **Event:** verbatimEventDate: 4.ix.1970; **Record Level:** institutionCode: UHM
- **Type status:** Other material. **Occurrence:** recordedBy: DE Hardy; individualCount: 30; lifeStage: adult; **Taxon:** kingdom: Animalia; phylum: Arthropoda; class: Insecta; order: Diptera; family: Canacidae; genus: Procanace; specificEpithet: Procanaceacuminata; scientificNameAuthorship: Hardy & Delfinado, 1980; **Location:** islandGroup: Hawaiian Islands; island: Maui; verbatimLocality: Waikane Stream; verbatimElevation: 600 ft.; **Event:** verbatimEventDate: 4.ix.1970; **Record Level:** institutionCode: UHM
- **Type status:** Other material. **Occurrence:** recordedBy: DE Hardy; individualCount: 1; lifeStage: adult; **Taxon:** kingdom: Animalia; phylum: Arthropoda; class: Insecta; order: Diptera; family: Canacidae; genus: Procanace; specificEpithet: Procanaceacuminata; scientificNameAuthorship: Hardy & Delfinado, 1980; **Location:** islandGroup: Hawaiian Islands; island: Maui; verbatimLocality: Kinihapai Stream, Iao Valley; verbatimElevation: 300 ft.; **Event:** verbatimEventDate: 2.x.1970; **Record Level:** institutionCode: UHM
- **Type status:** Other material. **Occurrence:** catalogNumber: 2006005037; recordedBy: DA Polhemus; lifeStage: adult; **Taxon:** kingdom: Animalia; phylum: Arthropoda; class: Insecta; order: Diptera; family: Canacidae; genus: Procanace; specificEpithet: Procanaceacuminata; scientificNameAuthorship: Hardy & Delfinado, 1980; **Location:** islandGroup: Hawaiian Islands; island: Hawaii; verbatimLocality: Hilo, NW of Honolii Stream; minimumElevationInMeters: 1312; **Identification:** identifiedBy: WN Mathis; dateIdentified: 1992; **Event:** verbatimEventDate: 26.vii.1990; **Record Level:** institutionCode: BPBM
- **Type status:** Other material. **Occurrence:** catalogNumber: 2006005039; recordedBy: DA Polhemus; individualCount: 1; sex: 1 male; lifeStage: adult; **Taxon:** kingdom: Animalia; phylum: Arthropoda; class: Insecta; order: Diptera; family: Canacidae; genus: Procanace; specificEpithet: Procanaceacuminata; scientificNameAuthorship: Hardy & Delfinado, 1980; **Location:** islandGroup: Hawaiian Islands; island: Hawaii; verbatimLocality: Hilo, NW of Honolii Stream; verbatimElevation: 400; minimumElevationInMeters: 400; **Identification:** identifiedBy: WN Mathis; dateIdentified: 1992; **Event:** eventDate: 26.vii.1990; verbatimEventDate: 26.x.1990; **Record Level:** institutionCode: USNM
- **Type status:** Other material. **Occurrence:** catalogNumber: 2006005038; recordedBy: DA Polhemus; lifeStage: adult; **Taxon:** kingdom: Animalia; phylum: Arthropoda; class: Insecta; order: Diptera; family: Canacidae; genus: Procanace; specificEpithet: Procanaceacuminata; scientificNameAuthorship: Hardy & Delfinado, 1980; **Location:** islandGroup: Hawaiian Islands; island: Hawaii; verbatimLocality: Hilo, NW of Honolii Stream; minimumElevationInMeters: 1312; **Identification:** identifiedBy: WN Mathis; dateIdentified: 1992; **Event:** verbatimEventDate: 26.x.1990; **Record Level:** institutionCode: BPBM
- **Type status:** Other material. **Occurrence:** recordedBy: DA Polhemus; individualCount: 5; sex: 3 males, 2 females; lifeStage: adult; **Taxon:** kingdom: Animalia; phylum: Arthropoda; class: Insecta; order: Diptera; family: Canacidae; genus: Procanace; specificEpithet: Procanaceacuminata; scientificNameAuthorship: Hardy & Delfinado, 1980; **Location:** islandGroup: Hawaiian Islands; island: Hawaii; verbatimLocality: Hilo, NW of Honolii Stream; minimumElevationInMeters: 400; **Identification:** identifiedBy: WN Mathis; dateIdentified: 1992; **Event:** verbatimEventDate: 26.x.1990; **Record Level:** institutionCode: USNM
- **Type status:** Other material. **Occurrence:** catalogNumber: 2006005039; recordedBy: DA Polhemus; lifeStage: adult; **Taxon:** kingdom: Animalia; phylum: Arthropoda; class: Insecta; order: Diptera; family: Canacidae; genus: Procanace; specificEpithet: Procanaceacuminata; scientificNameAuthorship: Hardy & Delfinado, 1980; **Location:** islandGroup: Hawaiian Islands; island: Hawaii; verbatimLocality: Hilo, NW of Honolii Stream; minimumElevationInMeters: 1312; **Identification:** identifiedBy: WN Mathis; dateIdentified: 1992; **Event:** verbatimEventDate: 26.x.1990; **Record Level:** institutionCode: BPBM
- **Type status:** Other material. **Occurrence:** catalogNumber: 2006004999; recordedBy: DA Polhemus; lifeStage: adult; **Taxon:** kingdom: Animalia; phylum: Arthropoda; class: Insecta; order: Diptera; family: Canacidae; genus: Procanace; specificEpithet: Procanaceacuminata; scientificNameAuthorship: Hardy & Delfinado, 1980; **Location:** islandGroup: Hawaiian Islands; island: Molokai; verbatimLocality: Pelekunu TNCH Preserve, Lower Pelekunu Valley; minimumElevationInMeters: 0; maximumElevationInMeters: 98; **Identification:** identifiedBy: WN Mathis; dateIdentified: 1992; **Event:** verbatimEventDate: 19.viii.1991; **Record Level:** institutionCode: BPBM
- **Type status:** Other material. **Occurrence:** catalogNumber: 2006004975; recordedBy: DA Polhemus; lifeStage: adult; **Taxon:** kingdom: Animalia; phylum: Arthropoda; class: Insecta; order: Diptera; family: Canacidae; genus: Procanace; specificEpithet: Procanaceacuminata; scientificNameAuthorship: Hardy & Delfinado, 1980; **Location:** islandGroup: Hawaiian Islands; island: Molokai; verbatimLocality: Pelekunu TNCH Preserve, Lower Pelekunu Valley; minimumElevationInMeters: 0; maximumElevationInMeters: 98; **Identification:** identifiedBy: WN Mathis; dateIdentified: 1992; **Event:** verbatimEventDate: 19.viii.1991; **Record Level:** institutionCode: BPBM
- **Type status:** Other material. **Occurrence:** catalogNumber: 2006004993; recordedBy: DA Polhemus; lifeStage: adult; **Taxon:** kingdom: Animalia; phylum: Arthropoda; class: Insecta; order: Diptera; family: Canacidae; genus: Procanace; specificEpithet: Procanaceacuminata; scientificNameAuthorship: Hardy & Delfinado, 1980; **Location:** islandGroup: Hawaiian Islands; island: Molokai; verbatimLocality: Pelekunu TNCH Preserve, Lower Pelekunu Valley; minimumElevationInMeters: 0; maximumElevationInMeters: 98; **Identification:** identifiedBy: WN Mathis; dateIdentified: 1992; **Event:** verbatimEventDate: 19.viii.1991; **Record Level:** institutionCode: BPBM
- **Type status:** Other material. **Occurrence:** catalogNumber: 2006004994; recordedBy: DA Polhemus; lifeStage: adult; **Taxon:** kingdom: Animalia; phylum: Arthropoda; class: Insecta; order: Diptera; family: Canacidae; genus: Procanace; specificEpithet: Procanaceacuminata; scientificNameAuthorship: Hardy & Delfinado, 1980; **Location:** islandGroup: Hawaiian Islands; island: Molokai; verbatimLocality: Pelekunu TNCH Preserve, Lower Pelekunu Valley; minimumElevationInMeters: 0; maximumElevationInMeters: 98; **Identification:** identifiedBy: WN Mathis; dateIdentified: 1992; **Event:** verbatimEventDate: 19.viii.1991; **Record Level:** institutionCode: BPBM
- **Type status:** Other material. **Occurrence:** catalogNumber: 2006004995; recordedBy: DA Polhemus; lifeStage: adult; **Taxon:** kingdom: Animalia; phylum: Arthropoda; class: Insecta; order: Diptera; family: Canacidae; genus: Procanace; specificEpithet: Procanaceacuminata; scientificNameAuthorship: Hardy & Delfinado, 1980; **Location:** islandGroup: Hawaiian Islands; island: Molokai; verbatimLocality: Pelekunu TNCH Preserve, Lower Pelekunu Valley; minimumElevationInMeters: 0; maximumElevationInMeters: 98; **Identification:** identifiedBy: WN Mathis; dateIdentified: 1992; **Event:** verbatimEventDate: 19.viii.1991; **Record Level:** institutionCode: BPBM
- **Type status:** Other material. **Occurrence:** catalogNumber: 2006004996; recordedBy: DA Polhemus; lifeStage: adult; **Taxon:** kingdom: Animalia; phylum: Arthropoda; class: Insecta; order: Diptera; family: Canacidae; genus: Procanace; specificEpithet: Procanaceacuminata; scientificNameAuthorship: Hardy & Delfinado, 1980; **Location:** islandGroup: Hawaiian Islands; island: Molokai; verbatimLocality: Pelekunu TNCH Preserve, Lower Pelekunu Valley; minimumElevationInMeters: 0; maximumElevationInMeters: 98; **Identification:** identifiedBy: WN Mathis; dateIdentified: 1992; **Event:** verbatimEventDate: 19.viii.1991; **Record Level:** institutionCode: BPBM
- **Type status:** Other material. **Occurrence:** catalogNumber: 2006004991; recordedBy: DA Polhemus; lifeStage: adult; **Taxon:** kingdom: Animalia; phylum: Arthropoda; class: Insecta; order: Diptera; family: Canacidae; genus: Procanace; specificEpithet: Procanaceacuminata; scientificNameAuthorship: Hardy & Delfinado, 1980; **Location:** islandGroup: Hawaiian Islands; island: Molokai; verbatimLocality: Pelekunu TNCH Preserve, Lower Pelekunu Valley; minimumElevationInMeters: 0; maximumElevationInMeters: 98; **Identification:** identifiedBy: WN Mathis; dateIdentified: 1992; **Event:** verbatimEventDate: 19.viii.1991; **Record Level:** institutionCode: BPBM
- **Type status:** Other material. **Occurrence:** catalogNumber: 2006004998; recordedBy: DA Polhemus; lifeStage: adult; **Taxon:** kingdom: Animalia; phylum: Arthropoda; class: Insecta; order: Diptera; family: Canacidae; genus: Procanace; specificEpithet: Procanaceacuminata; scientificNameAuthorship: Hardy & Delfinado, 1980; **Location:** islandGroup: Hawaiian Islands; island: Molokai; verbatimLocality: Pelekunu TNCH Preserve, Lower Pelekunu Valley; minimumElevationInMeters: 0; maximumElevationInMeters: 98; **Identification:** identifiedBy: WN Mathis; dateIdentified: 1992; **Event:** verbatimEventDate: 19.viii.1991; **Record Level:** institutionCode: BPBM
- **Type status:** Other material. **Occurrence:** catalogNumber: 2006004990; recordedBy: DA Polhemus; lifeStage: adult; **Taxon:** kingdom: Animalia; phylum: Arthropoda; class: Insecta; order: Diptera; family: Canacidae; genus: Procanace; specificEpithet: Procanaceacuminata; scientificNameAuthorship: Hardy & Delfinado, 1980; **Location:** islandGroup: Hawaiian Islands; island: Molokai; verbatimLocality: Pelekunu TNCH Preserve, Lower Pelekunu Valley; minimumElevationInMeters: 0; maximumElevationInMeters: 98; **Identification:** identifiedBy: WN Mathis; dateIdentified: 1992; **Event:** verbatimEventDate: 19.viii.1991; **Record Level:** institutionCode: BPBM
- **Type status:** Other material. **Occurrence:** catalogNumber: 2006005000; recordedBy: DA Polhemus; lifeStage: adult; **Taxon:** kingdom: Animalia; phylum: Arthropoda; class: Insecta; order: Diptera; family: Canacidae; genus: Procanace; specificEpithet: Procanaceacuminata; scientificNameAuthorship: Hardy & Delfinado, 1980; **Location:** islandGroup: Hawaiian Islands; island: Molokai; verbatimLocality: Pelekunu TNCH Preserve, Lower Pelekunu Valley; minimumElevationInMeters: 0; maximumElevationInMeters: 98; **Identification:** identifiedBy: WN Mathis; dateIdentified: 1992; **Event:** verbatimEventDate: 19.viii.1991; **Record Level:** institutionCode: BPBM
- **Type status:** Other material. **Occurrence:** catalogNumber: 2006005001; recordedBy: DA Polhemus; lifeStage: adult; **Taxon:** kingdom: Animalia; phylum: Arthropoda; class: Insecta; order: Diptera; family: Canacidae; genus: Procanace; specificEpithet: Procanaceacuminata; scientificNameAuthorship: Hardy & Delfinado, 1980; **Location:** islandGroup: Hawaiian Islands; island: Molokai; verbatimLocality: Pelekunu TNCH Preserve, Lower Pelekunu Valley; minimumElevationInMeters: 0; maximumElevationInMeters: 98; **Identification:** identifiedBy: WN Mathis; dateIdentified: 1992; **Event:** verbatimEventDate: 19.viii.1991; **Record Level:** institutionCode: BPBM
- **Type status:** Other material. **Occurrence:** catalogNumber: 2006005002; recordedBy: DA Polhemus; lifeStage: adult; **Taxon:** kingdom: Animalia; phylum: Arthropoda; class: Insecta; order: Diptera; family: Canacidae; genus: Procanace; specificEpithet: Procanaceacuminata; scientificNameAuthorship: Hardy & Delfinado, 1980; **Location:** islandGroup: Hawaiian Islands; island: Molokai; verbatimLocality: Pelekunu TNCH Preserve, Lower Pelekunu Valley; minimumElevationInMeters: 0; maximumElevationInMeters: 98; **Identification:** identifiedBy: WN Mathis; dateIdentified: 1992; **Event:** verbatimEventDate: 19.viii.1991; **Record Level:** institutionCode: BPBM
- **Type status:** Other material. **Occurrence:** catalogNumber: 2006005003; recordedBy: DA Polhemus; lifeStage: adult; **Taxon:** kingdom: Animalia; phylum: Arthropoda; class: Insecta; order: Diptera; family: Canacidae; genus: Procanace; specificEpithet: Procanaceacuminata; scientificNameAuthorship: Hardy & Delfinado, 1980; **Location:** islandGroup: Hawaiian Islands; island: Molokai; verbatimLocality: Pelekunu TNCH Preserve, Lower Pelekunu Valley; minimumElevationInMeters: 0; maximumElevationInMeters: 100; **Identification:** identifiedBy: WN Mathis; dateIdentified: 1992; **Event:** verbatimEventDate: 19.viii.1991; **Record Level:** institutionCode: BPBM
- **Type status:** Other material. **Occurrence:** catalogNumber: 2006005004; recordedBy: DA Polhemus; lifeStage: adult; **Taxon:** kingdom: Animalia; phylum: Arthropoda; class: Insecta; order: Diptera; family: Canacidae; genus: Procanace; specificEpithet: Procanaceacuminata; scientificNameAuthorship: Hardy & Delfinado, 1980; **Location:** islandGroup: Hawaiian Islands; island: Molokai; verbatimLocality: Pelekunu TNCH Preserve, Lower Pelekunu Valley; minimumElevationInMeters: 0; maximumElevationInMeters: 100; **Identification:** identifiedBy: WN Mathis; dateIdentified: 1992; **Event:** verbatimEventDate: 19.viii.1991; **Record Level:** institutionCode: BPBM
- **Type status:** Other material. **Occurrence:** catalogNumber: 2006005005; recordedBy: DA Polhemus; lifeStage: adult; **Taxon:** kingdom: Animalia; phylum: Arthropoda; class: Insecta; order: Diptera; family: Canacidae; genus: Procanace; specificEpithet: Procanaceacuminata; scientificNameAuthorship: Hardy & Delfinado, 1980; **Location:** islandGroup: Hawaiian Islands; island: Molokai; verbatimLocality: Pelekunu TNCH Preserve, Lower Pelekunu Valley; minimumElevationInMeters: 0; maximumElevationInMeters: 100; **Identification:** identifiedBy: WN Mathis; dateIdentified: 1992; **Event:** verbatimEventDate: 19.viii.1991; **Record Level:** institutionCode: BPBM
- **Type status:** Other material. **Occurrence:** catalogNumber: 2006004997; recordedBy: DA Polhemus; lifeStage: adult; **Taxon:** kingdom: Animalia; phylum: Arthropoda; class: Insecta; order: Diptera; family: Canacidae; genus: Procanace; specificEpithet: Procanaceacuminata; scientificNameAuthorship: Hardy & Delfinado, 1980; **Location:** islandGroup: Hawaiian Islands; island: Molokai; verbatimLocality: Pelekunu TNCH Preserve, Lower Pelekunu Valley; minimumElevationInMeters: 0; maximumElevationInMeters: 98; **Identification:** identifiedBy: WN Mathis; dateIdentified: 1992; **Event:** verbatimEventDate: 19.viii.1991; **Record Level:** institutionCode: BPBM
- **Type status:** Other material. **Occurrence:** catalogNumber: 2006004977; recordedBy: DA Polhemus; lifeStage: adult; **Taxon:** kingdom: Animalia; phylum: Arthropoda; class: Insecta; order: Diptera; family: Canacidae; genus: Procanace; specificEpithet: Procanaceacuminata; scientificNameAuthorship: Hardy & Delfinado, 1980; **Location:** islandGroup: Hawaiian Islands; island: Molokai; verbatimLocality: Pelekunu TNCH Preserve, Lower Pelekunu Valley; minimumElevationInMeters: 0; maximumElevationInMeters: 98; **Identification:** identifiedBy: WN Mathis; dateIdentified: 1992; **Event:** verbatimEventDate: 19.viii.1991; **Record Level:** institutionCode: BPBM
- **Type status:** Other material. **Occurrence:** catalogNumber: 2006004983; recordedBy: DA Polhemus; lifeStage: adult; **Taxon:** kingdom: Animalia; phylum: Arthropoda; class: Insecta; order: Diptera; family: Canacidae; genus: Procanace; specificEpithet: Procanaceacuminata; scientificNameAuthorship: Hardy & Delfinado, 1980; **Location:** islandGroup: Hawaiian Islands; island: Molokai; verbatimLocality: Pelekunu TNCH Preserve, Lower Pelekunu Valley; minimumElevationInMeters: 0; maximumElevationInMeters: 98; **Identification:** identifiedBy: WN Mathis; dateIdentified: 1992; **Event:** verbatimEventDate: 19.viii.1991; **Record Level:** institutionCode: BPBM
- **Type status:** Other material. **Occurrence:** catalogNumber: 2006004978; recordedBy: DA Polhemus; lifeStage: adult; **Taxon:** kingdom: Animalia; phylum: Arthropoda; class: Insecta; order: Diptera; family: Canacidae; genus: Procanace; specificEpithet: Procanaceacuminata; scientificNameAuthorship: Hardy & Delfinado, 1980; **Location:** islandGroup: Hawaiian Islands; island: Molokai; verbatimLocality: Pelekunu TNCH Preserve, Lower Pelekunu Valley; minimumElevationInMeters: 0; maximumElevationInMeters: 98; **Identification:** identifiedBy: WN Mathis; dateIdentified: 1992; **Event:** verbatimEventDate: 19.viii.1991; **Record Level:** institutionCode: BPBM
- **Type status:** Other material. **Occurrence:** catalogNumber: 2006004979; recordedBy: DA Polhemus; lifeStage: adult; **Taxon:** kingdom: Animalia; phylum: Arthropoda; class: Insecta; order: Diptera; family: Canacidae; genus: Procanace; specificEpithet: Procanaceacuminata; scientificNameAuthorship: Hardy & Delfinado, 1980; **Location:** islandGroup: Hawaiian Islands; island: Molokai; verbatimLocality: Pelekunu TNCH Preserve, Lower Pelekunu Valley; minimumElevationInMeters: 0; maximumElevationInMeters: 98; **Identification:** identifiedBy: WN Mathis; dateIdentified: 1992; **Event:** verbatimEventDate: 19.viii.1991; **Record Level:** institutionCode: BPBM
- **Type status:** Other material. **Occurrence:** catalogNumber: 2006004980; recordedBy: DA Polhemus; lifeStage: adult; **Taxon:** kingdom: Animalia; phylum: Arthropoda; class: Insecta; order: Diptera; family: Canacidae; genus: Procanace; specificEpithet: Procanaceacuminata; scientificNameAuthorship: Hardy & Delfinado, 1980; **Location:** islandGroup: Hawaiian Islands; island: Molokai; verbatimLocality: Pelekunu TNCH Preserve, Lower Pelekunu Valley; minimumElevationInMeters: 0; maximumElevationInMeters: 98; **Identification:** identifiedBy: WN Mathis; dateIdentified: 1992; **Event:** verbatimEventDate: 19.viii.1991; **Record Level:** institutionCode: BPBM
- **Type status:** Other material. **Occurrence:** catalogNumber: 2006004992; recordedBy: DA Polhemus; lifeStage: adult; **Taxon:** kingdom: Animalia; phylum: Arthropoda; class: Insecta; order: Diptera; family: Canacidae; genus: Procanace; specificEpithet: Procanaceacuminata; scientificNameAuthorship: Hardy & Delfinado, 1980; **Location:** islandGroup: Hawaiian Islands; island: Molokai; verbatimLocality: Pelekunu TNCH Preserve, Lower Pelekunu Valley; minimumElevationInMeters: 0; maximumElevationInMeters: 98; **Identification:** identifiedBy: WN Mathis; dateIdentified: 1992; **Event:** verbatimEventDate: 19.viii.1991; **Record Level:** institutionCode: BPBM
- **Type status:** Other material. **Occurrence:** catalogNumber: 2006004982; recordedBy: DA Polhemus; lifeStage: adult; **Taxon:** kingdom: Animalia; phylum: Arthropoda; class: Insecta; order: Diptera; family: Canacidae; genus: Procanace; specificEpithet: Procanaceacuminata; scientificNameAuthorship: Hardy & Delfinado, 1980; **Location:** islandGroup: Hawaiian Islands; island: Molokai; verbatimLocality: Pelekunu TNCH Preserve, Lower Pelekunu Valley; minimumElevationInMeters: 0; maximumElevationInMeters: 98; **Identification:** identifiedBy: WN Mathis; dateIdentified: 1992; **Event:** verbatimEventDate: 19.viii.1991; **Record Level:** institutionCode: BPBM
- **Type status:** Other material. **Occurrence:** catalogNumber: 2006005008; recordedBy: DA Polhemus; lifeStage: adult; **Taxon:** kingdom: Animalia; phylum: Arthropoda; class: Insecta; order: Diptera; family: Canacidae; genus: Procanace; specificEpithet: Procanaceacuminata; scientificNameAuthorship: Hardy & Delfinado, 1980; **Location:** islandGroup: Hawaiian Islands; island: Molokai; verbatimLocality: Pelekunu TNCH Preserve, Lower Pelekunu Valley; minimumElevationInMeters: 0; maximumElevationInMeters: 100; **Identification:** identifiedBy: WN Mathis; dateIdentified: 1992; **Event:** verbatimEventDate: 19.viii.1991; **Record Level:** institutionCode: BPBM
- **Type status:** Other material. **Occurrence:** catalogNumber: 2006004984; recordedBy: DA Polhemus; lifeStage: adult; **Taxon:** kingdom: Animalia; phylum: Arthropoda; class: Insecta; order: Diptera; family: Canacidae; genus: Procanace; specificEpithet: Procanaceacuminata; scientificNameAuthorship: Hardy & Delfinado, 1980; **Location:** islandGroup: Hawaiian Islands; island: Molokai; verbatimLocality: Pelekunu TNCH Preserve, Lower Pelekunu Valley; minimumElevationInMeters: 0; maximumElevationInMeters: 98; **Identification:** identifiedBy: WN Mathis; dateIdentified: 1992; **Event:** verbatimEventDate: 19.viii.1991; **Record Level:** institutionCode: BPBM
- **Type status:** Other material. **Occurrence:** catalogNumber: 2006004985; recordedBy: DA Polhemus; lifeStage: adult; **Taxon:** kingdom: Animalia; phylum: Arthropoda; class: Insecta; order: Diptera; family: Canacidae; genus: Procanace; specificEpithet: Procanaceacuminata; scientificNameAuthorship: Hardy & Delfinado, 1980; **Location:** islandGroup: Hawaiian Islands; island: Molokai; verbatimLocality: Pelekunu TNCH Preserve, Lower Pelekunu Valley; minimumElevationInMeters: 0; maximumElevationInMeters: 98; **Identification:** identifiedBy: WN Mathis; dateIdentified: 1992; **Event:** verbatimEventDate: 19.viii.1991; **Record Level:** institutionCode: BPBM
- **Type status:** Other material. **Occurrence:** catalogNumber: 2006004986; recordedBy: DA Polhemus; lifeStage: adult; **Taxon:** kingdom: Animalia; phylum: Arthropoda; class: Insecta; order: Diptera; family: Canacidae; genus: Procanace; specificEpithet: Procanaceacuminata; scientificNameAuthorship: Hardy & Delfinado, 1980; **Location:** islandGroup: Hawaiian Islands; island: Molokai; verbatimLocality: Pelekunu TNCH Preserve, Lower Pelekunu Valley; minimumElevationInMeters: 0; maximumElevationInMeters: 98; **Identification:** identifiedBy: WN Mathis; dateIdentified: 1992; **Event:** verbatimEventDate: 19.viii.1991; **Record Level:** institutionCode: BPBM
- **Type status:** Other material. **Occurrence:** catalogNumber: 2006004987; recordedBy: DA Polhemus; lifeStage: adult; **Taxon:** kingdom: Animalia; phylum: Arthropoda; class: Insecta; order: Diptera; family: Canacidae; genus: Procanace; specificEpithet: Procanaceacuminata; scientificNameAuthorship: Hardy & Delfinado, 1980; **Location:** islandGroup: Hawaiian Islands; island: Molokai; verbatimLocality: Pelekunu TNCH Preserve, Lower Pelekunu Valley; minimumElevationInMeters: 0; maximumElevationInMeters: 98; **Identification:** identifiedBy: WN Mathis; dateIdentified: 1992; **Event:** verbatimEventDate: 19.viii.1991; **Record Level:** institutionCode: BPBM
- **Type status:** Other material. **Occurrence:** catalogNumber: 2006004988; recordedBy: DA Polhemus; lifeStage: adult; **Taxon:** kingdom: Animalia; phylum: Arthropoda; class: Insecta; order: Diptera; family: Canacidae; genus: Procanace; specificEpithet: Procanaceacuminata; scientificNameAuthorship: Hardy & Delfinado, 1980; **Location:** islandGroup: Hawaiian Islands; island: Molokai; verbatimLocality: Pelekunu TNCH Preserve, Lower Pelekunu Valley; minimumElevationInMeters: 0; maximumElevationInMeters: 98; **Identification:** identifiedBy: WN Mathis; dateIdentified: 1992; **Event:** verbatimEventDate: 19.viii.1991; **Record Level:** institutionCode: BPBM
- **Type status:** Other material. **Occurrence:** catalogNumber: 2006005032; recordedBy: DA Polhemus; lifeStage: adult; **Taxon:** kingdom: Animalia; phylum: Arthropoda; class: Insecta; order: Diptera; family: Canacidae; genus: Procanace; specificEpithet: Procanaceacuminata; scientificNameAuthorship: Hardy & Delfinado, 1980; **Location:** islandGroup: Hawaiian Islands; island: Molokai; verbatimLocality: Pelekunu TNCH Preserve, Lower Pelekunu Valley; minimumElevationInMeters: 0; maximumElevationInMeters: 100; **Identification:** identifiedBy: WN Mathis; dateIdentified: 1992; **Event:** verbatimEventDate: 19.viii.1991; **Record Level:** institutionCode: BPBM
- **Type status:** Other material. **Occurrence:** catalogNumber: 2006004981; recordedBy: DA Polhemus; lifeStage: adult; **Taxon:** kingdom: Animalia; phylum: Arthropoda; class: Insecta; order: Diptera; family: Canacidae; genus: Procanace; specificEpithet: Procanaceacuminata; scientificNameAuthorship: Hardy & Delfinado, 1980; **Location:** islandGroup: Hawaiian Islands; island: Molokai; verbatimLocality: Pelekunu TNCH Preserve, Lower Pelekunu Valley; minimumElevationInMeters: 0; maximumElevationInMeters: 98; **Identification:** identifiedBy: WN Mathis; dateIdentified: 1992; **Event:** verbatimEventDate: 19.viii.1991; **Record Level:** institutionCode: BPBM
- **Type status:** Other material. **Occurrence:** catalogNumber: 2006004989; recordedBy: DA Polhemus; lifeStage: adult; **Taxon:** kingdom: Animalia; phylum: Arthropoda; class: Insecta; order: Diptera; family: Canacidae; genus: Procanace; specificEpithet: Procanaceacuminata; scientificNameAuthorship: Hardy & Delfinado, 1980; **Location:** islandGroup: Hawaiian Islands; island: Molokai; verbatimLocality: Pelekunu TNCH Preserve, Lower Pelekunu Valley; minimumElevationInMeters: 0; maximumElevationInMeters: 98; **Identification:** identifiedBy: WN Mathis; dateIdentified: 1992; **Event:** verbatimEventDate: 19.viii.1991; **Record Level:** institutionCode: BPBM
- **Type status:** Other material. **Occurrence:** catalogNumber: 2006005025; recordedBy: DA Polhemus; lifeStage: adult; **Taxon:** kingdom: Animalia; phylum: Arthropoda; class: Insecta; order: Diptera; family: Canacidae; genus: Procanace; specificEpithet: Procanaceacuminata; scientificNameAuthorship: Hardy & Delfinado, 1980; **Location:** islandGroup: Hawaiian Islands; island: Molokai; verbatimLocality: Pelekunu TNCH Preserve, Lower Pelekunu Valley; minimumElevationInMeters: 0; maximumElevationInMeters: 100; **Identification:** identifiedBy: WN Mathis; dateIdentified: 1992; **Event:** verbatimEventDate: 19.viii.1991; **Record Level:** institutionCode: BPBM
- **Type status:** Other material. **Occurrence:** catalogNumber: 2006005026; recordedBy: DA Polhemus; lifeStage: adult; **Taxon:** kingdom: Animalia; phylum: Arthropoda; class: Insecta; order: Diptera; family: Canacidae; genus: Procanace; specificEpithet: Procanaceacuminata; scientificNameAuthorship: Hardy & Delfinado, 1980; **Location:** islandGroup: Hawaiian Islands; island: Molokai; verbatimLocality: Pelekunu TNCH Preserve, Lower Pelekunu Valley; minimumElevationInMeters: 0; maximumElevationInMeters: 100; **Identification:** identifiedBy: WN Mathis; dateIdentified: 1992; **Event:** verbatimEventDate: 19.viii.1991; **Record Level:** institutionCode: BPBM
- **Type status:** Other material. **Occurrence:** catalogNumber: 2006005027; recordedBy: DA Polhemus; lifeStage: adult; **Taxon:** kingdom: Animalia; phylum: Arthropoda; class: Insecta; order: Diptera; family: Canacidae; genus: Procanace; specificEpithet: Procanaceacuminata; scientificNameAuthorship: Hardy & Delfinado, 1980; **Location:** islandGroup: Hawaiian Islands; island: Molokai; verbatimLocality: Pelekunu TNCH Preserve, Lower Pelekunu Valley; minimumElevationInMeters: 0; maximumElevationInMeters: 100; **Identification:** identifiedBy: WN Mathis; dateIdentified: 1992; **Event:** verbatimEventDate: 19.viii.1991; **Record Level:** institutionCode: BPBM
- **Type status:** Other material. **Occurrence:** catalogNumber: 2006005028; recordedBy: DA Polhemus; lifeStage: adult; **Taxon:** kingdom: Animalia; phylum: Arthropoda; class: Insecta; order: Diptera; family: Canacidae; genus: Procanace; specificEpithet: Procanaceacuminata; scientificNameAuthorship: Hardy & Delfinado, 1980; **Location:** islandGroup: Hawaiian Islands; island: Molokai; verbatimLocality: Pelekunu TNCH Preserve, Lower Pelekunu Valley; minimumElevationInMeters: 0; maximumElevationInMeters: 100; **Identification:** identifiedBy: WN Mathis; dateIdentified: 1992; **Event:** verbatimEventDate: 19.viii.1991; **Record Level:** institutionCode: BPBM
- **Type status:** Other material. **Occurrence:** catalogNumber: 2006005006; recordedBy: DA Polhemus; lifeStage: adult; **Taxon:** kingdom: Animalia; phylum: Arthropoda; class: Insecta; order: Diptera; family: Canacidae; genus: Procanace; specificEpithet: Procanaceacuminata; scientificNameAuthorship: Hardy & Delfinado, 1980; **Location:** islandGroup: Hawaiian Islands; island: Molokai; verbatimLocality: Pelekunu TNCH Preserve, Lower Pelekunu Valley; minimumElevationInMeters: 0; maximumElevationInMeters: 100; **Identification:** identifiedBy: WN Mathis; dateIdentified: 1992; **Event:** verbatimEventDate: 19.viii.1991; **Record Level:** institutionCode: BPBM
- **Type status:** Other material. **Occurrence:** catalogNumber: 2006005029; recordedBy: DA Polhemus; lifeStage: adult; **Taxon:** kingdom: Animalia; phylum: Arthropoda; class: Insecta; order: Diptera; family: Canacidae; genus: Procanace; specificEpithet: Procanaceacuminata; scientificNameAuthorship: Hardy & Delfinado, 1980; **Location:** islandGroup: Hawaiian Islands; island: Molokai; verbatimLocality: Pelekunu TNCH Preserve, Lower Pelekunu Valley; minimumElevationInMeters: 0; maximumElevationInMeters: 100; **Identification:** identifiedBy: WN Mathis; dateIdentified: 1992; **Event:** verbatimEventDate: 19.viii.1991; **Record Level:** institutionCode: BPBM
- **Type status:** Other material. **Occurrence:** catalogNumber: 2006005031; recordedBy: DA Polhemus; lifeStage: adult; **Taxon:** kingdom: Animalia; phylum: Arthropoda; class: Insecta; order: Diptera; family: Canacidae; genus: Procanace; specificEpithet: Procanaceacuminata; scientificNameAuthorship: Hardy & Delfinado, 1980; **Location:** islandGroup: Hawaiian Islands; island: Molokai; verbatimLocality: Pelekunu TNCH Preserve, Lower Pelekunu Valley; minimumElevationInMeters: 0; maximumElevationInMeters: 100; **Identification:** identifiedBy: WN Mathis; dateIdentified: 1992; **Event:** verbatimEventDate: 19.viii.1991; **Record Level:** institutionCode: BPBM
- **Type status:** Other material. **Occurrence:** catalogNumber: 2006005022; recordedBy: DA Polhemus; lifeStage: adult; **Taxon:** kingdom: Animalia; phylum: Arthropoda; class: Insecta; order: Diptera; family: Canacidae; genus: Procanace; specificEpithet: Procanaceacuminata; scientificNameAuthorship: Hardy & Delfinado, 1980; **Location:** islandGroup: Hawaiian Islands; island: Molokai; verbatimLocality: Pelekunu TNCH Preserve, Lower Pelekunu Valley; minimumElevationInMeters: 0; maximumElevationInMeters: 98; **Identification:** identifiedBy: WN Mathis; dateIdentified: 1992; **Event:** verbatimEventDate: 19.viii.1991; **Record Level:** institutionCode: BPBM
- **Type status:** Other material. **Occurrence:** catalogNumber: 2006005033; recordedBy: DA Polhemus; lifeStage: adult; **Taxon:** kingdom: Animalia; phylum: Arthropoda; class: Insecta; order: Diptera; family: Canacidae; genus: Procanace; specificEpithet: Procanaceacuminata; scientificNameAuthorship: Hardy & Delfinado, 1980; **Location:** islandGroup: Hawaiian Islands; island: Molokai; verbatimLocality: Pelekunu TNCH Preserve, Lower Pelekunu Valley; minimumElevationInMeters: 0; maximumElevationInMeters: 100; **Identification:** identifiedBy: WN Mathis; dateIdentified: 1992; **Event:** verbatimEventDate: 19.viii.1991; **Record Level:** institutionCode: BPBM
- **Type status:** Other material. **Occurrence:** catalogNumber: 2006005034; recordedBy: DA Polhemus; lifeStage: adult; **Taxon:** kingdom: Animalia; phylum: Arthropoda; class: Insecta; order: Diptera; family: Canacidae; genus: Procanace; specificEpithet: Procanaceacuminata; scientificNameAuthorship: Hardy & Delfinado, 1980; **Location:** islandGroup: Hawaiian Islands; island: Molokai; verbatimLocality: Pelekunu TNCH Preserve, Lower Pelekunu Valley; minimumElevationInMeters: 0; maximumElevationInMeters: 100; **Identification:** identifiedBy: WN Mathis; dateIdentified: 1992; **Event:** verbatimEventDate: 19.viii.1991; **Record Level:** institutionCode: BPBM
- **Type status:** Other material. **Occurrence:** catalogNumber: 2006005036; recordedBy: DA Polhemus; lifeStage: adult; **Taxon:** kingdom: Animalia; phylum: Arthropoda; class: Insecta; order: Diptera; family: Canacidae; genus: Procanace; specificEpithet: Procanaceacuminata; scientificNameAuthorship: Hardy & Delfinado, 1980; **Location:** islandGroup: Hawaiian Islands; island: Molokai; verbatimLocality: Pelekunu TNCH Preserve, Lower Pelekunu Valley; minimumElevationInMeters: 0; maximumElevationInMeters: 100; **Identification:** identifiedBy: WN Mathis; dateIdentified: 1992; **Event:** verbatimEventDate: 19.viii.1991; **Record Level:** institutionCode: BPBM
- **Type status:** Other material. **Occurrence:** catalogNumber: 2006005035; recordedBy: DA Polhemus; lifeStage: adult; **Taxon:** kingdom: Animalia; phylum: Arthropoda; class: Insecta; order: Diptera; family: Canacidae; genus: Procanace; specificEpithet: Procanaceacuminata; scientificNameAuthorship: Hardy & Delfinado, 1980; **Location:** islandGroup: Hawaiian Islands; island: Molokai; verbatimLocality: Pelekunu TNCH Preserve, Lower Pelekunu Valley; minimumElevationInMeters: 0; maximumElevationInMeters: 100; **Identification:** identifiedBy: WN Mathis; dateIdentified: 1992; **Event:** verbatimEventDate: 19.viii.1991; **Record Level:** institutionCode: BPBM
- **Type status:** Other material. **Occurrence:** catalogNumber: 2006005030; recordedBy: DA Polhemus; lifeStage: adult; **Taxon:** kingdom: Animalia; phylum: Arthropoda; class: Insecta; order: Diptera; family: Canacidae; genus: Procanace; specificEpithet: Procanaceacuminata; scientificNameAuthorship: Hardy & Delfinado, 1980; **Location:** islandGroup: Hawaiian Islands; island: Molokai; verbatimLocality: Pelekunu TNCH Preserve, Lower Pelekunu Valley; minimumElevationInMeters: 0; maximumElevationInMeters: 100; **Identification:** identifiedBy: WN Mathis; dateIdentified: 1992; **Event:** verbatimEventDate: 19.viii.1991; **Record Level:** institutionCode: BPBM
- **Type status:** Other material. **Occurrence:** catalogNumber: 2006005016; recordedBy: DA Polhemus; lifeStage: adult; **Taxon:** kingdom: Animalia; phylum: Arthropoda; class: Insecta; order: Diptera; family: Canacidae; genus: Procanace; specificEpithet: Procanaceacuminata; scientificNameAuthorship: Hardy & Delfinado, 1980; **Location:** islandGroup: Hawaiian Islands; island: Molokai; verbatimLocality: Pelekunu TNCH Preserve, Lower Pelekunu Valley; minimumElevationInMeters: 0; maximumElevationInMeters: 98; **Identification:** identifiedBy: WN Mathis; dateIdentified: 1992; **Event:** verbatimEventDate: 19.viii.1991; **Record Level:** institutionCode: BPBM
- **Type status:** Other material. **Occurrence:** catalogNumber: 2006004974; recordedBy: DA Polhemus; lifeStage: adult; **Taxon:** kingdom: Animalia; phylum: Arthropoda; class: Insecta; order: Diptera; family: Canacidae; genus: Procanace; specificEpithet: Procanaceacuminata; scientificNameAuthorship: Hardy & Delfinado, 1980; **Location:** islandGroup: Hawaiian Islands; island: Molokai; verbatimLocality: Pelekunu TNCH Preserve, Lower Pelekunu Valley; minimumElevationInMeters: 0; maximumElevationInMeters: 98; **Identification:** identifiedBy: WN Mathis; dateIdentified: 1992; **Event:** verbatimEventDate: 19.viii.1991; **Record Level:** institutionCode: BPBM
- **Type status:** Other material. **Occurrence:** catalogNumber: 2006005009; recordedBy: DA Polhemus; lifeStage: adult; **Taxon:** kingdom: Animalia; phylum: Arthropoda; class: Insecta; order: Diptera; family: Canacidae; genus: Procanace; specificEpithet: Procanaceacuminata; scientificNameAuthorship: Hardy & Delfinado, 1980; **Location:** islandGroup: Hawaiian Islands; island: Molokai; verbatimLocality: Pelekunu TNCH Preserve, Lower Pelekunu Valley; minimumElevationInMeters: 0; maximumElevationInMeters: 100; **Identification:** identifiedBy: WN Mathis; dateIdentified: 1992; **Event:** verbatimEventDate: 19.viii.1991; **Record Level:** institutionCode: BPBM
- **Type status:** Other material. **Occurrence:** catalogNumber: 2006005010; recordedBy: DA Polhemus; lifeStage: adult; **Taxon:** kingdom: Animalia; phylum: Arthropoda; class: Insecta; order: Diptera; family: Canacidae; genus: Procanace; specificEpithet: Procanaceacuminata; scientificNameAuthorship: Hardy & Delfinado, 1980; **Location:** islandGroup: Hawaiian Islands; island: Molokai; verbatimLocality: Pelekunu TNCH Preserve, Lower Pelekunu Valley; minimumElevationInMeters: 0; maximumElevationInMeters: 100; **Identification:** identifiedBy: WN Mathis; dateIdentified: 1992; **Event:** verbatimEventDate: 19.viii.1991; **Record Level:** institutionCode: BPBM
- **Type status:** Other material. **Occurrence:** catalogNumber: 2006005011; recordedBy: DA Polhemus; lifeStage: adult; **Taxon:** kingdom: Animalia; phylum: Arthropoda; class: Insecta; order: Diptera; family: Canacidae; genus: Procanace; specificEpithet: Procanaceacuminata; scientificNameAuthorship: Hardy & Delfinado, 1980; **Location:** islandGroup: Hawaiian Islands; island: Molokai; verbatimLocality: Pelekunu TNCH Preserve, Lower Pelekunu Valley; minimumElevationInMeters: 0; maximumElevationInMeters: 100; **Identification:** identifiedBy: WN Mathis; dateIdentified: 1992; **Event:** verbatimEventDate: 19.viii.1991; **Record Level:** institutionCode: BPBM
- **Type status:** Other material. **Occurrence:** catalogNumber: 2006005012; recordedBy: DA Polhemus; lifeStage: adult; **Taxon:** kingdom: Animalia; phylum: Arthropoda; class: Insecta; order: Diptera; family: Canacidae; genus: Procanace; specificEpithet: Procanaceacuminata; scientificNameAuthorship: Hardy & Delfinado, 1980; **Location:** islandGroup: Hawaiian Islands; island: Molokai; verbatimLocality: Pelekunu TNCH Preserve, Lower Pelekunu Valley; minimumElevationInMeters: 0; maximumElevationInMeters: 100; **Identification:** identifiedBy: WN Mathis; dateIdentified: 1992; **Event:** verbatimEventDate: 19.viii.1991; **Record Level:** institutionCode: BPBM
- **Type status:** Other material. **Occurrence:** catalogNumber: 2006005013; recordedBy: DA Polhemus; lifeStage: adult; **Taxon:** kingdom: Animalia; phylum: Arthropoda; class: Insecta; order: Diptera; family: Canacidae; genus: Procanace; specificEpithet: Procanaceacuminata; scientificNameAuthorship: Hardy & Delfinado, 1980; **Location:** islandGroup: Hawaiian Islands; island: Molokai; verbatimLocality: Pelekunu TNCH Preserve, Lower Pelekunu Valley; minimumElevationInMeters: 0; maximumElevationInMeters: 100; **Identification:** identifiedBy: WN Mathis; dateIdentified: 1992; **Event:** verbatimEventDate: 19.viii.1991; **Record Level:** institutionCode: BPBM
- **Type status:** Other material. **Occurrence:** catalogNumber: 2006005024; recordedBy: DA Polhemus; lifeStage: adult; **Taxon:** kingdom: Animalia; phylum: Arthropoda; class: Insecta; order: Diptera; family: Canacidae; genus: Procanace; specificEpithet: Procanaceacuminata; scientificNameAuthorship: Hardy & Delfinado, 1980; **Location:** islandGroup: Hawaiian Islands; island: Molokai; verbatimLocality: Pelekunu TNCH Preserve, Lower Pelekunu Valley; minimumElevationInMeters: 0; maximumElevationInMeters: 100; **Identification:** identifiedBy: WN Mathis; dateIdentified: 1992; **Event:** verbatimEventDate: 19.viii.1991; **Record Level:** institutionCode: BPBM
- **Type status:** Other material. **Occurrence:** catalogNumber: 2006005015; recordedBy: DA Polhemus; lifeStage: adult; **Taxon:** kingdom: Animalia; phylum: Arthropoda; class: Insecta; order: Diptera; family: Canacidae; genus: Procanace; specificEpithet: Procanaceacuminata; scientificNameAuthorship: Hardy & Delfinado, 1980; **Location:** islandGroup: Hawaiian Islands; island: Molokai; verbatimLocality: Pelekunu TNCH Preserve, Lower Pelekunu Valley; minimumElevationInMeters: 0; maximumElevationInMeters: 98; **Identification:** identifiedBy: WN Mathis; dateIdentified: 1992; **Event:** verbatimEventDate: 19.viii.1991; **Record Level:** institutionCode: BPBM
- **Type status:** Other material. **Occurrence:** catalogNumber: 2006005023; recordedBy: DA Polhemus; lifeStage: adult; **Taxon:** kingdom: Animalia; phylum: Arthropoda; class: Insecta; order: Diptera; family: Canacidae; genus: Procanace; specificEpithet: Procanaceacuminata; scientificNameAuthorship: Hardy & Delfinado, 1980; **Location:** islandGroup: Hawaiian Islands; island: Molokai; verbatimLocality: Pelekunu TNCH Preserve, Lower Pelekunu Valley; minimumElevationInMeters: 0; maximumElevationInMeters: 100; **Identification:** identifiedBy: WN Mathis; dateIdentified: 1992; **Event:** verbatimEventDate: 19.viii.1991; **Record Level:** institutionCode: BPBM
- **Type status:** Other material. **Occurrence:** catalogNumber: 2006005017; recordedBy: DA Polhemus; lifeStage: adult; **Taxon:** kingdom: Animalia; phylum: Arthropoda; class: Insecta; order: Diptera; family: Canacidae; genus: Procanace; specificEpithet: Procanaceacuminata; scientificNameAuthorship: Hardy & Delfinado, 1980; **Location:** islandGroup: Hawaiian Islands; island: Molokai; verbatimLocality: Pelekunu TNCH Preserve, Lower Pelekunu Valley; minimumElevationInMeters: 0; maximumElevationInMeters: 98; **Identification:** identifiedBy: WN Mathis; dateIdentified: 1992; **Event:** verbatimEventDate: 19.viii.1991; **Record Level:** institutionCode: BPBM
- **Type status:** Other material. **Occurrence:** catalogNumber: 2006005018; recordedBy: DA Polhemus; lifeStage: adult; **Taxon:** kingdom: Animalia; phylum: Arthropoda; class: Insecta; order: Diptera; family: Canacidae; genus: Procanace; specificEpithet: Procanaceacuminata; scientificNameAuthorship: Hardy & Delfinado, 1980; **Location:** islandGroup: Hawaiian Islands; island: Molokai; verbatimLocality: Pelekunu TNCH Preserve, Lower Pelekunu Valley; minimumElevationInMeters: 0; maximumElevationInMeters: 98; **Identification:** identifiedBy: WN Mathis; dateIdentified: 1992; **Event:** verbatimEventDate: 19.viii.1991; **Record Level:** institutionCode: BPBM
- **Type status:** Other material. **Occurrence:** catalogNumber: 2006005019; recordedBy: DA Polhemus; lifeStage: adult; **Taxon:** kingdom: Animalia; phylum: Arthropoda; class: Insecta; order: Diptera; family: Canacidae; genus: Procanace; specificEpithet: Procanaceacuminata; scientificNameAuthorship: Hardy & Delfinado, 1980; **Location:** islandGroup: Hawaiian Islands; island: Molokai; verbatimLocality: Pelekunu TNCH Preserve, Lower Pelekunu Valley; minimumElevationInMeters: 0; maximumElevationInMeters: 98; **Identification:** identifiedBy: WN Mathis; dateIdentified: 1992; **Event:** verbatimEventDate: 19.viii.1991; **Record Level:** institutionCode: BPBM
- **Type status:** Other material. **Occurrence:** catalogNumber: 2006005020; recordedBy: DA Polhemus; lifeStage: adult; **Taxon:** kingdom: Animalia; phylum: Arthropoda; class: Insecta; order: Diptera; family: Canacidae; genus: Procanace; specificEpithet: Procanaceacuminata; scientificNameAuthorship: Hardy & Delfinado, 1980; **Location:** islandGroup: Hawaiian Islands; island: Molokai; verbatimLocality: Pelekunu TNCH Preserve, Lower Pelekunu Valley; minimumElevationInMeters: 0; maximumElevationInMeters: 98; **Identification:** identifiedBy: WN Mathis; dateIdentified: 1992; **Event:** verbatimEventDate: 19.viii.1991; **Record Level:** institutionCode: BPBM
- **Type status:** Other material. **Occurrence:** catalogNumber: 2006005021; recordedBy: DA Polhemus; lifeStage: adult; **Taxon:** kingdom: Animalia; phylum: Arthropoda; class: Insecta; order: Diptera; family: Canacidae; genus: Procanace; specificEpithet: Procanaceacuminata; scientificNameAuthorship: Hardy & Delfinado, 1980; **Location:** islandGroup: Hawaiian Islands; island: Molokai; verbatimLocality: Pelekunu TNCH Preserve, Lower Pelekunu Valley; minimumElevationInMeters: 0; maximumElevationInMeters: 98; **Identification:** identifiedBy: WN Mathis; dateIdentified: 1992; **Event:** verbatimEventDate: 19.viii.1991; **Record Level:** institutionCode: BPBM
- **Type status:** Other material. **Occurrence:** catalogNumber: 2006005007; recordedBy: DA Polhemus; lifeStage: adult; **Taxon:** kingdom: Animalia; phylum: Arthropoda; class: Insecta; order: Diptera; family: Canacidae; genus: Procanace; specificEpithet: Procanaceacuminata; scientificNameAuthorship: Hardy & Delfinado, 1980; **Location:** islandGroup: Hawaiian Islands; island: Molokai; verbatimLocality: Pelekunu TNCH Preserve, Lower Pelekunu Valley; minimumElevationInMeters: 0; maximumElevationInMeters: 100; **Identification:** identifiedBy: WN Mathis; dateIdentified: 1992; **Event:** verbatimEventDate: 19.viii.1991; **Record Level:** institutionCode: BPBM
- **Type status:** Other material. **Occurrence:** catalogNumber: 2006005014; recordedBy: DA Polhemus; lifeStage: adult; **Taxon:** kingdom: Animalia; phylum: Arthropoda; class: Insecta; order: Diptera; family: Canacidae; genus: Procanace; specificEpithet: Procanaceacuminata; scientificNameAuthorship: Hardy & Delfinado, 1980; **Location:** islandGroup: Hawaiian Islands; island: Molokai; verbatimLocality: Pelekunu TNCH Preserve, Lower Pelekunu Valley; minimumElevationInMeters: 0; maximumElevationInMeters: 100; **Identification:** identifiedBy: WN Mathis; dateIdentified: 1992; **Event:** verbatimEventDate: 19.viii.1991; **Record Level:** institutionCode: BPBM
- **Type status:** Other material. **Occurrence:** catalogNumber: 2006004976; recordedBy: DA Polhemus; lifeStage: adult; **Taxon:** kingdom: Animalia; phylum: Arthropoda; class: Insecta; order: Diptera; family: Canacidae; genus: Procanace; specificEpithet: Procanaceacuminata; scientificNameAuthorship: Hardy & Delfinado, 1980; **Location:** islandGroup: Hawaiian Islands; island: Molokai; verbatimLocality: Pelekunu TNCH Preserve, Lower Pelekunu Valley; minimumElevationInMeters: 0; maximumElevationInMeters: 98; **Identification:** identifiedBy: WN Mathis; dateIdentified: 1992; **Event:** verbatimEventDate: 19.viii.1991; **Record Level:** institutionCode: BPBM
- **Type status:** Other material. **Occurrence:** catalogNumber: 2006004969; recordedBy: DA Polhemus; lifeStage: adult; **Taxon:** kingdom: Animalia; phylum: Arthropoda; class: Insecta; order: Diptera; family: Canacidae; genus: Procanace; specificEpithet: Procanaceacuminata; scientificNameAuthorship: Hardy & Delfinado, 1980; **Location:** islandGroup: Hawaiian Islands; island: Molokai; verbatimLocality: Pelekunu TNCH Preserve, Lower Pelekunu Valley; minimumElevationInMeters: 0; maximumElevationInMeters: 98; **Identification:** identifiedBy: WN Mathis; dateIdentified: 1992; **Event:** verbatimEventDate: 19.viii.1991; **Record Level:** institutionCode: BPBM
- **Type status:** Other material. **Occurrence:** catalogNumber: 2006004970; recordedBy: DA Polhemus; lifeStage: adult; **Taxon:** kingdom: Animalia; phylum: Arthropoda; class: Insecta; order: Diptera; family: Canacidae; genus: Procanace; specificEpithet: Procanaceacuminata; scientificNameAuthorship: Hardy & Delfinado, 1980; **Location:** islandGroup: Hawaiian Islands; island: Molokai; verbatimLocality: Pelekunu TNCH Preserve, Lower Pelekunu Valley; minimumElevationInMeters: 0; maximumElevationInMeters: 98; **Identification:** identifiedBy: WN Mathis; dateIdentified: 1992; **Event:** verbatimEventDate: 19.viii.1991; **Record Level:** institutionCode: BPBM
- **Type status:** Other material. **Occurrence:** catalogNumber: 2006004971; recordedBy: DA Polhemus; lifeStage: adult; **Taxon:** kingdom: Animalia; phylum: Arthropoda; class: Insecta; order: Diptera; family: Canacidae; genus: Procanace; specificEpithet: Procanaceacuminata; scientificNameAuthorship: Hardy & Delfinado, 1980; **Location:** islandGroup: Hawaiian Islands; island: Molokai; verbatimLocality: Pelekunu TNCH Preserve, Lower Pelekunu Valley; minimumElevationInMeters: 0; maximumElevationInMeters: 98; **Identification:** identifiedBy: WN Mathis; dateIdentified: 1992; **Event:** verbatimEventDate: 19.viii.1991; **Record Level:** institutionCode: BPBM
- **Type status:** Other material. **Occurrence:** catalogNumber: 2006004972; recordedBy: DA Polhemus; lifeStage: adult; **Taxon:** kingdom: Animalia; phylum: Arthropoda; class: Insecta; order: Diptera; family: Canacidae; genus: Procanace; specificEpithet: Procanaceacuminata; scientificNameAuthorship: Hardy & Delfinado, 1980; **Location:** islandGroup: Hawaiian Islands; island: Molokai; verbatimLocality: Pelekunu TNCH Preserve, Lower Pelekunu Valley; minimumElevationInMeters: 0; maximumElevationInMeters: 98; **Identification:** identifiedBy: WN Mathis; dateIdentified: 1992; **Event:** verbatimEventDate: 19.viii.1991; **Record Level:** institutionCode: BPBM
- **Type status:** Other material. **Occurrence:** catalogNumber: 2006004973; recordedBy: DA Polhemus; lifeStage: adult; **Taxon:** kingdom: Animalia; phylum: Arthropoda; class: Insecta; order: Diptera; family: Canacidae; genus: Procanace; specificEpithet: Procanaceacuminata; scientificNameAuthorship: Hardy & Delfinado, 1980; **Location:** islandGroup: Hawaiian Islands; island: Molokai; verbatimLocality: Pelekunu TNCH Preserve, Lower Pelekunu Valley; minimumElevationInMeters: 0; maximumElevationInMeters: 98; **Identification:** identifiedBy: WN Mathis; dateIdentified: 1992; **Event:** verbatimEventDate: 19.viii.1991; **Record Level:** institutionCode: BPBM
- **Type status:** Other material. **Occurrence:** recordedBy: DA Polhemus; individualCount: 20; sex: 13 males, 7 females; lifeStage: adult; **Taxon:** kingdom: Animalia; phylum: Arthropoda; class: Insecta; order: Diptera; family: Canacidae; genus: Procanace; specificEpithet: Procanaceacuminata; **Location:** islandGroup: Hawaiian Islands; island: Molokai; verbatimLocality: Pelekunu TNCH Preserve, Lower Pelekunu Valley; minimumElevationInMeters: 0; maximumElevationInMeters: 30; **Event:** verbatimEventDate: 19.viii.1991; **Record Level:** institutionCode: USNM
- **Type status:** Other material. **Occurrence:** recordedBy: DA Polhemus; individualCount: 12; sex: 7 males, 5 females; lifeStage: adult; **Taxon:** kingdom: Animalia; phylum: Arthropoda; class: Insecta; order: Diptera; family: Canacidae; genus: Procanace; specificEpithet: Procanaceacuminata; **Location:** islandGroup: Hawaiian Islands; island: Molokai; verbatimLocality: Pelekunu TNCH Preserve, Lower Pelekunu Valley; minimumElevationInMeters: 0; maximumElevationInMeters: 100; **Event:** verbatimEventDate: 19.viii.1991; **Record Level:** institutionCode: USNM
- **Type status:** Other material. **Occurrence:** recordedBy: PM O'Grady, RT Lapoint, GM Bennett, NA Pantoja; lifeStage: adult; **Taxon:** kingdom: Animalia; phylum: Arthropoda; class: Insecta; order: Diptera; family: Canacidae; genus: Procanace; specificEpithet: Procanaceacuminata; scientificNameAuthorship: Hardy & Delfinado, 1980; **Location:** islandGroup: Hawaiian Islands; island: Hawaii; verbatimLocality: Kawainui Stream, 6 ton bridge on Scenic Loop; **Identification:** identifiedBy: PM O'Grady; dateIdentified: 2014; **Event:** verbatimEventDate: 2.viii.2010; **Record Level:** institutionCode: EMEC; collectionCode: 205136

##### Ecological interactions

###### Native status

endemic

##### Distribution

**Figure 10.**
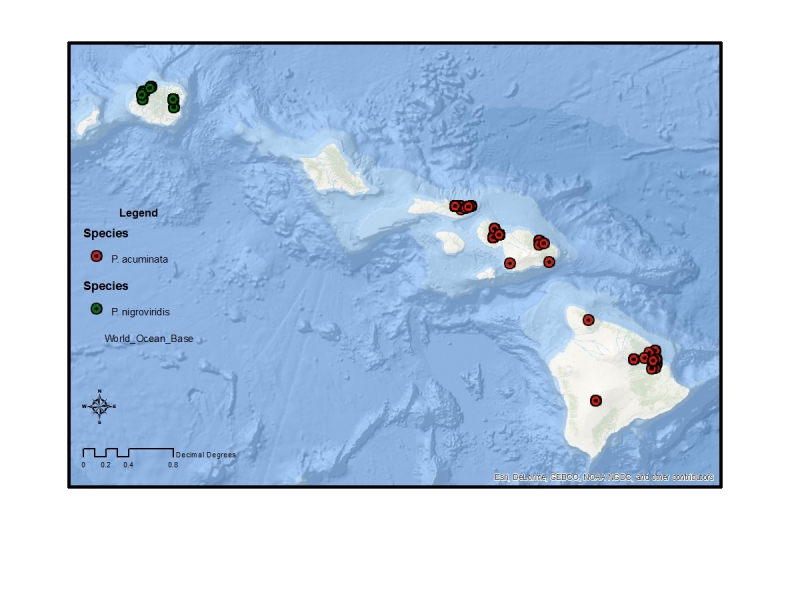
Distribution of *P. acuminata* on Oahu, Molokai, Maui and Hawaii and *P. nigroviridis* on Kauai.

HAWAIIAN ISLANDS: Molokai, Maui, Hawaii (Fig. 10​).

##### Notes

Hardy and Delfinado (1980), [original description; female terminalia (dorsal and ventral), spermathecae, ninth sternum, epandrium and surstylus]; Mathis (1992), [World Catalog]; Nishida (2002), [Hawaiian Arthropod Checklist].

#### Procanace bifurcata

Hardy and Delfinado, 1980

##### Materials

- **Type status:** Holotype. **Occurrence:** recordedBy: MD Delfinado; individualCount: 1; sex: male; lifeStage: adult; **Taxon:** kingdom: Animalia; phylum: Arthropoda; class: Insecta; order: Diptera; family: Canacidae; genus: Procanace; specificEpithet: Procanacebifurcata; scientificNameAuthorship: Hardy & Delfinado, 1980; **Location:** islandGroup: Hawaiian Islands; island: Oahu; verbatimLocality: Opaeula Stream; verbatimElevation: 1150 ft.; **Identification:** identifiedBy: DE Hardy & MD Delfinado; dateIdentified: 1980; **Event:** verbatimEventDate: 12.vi.1970; **Record Level:** institutionCode: BPBM
- **Type status:** Paratype. **Occurrence:** recordedBy: FX Williams; lifeStage: adult; **Taxon:** kingdom: Animalia; phylum: Arthropoda; class: Insecta; order: Diptera; family: Canacidae; genus: Procanace; specificEpithet: Procanacebifurcata; scientificNameAuthorship: Hardy & Delfinado, 1980; **Location:** islandGroup: Hawaiian Islands; island: Oahu; verbatimLocality: Waihinui, Manoa; **Identification:** identifiedBy: DE Hardy & MD Delfinado; dateIdentified: 1980; **Event:** verbatimEventDate: 13.viii.1933
- **Type status:** Paratype. **Occurrence:** recordedBy: FX Williams; lifeStage: adult; **Taxon:** kingdom: Animalia; phylum: Arthropoda; class: Insecta; order: Diptera; family: Canacidae; genus: Procanace; specificEpithet: Procanacebifurcata; scientificNameAuthorship: Hardy & Delfinado, 1980; **Location:** islandGroup: Hawaiian Islands; island: Oahu; verbatimLocality: Waihai-iki, Manoa Valley; **Identification:** identifiedBy: DE Hardy & MD Delfinado; dateIdentified: 1980; **Event:** verbatimEventDate: 22.iii.1936
- **Type status:** Paratype. **Occurrence:** recordedBy: DE Hardy; individualCount: 1; lifeStage: adult; **Taxon:** kingdom: Animalia; phylum: Arthropoda; class: Insecta; order: Diptera; family: Canacidae; genus: Procanace; specificEpithet: Procanacebifurcata; scientificNameAuthorship: Hardy & Delfinado, 1980; **Location:** islandGroup: Hawaiian Islands; island: Oahu; verbatimLocality: Head of Kaluanui Valley; **Identification:** identifiedBy: DE Hardy & MD Delfinado; dateIdentified: 1980; **Event:** verbatimEventDate: v.1951; **Record Level:** institutionCode: UHM
- **Type status:** Paratype. **Occurrence:** recordedBy: DE Hardy; lifeStage: adult; **Taxon:** kingdom: Animalia; phylum: Arthropoda; class: Insecta; order: Diptera; family: Canacidae; genus: Procanace; specificEpithet: Procanacebifurcata; scientificNameAuthorship: Hardy & Delfinado, 1980; **Location:** islandGroup: Hawaiian Islands; island: Oahu; verbatimLocality: Head of Kalunui Valley; **Identification:** identifiedBy: DE Hardy & MD Delfinado; dateIdentified: 1980; **Event:** verbatimEventDate: v.1951
- **Type status:** Paratype. **Occurrence:** recordedBy: MS Adachi, DE Hardy; lifeStage: adult; **Taxon:** kingdom: Animalia; phylum: Arthropoda; class: Insecta; order: Diptera; family: Canacidae; genus: Procanace; specificEpithet: Procanacebifurcata; scientificNameAuthorship: Hardy & Delfinado, 1980; **Location:** islandGroup: Hawaiian Islands; island: Oahu; verbatimLocality: Manoa Valley; **Identification:** identifiedBy: DE Hardy & MD Delfinado; dateIdentified: 1980; **Event:** verbatimEventDate: i.1952
- **Type status:** Paratype. **Occurrence:** recordedBy: MS Adachi; individualCount: 1; lifeStage: adult; **Taxon:** kingdom: Animalia; phylum: Arthropoda; class: Insecta; order: Diptera; family: Canacidae; genus: Procanace; specificEpithet: Procanacebifurcata; scientificNameAuthorship: Hardy & Delfinado, 1980; **Location:** islandGroup: Hawaiian Islands; island: Oahu; verbatimLocality: Manoa Valley; **Identification:** identifiedBy: DE Hardy & MD Delfinado; dateIdentified: 1980; **Event:** verbatimEventDate: i.1952; **Record Level:** institutionCode: UHM
- **Type status:** Paratype. **Occurrence:** recordedBy: MS Adachi, DE Hardy; lifeStage: adult; **Taxon:** kingdom: Animalia; phylum: Arthropoda; class: Insecta; order: Diptera; family: Canacidae; genus: Procanace; specificEpithet: Procanacebifurcata; scientificNameAuthorship: Hardy & Delfinado, 1980; **Location:** islandGroup: Hawaiian Islands; island: Oahu; verbatimLocality: Manoa Valley; **Identification:** identifiedBy: DE Hardy & MD Delfinado; dateIdentified: 1980; **Event:** verbatimEventDate: i.1953
- **Type status:** Paratype. **Occurrence:** recordedBy: JA Tenorio; lifeStage: adult; **Taxon:** kingdom: Animalia; phylum: Arthropoda; class: Insecta; order: Diptera; family: Canacidae; genus: Procanace; specificEpithet: Procanacebifurcata; scientificNameAuthorship: Hardy & Delfinado, 1980; **Location:** islandGroup: Hawaiian Islands; island: Oahu; verbatimLocality: Punaluu Stream; **Identification:** identifiedBy: DE Hardy & MD Delfinado; dateIdentified: 1980; **Event:** verbatimEventDate: 24.v.1962
- **Type status:** Paratype. **Occurrence:** recordedBy: MD Delfinado; individualCount: 26; lifeStage: adult; **Taxon:** kingdom: Animalia; phylum: Arthropoda; class: Insecta; order: Diptera; family: Canacidae; genus: Procanace; specificEpithet: Procanacebifurcata; scientificNameAuthorship: Hardy & Delfinado, 1980; **Location:** islandGroup: Hawaiian Islands; island: Oahu; verbatimLocality: Opaeula Stream; verbatimElevation: 1150 ft.; **Identification:** identifiedBy: DE Hardy & MD Delfinado; dateIdentified: 1980; **Event:** verbatimEventDate: 12.v.1970; **Record Level:** institutionCode: UHM
- **Type status:** Paratype. **Occurrence:** recordedBy: MD Delfinado; lifeStage: adult; **Taxon:** kingdom: Animalia; phylum: Arthropoda; class: Insecta; order: Diptera; family: Canacidae; genus: Procanace; specificEpithet: Procanacebifurcata; scientificNameAuthorship: Hardy & Delfinado, 1980; **Location:** islandGroup: Hawaiian Islands; island: Oahu; verbatimLocality: Kahalanui Stream; **Identification:** identifiedBy: DE Hardy & MD Delfinado; dateIdentified: 1980; **Event:** verbatimEventDate: 26.v.1970
- **Type status:** Paratype. **Occurrence:** recordedBy: MD Delfinado; individualCount: 1; lifeStage: adult; **Taxon:** kingdom: Animalia; phylum: Arthropoda; class: Insecta; order: Diptera; family: Canacidae; genus: Procanace; specificEpithet: Procanacebifurcata; scientificNameAuthorship: Hardy & Delfinado, 1980; **Location:** islandGroup: Hawaiian Islands; island: Oahu; verbatimLocality: Kawai-iki Stream; **Identification:** identifiedBy: DE Hardy & MD Delfinado; dateIdentified: 1980; **Event:** verbatimEventDate: 12.vi.1970; **Record Level:** institutionCode: UHM
- **Type status:** Paratype. **Occurrence:** recordedBy: MD Delfinado; individualCount: 1; sex: female; lifeStage: adult; **Taxon:** kingdom: Animalia; phylum: Arthropoda; class: Insecta; order: Diptera; family: Canacidae; genus: Procanace; specificEpithet: Procanacebifurcata; scientificNameAuthorship: Hardy & Delfinado, 1980; **Location:** islandGroup: Hawaiian Islands; island: Oahu; verbatimLocality: Opaeula Stream; verbatimElevation: 1150 ft.; **Identification:** identifiedBy: DE Hardy & MD Delfinado; dateIdentified: 1980; **Event:** verbatimEventDate: 12.vi.1970; **Record Level:** institutionCode: BPBM
- **Type status:** Paratype. **Occurrence:** recordedBy: MD Delfinado; lifeStage: adult; **Taxon:** kingdom: Animalia; phylum: Arthropoda; class: Insecta; order: Diptera; family: Canacidae; genus: Procanace; specificEpithet: Procanacebifurcata; scientificNameAuthorship: Hardy & Delfinado, 1980; **Location:** islandGroup: Hawaiian Islands; island: Oahu; verbatimLocality: Opaeula Stream; verbatimElevation: 1150 ft.; **Identification:** identifiedBy: DE Hardy & MD Delfinado; dateIdentified: 1980; **Event:** verbatimEventDate: 12.vi.1970
- **Type status:** Paratype. **Occurrence:** recordedBy: MD Delfinado; lifeStage: adult; **Taxon:** kingdom: Animalia; phylum: Arthropoda; class: Insecta; order: Diptera; family: Canacidae; genus: Procanace; specificEpithet: Procanacebifurcata; scientificNameAuthorship: Hardy & Delfinado, 1980; **Location:** islandGroup: Hawaiian Islands; island: Oahu; verbatimLocality: Kawai-iki Stream; **Identification:** identifiedBy: DE Hardy & MD Delfinado; dateIdentified: 1980; **Event:** verbatimEventDate: 12.vi.1970
- **Type status:** Paratype. **Occurrence:** recordedBy: MD Delfinado, DE Hardy; lifeStage: adult; **Taxon:** kingdom: Animalia; phylum: Arthropoda; class: Insecta; order: Diptera; family: Canacidae; genus: Procanace; specificEpithet: Procanacebifurcata; scientificNameAuthorship: Hardy & Delfinado, 1980; **Location:** islandGroup: Hawaiian Islands; island: Kauai; verbatimLocality: Hanakapiai, mouth of River nr beach; **Identification:** identifiedBy: DE Hardy & MD Delfinado; dateIdentified: 1980; **Event:** verbatimEventDate: 10.viii.1971
- **Type status:** Other material. **Occurrence:** catalogNumber: 2006005050; recordedBy: FX Williams; lifeStage: adult; **Taxon:** kingdom: Animalia; phylum: Arthropoda; class: Insecta; order: Diptera; family: Canacidae; genus: Procanace; specificEpithet: Procanacebifurcata; scientificNameAuthorship: Hardy & Delfinado, 1980; **Location:** islandGroup: Hawaiian Islands; island: Oahu; verbatimLocality: Honolulu, Manoa; **Identification:** identifiedBy: DE Hardy & MD Delfinado; dateIdentified: 1980; **Event:** verbatimEventDate: 13.viii.1933; **Record Level:** institutionCode: BPBM
- **Type status:** Other material. **Occurrence:** catalogNumber: 2006005042; recordedBy: FX Williams; lifeStage: adult; **Taxon:** kingdom: Animalia; phylum: Arthropoda; class: Insecta; order: Diptera; family: Canacidae; genus: Procanace; specificEpithet: Procanacebifurcata; scientificNameAuthorship: Hardy & Delfinado, 1980; **Location:** islandGroup: Hawaiian Islands; island: Oahu; verbatimLocality: Manoa Valley, Waihi-iki; **Identification:** identifiedBy: DE Hardy & MD Delfinado; dateIdentified: 1980; **Event:** verbatimEventDate: 22.iii.1936; **Record Level:** institutionCode: BPBM
- **Type status:** Other material. **Occurrence:** catalogNumber: 2006005048; recordedBy: DE Hardy; lifeStage: adult; **Taxon:** kingdom: Animalia; phylum: Arthropoda; class: Insecta; order: Diptera; family: Canacidae; genus: Procanace; specificEpithet: Procanacebifurcata; scientificNameAuthorship: Hardy & Delfinado, 1980; **Location:** islandGroup: Hawaiian Islands; island: Oahu; verbatimLocality: Head of Kaluanui Valley; **Identification:** identifiedBy: DE Hardy & MD Delfinado; dateIdentified: 1980; **Event:** verbatimEventDate: May 1951; **Record Level:** institutionCode: BPBM
- **Type status:** Other material. **Occurrence:** catalogNumber: 2006005049; recordedBy: DE Hardy; lifeStage: adult; **Taxon:** kingdom: Animalia; phylum: Arthropoda; class: Insecta; order: Diptera; family: Canacidae; genus: Procanace; specificEpithet: Procanacebifurcata; scientificNameAuthorship: Hardy & Delfinado, 1980; **Location:** islandGroup: Hawaiian Islands; island: Oahu; verbatimLocality: Head of Kaluanui Valley; **Identification:** identifiedBy: DE Hardy & MD Delfinado; dateIdentified: 1980; **Event:** verbatimEventDate: v.1951; **Record Level:** institutionCode: BPBM
- **Type status:** Other material. **Occurrence:** catalogNumber: 2006005051; recordedBy: MS Adachi; lifeStage: adult; **Taxon:** kingdom: Animalia; phylum: Arthropoda; class: Insecta; order: Diptera; family: Canacidae; genus: Procanace; specificEpithet: Procanacebifurcata; scientificNameAuthorship: Hardy & Delfinado, 1980; **Location:** islandGroup: Hawaiian Islands; island: Oahu; verbatimLocality: Manoa Valley; **Identification:** identifiedBy: DE Hardy & MD Delfinado; dateIdentified: 1980; **Event:** verbatimEventDate: January 1952; **Record Level:** institutionCode: BPBM
- **Type status:** Other material. **Occurrence:** catalogNumber: 2006005043; recordedBy: JA Tenorio; lifeStage: adult; **Taxon:** kingdom: Animalia; phylum: Arthropoda; class: Insecta; order: Diptera; family: Canacidae; genus: Procanace; specificEpithet: Procanacebifurcata; scientificNameAuthorship: Hardy & Delfinado, 1980; **Location:** islandGroup: Hawaiian Islands; island: Oahu; verbatimLocality: Punaluu; **Identification:** identifiedBy: DE Hardy & MD Delfinado; dateIdentified: 1980; **Event:** verbatimEventDate: 24.v.1968; **Record Level:** institutionCode: BPBM
- **Type status:** Other material. **Occurrence:** recordedBy: DE Hardy; individualCount: 58; lifeStage: adult; **Taxon:** kingdom: Animalia; phylum: Arthropoda; class: Insecta; order: Diptera; family: Canacidae; genus: Procanace; specificEpithet: Procanacebifurcata; scientificNameAuthorship: Hardy & Delfinado, 1980; **Location:** islandGroup: Hawaiian Islands; island: Molokai; verbatimLocality: Moaula Valley; **Event:** verbatimEventDate: 16.iii.1970; **Record Level:** institutionCode: UHM
- **Type status:** Other material. **Occurrence:** catalogNumber: 2006005041; recordedBy: MD Delfinado; lifeStage: adult; **Taxon:** kingdom: Animalia; phylum: Arthropoda; class: Insecta; order: Diptera; family: Canacidae; genus: Procanace; specificEpithet: Procanacebifurcata; scientificNameAuthorship: Hardy & Delfinado, 1980; **Location:** islandGroup: Hawaiian Islands; island: Oahu; verbatimLocality: Kahalanui Stream; **Identification:** identifiedBy: DE Hardy & MD Delfinado; dateIdentified: 1980; **Event:** verbatimEventDate: 26.v.1970; **Record Level:** institutionCode: BPBM
- **Type status:** Other material. **Occurrence:** recordedBy: MD Delfinado; individualCount: 5; lifeStage: adult; **Taxon:** kingdom: Animalia; phylum: Arthropoda; class: Insecta; order: Diptera; family: Canacidae; genus: Procanace; specificEpithet: Procanacebifurcata; scientificNameAuthorship: Hardy & Delfinado, 1980; **Location:** islandGroup: Hawaiian Islands; island: Oahu; verbatimLocality: Opaeula Stream; **Event:** verbatimEventDate: 12.vi.1970; **Record Level:** institutionCode: UHM
- **Type status:** Other material. **Occurrence:** recordedBy: JA Tenorio; individualCount: 3; lifeStage: adult; **Taxon:** kingdom: Animalia; phylum: Arthropoda; class: Insecta; order: Diptera; family: Canacidae; genus: Procanace; specificEpithet: Procanacebifurcata; scientificNameAuthorship: Hardy & Delfinado, 1980; **Location:** islandGroup: Hawaiian Islands; island: Oahu; verbatimLocality: Opaeula Stream; **Event:** verbatimEventDate: 12.vi.1970; **Record Level:** institutionCode: UHM
- **Type status:** Other material. **Occurrence:** catalogNumber: 2006005045; recordedBy: MD Delfinado; lifeStage: adult; **Taxon:** kingdom: Animalia; phylum: Arthropoda; class: Insecta; order: Diptera; family: Canacidae; genus: Procanace; specificEpithet: Procanacebifurcata; scientificNameAuthorship: Hardy & Delfinado, 1980; **Location:** islandGroup: Hawaiian Islands; island: Oahu; verbatimLocality: Opaeula Stream; minimumElevationInMeters: 1150; **Identification:** identifiedBy: DE Hardy & MD Delfinado; dateIdentified: 1980; **Event:** verbatimEventDate: 12.vi.1970; **Record Level:** institutionCode: BPBM
- **Type status:** Other material. **Occurrence:** catalogNumber: 2006005044; recordedBy: MD Delfinado; lifeStage: adult; **Taxon:** kingdom: Animalia; phylum: Arthropoda; class: Insecta; order: Diptera; family: Canacidae; genus: Procanace; specificEpithet: Procanacebifurcata; scientificNameAuthorship: Hardy & Delfinado, 1980; **Location:** islandGroup: Hawaiian Islands; island: Oahu; verbatimLocality: Opaeula Stream; minimumElevationInMeters: 1150; **Identification:** identifiedBy: DE Hardy & MD Delfinado; dateIdentified: 1980; **Event:** verbatimEventDate: 12.vi.1970; **Record Level:** institutionCode: BPBM
- **Type status:** Other material. **Occurrence:** catalogNumber: 2006005046; recordedBy: MD Delfinado; lifeStage: adult; **Taxon:** kingdom: Animalia; phylum: Arthropoda; class: Insecta; order: Diptera; family: Canacidae; genus: Procanace; specificEpithet: Procanacebifurcata; scientificNameAuthorship: Hardy & Delfinado, 1980; **Location:** islandGroup: Hawaiian Islands; island: Oahu; verbatimLocality: Opaeula Stream; minimumElevationInMeters: 1150; **Identification:** identifiedBy: DE Hardy & MD Delfinado; dateIdentified: 1980; **Event:** verbatimEventDate: 12.vi.1970; **Record Level:** institutionCode: BPBM
- **Type status:** Other material. **Occurrence:** catalogNumber: 2006005047; recordedBy: MD Delfinado; lifeStage: adult; **Taxon:** kingdom: Animalia; phylum: Arthropoda; class: Insecta; order: Diptera; family: Canacidae; genus: Procanace; specificEpithet: Procanacebifurcata; scientificNameAuthorship: Hardy & Delfinado, 1980; **Location:** islandGroup: Hawaiian Islands; island: Oahu; verbatimLocality: West Maui, Iao Valley; minimumElevationInMeters: 1150; **Identification:** identifiedBy: DE Hardy & MD Delfinado; dateIdentified: 1980; **Event:** verbatimEventDate: 12.vi.1970; **Record Level:** institutionCode: BPBM
- **Type status:** Other material. **Occurrence:** catalogNumber: 2006005040; recordedBy: MD Delfinado; lifeStage: adult; **Taxon:** kingdom: Animalia; phylum: Arthropoda; class: Insecta; order: Diptera; family: Canacidae; genus: Procanace; specificEpithet: Procanacebifurcata; scientificNameAuthorship: Hardy & Delfinado, 1980; **Location:** islandGroup: Hawaiian Islands; island: Oahu; verbatimLocality: Kawai-iki Stream; **Identification:** identifiedBy: DE Hardy & MD Delfinado; dateIdentified: 1980; **Event:** verbatimEventDate: 12.vi.1970; **Record Level:** institutionCode: BPBM
- **Type status:** Other material. **Occurrence:** recordedBy: DE Hardy; individualCount: 72; lifeStage: adult; **Taxon:** kingdom: Animalia; phylum: Arthropoda; class: Insecta; order: Diptera; family: Canacidae; genus: Procanace; specificEpithet: Procanacebifurcata; scientificNameAuthorship: Hardy & Delfinado, 1980; **Location:** islandGroup: Hawaiian Islands; island: Maui; verbatimLocality: Iao Stream, Iao Valley; verbatimElevation: 800 ft.; **Event:** verbatimEventDate: 2.ix.1970; **Record Level:** institutionCode: UHM
- **Type status:** Other material. **Occurrence:** recordedBy: DE Hardy; individualCount: 2; lifeStage: adult; **Taxon:** kingdom: Animalia; phylum: Arthropoda; class: Insecta; order: Diptera; family: Canacidae; genus: Procanace; specificEpithet: Procanacebifurcata; scientificNameAuthorship: Hardy & Delfinado, 1980; **Location:** islandGroup: Hawaiian Islands; island: Maui; verbatimLocality: Waikane Stream; **Event:** verbatimEventDate: 4.ix.1970; **Record Level:** institutionCode: UHM
- **Type status:** Other material. **Occurrence:** recordedBy: DE Hardy; individualCount: 28; lifeStage: adult; **Taxon:** kingdom: Animalia; phylum: Arthropoda; class: Insecta; order: Diptera; family: Canacidae; genus: Procanace; specificEpithet: Procanacebifurcata; scientificNameAuthorship: Hardy & Delfinado, 1980; **Location:** islandGroup: Hawaiian Islands; island: Maui; verbatimLocality: Kipahulu Valley; verbatimElevation: 500 ft.; **Event:** verbatimEventDate: 1.x.1970; **Record Level:** institutionCode: UHM
- **Type status:** Other material. **Occurrence:** recordedBy: DE Hardy; individualCount: 48; lifeStage: adult; **Taxon:** kingdom: Animalia; phylum: Arthropoda; class: Insecta; order: Diptera; family: Canacidae; genus: Procanace; specificEpithet: Procanacebifurcata; scientificNameAuthorship: Hardy & Delfinado, 1980; **Location:** islandGroup: Hawaiian Islands; island: Maui; verbatimLocality: Kinihapai Stream, Iao Valley; verbatimElevation: 500 ft.; **Event:** verbatimEventDate: 2.x.1970; **Record Level:** institutionCode: UHM
- **Type status:** Other material. **Occurrence:** recordedBy: DE Hardy; individualCount: 9; lifeStage: adult; **Taxon:** kingdom: Animalia; phylum: Arthropoda; class: Insecta; order: Diptera; family: Canacidae; genus: Procanace; specificEpithet: Procanacebifurcata; scientificNameAuthorship: Hardy & Delfinado, 1980; **Location:** islandGroup: Hawaiian Islands; island: Maui; verbatimLocality: Kopiliula Stream, Hana; verbatimElevation: 500 ft.; **Event:** verbatimEventDate: 2.x.1970; **Record Level:** institutionCode: UHM
- **Type status:** Other material. **Occurrence:** recordedBy: RA Englund; individualCount: 5; sex: females; lifeStage: adult; **Taxon:** kingdom: Animalia; phylum: Arthropoda; class: Insecta; order: Diptera; family: Canacidae; genus: Procanace; specificEpithet: Procanacebifurcata; scientificNameAuthorship: Hardy & Delfinado, 1980; **Location:** islandGroup: Hawaiian Islands; island: Kauai; verbatimLocality: Kauai, Hanakapai Stream, 5m elevation, 200m from ocean; **Identification:** identifiedBy: PM O'Grady; dateIdentified: i.2016; **Event:** eventDate: 2.i.2002; **Record Level:** institutionCode: BPBM

##### Ecological interactions

###### Native status

endemic

##### Distribution

**Figure 11.**
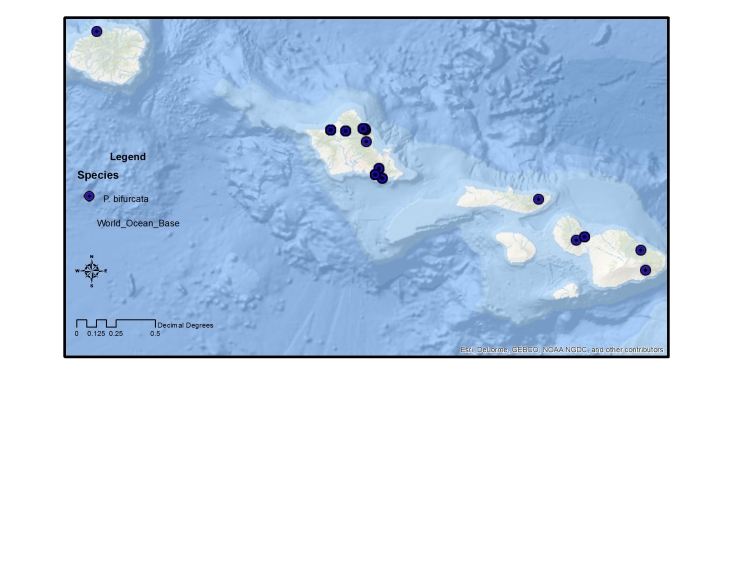
Distribution of *P. bifurcata* (Kauai, Oahu, Molokai and Maui).

HAWAIIAN ISLANDS: Kauai, Oahu, Molokai and Maui (Fig. 11​).

##### Notes

Hardy and Delfinado (1980), [original description; female terminalia (dorsal and ventral), spermathecae, ninth sternum, epandrium and surstylus]; Mathis (1992), [World Catalog]; Nishida (2002), [Hawaiian Arthropod Checklist].

#### Procanace confusa

Hardy and Delfinado, 1980

##### Materials

- **Type status:** Holotype. **Occurrence:** recordedBy: DE Hardy; individualCount: 1; sex: male; lifeStage: adult; **Taxon:** kingdom: Animalia; phylum: Arthropoda; class: Insecta; order: Diptera; family: Canacidae; genus: Procanace; specificEpithet: Procanaceconfusa; scientificNameAuthorship: Hardy & Delfinado, 1980; **Location:** islandGroup: Hawaiian Islands; island: Hawaii; verbatimLocality: Akaka Falls; **Identification:** identifiedBy: DE Hardy & MD Delfinado; dateIdentified: 1980; **Event:** verbatimEventDate: 19.vi.1964; **Record Level:** institutionCode: BPBM
- **Type status:** Paratype. **Occurrence:** recordedBy: M Tamashiro; lifeStage: adult; **Taxon:** kingdom: Animalia; phylum: Arthropoda; class: Insecta; order: Diptera; family: Canacidae; genus: Procanace; specificEpithet: Procanaceconfusa; scientificNameAuthorship: Hardy & Delfinado, 1980; **Location:** islandGroup: Hawaiian Islands; island: Maui; verbatimLocality: Waihee Stream; **Identification:** identifiedBy: DE Hardy & MD Delfinado; dateIdentified: 1980; **Event:** verbatimEventDate: v.1952
- **Type status:** Paratype. **Occurrence:** recordedBy: M Tamashiro; individualCount: 1; lifeStage: adult; **Taxon:** kingdom: Animalia; phylum: Arthropoda; class: Insecta; order: Diptera; family: Canacidae; genus: Procanace; specificEpithet: Procanaceconfusa; scientificNameAuthorship: Hardy & Delfinado, 1980; **Location:** islandGroup: Hawaiian Islands; island: Maui; verbatimLocality: Waihee Valley; **Identification:** identifiedBy: DE Hardy & MD Delfinado; dateIdentified: 1980; **Event:** verbatimEventDate: v.1952; **Record Level:** institutionCode: UHM
- **Type status:** Paratype. **Occurrence:** recordedBy: M Tamashiro; individualCount: 1; lifeStage: adult; **Taxon:** kingdom: Animalia; phylum: Arthropoda; class: Insecta; order: Diptera; family: Canacidae; genus: Procanace; specificEpithet: Procanaceconfusa; scientificNameAuthorship: Hardy & Delfinado, 1980; **Location:** islandGroup: Hawaiian Islands; island: Maui; verbatimLocality: Waihee Valley; **Identification:** identifiedBy: DE Hardy & MD Delfinado; dateIdentified: 1980; **Event:** verbatimEventDate: vi.1952; **Record Level:** institutionCode: UHM
- **Type status:** Paratype. **Occurrence:** recordedBy: DE Hardy, JA Tenorio; lifeStage: adult; **Taxon:** kingdom: Animalia; phylum: Arthropoda; class: Insecta; order: Diptera; family: Canacidae; genus: Procanace; specificEpithet: Procanaceconfusa; scientificNameAuthorship: Hardy & Delfinado, 1980; **Location:** islandGroup: Hawaiian Islands; island: Maui; verbatimLocality: Iao Valley, on wet rocks in swift running stream; **Identification:** identifiedBy: DE Hardy & MD Delfinado; dateIdentified: 1980; **Event:** verbatimEventDate: vi.1952
- **Type status:** Paratype. **Occurrence:** recordedBy: CR Joyce; sex: 1 female; lifeStage: adult; **Taxon:** kingdom: Animalia; phylum: Arthropoda; class: Insecta; order: Diptera; family: Canacidae; genus: Procanace; specificEpithet: Procanaceconfusa; scientificNameAuthorship: Hardy & Delfinado, 1980; **Location:** islandGroup: Hawaiian Islands; island: Maui; verbatimLocality: Waialua; **Identification:** identifiedBy: DE Hardy & MD Delfinado; dateIdentified: 1980; **Event:** verbatimEventDate: vi.1953; **Record Level:** institutionCode: USNM
- **Type status:** Paratype. **Occurrence:** recordedBy: DE Hardy; individualCount: 3; sex: 3 females; lifeStage: adult; **Taxon:** kingdom: Animalia; phylum: Arthropoda; class: Insecta; order: Diptera; family: Canacidae; genus: Procanace; specificEpithet: Procanaceconfusa; scientificNameAuthorship: Hardy & Delfinado, 1980; **Location:** islandGroup: Hawaiian Islands; island: Maui; verbatimLocality: Maui, Wailua; **Identification:** identifiedBy: DE Hardy & MD Delfinado; dateIdentified: 1980; **Event:** verbatimEventDate: vii.1953; **Record Level:** institutionCode: USNM
- **Type status:** Paratype. **Occurrence:** recordedBy: DE Hardy; individualCount: 29; lifeStage: adult; **Taxon:** kingdom: Animalia; phylum: Arthropoda; class: Insecta; order: Diptera; family: Canacidae; genus: Procanace; specificEpithet: Procanaceconfusa; scientificNameAuthorship: Hardy & Delfinado, 1980; **Location:** islandGroup: Hawaiian Islands; island: Hawaii; verbatimLocality: Akaka Falls; **Identification:** identifiedBy: DE Hardy & MD Delfinado; dateIdentified: 1980; **Event:** verbatimEventDate: 19.vi.1964; **Record Level:** institutionCode: UHM
- **Type status:** Paratype. **Occurrence:** recordedBy: DE Hardy; individualCount: 1; sex: female; lifeStage: adult; **Taxon:** kingdom: Animalia; phylum: Arthropoda; class: Insecta; order: Diptera; family: Canacidae; genus: Procanace; specificEpithet: Procanaceconfusa; scientificNameAuthorship: Hardy & Delfinado, 1980; **Location:** islandGroup: Hawaiian Islands; island: Hawaii; verbatimLocality: Akaka Falls; **Identification:** identifiedBy: DE Hardy & MD Delfinado; dateIdentified: 1980; **Event:** verbatimEventDate: 19.vi.1964; **Record Level:** institutionCode: BPBM
- **Type status:** Paratype. **Occurrence:** recordedBy: DE Hardy; lifeStage: adult; **Taxon:** kingdom: Animalia; phylum: Arthropoda; class: Insecta; order: Diptera; family: Canacidae; genus: Procanace; specificEpithet: Procanaceconfusa; scientificNameAuthorship: Hardy & Delfinado, 1980; **Location:** islandGroup: Hawaiian Islands; island: Hawaii; verbatimLocality: Akaka Falls; **Identification:** identifiedBy: DE Hardy & MD Delfinado; dateIdentified: 1980; **Event:** verbatimEventDate: 19.vi.1964
- **Type status:** Paratype. **Occurrence:** recordedBy: DE Hardy, JA Tenorio; sex: 1 female; lifeStage: adult; **Taxon:** kingdom: Animalia; phylum: Arthropoda; class: Insecta; order: Diptera; family: Canacidae; genus: Procanace; specificEpithet: Procanaceconfusa; scientificNameAuthorship: Hardy & Delfinado, 1980; **Location:** islandGroup: Hawaiian Islands; island: Maui; verbatimLocality: Iao Valley, on wet rocks in swift running stream; **Identification:** identifiedBy: DE Hardy & MD Delfinado; dateIdentified: 1980; **Event:** verbatimEventDate: 13.ix.1968; **Record Level:** institutionCode: USNM
- **Type status:** Paratype. **Occurrence:** recordedBy: DE Hardy; individualCount: 15; lifeStage: adult; **Taxon:** kingdom: Animalia; phylum: Arthropoda; class: Insecta; order: Diptera; family: Canacidae; genus: Procanace; specificEpithet: Procanaceconfusa; scientificNameAuthorship: Hardy & Delfinado, 1980; **Location:** islandGroup: Hawaiian Islands; island: Maui; verbatimLocality: Kipahulu Valley; **Identification:** identifiedBy: DE Hardy & MD Delfinado; dateIdentified: 1980; **Event:** verbatimEventDate: 21.ii.1970; **Record Level:** institutionCode: UHM
- **Type status:** Paratype. **Occurrence:** recordedBy: DE Hardy; lifeStage: adult; **Taxon:** kingdom: Animalia; phylum: Arthropoda; class: Insecta; order: Diptera; family: Canacidae; genus: Procanace; specificEpithet: Procanaceconfusa; scientificNameAuthorship: Hardy & Delfinado, 1980; **Location:** islandGroup: Hawaiian Islands; island: Maui; verbatimLocality: Kipahulu Valley, Seven Sacred Pools; **Identification:** identifiedBy: DE Hardy & MD Delfinado; dateIdentified: 1980; **Event:** verbatimEventDate: 21.ii.1970
- **Type status:** Paratype. **Occurrence:** recordedBy: JA Tenorio; individualCount: 2; sex: 2 females; lifeStage: adult; **Taxon:** kingdom: Animalia; phylum: Arthropoda; class: Insecta; order: Diptera; family: Canacidae; genus: Procanace; specificEpithet: Procanaceconfusa; scientificNameAuthorship: Hardy & Delfinado, 1980; **Location:** islandGroup: Hawaiian Islands; island: Maui; verbatimLocality: Waiakani Falls, roadside water dripping; **Identification:** identifiedBy: DE Hardy & MD Delfinado; dateIdentified: 1980; **Event:** verbatimEventDate: 26.iii.1970; **Record Level:** institutionCode: USNM
- **Type status:** Paratype. **Occurrence:** recordedBy: DE Hardy, JA Tenorio; sex: 1 male; lifeStage: adult; **Taxon:** kingdom: Animalia; phylum: Arthropoda; class: Insecta; order: Diptera; family: Canacidae; genus: Procanace; specificEpithet: Procanaceconfusa; scientificNameAuthorship: Hardy & Delfinado, 1980; **Location:** islandGroup: Hawaiian Islands; island: Maui; verbatimLocality: Iao Valley, on wet rocks in swift running stream; **Identification:** identifiedBy: DE Hardy & MD Delfinado; dateIdentified: 1980; **Event:** verbatimEventDate: 28-31.iii.1970; **Record Level:** institutionCode: USNM
- **Type status:** Paratype. **Occurrence:** recordedBy: DE Hardy; individualCount: 10; lifeStage: adult; **Taxon:** kingdom: Animalia; phylum: Arthropoda; class: Insecta; order: Diptera; family: Canacidae; genus: Procanace; specificEpithet: Procanaceconfusa; scientificNameAuthorship: Hardy & Delfinado, 1980; **Location:** islandGroup: Hawaiian Islands; island: Maui; verbatimLocality: Stream in Iao Valley; verbatimElevation: 1375 ft.; **Identification:** identifiedBy: DE Hardy & MD Delfinado; dateIdentified: 1980; **Event:** verbatimEventDate: 31.iii.1970; **Record Level:** institutionCode: UHM
- **Type status:** Paratype. **Occurrence:** recordedBy: JA Tenorio; individualCount: 1; lifeStage: adult; **Taxon:** kingdom: Animalia; phylum: Arthropoda; class: Insecta; order: Diptera; family: Canacidae; genus: Procanace; specificEpithet: Procanaceconfusa; scientificNameAuthorship: Hardy & Delfinado, 1980; **Location:** islandGroup: Hawaiian Islands; island: Hawaii; verbatimLocality: Kapue Stream; verbatimElevation: 750 ft.; **Identification:** identifiedBy: DE Hardy & MD Delfinado; dateIdentified: 1980; **Event:** verbatimEventDate: 29.v.1970; **Record Level:** institutionCode: UHM
- **Type status:** Paratype. **Occurrence:** recordedBy: MD Delfinado; individualCount: 2; lifeStage: adult; **Taxon:** kingdom: Animalia; phylum: Arthropoda; class: Insecta; order: Diptera; family: Canacidae; genus: Procanace; specificEpithet: Procanaceconfusa; scientificNameAuthorship: Hardy & Delfinado, 1980; **Location:** islandGroup: Hawaiian Islands; island: Hawaii; verbatimLocality: Kapue Stream; verbatimElevation: 1000 ft.; **Identification:** identifiedBy: DE Hardy & MD Delfinado; dateIdentified: 1980; **Event:** verbatimEventDate: 29.v.1970; **Record Level:** institutionCode: UHM
- **Type status:** Paratype. **Occurrence:** recordedBy: MD Delfinado; individualCount: 5; lifeStage: adult; **Taxon:** kingdom: Animalia; phylum: Arthropoda; class: Insecta; order: Diptera; family: Canacidae; genus: Procanace; specificEpithet: Procanaceconfusa; scientificNameAuthorship: Hardy & Delfinado, 1980; **Location:** islandGroup: Hawaiian Islands; island: Hawaii; verbatimLocality: Kaieie Stream; verbatimElevation: 1000 ft.; **Identification:** identifiedBy: DE Hardy & MD Delfinado; dateIdentified: 1980; **Event:** verbatimEventDate: 29.v.1970; **Record Level:** institutionCode: UHM
- **Type status:** Paratype. **Occurrence:** recordedBy: MD Delfinado; individualCount: 29; lifeStage: adult; **Taxon:** kingdom: Animalia; phylum: Arthropoda; class: Insecta; order: Diptera; family: Canacidae; genus: Procanace; specificEpithet: Procanaceconfusa; scientificNameAuthorship: Hardy & Delfinado, 1980; **Location:** islandGroup: Hawaiian Islands; island: Hawaii; verbatimLocality: Pahoehoe Stream; **Identification:** identifiedBy: DE Hardy & MD Delfinado; dateIdentified: 1980; **Event:** verbatimEventDate: 29.v.1970; **Record Level:** institutionCode: UHM
- **Type status:** Paratype. **Occurrence:** recordedBy: MD Delfinado, JA Tenorio; lifeStage: adult; **Taxon:** kingdom: Animalia; phylum: Arthropoda; class: Insecta; order: Diptera; family: Canacidae; genus: Procanace; specificEpithet: Procanaceconfusa; scientificNameAuthorship: Hardy & Delfinado, 1980; **Location:** islandGroup: Hawaiian Islands; island: Hawaii; verbatimLocality: Pahoehoe Stream; **Identification:** identifiedBy: DE Hardy & MD Delfinado; dateIdentified: 1980; **Event:** verbatimEventDate: 29.v.1970
- **Type status:** Paratype. **Occurrence:** recordedBy: MD Delfinado, JA Tenorio; lifeStage: adult; **Taxon:** kingdom: Animalia; phylum: Arthropoda; class: Insecta; order: Diptera; family: Canacidae; genus: Procanace; specificEpithet: Procanaceconfusa; scientificNameAuthorship: Hardy & Delfinado, 1980; **Location:** islandGroup: Hawaiian Islands; island: Hawaii; verbatimLocality: Kapue Stream; **Identification:** identifiedBy: DE Hardy & MD Delfinado; dateIdentified: 1980; **Event:** verbatimEventDate: 29.v.1970
- **Type status:** Paratype. **Occurrence:** recordedBy: JA Tenorio; individualCount: 1; sex: 1 female; lifeStage: adult; **Taxon:** kingdom: Animalia; phylum: Arthropoda; class: Insecta; order: Diptera; family: Canacidae; genus: Procanace; specificEpithet: Procanaceconfusa; scientificNameAuthorship: Hardy & Delfinado, 1980; **Location:** islandGroup: Hawaiian Islands; island: Hawaii; verbatimLocality: Kapue Stream, on rocks in stream; verbatimElevation: 750 ft.; **Identification:** identifiedBy: DE Hardy & MD Delfinado; dateIdentified: 1980; **Event:** verbatimEventDate: 29.v.1970; **Record Level:** institutionCode: USNM
- **Type status:** Paratype. **Occurrence:** recordedBy: JA Tenorio; individualCount: 2; lifeStage: adult; **Taxon:** kingdom: Animalia; phylum: Arthropoda; class: Insecta; order: Diptera; family: Canacidae; genus: Procanace; specificEpithet: Procanaceconfusa; scientificNameAuthorship: Hardy & Delfinado, 1980; **Location:** islandGroup: Hawaiian Islands; island: Hawaii; verbatimLocality: Wailuku River; verbatimElevation: 500 ft.; **Identification:** identifiedBy: DE Hardy & MD Delfinado; dateIdentified: 1980; **Event:** verbatimEventDate: 23.vi.1970; **Record Level:** institutionCode: UHM
- **Type status:** Paratype. **Occurrence:** recordedBy: DE Hardy, MD Delfinado, JA Tenorio; lifeStage: adult; **Taxon:** kingdom: Animalia; phylum: Arthropoda; class: Insecta; order: Diptera; family: Canacidae; genus: Procanace; specificEpithet: Procanaceconfusa; scientificNameAuthorship: Hardy & Delfinado, 1980; **Location:** islandGroup: Hawaiian Islands; island: Maui; verbatimLocality: Kopiliula Stream; **Identification:** identifiedBy: DE Hardy & MD Delfinado; dateIdentified: 1980; **Event:** verbatimEventDate: viii.1970
- **Type status:** Paratype. **Occurrence:** recordedBy: MD Delfinado, JA Tenorio; lifeStage: adult; **Taxon:** kingdom: Animalia; phylum: Arthropoda; class: Insecta; order: Diptera; family: Canacidae; genus: Procanace; specificEpithet: Procanaceconfusa; scientificNameAuthorship: Hardy & Delfinado, 1980; **Location:** islandGroup: Hawaiian Islands; island: Hawaii; verbatimLocality: Wailuku River; **Identification:** identifiedBy: DE Hardy & MD Delfinado; dateIdentified: 1980; **Event:** verbatimEventDate: iii-vii.1971
- **Type status:** Other material. **Occurrence:** catalogNumber: 2006005065; recordedBy: M Tamachiro; sex: male; lifeStage: adult; **Taxon:** kingdom: Animalia; phylum: Arthropoda; class: Insecta; order: Diptera; family: Canacidae; genus: Procanace; specificEpithet: Procanaceconfusa; scientificNameAuthorship: Hardy & Delfinado, 1980; **Location:** islandGroup: Hawaiian Islands; island: Maui; verbatimLocality: Waihee; **Identification:** identifiedBy: DE Hardy & MD Delfinado; dateIdentified: 1980; **Event:** verbatimEventDate: v.1952; **Record Level:** institutionCode: BPBM
- **Type status:** Other material. **Occurrence:** catalogNumber: 2006005066; recordedBy: DE Hardy; lifeStage: adult; **Taxon:** kingdom: Animalia; phylum: Arthropoda; class: Insecta; order: Diptera; family: Canacidae; genus: Procanace; specificEpithet: Procanaceconfusa; scientificNameAuthorship: Hardy & Delfinado, 1980; **Location:** islandGroup: Hawaiian Islands; island: Maui; verbatimLocality: West Maui, Iao Valley; **Identification:** identifiedBy: DE Hardy & MD Delfinado; dateIdentified: 1980; **Event:** verbatimEventDate: vi.1952; **Record Level:** institutionCode: BPBM
- **Type status:** Other material. **Occurrence:** catalogNumber: 2006005052; recordedBy: CR Joyce; sex: male; lifeStage: adult; **Taxon:** kingdom: Animalia; phylum: Arthropoda; class: Insecta; order: Diptera; family: Canacidae; genus: Procanace; specificEpithet: Procanaceconfusa; scientificNameAuthorship: Hardy & Delfinado, 1980; **Location:** islandGroup: Hawaiian Islands; island: Maui; verbatimLocality: Wailua; **Identification:** identifiedBy: DE Hardy & MD Delfinado; dateIdentified: 1980; **Event:** verbatimEventDate: June 1953; **Record Level:** institutionCode: BPBM
- **Type status:** Other material. **Occurrence:** catalogNumber: 2006005053; recordedBy: CR Joyce; lifeStage: adult; **Taxon:** kingdom: Animalia; phylum: Arthropoda; class: Insecta; order: Diptera; family: Canacidae; genus: Procanace; specificEpithet: Procanaceconfusa; scientificNameAuthorship: Hardy & Delfinado, 1980; **Location:** islandGroup: Hawaiian Islands; island: Maui; verbatimLocality: Wailua; **Identification:** identifiedBy: DE Hardy & MD Delfinado; dateIdentified: 1980; **Event:** verbatimEventDate: June 1953; **Record Level:** institutionCode: BPBM
- **Type status:** Other material. **Occurrence:** catalogNumber: 2006005054; recordedBy: CR Joyce; lifeStage: adult; **Taxon:** kingdom: Animalia; phylum: Arthropoda; class: Insecta; order: Diptera; family: Canacidae; genus: Procanace; specificEpithet: Procanaceconfusa; scientificNameAuthorship: Hardy & Delfinado, 1980; **Location:** islandGroup: Hawaiian Islands; island: Maui; verbatimLocality: Wailua; **Identification:** identifiedBy: DE Hardy & MD Delfinado; dateIdentified: 1980; **Event:** verbatimEventDate: June 1953; **Record Level:** institutionCode: BPBM
- **Type status:** Other material. **Occurrence:** catalogNumber: 2006005055; recordedBy: CR Joyce; lifeStage: adult; **Taxon:** kingdom: Animalia; phylum: Arthropoda; class: Insecta; order: Diptera; family: Canacidae; genus: Procanace; specificEpithet: Procanaceconfusa; scientificNameAuthorship: Hardy & Delfinado, 1980; **Location:** islandGroup: Hawaiian Islands; island: Maui; verbatimLocality: Wailua; **Identification:** identifiedBy: DE Hardy & MD Delfinado; dateIdentified: 1980; **Event:** verbatimEventDate: vi.1953; **Record Level:** institutionCode: BPBM
- **Type status:** Other material. **Occurrence:** catalogNumber: 2006005067; recordedBy: CR Joyce; lifeStage: adult; **Taxon:** kingdom: Animalia; phylum: Arthropoda; class: Insecta; order: Diptera; family: Canacidae; genus: Procanace; specificEpithet: Procanaceconfusa; scientificNameAuthorship: Hardy & Delfinado, 1980; **Location:** islandGroup: Hawaiian Islands; island: Maui; verbatimLocality: Keanae; **Identification:** identifiedBy: DE Hardy & MD Delfinado; dateIdentified: 1980; **Event:** verbatimEventDate: vii.1953; **Record Level:** institutionCode: BPBM
- **Type status:** Other material. **Occurrence:** catalogNumber: 2006005070; recordedBy: DE Hardy; lifeStage: adult; **Taxon:** kingdom: Animalia; phylum: Arthropoda; class: Insecta; order: Diptera; family: Canacidae; genus: Procanace; specificEpithet: Procanaceconfusa; scientificNameAuthorship: Hardy & Delfinado, 1980; **Location:** islandGroup: Hawaiian Islands; island: Maui; verbatimLocality: West Maui, Iao Valley; minimumElevationInMeters: 1375; **Identification:** identifiedBy: DE Hardy & MD Delfinado; dateIdentified: 1980; **Event:** verbatimEventDate: 21.iii.1970; **Record Level:** institutionCode: BPBM
- **Type status:** Other material. **Occurrence:** catalogNumber: 2006005071; recordedBy: DE Hardy; lifeStage: adult; **Taxon:** kingdom: Animalia; phylum: Arthropoda; class: Insecta; order: Diptera; family: Canacidae; genus: Procanace; specificEpithet: Procanaceconfusa; scientificNameAuthorship: Hardy & Delfinado, 1980; **Location:** islandGroup: Hawaiian Islands; island: Maui; verbatimLocality: West Maui, Iao Valley; minimumElevationInMeters: 1375; **Identification:** identifiedBy: DE Hardy & MD Delfinado; dateIdentified: 1980; **Event:** verbatimEventDate: 21.iii.1970; **Record Level:** institutionCode: BPBM
- **Type status:** Other material. **Occurrence:** catalogNumber: 2006005068; recordedBy: JA Tenorio; lifeStage: adult; **Taxon:** kingdom: Animalia; phylum: Arthropoda; class: Insecta; order: Diptera; family: Canacidae; genus: Procanace; specificEpithet: Procanaceconfusa; scientificNameAuthorship: Hardy & Delfinado, 1980; **Location:** islandGroup: Hawaiian Islands; island: Maui; verbatimLocality: West Maui, Iao Valley, on stream; **Identification:** identifiedBy: DE Hardy & MD Delfinado; dateIdentified: 1980; **Event:** verbatimEventDate: 28.iii.1970; **Record Level:** institutionCode: BPBM
- **Type status:** Other material. **Occurrence:** catalogNumber: 2006005069; recordedBy: JA Tenorio; lifeStage: adult; **Taxon:** kingdom: Animalia; phylum: Arthropoda; class: Insecta; order: Diptera; family: Canacidae; genus: Procanace; specificEpithet: Procanaceconfusa; scientificNameAuthorship: Hardy & Delfinado, 1980; **Location:** islandGroup: Hawaiian Islands; island: Maui; verbatimLocality: West Maui, Iao Valley, on stream; **Identification:** identifiedBy: DE Hardy & MD Delfinado; dateIdentified: 1980; **Event:** verbatimEventDate: 28.iii.1970; **Record Level:** institutionCode: BPBM
- **Type status:** Other material. **Occurrence:** catalogNumber: 2006005061; recordedBy: JA Tenorio; sex: female; lifeStage: adult; **Taxon:** kingdom: Animalia; phylum: Arthropoda; class: Insecta; order: Diptera; family: Canacidae; genus: Procanace; specificEpithet: Procanaceconfusa; scientificNameAuthorship: Hardy & Delfinado, 1980; **Location:** islandGroup: Hawaiian Islands; island: Hawaii; verbatimLocality: Pahoehoe Stream; **Identification:** identifiedBy: DE Hardy & MD Delfinado; dateIdentified: 1980; **Event:** verbatimEventDate: 28.v.1970; **Record Level:** institutionCode: BPBM
- **Type status:** Other material. **Occurrence:** catalogNumber: 2006005062; recordedBy: JA Tenorio; sex: female; lifeStage: adult; **Taxon:** kingdom: Animalia; phylum: Arthropoda; class: Insecta; order: Diptera; family: Canacidae; genus: Procanace; specificEpithet: Procanaceconfusa; scientificNameAuthorship: Hardy & Delfinado, 1980; **Location:** islandGroup: Hawaiian Islands; island: Hawaii; verbatimLocality: Pahoehoe Stream; **Identification:** identifiedBy: DE Hardy & MD Delfinado; dateIdentified: 1980; **Event:** verbatimEventDate: 28.v.1970; **Record Level:** institutionCode: BPBM
- **Type status:** Other material. **Occurrence:** catalogNumber: 2006005064; recordedBy: JA Tenorio; sex: male; lifeStage: adult; **Taxon:** kingdom: Animalia; phylum: Arthropoda; class: Insecta; order: Diptera; family: Canacidae; genus: Procanace; specificEpithet: Procanaceconfusa; scientificNameAuthorship: Hardy & Delfinado, 1980; **Location:** islandGroup: Hawaiian Islands; island: Hawaii; verbatimLocality: Pahoehoe Stream; **Identification:** identifiedBy: DE Hardy & MD Delfinado; dateIdentified: 1980; **Event:** verbatimEventDate: 28.v.1970; **Record Level:** institutionCode: BPBM
- **Type status:** Other material. **Occurrence:** catalogNumber: 2006005063; recordedBy: JA Tenorio; sex: male; lifeStage: adult; **Taxon:** kingdom: Animalia; phylum: Arthropoda; class: Insecta; order: Diptera; family: Canacidae; genus: Procanace; specificEpithet: Procanaceconfusa; scientificNameAuthorship: Hardy & Delfinado, 1980; **Location:** islandGroup: Hawaiian Islands; island: Hawaii; verbatimLocality: Pahoehoe Stream; **Identification:** identifiedBy: DE Hardy & MD Delfinado; dateIdentified: 1980; **Event:** verbatimEventDate: 28.v.1970; **Record Level:** institutionCode: BPBM
- **Type status:** Other material. **Occurrence:** catalogNumber: 2006005058; recordedBy: JA Tenorio; sex: male; lifeStage: adult; **Taxon:** kingdom: Animalia; phylum: Arthropoda; class: Insecta; order: Diptera; family: Canacidae; genus: Procanace; specificEpithet: Procanaceconfusa; scientificNameAuthorship: Hardy & Delfinado, 1980; **Location:** islandGroup: Hawaiian Islands; island: Hawaii; verbatimLocality: Kapue Stream; minimumElevationInMeters: 750; **Identification:** identifiedBy: DE Hardy & MD Delfinado; dateIdentified: 1980; **Event:** verbatimEventDate: 29.v.1970; **Record Level:** institutionCode: BPBM
- **Type status:** Other material. **Occurrence:** catalogNumber: 2006005059; recordedBy: JA Tenorio; sex: female; lifeStage: adult; **Taxon:** kingdom: Animalia; phylum: Arthropoda; class: Insecta; order: Diptera; family: Canacidae; genus: Procanace; specificEpithet: Procanaceconfusa; scientificNameAuthorship: Hardy & Delfinado, 1980; **Location:** islandGroup: Hawaiian Islands; island: Hawaii; verbatimLocality: Kapue Stream; minimumElevationInMeters: 750; **Identification:** identifiedBy: DE Hardy & MD Delfinado; dateIdentified: 1980; **Event:** verbatimEventDate: 29.v.1970; **Record Level:** institutionCode: BPBM
- **Type status:** Other material. **Occurrence:** catalogNumber: 2006005060; recordedBy: JA Tenorio; sex: female; lifeStage: adult; **Taxon:** kingdom: Animalia; phylum: Arthropoda; class: Insecta; order: Diptera; family: Canacidae; genus: Procanace; specificEpithet: Procanaceconfusa; scientificNameAuthorship: Hardy & Delfinado, 1980; **Location:** islandGroup: Hawaiian Islands; island: Hawaii; verbatimLocality: Kaieie Stream; minimumElevationInMeters: 1000; **Identification:** identifiedBy: DE Hardy & MD Delfinado; dateIdentified: 1980; **Event:** verbatimEventDate: 29.v.1970; **Record Level:** institutionCode: BPBM
- **Type status:** Other material. **Occurrence:** catalogNumber: 2006005057; recordedBy: JA Tenorio; sex: female; lifeStage: adult; **Taxon:** kingdom: Animalia; phylum: Arthropoda; class: Insecta; order: Diptera; family: Canacidae; genus: Procanace; specificEpithet: Procanaceconfusa; scientificNameAuthorship: Hardy & Delfinado, 1980; **Location:** islandGroup: Hawaiian Islands; island: Hawaii; verbatimLocality: Kapue Stream; minimumElevationInMeters: 750; **Identification:** identifiedBy: DE Hardy & MD Delfinado; dateIdentified: 1980; **Event:** verbatimEventDate: 29.v.1970; **Record Level:** institutionCode: BPBM
- **Type status:** Other material. **Occurrence:** catalogNumber: 2006005056; recordedBy: JA Tenorio; sex: male; lifeStage: adult; **Taxon:** kingdom: Animalia; phylum: Arthropoda; class: Insecta; order: Diptera; family: Canacidae; genus: Procanace; specificEpithet: Procanaceconfusa; scientificNameAuthorship: Hardy & Delfinado, 1980; **Location:** islandGroup: Hawaiian Islands; island: Maui; verbatimLocality: Kapue Stream; minimumElevationInMeters: 750; **Identification:** identifiedBy: DE Hardy & MD Delfinado; dateIdentified: 1980; **Event:** verbatimEventDate: 29.v.1970; **Record Level:** institutionCode: BPBM
- **Type status:** Other material. **Occurrence:** catalogNumber: 2006005114; recordedBy: GM Nishida; lifeStage: adult; **Taxon:** kingdom: Animalia; phylum: Arthropoda; class: Insecta; order: Diptera; family: Canacidae; genus: Procanace; specificEpithet: Procanaceconfusa; scientificNameAuthorship: Hardy & Delfinado, 1980; **Location:** islandGroup: Hawaiian Islands; island: Maui; verbatimLocality: East Maui, Hahalawe Gulch; minimumElevationInMeters: 1197; maximumElevationInMeters: 1345; **Event:** verbatimEventDate: 05.v.1984; **Record Level:** institutionCode: BPBM
- **Type status:** Other material. **Occurrence:** catalogNumber: 2006005108; recordedBy: GM Nishida; lifeStage: adult; **Taxon:** kingdom: Animalia; phylum: Arthropoda; class: Insecta; order: Diptera; family: Canacidae; genus: Procanace; specificEpithet: Procanaceconfusa; scientificNameAuthorship: Hardy & Delfinado, 1980; **Location:** islandGroup: Hawaiian Islands; island: Maui; verbatimLocality: East Maui, Hahalawe Gulch; minimumElevationInMeters: 1197; maximumElevationInMeters: 1345; **Event:** verbatimEventDate: 05.v.1984; **Record Level:** institutionCode: BPBM
- **Type status:** Other material. **Occurrence:** catalogNumber: 2006005109; recordedBy: GM Nishida; lifeStage: adult; **Taxon:** kingdom: Animalia; phylum: Arthropoda; class: Insecta; order: Diptera; family: Canacidae; genus: Procanace; specificEpithet: Procanaceconfusa; scientificNameAuthorship: Hardy & Delfinado, 1980; **Location:** islandGroup: Hawaiian Islands; island: Maui; verbatimLocality: East Maui, Hahalawe Gulch; minimumElevationInMeters: 1197; maximumElevationInMeters: 1345; **Event:** verbatimEventDate: 05.v.1984; **Record Level:** institutionCode: BPBM
- **Type status:** Other material. **Occurrence:** catalogNumber: 2006005110; recordedBy: GM Nishida; lifeStage: adult; **Taxon:** kingdom: Animalia; phylum: Arthropoda; class: Insecta; order: Diptera; family: Canacidae; genus: Procanace; specificEpithet: Procanaceconfusa; scientificNameAuthorship: Hardy & Delfinado, 1980; **Location:** islandGroup: Hawaiian Islands; island: Maui; verbatimLocality: East Maui, Hahalawe Gulch; minimumElevationInMeters: 1197; maximumElevationInMeters: 1345; **Event:** verbatimEventDate: 05.v.1984; **Record Level:** institutionCode: BPBM
- **Type status:** Other material. **Occurrence:** catalogNumber: 2006005111; recordedBy: GM Nishida; lifeStage: adult; **Taxon:** kingdom: Animalia; phylum: Arthropoda; class: Insecta; order: Diptera; family: Canacidae; genus: Procanace; specificEpithet: Procanaceconfusa; scientificNameAuthorship: Hardy & Delfinado, 1980; **Location:** islandGroup: Hawaiian Islands; island: Maui; verbatimLocality: East Maui, Hahalawe Gulch; minimumElevationInMeters: 1197; maximumElevationInMeters: 1345; **Event:** verbatimEventDate: 05.v.1984; **Record Level:** institutionCode: BPBM
- **Type status:** Other material. **Occurrence:** catalogNumber: 2006005113; recordedBy: GM Nishida; lifeStage: adult; **Taxon:** kingdom: Animalia; phylum: Arthropoda; class: Insecta; order: Diptera; family: Canacidae; genus: Procanace; specificEpithet: Procanaceconfusa; scientificNameAuthorship: Hardy & Delfinado, 1980; **Location:** islandGroup: Hawaiian Islands; island: Maui; verbatimLocality: East Maui, Hahalawe Gulch; minimumElevationInMeters: 1197; maximumElevationInMeters: 1345; **Event:** verbatimEventDate: 05.v.1984; **Record Level:** institutionCode: BPBM
- **Type status:** Other material. **Occurrence:** catalogNumber: 2006005115; recordedBy: GM Nishida; lifeStage: adult; **Taxon:** kingdom: Animalia; phylum: Arthropoda; class: Insecta; order: Diptera; family: Canacidae; genus: Procanace; specificEpithet: Procanaceconfusa; scientificNameAuthorship: Hardy & Delfinado, 1980; **Location:** islandGroup: Hawaiian Islands; island: Maui; verbatimLocality: East Maui, Hahalawe Gulch; minimumElevationInMeters: 1197; maximumElevationInMeters: 1345; **Event:** verbatimEventDate: 05.v.1984; **Record Level:** institutionCode: BPBM
- **Type status:** Other material. **Occurrence:** catalogNumber: 2006005117; recordedBy: GM Nishida; lifeStage: adult; **Taxon:** kingdom: Animalia; phylum: Arthropoda; class: Insecta; order: Diptera; family: Canacidae; genus: Procanace; specificEpithet: Procanaceconfusa; scientificNameAuthorship: Hardy & Delfinado, 1980; **Location:** islandGroup: Hawaiian Islands; island: Maui; verbatimLocality: East Maui, Hahalawe Gulch; minimumElevationInMeters: 1197; maximumElevationInMeters: 1345; **Event:** verbatimEventDate: 05.v.1984; **Record Level:** institutionCode: BPBM
- **Type status:** Other material. **Occurrence:** catalogNumber: 2006005118; recordedBy: GM Nishida; lifeStage: adult; **Taxon:** kingdom: Animalia; phylum: Arthropoda; class: Insecta; order: Diptera; family: Canacidae; genus: Procanace; specificEpithet: Procanaceconfusa; scientificNameAuthorship: Hardy & Delfinado, 1980; **Location:** islandGroup: Hawaiian Islands; island: Maui; verbatimLocality: East Maui, Hahalawe Gulch; minimumElevationInMeters: 1197; maximumElevationInMeters: 1345; **Event:** verbatimEventDate: 05.v.1984; **Record Level:** institutionCode: BPBM
- **Type status:** Other material. **Occurrence:** catalogNumber: 2006005116; recordedBy: GM Nishida; lifeStage: adult; **Taxon:** kingdom: Animalia; phylum: Arthropoda; class: Insecta; order: Diptera; family: Canacidae; genus: Procanace; specificEpithet: Procanaceconfusa; scientificNameAuthorship: Hardy & Delfinado, 1980; **Location:** islandGroup: Hawaiian Islands; island: Maui; verbatimLocality: East Maui, Hahalawe Gulch; minimumElevationInMeters: 1197; maximumElevationInMeters: 1345; **Event:** verbatimEventDate: 05.v.1984; **Record Level:** institutionCode: BPBM
- **Type status:** Other material. **Occurrence:** catalogNumber: 2006005107; recordedBy: DA Polhemus; lifeStage: adult; **Taxon:** kingdom: Animalia; phylum: Arthropoda; class: Insecta; order: Diptera; family: Canacidae; genus: Procanace; specificEpithet: Procanaceconfusa; scientificNameAuthorship: Hardy & Delfinado, 1980; **Location:** islandGroup: Hawaiian Islands; island: Hawaii; verbatimLocality: Hilo, NW of Honolii Stream; minimumElevationInMeters: 1312; **Identification:** identifiedBy: WN Mathis; dateIdentified: 1992; **Event:** verbatimEventDate: 26.vii.1990; **Record Level:** institutionCode: BPBM
- **Type status:** Other material. **Occurrence:** catalogNumber: 2006005106; recordedBy: DA Polhemus; lifeStage: adult; **Taxon:** kingdom: Animalia; phylum: Arthropoda; class: Insecta; order: Diptera; family: Canacidae; genus: Procanace; specificEpithet: Procanaceconfusa; scientificNameAuthorship: Hardy & Delfinado, 1980; **Location:** islandGroup: Hawaiian Islands; island: Hawaii; verbatimLocality: Hilo, NW of Honolii Stream; minimumElevationInMeters: 1312; **Identification:** identifiedBy: WN Mathis; dateIdentified: 1992; **Event:** verbatimEventDate: 26.vii.1990; **Record Level:** institutionCode: BPBM
- **Type status:** Other material. **Occurrence:** catalogNumber: 2006005098; recordedBy: DA Polhemus; lifeStage: adult; **Taxon:** kingdom: Animalia; phylum: Arthropoda; class: Insecta; order: Diptera; family: Canacidae; genus: Procanace; specificEpithet: Procanaceconfusa; scientificNameAuthorship: Hardy & Delfinado, 1980; **Location:** islandGroup: Hawaiian Islands; island: Hawaii; verbatimLocality: Opaeula Stream; minimumElevationInMeters: 1312; **Identification:** identifiedBy: WN Mathis; dateIdentified: 1992; **Event:** verbatimEventDate: 26.vii.1990; **Record Level:** institutionCode: BPBM
- **Type status:** Other material. **Occurrence:** catalogNumber: 2006005091; recordedBy: DA Polhemus; lifeStage: adult; **Taxon:** kingdom: Animalia; phylum: Arthropoda; class: Insecta; order: Diptera; family: Canacidae; genus: Procanace; specificEpithet: Procanaceconfusa; scientificNameAuthorship: Hardy & Delfinado, 1980; **Location:** islandGroup: Hawaiian Islands; island: Hawaii; verbatimLocality: Hilo, NW of Honolii Stream; minimumElevationInMeters: 1312; **Identification:** identifiedBy: WN Mathis; dateIdentified: 1992; **Event:** verbatimEventDate: 26.vii.1990; **Record Level:** institutionCode: BPBM
- **Type status:** Other material. **Occurrence:** catalogNumber: 2006005092; recordedBy: DA Polhemus; lifeStage: adult; **Taxon:** kingdom: Animalia; phylum: Arthropoda; class: Insecta; order: Diptera; family: Canacidae; genus: Procanace; specificEpithet: Procanaceconfusa; scientificNameAuthorship: Hardy & Delfinado, 1980; **Location:** islandGroup: Hawaiian Islands; island: Hawaii; verbatimLocality: Hilo, NW of Honolii Stream; minimumElevationInMeters: 1312; **Identification:** identifiedBy: WN Mathis; dateIdentified: 1992; **Event:** verbatimEventDate: 26.vii.1990; **Record Level:** institutionCode: BPBM
- **Type status:** Other material. **Occurrence:** catalogNumber: 2006005093; recordedBy: DA Polhemus; lifeStage: adult; **Taxon:** kingdom: Animalia; phylum: Arthropoda; class: Insecta; order: Diptera; family: Canacidae; genus: Procanace; specificEpithet: Procanaceconfusa; scientificNameAuthorship: Hardy & Delfinado, 1980; **Location:** islandGroup: Hawaiian Islands; island: Hawaii; verbatimLocality: Hilo, NW of Honolii Stream; minimumElevationInMeters: 1312; **Identification:** identifiedBy: WN Mathis; dateIdentified: 1992; **Event:** verbatimEventDate: 26.vii.1990; **Record Level:** institutionCode: BPBM
- **Type status:** Other material. **Occurrence:** catalogNumber: 2006005094; recordedBy: DA Polhemus; lifeStage: adult; **Taxon:** kingdom: Animalia; phylum: Arthropoda; class: Insecta; order: Diptera; family: Canacidae; genus: Procanace; specificEpithet: Procanaceconfusa; scientificNameAuthorship: Hardy & Delfinado, 1980; **Location:** islandGroup: Hawaiian Islands; island: Hawaii; verbatimLocality: Hilo, NW of Honolii Stream; minimumElevationInMeters: 1312; **Identification:** identifiedBy: WN Mathis; dateIdentified: 1992; **Event:** verbatimEventDate: 26.vii.1990; **Record Level:** institutionCode: BPBM
- **Type status:** Other material. **Occurrence:** catalogNumber: 2006005095; recordedBy: DA Polhemus; lifeStage: adult; **Taxon:** kingdom: Animalia; phylum: Arthropoda; class: Insecta; order: Diptera; family: Canacidae; genus: Procanace; specificEpithet: Procanaceconfusa; scientificNameAuthorship: Hardy & Delfinado, 1980; **Location:** islandGroup: Hawaiian Islands; island: Hawaii; verbatimLocality: Hilo, NW of Honolii Stream; minimumElevationInMeters: 1312; **Identification:** identifiedBy: WN Mathis; dateIdentified: 1992; **Event:** verbatimEventDate: 26.vii.1990; **Record Level:** institutionCode: BPBM
- **Type status:** Other material. **Occurrence:** catalogNumber: 2006005097; recordedBy: DA Polhemus; lifeStage: adult; **Taxon:** kingdom: Animalia; phylum: Arthropoda; class: Insecta; order: Diptera; family: Canacidae; genus: Procanace; specificEpithet: Procanaceconfusa; scientificNameAuthorship: Hardy & Delfinado, 1980; **Location:** islandGroup: Hawaiian Islands; island: Hawaii; verbatimLocality: Hilo, NW of Honolii Stream; minimumElevationInMeters: 1312; **Identification:** identifiedBy: WN Mathis; dateIdentified: 1992; **Event:** verbatimEventDate: 26.vii.1990; **Record Level:** institutionCode: BPBM
- **Type status:** Other material. **Occurrence:** catalogNumber: 2006005088; recordedBy: DA Polhemus; lifeStage: adult; **Taxon:** kingdom: Animalia; phylum: Arthropoda; class: Insecta; order: Diptera; family: Canacidae; genus: Procanace; specificEpithet: Procanaceconfusa; scientificNameAuthorship: Hardy & Delfinado, 1980; **Location:** islandGroup: Hawaiian Islands; island: Hawaii; verbatimLocality: Hilo, NW of Honolii Stream; minimumElevationInMeters: 1312; **Identification:** identifiedBy: WN Mathis; dateIdentified: 1992; **Event:** verbatimEventDate: 26.vii.1990; **Record Level:** institutionCode: BPBM
- **Type status:** Other material. **Occurrence:** catalogNumber: 2006005099; recordedBy: DA Polhemus; lifeStage: adult; **Taxon:** kingdom: Animalia; phylum: Arthropoda; class: Insecta; order: Diptera; family: Canacidae; genus: Procanace; specificEpithet: Procanaceconfusa; scientificNameAuthorship: Hardy & Delfinado, 1980; **Location:** islandGroup: Hawaiian Islands; island: Hawaii; verbatimLocality: Hilo, NW of Honolii Stream; minimumElevationInMeters: 1312; **Identification:** identifiedBy: WN Mathis; dateIdentified: 1992; **Event:** verbatimEventDate: 26.vii.1990; **Record Level:** institutionCode: BPBM
- **Type status:** Other material. **Occurrence:** catalogNumber: 2006005100; recordedBy: DA Polhemus; lifeStage: adult; **Taxon:** kingdom: Animalia; phylum: Arthropoda; class: Insecta; order: Diptera; family: Canacidae; genus: Procanace; specificEpithet: Procanaceconfusa; scientificNameAuthorship: Hardy & Delfinado, 1980; **Location:** islandGroup: Hawaiian Islands; island: Hawaii; verbatimLocality: Hilo, NW of Honolii Stream; minimumElevationInMeters: 1312; **Identification:** identifiedBy: WN Mathis; dateIdentified: 1992; **Event:** verbatimEventDate: 26.vii.1990; **Record Level:** institutionCode: BPBM
- **Type status:** Other material. **Occurrence:** catalogNumber: 2006005101; recordedBy: DA Polhemus; lifeStage: adult; **Taxon:** kingdom: Animalia; phylum: Arthropoda; class: Insecta; order: Diptera; family: Canacidae; genus: Procanace; specificEpithet: Procanaceconfusa; scientificNameAuthorship: Hardy & Delfinado, 1980; **Location:** islandGroup: Hawaiian Islands; island: Hawaii; verbatimLocality: Hilo, NW of Honolii Stream; minimumElevationInMeters: 1312; **Identification:** identifiedBy: WN Mathis; dateIdentified: 1992; **Event:** verbatimEventDate: 26.vii.1990; **Record Level:** institutionCode: BPBM
- **Type status:** Other material. **Occurrence:** catalogNumber: 2006005102; recordedBy: DA Polhemus; lifeStage: adult; **Taxon:** kingdom: Animalia; phylum: Arthropoda; class: Insecta; order: Diptera; family: Canacidae; genus: Procanace; specificEpithet: Procanaceconfusa; scientificNameAuthorship: Hardy & Delfinado, 1980; **Location:** islandGroup: Hawaiian Islands; island: Hawaii; verbatimLocality: Hilo, NW of Honolii Stream; minimumElevationInMeters: 1312; **Identification:** identifiedBy: WN Mathis; dateIdentified: 1992; **Event:** verbatimEventDate: 26.vii.1990; **Record Level:** institutionCode: BPBM
- **Type status:** Other material. **Occurrence:** catalogNumber: 2006005096; recordedBy: DA Polhemus; lifeStage: adult; **Taxon:** kingdom: Animalia; phylum: Arthropoda; class: Insecta; order: Diptera; family: Canacidae; genus: Procanace; specificEpithet: Procanaceconfusa; scientificNameAuthorship: Hardy & Delfinado, 1980; **Location:** islandGroup: Hawaiian Islands; island: Hawaii; verbatimLocality: Hilo, NW of Honolii Stream; minimumElevationInMeters: 1312; **Identification:** identifiedBy: WN Mathis; dateIdentified: 1992; **Event:** verbatimEventDate: 26.vii.1990; **Record Level:** institutionCode: BPBM
- **Type status:** Other material. **Occurrence:** catalogNumber: 2006005082; recordedBy: DA Polhemus; lifeStage: adult; **Taxon:** kingdom: Animalia; phylum: Arthropoda; class: Insecta; order: Diptera; family: Canacidae; genus: Procanace; specificEpithet: Procanaceconfusa; scientificNameAuthorship: Hardy & Delfinado, 1980; **Location:** islandGroup: Hawaiian Islands; island: Hawaii; verbatimLocality: Hilo, NW of Honolii Stream; minimumElevationInMeters: 1312; **Identification:** identifiedBy: WN Mathis; dateIdentified: 1992; **Event:** verbatimEventDate: 26.vii.1990; **Record Level:** institutionCode: BPBM
- **Type status:** Other material. **Occurrence:** catalogNumber: 2006005079; recordedBy: DA Polhemus; lifeStage: adult; **Taxon:** kingdom: Animalia; phylum: Arthropoda; class: Insecta; order: Diptera; family: Canacidae; genus: Procanace; specificEpithet: Procanaceconfusa; scientificNameAuthorship: Hardy & Delfinado, 1980; **Location:** islandGroup: Hawaiian Islands; island: Hawaii; verbatimLocality: Hilo, NW of Honolii Stream; minimumElevationInMeters: 1312; **Identification:** identifiedBy: WN Mathis; dateIdentified: 1992; **Event:** verbatimEventDate: 26.vii.1990; **Record Level:** institutionCode: BPBM
- **Type status:** Other material. **Occurrence:** catalogNumber: 2006005090; recordedBy: DA Polhemus; lifeStage: adult; **Taxon:** kingdom: Animalia; phylum: Arthropoda; class: Insecta; order: Diptera; family: Canacidae; genus: Procanace; specificEpithet: Procanaceconfusa; scientificNameAuthorship: Hardy & Delfinado, 1980; **Location:** islandGroup: Hawaiian Islands; island: Hawaii; verbatimLocality: Hilo, NW of Honolii Stream; minimumElevationInMeters: 1312; **Identification:** identifiedBy: WN Mathis; dateIdentified: 1992; **Event:** verbatimEventDate: 26.vii.1990; **Record Level:** institutionCode: BPBM
- **Type status:** Other material. **Occurrence:** catalogNumber: 2006005081; recordedBy: DA Polhemus; lifeStage: adult; **Taxon:** kingdom: Animalia; phylum: Arthropoda; class: Insecta; order: Diptera; family: Canacidae; genus: Procanace; specificEpithet: Procanaceconfusa; scientificNameAuthorship: Hardy & Delfinado, 1980; **Location:** islandGroup: Hawaiian Islands; island: Hawaii; verbatimLocality: Hilo, NW of Honolii Stream; minimumElevationInMeters: 1312; **Identification:** identifiedBy: WN Mathis; dateIdentified: 1992; **Event:** verbatimEventDate: 26.vii.1990; **Record Level:** institutionCode: BPBM
- **Type status:** Other material. **Occurrence:** catalogNumber: 2006005089; recordedBy: DA Polhemus; lifeStage: adult; **Taxon:** kingdom: Animalia; phylum: Arthropoda; class: Insecta; order: Diptera; family: Canacidae; genus: Procanace; specificEpithet: Procanaceconfusa; scientificNameAuthorship: Hardy & Delfinado, 1980; **Location:** islandGroup: Hawaiian Islands; island: Hawaii; verbatimLocality: Hilo, NW of Honolii Stream; minimumElevationInMeters: 1312; **Identification:** identifiedBy: WN Mathis; dateIdentified: 1992; **Event:** verbatimEventDate: 26.vii.1990; **Record Level:** institutionCode: BPBM
- **Type status:** Other material. **Occurrence:** catalogNumber: 2006005083; recordedBy: DA Polhemus; lifeStage: adult; **Taxon:** kingdom: Animalia; phylum: Arthropoda; class: Insecta; order: Diptera; family: Canacidae; genus: Procanace; specificEpithet: Procanaceconfusa; scientificNameAuthorship: Hardy & Delfinado, 1980; **Location:** islandGroup: Hawaiian Islands; island: Hawaii; verbatimLocality: Hilo, NW of Honolii Stream; minimumElevationInMeters: 1312; **Identification:** identifiedBy: WN Mathis; dateIdentified: 1992; **Event:** verbatimEventDate: 26.vii.1990; **Record Level:** institutionCode: BPBM
- **Type status:** Other material. **Occurrence:** catalogNumber: 2006005084; recordedBy: DA Polhemus; lifeStage: adult; **Taxon:** kingdom: Animalia; phylum: Arthropoda; class: Insecta; order: Diptera; family: Canacidae; genus: Procanace; specificEpithet: Procanaceconfusa; scientificNameAuthorship: Hardy & Delfinado, 1980; **Location:** islandGroup: Hawaiian Islands; island: Hawaii; verbatimLocality: Hilo, NW of Honolii Stream; minimumElevationInMeters: 1312; **Identification:** identifiedBy: WN Mathis; dateIdentified: 1992; **Event:** verbatimEventDate: 26.vii.1990; **Record Level:** institutionCode: BPBM
- **Type status:** Other material. **Occurrence:** catalogNumber: 2006005085; recordedBy: DA Polhemus; lifeStage: adult; **Taxon:** kingdom: Animalia; phylum: Arthropoda; class: Insecta; order: Diptera; family: Canacidae; genus: Procanace; specificEpithet: Procanaceconfusa; scientificNameAuthorship: Hardy & Delfinado, 1980; **Location:** islandGroup: Hawaiian Islands; island: Hawaii; verbatimLocality: Hilo, NW of Honolii Stream; minimumElevationInMeters: 1312; **Identification:** identifiedBy: WN Mathis; dateIdentified: 1992; **Event:** verbatimEventDate: 26.vii.1990; **Record Level:** institutionCode: BPBM
- **Type status:** Other material. **Occurrence:** catalogNumber: 2006005086; recordedBy: DA Polhemus; lifeStage: adult; **Taxon:** kingdom: Animalia; phylum: Arthropoda; class: Insecta; order: Diptera; family: Canacidae; genus: Procanace; specificEpithet: Procanaceconfusa; scientificNameAuthorship: Hardy & Delfinado, 1980; **Location:** islandGroup: Hawaiian Islands; island: Hawaii; verbatimLocality: Hilo, NW of Honolii Stream; minimumElevationInMeters: 1312; **Identification:** identifiedBy: WN Mathis; dateIdentified: 1992; **Event:** verbatimEventDate: 26.vii.1990; **Record Level:** institutionCode: BPBM
- **Type status:** Other material. **Occurrence:** catalogNumber: 2006005087; recordedBy: DA Polhemus; lifeStage: adult; **Taxon:** kingdom: Animalia; phylum: Arthropoda; class: Insecta; order: Diptera; family: Canacidae; genus: Procanace; specificEpithet: Procanaceconfusa; scientificNameAuthorship: Hardy & Delfinado, 1980; **Location:** islandGroup: Hawaiian Islands; island: Hawaii; verbatimLocality: Hilo, NW of Honolii Stream; minimumElevationInMeters: 1312; **Identification:** identifiedBy: WN Mathis; dateIdentified: 1992; **Event:** verbatimEventDate: 26.vii.1990; **Record Level:** institutionCode: BPBM
- **Type status:** Other material. **Occurrence:** catalogNumber: 2006005080; recordedBy: DA Polhemus; lifeStage: adult; **Taxon:** kingdom: Animalia; phylum: Arthropoda; class: Insecta; order: Diptera; family: Canacidae; genus: Procanace; specificEpithet: Procanaceconfusa; scientificNameAuthorship: Hardy & Delfinado, 1980; **Location:** islandGroup: Hawaiian Islands; island: Hawaii; verbatimLocality: Hilo, NW of Honolii Stream; minimumElevationInMeters: 1312; **Identification:** identifiedBy: WN Mathis; dateIdentified: 1992; **Event:** verbatimEventDate: 26.vii.1990; **Record Level:** institutionCode: BPBM
- **Type status:** Other material. **Occurrence:** recordedBy: DA Polhemus; individualCount: 25; sex: 11 males, 14 females; lifeStage: adult; **Taxon:** kingdom: Animalia; phylum: Arthropoda; class: Insecta; order: Diptera; family: Canacidae; genus: Procanace; specificEpithet: Procanaceconfusa; scientificNameAuthorship: Hardy & Delfinado, 1980; **Location:** islandGroup: Hawaiian Islands; island: Hawaii; verbatimLocality: Hilo, NW of Honolii Stream; minimumElevationInMeters: 1312; **Identification:** identifiedBy: WN Mathis; dateIdentified: 1992; **Event:** verbatimEventDate: 26.vii.1990; **Record Level:** institutionCode: USNM
- **Type status:** Other material. **Occurrence:** catalogNumber: 2006005103; recordedBy: DA Polhemus; lifeStage: adult; **Taxon:** kingdom: Animalia; phylum: Arthropoda; class: Insecta; order: Diptera; family: Canacidae; genus: Procanace; specificEpithet: Procanaceconfusa; scientificNameAuthorship: Hardy & Delfinado, 1980; **Location:** islandGroup: Hawaiian Islands; island: Hawaii; verbatimLocality: Hilo, NW of Honolii Stream; minimumElevationInMeters: 1312; **Identification:** identifiedBy: WN Mathis; dateIdentified: 1992; **Event:** verbatimEventDate: 26.x.1990; **Record Level:** institutionCode: BPBM
- **Type status:** Other material. **Occurrence:** catalogNumber: 2006005104; recordedBy: DA Polhemus; lifeStage: adult; **Taxon:** kingdom: Animalia; phylum: Arthropoda; class: Insecta; order: Diptera; family: Canacidae; genus: Procanace; specificEpithet: Procanaceconfusa; scientificNameAuthorship: Hardy & Delfinado, 1980; **Location:** islandGroup: Hawaiian Islands; island: Hawaii; verbatimLocality: Hilo, NW of Honolii Stream; minimumElevationInMeters: 1312; **Identification:** identifiedBy: WN Mathis; dateIdentified: 1992; **Event:** verbatimEventDate: 26.x.1990; **Record Level:** institutionCode: BPBM
- **Type status:** Other material. **Occurrence:** catalogNumber: 2006005105; recordedBy: DA Polhemus; lifeStage: adult; **Taxon:** kingdom: Animalia; phylum: Arthropoda; class: Insecta; order: Diptera; family: Canacidae; genus: Procanace; specificEpithet: Procanaceconfusa; scientificNameAuthorship: Hardy & Delfinado, 1980; **Location:** islandGroup: Hawaiian Islands; island: Hawaii; verbatimLocality: Hilo, NW of Honolii Stream; minimumElevationInMeters: 1312; **Identification:** identifiedBy: WN Mathis; dateIdentified: 1992; **Event:** verbatimEventDate: 26.x.1990; **Record Level:** institutionCode: BPBM
- **Type status:** Other material. **Occurrence:** catalogNumber: 2006005072; recordedBy: DA Polhemus; lifeStage: adult; **Taxon:** kingdom: Animalia; phylum: Arthropoda; class: Insecta; order: Diptera; family: Canacidae; genus: Procanace; specificEpithet: Procanaceconfusa; scientificNameAuthorship: Hardy & Delfinado, 1980; **Location:** islandGroup: Hawaiian Islands; island: Maui; verbatimLocality: West Maui, Iao Valley; **Identification:** identifiedBy: WN Mathis; dateIdentified: 1992; **Event:** verbatimEventDate: 30.x.1990; **Record Level:** institutionCode: BPBM
- **Type status:** Other material. **Occurrence:** catalogNumber: 2006005073; recordedBy: DA Polhemus; lifeStage: adult; **Taxon:** kingdom: Animalia; phylum: Arthropoda; class: Insecta; order: Diptera; family: Canacidae; genus: Procanace; specificEpithet: Procanaceconfusa; scientificNameAuthorship: Hardy & Delfinado, 1980; **Location:** islandGroup: Hawaiian Islands; island: Maui; **Identification:** identifiedBy: WN Mathis; dateIdentified: 1992; **Event:** verbatimEventDate: 30.x.1990; **Record Level:** institutionCode: BPBM
- **Type status:** Other material. **Occurrence:** catalogNumber: 2006005074; recordedBy: DA Polhemus; lifeStage: adult; **Taxon:** kingdom: Animalia; phylum: Arthropoda; class: Insecta; order: Diptera; family: Canacidae; genus: Procanace; specificEpithet: Procanaceconfusa; scientificNameAuthorship: Hardy & Delfinado, 1980; **Location:** islandGroup: Hawaiian Islands; island: Maui; verbatimLocality: West Maui, Iao Valley; **Identification:** identifiedBy: WN Mathis; dateIdentified: 1992; **Event:** verbatimEventDate: 30.x.1990; **Record Level:** institutionCode: BPBM
- **Type status:** Other material. **Occurrence:** catalogNumber: 2006005075; recordedBy: DA Polhemus; lifeStage: adult; **Taxon:** kingdom: Animalia; phylum: Arthropoda; class: Insecta; order: Diptera; family: Canacidae; genus: Procanace; specificEpithet: Procanaceconfusa; scientificNameAuthorship: Hardy & Delfinado, 1980; **Location:** islandGroup: Hawaiian Islands; island: Maui; verbatimLocality: West Maui, Iao Valley; **Identification:** identifiedBy: WN Mathis; dateIdentified: 1992; **Event:** verbatimEventDate: 30.x.1990; **Record Level:** institutionCode: BPBM
- **Type status:** Other material. **Occurrence:** catalogNumber: 2006005076; recordedBy: DA Polhemus; lifeStage: adult; **Taxon:** kingdom: Animalia; phylum: Arthropoda; class: Insecta; order: Diptera; family: Canacidae; genus: Procanace; specificEpithet: Procanaceconfusa; scientificNameAuthorship: Hardy & Delfinado, 1980; **Location:** islandGroup: Hawaiian Islands; island: Maui; verbatimLocality: West Maui, Iao Valley; **Identification:** identifiedBy: WN Mathis; dateIdentified: 1992; **Event:** verbatimEventDate: 30.x.1990; **Record Level:** institutionCode: BPBM
- **Type status:** Other material. **Occurrence:** catalogNumber: 2006005077; recordedBy: DA Polhemus; lifeStage: adult; **Taxon:** kingdom: Animalia; phylum: Arthropoda; class: Insecta; order: Diptera; family: Canacidae; genus: Procanace; specificEpithet: Procanaceconfusa; scientificNameAuthorship: Hardy & Delfinado, 1980; **Location:** islandGroup: Hawaiian Islands; island: Maui; verbatimLocality: West Maui, Iao Valley; **Identification:** identifiedBy: WN Mathis; dateIdentified: 1992; **Event:** verbatimEventDate: 30.x.1990; **Record Level:** institutionCode: BPBM
- **Type status:** Other material. **Occurrence:** catalogNumber: 2006005078; recordedBy: DA Polhemus; lifeStage: adult; **Taxon:** kingdom: Animalia; phylum: Arthropoda; class: Insecta; order: Diptera; family: Canacidae; genus: Procanace; specificEpithet: Procanaceconfusa; scientificNameAuthorship: Hardy & Delfinado, 1980; **Location:** islandGroup: Hawaiian Islands; island: Maui; verbatimLocality: West Maui, Iao Valley; **Identification:** identifiedBy: WN Mathis; dateIdentified: 1992; **Event:** verbatimEventDate: 30.x.1990; **Record Level:** institutionCode: BPBM
- **Type status:** Other material. **Occurrence:** recordedBy: DA Polhemus; individualCount: 4; sex: 1 male, 3 females; lifeStage: adult; **Taxon:** kingdom: Animalia; phylum: Arthropoda; class: Insecta; order: Diptera; family: Canacidae; genus: Procanace; specificEpithet: Procanaceconfusa; scientificNameAuthorship: Hardy & Delfinado, 1980; **Location:** islandGroup: Hawaiian Islands; island: Maui; verbatimLocality: West Maui, Iao Valley; **Identification:** identifiedBy: WN Mathis; dateIdentified: 1992; **Event:** verbatimEventDate: 30.x.1990; **Record Level:** institutionCode: USNM

##### Ecological interactions

###### Native status

endemic

##### Distribution

**Figure 12.**
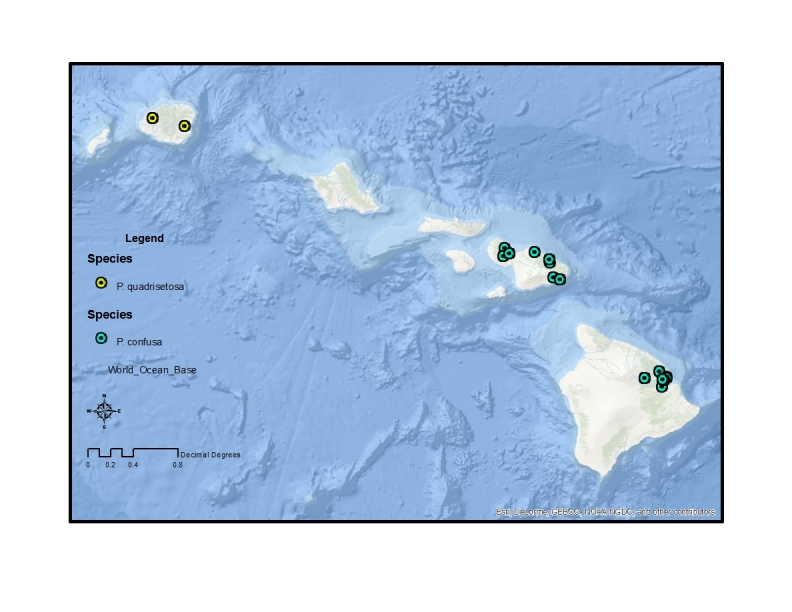
Distribution of *P. confusa* (Maui, Hawaii) and *P. quadrisetosa* (Kauai).

HAWAIIAN ISLANDS: Maui, Hawaii (Fig. 12​).

##### Notes

Hardy and Delfinado (1980), [original description; female terminalia (dorsal and ventral), spermathecae, ninth sternum, epandrium and surstylus]; Mathis (1992), [World Catalog]; Nishida (2002), [Hawaiian Arthropod Checklist].

#### Procanace constricta

Hardy and Delfinado, 1980

##### Materials

- **Type status:** Holotype. **Occurrence:** recordedBy: DE Hardy; individualCount: 1; sex: male; lifeStage: adult; **Taxon:** kingdom: Animalia; phylum: Arthropoda; class: Insecta; order: Diptera; family: Canacidae; genus: Procanace; specificEpithet: Procanaceconstricta; scientificNameAuthorship: Hardy & Delfinado, 1980; **Location:** islandGroup: Hawaiian Islands; island: Molokai; verbatimLocality: Halawa Valley, collected on wet rocks in swift moving stream; **Identification:** identifiedBy: DE Hardy & MD Delfinado; dateIdentified: 1980; **Event:** verbatimEventDate: vii.1952; **Record Level:** institutionCode: BPBM
- **Type status:** Paratype. **Occurrence:** recordedBy: DE Hardy; individualCount: 1; sex: female; lifeStage: adult; **Taxon:** kingdom: Animalia; phylum: Arthropoda; class: Insecta; order: Diptera; family: Canacidae; genus: Procanace; specificEpithet: Procanaceconstricta; scientificNameAuthorship: Hardy & Delfinado, 1980; **Location:** islandGroup: Hawaiian Islands; island: Molokai; verbatimLocality: Halawa Valley, collected on wet rocks in swift moving stream; **Identification:** identifiedBy: DE Hardy & MD Delfinado; dateIdentified: 1980; **Event:** verbatimEventDate: vii.1952; **Record Level:** institutionCode: BPBM
- **Type status:** Paratype. **Occurrence:** recordedBy: DE Hardy; lifeStage: adult; **Taxon:** kingdom: Animalia; phylum: Arthropoda; class: Insecta; order: Diptera; family: Canacidae; genus: Procanace; specificEpithet: Procanaceconstricta; scientificNameAuthorship: Hardy & Delfinado, 1980; **Location:** islandGroup: Hawaiian Islands; island: Molokai; verbatimLocality: Halawa Valley, collected on wet rocks in swift moving stream; **Identification:** identifiedBy: DE Hardy & MD Delfinado; dateIdentified: 1980; **Event:** verbatimEventDate: vii.1952
- **Type status:** Paratype. **Occurrence:** recordedBy: DE Hardy; sex: 1 female; lifeStage: adult; **Taxon:** kingdom: Animalia; phylum: Arthropoda; class: Insecta; order: Diptera; family: Canacidae; genus: Procanace; specificEpithet: Procanaceconstricta; scientificNameAuthorship: Hardy & Delfinado, 1980; **Location:** islandGroup: Hawaiian Islands; island: Maui; verbatimLocality: Wailua, Maui; **Identification:** identifiedBy: DE Hardy & MD Delfinado; dateIdentified: 1980; **Event:** verbatimEventDate: vii.1953; **Record Level:** institutionCode: USNM
- **Type status:** Paratype. **Occurrence:** recordedBy: DE Hardy, JA Tenorio; sex: 1 female; lifeStage: adult; **Taxon:** kingdom: Animalia; phylum: Arthropoda; class: Insecta; order: Diptera; family: Canacidae; genus: Procanace; specificEpithet: Procanaceconstricta; scientificNameAuthorship: Hardy & Delfinado, 1980; **Location:** islandGroup: Hawaiian Islands; island: Molokai; verbatimLocality: below Moaulu Falls; **Identification:** identifiedBy: DE Hardy & MD Delfinado; dateIdentified: 1980; **Event:** verbatimEventDate: 16.iii.1970; **Record Level:** institutionCode: USNM
- **Type status:** Paratype. **Occurrence:** recordedBy: JA Tenorio; sex: 1 female; lifeStage: adult; **Taxon:** kingdom: Animalia; phylum: Arthropoda; class: Insecta; order: Diptera; family: Canacidae; genus: Procanace; specificEpithet: Procanaceconstricta; scientificNameAuthorship: Hardy & Delfinado, 1980; **Location:** islandGroup: Hawaiian Islands; island: Molokai; verbatimLocality: Halawa Valley, waterfalls; **Identification:** identifiedBy: DE Hardy & MD Delfinado; dateIdentified: 1980; **Event:** verbatimEventDate: 16.iii.1970; **Record Level:** institutionCode: USNM
- **Type status:** Paratype. **Occurrence:** recordedBy: DE Hardy, JA Tenorio; lifeStage: adult; **Taxon:** kingdom: Animalia; phylum: Arthropoda; class: Insecta; order: Diptera; family: Canacidae; genus: Procanace; specificEpithet: Procanaceconstricta; scientificNameAuthorship: Hardy & Delfinado, 1980; **Location:** islandGroup: Hawaiian Islands; island: Maui; verbatimLocality: Kopiliula and Waikane Streams; **Identification:** identifiedBy: DE Hardy & MD Delfinado; dateIdentified: 1980; **Event:** verbatimEventDate: 9.iv.1970
- **Type status:** Paratype. **Occurrence:** recordedBy: J Kjargaard; lifeStage: adult; **Taxon:** kingdom: Animalia; phylum: Arthropoda; class: Insecta; order: Diptera; family: Canacidae; genus: Procanace; specificEpithet: Procanaceconstricta; scientificNameAuthorship: Hardy & Delfinado, 1980; **Location:** islandGroup: Hawaiian Islands; island: Molokai; verbatimLocality: Wailua Stream gauge; verbatimElevation: 605 ft.; **Identification:** identifiedBy: DE Hardy & MD Delfinado; dateIdentified: 1980; **Event:** verbatimEventDate: 4.vi.1970
- **Type status:** Paratype. **Occurrence:** recordedBy: DE Hardy; individualCount: 7; lifeStage: adult; **Taxon:** kingdom: Animalia; phylum: Arthropoda; class: Insecta; order: Diptera; family: Canacidae; genus: Procanace; specificEpithet: Procanaceconstricta; scientificNameAuthorship: Hardy & Delfinado, 1980; **Location:** islandGroup: Hawaiian Islands; island: Maui; verbatimLocality: Waikane Stream; verbatimElevation: 600 ft.; **Identification:** identifiedBy: DE Hardy & MD Delfinado; dateIdentified: 1980; **Event:** verbatimEventDate: 4.ix.1970; **Record Level:** institutionCode: UHM
- **Type status:** Paratype. **Occurrence:** recordedBy: DE Hardy; individualCount: 7; lifeStage: adult; **Taxon:** kingdom: Animalia; phylum: Arthropoda; class: Insecta; order: Diptera; family: Canacidae; genus: Procanace; specificEpithet: Procanaceconstricta; scientificNameAuthorship: Hardy & Delfinado, 1980; **Location:** islandGroup: Hawaiian Islands; island: Maui; verbatimLocality: Kopiliula Stream, Hana; verbatimElevation: 1200 ft.; **Identification:** identifiedBy: DE Hardy & MD Delfinado; dateIdentified: 1980; **Event:** verbatimEventDate: 4.ix.1970; **Record Level:** institutionCode: UHM
- **Type status:** Paratype. **Occurrence:** recordedBy: DE Hardy; individualCount: 2; sex: 2 females; lifeStage: adult; **Taxon:** kingdom: Animalia; phylum: Arthropoda; class: Insecta; order: Diptera; family: Canacidae; genus: Procanace; specificEpithet: Procanaceconstricta; scientificNameAuthorship: Hardy & Delfinado, 1980; **Location:** islandGroup: Hawaiian Islands; island: Maui; verbatimLocality: Kopiliula Stream, Hana; verbatimElevation: 1200 ft.; **Identification:** identifiedBy: DE Hardy & MD Delfinado; dateIdentified: 1980; **Event:** verbatimEventDate: 4.ix.1970; **Record Level:** institutionCode: USNM
- **Type status:** Paratype. **Occurrence:** recordedBy: DE Hardy; sex: 1 female; lifeStage: adult; **Taxon:** kingdom: Animalia; phylum: Arthropoda; class: Insecta; order: Diptera; family: Canacidae; genus: Procanace; specificEpithet: Procanaceconstricta; scientificNameAuthorship: Hardy & Delfinado, 1980; **Location:** islandGroup: Hawaiian Islands; island: Maui; verbatimLocality: Waikane Stream; verbatimElevation: 600 ft.; **Identification:** identifiedBy: DE Hardy & MD Delfinado; dateIdentified: 1980; **Event:** verbatimEventDate: 4.ix.1970; **Record Level:** institutionCode: USNM
- **Type status:** Paratype. **Occurrence:** recordedBy: MD Delfinado, JA Tenorio; lifeStage: adult; **Taxon:** kingdom: Animalia; phylum: Arthropoda; class: Insecta; order: Diptera; family: Canacidae; genus: Procanace; specificEpithet: Procanaceconstricta; scientificNameAuthorship: Hardy & Delfinado, 1980; **Location:** islandGroup: Hawaiian Islands; island: Hawaii; verbatimLocality: Wailuku River; **Identification:** identifiedBy: DE Hardy & MD Delfinado; dateIdentified: 1980; **Event:** verbatimEventDate: 22.vi.1971
- **Type status:** Paratype. **Occurrence:** recordedBy: JA Tenorio; individualCount: 4; lifeStage: adult; **Taxon:** kingdom: Animalia; phylum: Arthropoda; class: Insecta; order: Diptera; family: Canacidae; genus: Procanace; specificEpithet: Procanaceconstricta; scientificNameAuthorship: Hardy & Delfinado, 1980; **Location:** islandGroup: Hawaiian Islands; island: Hawaii; verbatimLocality: Wailuku River; verbatimElevation: 500 ft.; **Identification:** identifiedBy: DE Hardy & MD Delfinado; dateIdentified: 1980; **Event:** verbatimEventDate: 23.vi.1971; **Record Level:** institutionCode: UHM
- **Type status:** Other material. **Occurrence:** recordedBy: WW Wirth; sex: 1 female; lifeStage: adult; **Taxon:** kingdom: Animalia; phylum: Arthropoda; class: Insecta; order: Diptera; family: Canacidae; genus: Procanace; specificEpithet: Procanaceconstricta; scientificNameAuthorship: Cresson, 1926; **Location:** islandGroup: Hawaiian Islands; island: Oahu; verbatimLocality: Kaluanui Valley, at a stream; verbatimElevation: 609 m; **Identification:** identifiedBy: PM O'Grady; dateIdentified: 22 Jan 2016; **Event:** verbatimEventDate: 14.v.1946; **Record Level:** institutionCode: USNM
- **Type status:** Other material. **Occurrence:** recordedBy: MD Delfinado; individualCount: 2; lifeStage: adult; **Taxon:** kingdom: Animalia; phylum: Arthropoda; class: Insecta; order: Diptera; family: Canacidae; genus: Procanace; specificEpithet: Procanaceconstricta; scientificNameAuthorship: Hardy & Delfinado, 1980; **Location:** islandGroup: Hawaiian Islands; island: Maui; verbatimLocality: Seven Sacred Pools; **Event:** verbatimEventDate: 3.viii.1970; **Record Level:** institutionCode: UHM
- **Type status:** Other material. **Occurrence:** recordedBy: DE Hardy; individualCount: 26; lifeStage: adult; **Taxon:** kingdom: Animalia; phylum: Arthropoda; class: Insecta; order: Diptera; family: Canacidae; genus: Procanace; specificEpithet: Procanaceconstricta; scientificNameAuthorship: Hardy & Delfinado, 1980; **Location:** islandGroup: Hawaiian Islands; island: Maui; verbatimLocality: Kopiliula Stream, Hana; verbatimElevation: 1200 ft.; **Event:** verbatimEventDate: 4.ix.1970; **Record Level:** institutionCode: UHM
- **Type status:** Other material. **Occurrence:** catalogNumber: 2006005120; recordedBy: DE Hardy; lifeStage: adult; **Taxon:** kingdom: Animalia; phylum: Arthropoda; class: Insecta; order: Diptera; family: Canacidae; genus: Procanace; specificEpithet: Procanaceconstricta; scientificNameAuthorship: Hardy & Delfinado, 1980; **Location:** islandGroup: Hawaiian Islands; island: Maui; verbatimLocality: Waikane Stream; minimumElevationInMeters: 600; **Identification:** identifiedBy: DE Hardy & MD Delfinado; **Event:** verbatimEventDate: 04.ix.1970; **Record Level:** institutionCode: BPBM
- **Type status:** Other material. **Occurrence:** catalogNumber: 2006005121; recordedBy: DE Hardy; lifeStage: adult; **Taxon:** kingdom: Animalia; phylum: Arthropoda; class: Insecta; order: Diptera; family: Canacidae; genus: Procanace; specificEpithet: Procanaceconstricta; scientificNameAuthorship: Hardy & Delfinado, 1980; **Location:** islandGroup: Hawaiian Islands; island: Maui; verbatimLocality: Waikane Stream; minimumElevationInMeters: 600; **Identification:** identifiedBy: DE Hardy & MD Delfinado; **Event:** verbatimEventDate: 04.ix.1970; **Record Level:** institutionCode: BPBM
- **Type status:** Other material. **Occurrence:** catalogNumber: 2006005122; recordedBy: DE Hardy; lifeStage: adult; **Taxon:** kingdom: Animalia; phylum: Arthropoda; class: Insecta; order: Diptera; family: Canacidae; genus: Procanace; specificEpithet: Procanaceconstricta; scientificNameAuthorship: Hardy & Delfinado, 1980; **Location:** islandGroup: Hawaiian Islands; island: Maui; verbatimLocality: Waikane Stream; minimumElevationInMeters: 600; **Identification:** identifiedBy: DE Hardy & MD Delfinado; **Event:** verbatimEventDate: 04.ix.1970; **Record Level:** institutionCode: BPBM
- **Type status:** Other material. **Occurrence:** catalogNumber: 2006005123; recordedBy: DE Hardy; lifeStage: adult; **Taxon:** kingdom: Animalia; phylum: Arthropoda; class: Insecta; order: Diptera; family: Canacidae; genus: Procanace; specificEpithet: Procanaceconstricta; scientificNameAuthorship: Hardy & Delfinado, 1980; **Location:** islandGroup: Hawaiian Islands; island: Maui; verbatimLocality: Waikane Stream; minimumElevationInMeters: 600; **Identification:** identifiedBy: DE Hardy & MD Delfinado; **Event:** verbatimEventDate: 04.ix.1970; **Record Level:** institutionCode: BPBM
- **Type status:** Other material. **Occurrence:** recordedBy: DE Hardy; individualCount: 5; lifeStage: adult; **Taxon:** kingdom: Animalia; phylum: Arthropoda; class: Insecta; order: Diptera; family: Canacidae; genus: Procanace; specificEpithet: Procanaceconstricta; scientificNameAuthorship: Hardy & Delfinado, 1980; **Location:** islandGroup: Hawaiian Islands; island: Maui; verbatimLocality: Kipahulu Valley; verbatimElevation: 500 ft.; **Event:** verbatimEventDate: 2.x.1970; **Record Level:** institutionCode: UHM
- **Type status:** Other material. **Occurrence:** recordedBy: DE Hardy; individualCount: 2; lifeStage: adult; **Taxon:** kingdom: Animalia; phylum: Arthropoda; class: Insecta; order: Diptera; family: Canacidae; genus: Procanace; specificEpithet: Procanaceconstricta; scientificNameAuthorship: Hardy & Delfinado, 1980; **Location:** islandGroup: Hawaiian Islands; island: Maui; verbatimLocality: Kinihapai Stream, Iao Valley; verbatimElevation: 500 ft.; **Event:** verbatimEventDate: 2.x.1970; **Record Level:** institutionCode: UHM
- **Type status:** Other material. **Occurrence:** recordedBy: BS Ort; individualCount: 4; sex: females; lifeStage: adult; **Taxon:** kingdom: Animalia; phylum: Arthropoda; class: Insecta; order: Diptera; family: Canacidae; genus: Procanace; specificEpithet: Procanaceconstricta; scientificNameAuthorship: Hardy & Delfinado, 1980; **Location:** islandGroup: Hawaiian Islands; island: Hawaii; verbatimLocality: Waipio Valley,Â Near base of He'eilawe Falls at dammed reservoir; **Identification:** identifiedBy: PM O'Grady; dateIdentified: 2014; **Event:** verbatimEventDate: 2.viii.2010; **Record Level:** institutionCode: EMEC
- **Type status:** Other material. **Occurrence:** recordedBy: PM O'Grady, RT Lapoint, B Ort, NA Pantoja; lifeStage: adult; **Taxon:** kingdom: Animalia; phylum: Arthropoda; class: Insecta; order: Diptera; family: Canacidae; genus: Procanace; specificEpithet: Procanaceconstricta; scientificNameAuthorship: Hardy & Delfinado, 1980; **Location:** islandGroup: Hawaiian Islands; island: Hawaii; verbatimLocality: Kohala Mountains, sweeping in stream where bridge crosses road, 0.5 miles before end; **Identification:** identifiedBy: PM O'Grady; dateIdentified: 2014; **Event:** verbatimEventDate: 4.viii.2010; **Record Level:** institutionCode: EMEC; collectionCode: 205268

##### Ecological interactions

###### Native status

endemic

##### Distribution

**Figure 13.**
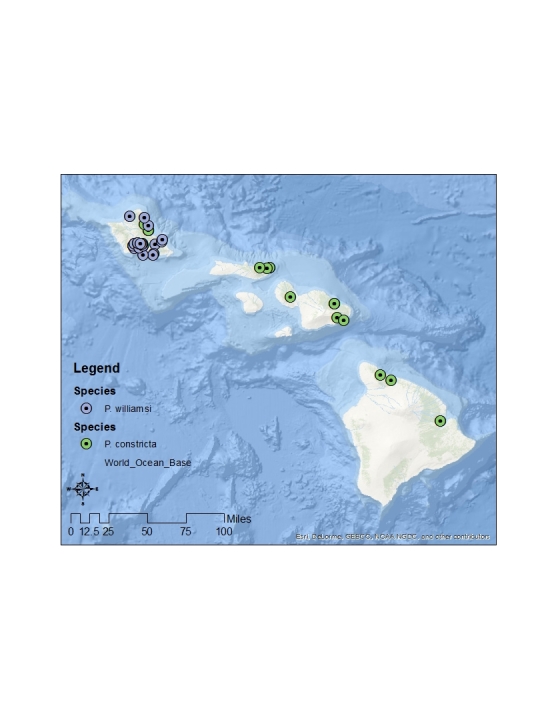
Distribution of *P. constricta* (Molokai, Maui, Hawaii) and *P. williamsi* (Oahu).

HAWAIIAN ISLANDS: Oahu, Molokai, Maui, Hawaii (Fig. 13​).

##### Notes

Hardy and Delfinado (1980), [original description; female terminalia (dorsal and ventral), spermathecae, ninth sternum, epandrium and surstylus]; Mathis (1992), [World Catalog]; Nishida (2002), [Hawaiian Arthropod Checklist].

#### Procanace nigroviridis

Cresson, 1926

##### Materials

- **Type status:** Holotype. **Occurrence:** recordedBy: EH Bryan; individualCount: 1; sex: male; lifeStage: adult; **Taxon:** kingdom: Animalia; phylum: Arthropoda; class: Insecta; order: Diptera; family: Canacidae; genus: Procanace; specificEpithet: Procanacenigroviridis; scientificNameAuthorship: Cresson, 1926; **Location:** islandGroup: Hawaiian Islands; island: Kauai; verbatimLocality: Awaawapuhi; **Identification:** identifiedBy: DE Hardy & MD Delfinado; dateIdentified: 1980; **Event:** verbatimEventDate: 16.vi.1922; **Record Level:** institutionCode: BPBM
- **Type status:** Other material. **Occurrence:** catalogNumber: 2006005125; recordedBy: EH Bryan, Jr.; lifeStage: adult; **Taxon:** kingdom: Animalia; phylum: Arthropoda; class: Insecta; order: Diptera; family: Canacidae; genus: Procanace; specificEpithet: Procanacenigroviridis; scientificNameAuthorship: Cresson, 1926; **Location:** islandGroup: Hawaiian Islands; island: Kauai; verbatimLocality: Awaawapuhi; **Identification:** identifiedBy: DE Hardy; dateIdentified: 1980; **Event:** verbatimEventDate: 16.vi.1922; **Record Level:** institutionCode: BPBM
- **Type status:** Other material. **Occurrence:** recordedBy: DE Hardy; individualCount: 101; lifeStage: adult; **Taxon:** kingdom: Animalia; phylum: Arthropoda; class: Insecta; order: Diptera; family: Canacidae; genus: Procanace; specificEpithet: Procanacenigroviridis; scientificNameAuthorship: Cresson, 1926; **Location:** islandGroup: Hawaiian Islands; island: Kauai; verbatimLocality: Kalalau Valley; **Event:** verbatimEventDate: viii.1953; **Record Level:** institutionCode: UHM
- **Type status:** Other material. **Occurrence:** recordedBy: J Kjargaard; individualCount: 1; lifeStage: adult; **Taxon:** kingdom: Animalia; phylum: Arthropoda; class: Insecta; order: Diptera; family: Canacidae; genus: Procanace; specificEpithet: Procanacenigroviridis; scientificNameAuthorship: Cresson, 1926; **Location:** islandGroup: Hawaiian Islands; island: Kauai; verbatimLocality: Kalalau; verbatimElevation: 2.5 miles upstream on rocks; **Event:** verbatimEventDate: 22.iii.1970; **Record Level:** institutionCode: UHM
- **Type status:** Other material. **Occurrence:** recordedBy: L Uyenishi; individualCount: 1; lifeStage: adult; **Taxon:** kingdom: Animalia; phylum: Arthropoda; class: Insecta; order: Diptera; family: Canacidae; genus: Procanace; specificEpithet: Procanacenigroviridis; scientificNameAuthorship: Cresson, 1926; **Location:** islandGroup: Hawaiian Islands; island: Kauai; verbatimLocality: Waipoo Falls; **Event:** verbatimEventDate: 2.iv.1970; **Record Level:** institutionCode: UHM
- **Type status:** Other material. **Occurrence:** recordedBy: J Kjargaard; individualCount: 5; lifeStage: adult; **Taxon:** kingdom: Animalia; phylum: Arthropoda; class: Insecta; order: Diptera; family: Canacidae; genus: Procanace; specificEpithet: Procanacenigroviridis; scientificNameAuthorship: Cresson, 1926; **Location:** islandGroup: Hawaiian Islands; island: Kauai; verbatimLocality: Kalalau; verbatimElevation: 3/4 mile upstream on rocks; **Event:** verbatimEventDate: 4.iv.1970; **Record Level:** institutionCode: UHM
- **Type status:** Other material. **Occurrence:** recordedBy: MD Delfinado; individualCount: 1; lifeStage: adult; **Taxon:** kingdom: Animalia; phylum: Arthropoda; class: Insecta; order: Diptera; family: Canacidae; genus: Procanace; specificEpithet: Procanacenigroviridis; scientificNameAuthorship: Cresson, 1926; **Location:** islandGroup: Hawaiian Islands; island: Kauai; verbatimLocality: Top of Wailua Falls; **Event:** verbatimEventDate: 4.iv.1970; **Record Level:** institutionCode: UHM
- **Type status:** Other material. **Occurrence:** recordedBy: MD Delfinado; individualCount: 13; lifeStage: adult; **Taxon:** kingdom: Animalia; phylum: Arthropoda; class: Insecta; order: Diptera; family: Canacidae; genus: Procanace; specificEpithet: Procanacenigroviridis; scientificNameAuthorship: Cresson, 1926; **Location:** islandGroup: Hawaiian Islands; island: Kauai; verbatimLocality: Wailua Falls; **Event:** verbatimEventDate: 4.iv.1970; **Record Level:** institutionCode: UHM
- **Type status:** Other material. **Occurrence:** recordedBy: J Kjargaard; individualCount: 5; sex: 1 male, 4 females; lifeStage: adult; **Taxon:** kingdom: Animalia; phylum: Arthropoda; class: Insecta; order: Diptera; family: Canacidae; genus: Procanace; specificEpithet: Procanacenigroviridis; scientificNameAuthorship: Cresson, 1926; **Location:** islandGroup: Hawaiian Islands; island: Kauai; verbatimLocality: Kalalau; verbatimElevation: 3/4 mile upstream on rocks; **Event:** verbatimEventDate: 4.iv.1970; **Record Level:** institutionCode: USNM
- **Type status:** Other material. **Occurrence:** catalogNumber: 2006005163; recordedBy: DA Polhemus; lifeStage: adult; **Taxon:** kingdom: Animalia; phylum: Arthropoda; class: Insecta; order: Diptera; family: Canacidae; genus: Procanace; specificEpithet: Procanacenigroviridis; scientificNameAuthorship: Cresson, 1926; **Location:** islandGroup: Hawaiian Islands; island: Kauai; verbatimLocality: Makaleha Stream, at Makaleha Springs; minimumElevationInMeters: 787; **Identification:** identifiedBy: WN Mathis; dateIdentified: 1992; **Event:** verbatimEventDate: 08.xi.1990; **Record Level:** institutionCode: BPBM
- **Type status:** Other material. **Occurrence:** catalogNumber: 2006005156; recordedBy: DA Polhemus; lifeStage: adult; **Taxon:** kingdom: Animalia; phylum: Arthropoda; class: Insecta; order: Diptera; family: Canacidae; genus: Procanace; specificEpithet: Procanacenigroviridis; scientificNameAuthorship: Cresson, 1926; **Location:** islandGroup: Hawaiian Islands; island: Kauai; verbatimLocality: Makaleha Stream, at Makaleha Springs; minimumElevationInMeters: 787; **Identification:** identifiedBy: WN Mathis; dateIdentified: 1992; **Event:** verbatimEventDate: 08.xi.1990; **Record Level:** institutionCode: BPBM
- **Type status:** Other material. **Occurrence:** catalogNumber: 2006005157; recordedBy: DA Polhemus; lifeStage: adult; **Taxon:** kingdom: Animalia; phylum: Arthropoda; class: Insecta; order: Diptera; family: Canacidae; genus: Procanace; specificEpithet: Procanacenigroviridis; scientificNameAuthorship: Cresson, 1926; **Location:** islandGroup: Hawaiian Islands; island: Kauai; verbatimLocality: Makaleha Stream, at Makaleha Springs; minimumElevationInMeters: 787; **Identification:** identifiedBy: WN Mathis; dateIdentified: 1992; **Event:** verbatimEventDate: 08.xi.1990; **Record Level:** institutionCode: BPBM
- **Type status:** Other material. **Occurrence:** catalogNumber: 2006005158; recordedBy: DA Polhemus; lifeStage: adult; **Taxon:** kingdom: Animalia; phylum: Arthropoda; class: Insecta; order: Diptera; family: Canacidae; genus: Procanace; specificEpithet: Procanacenigroviridis; scientificNameAuthorship: Cresson, 1926; **Location:** islandGroup: Hawaiian Islands; island: Kauai; verbatimLocality: Makaleha Stream, at Makaleha Springs; minimumElevationInMeters: 787; **Identification:** identifiedBy: WN Mathis; dateIdentified: 1992; **Event:** verbatimEventDate: 08.xi.1990; **Record Level:** institutionCode: BPBM
- **Type status:** Other material. **Occurrence:** catalogNumber: 2006005159; recordedBy: DA Polhemus; lifeStage: adult; **Taxon:** kingdom: Animalia; phylum: Arthropoda; class: Insecta; order: Diptera; family: Canacidae; genus: Procanace; specificEpithet: Procanacenigroviridis; scientificNameAuthorship: Cresson, 1926; **Location:** islandGroup: Hawaiian Islands; island: Kauai; verbatimLocality: Makaleha Stream, at Makaleha Springs; minimumElevationInMeters: 787; **Identification:** identifiedBy: WN Mathis; dateIdentified: 1992; **Event:** verbatimEventDate: 08.xi.1990; **Record Level:** institutionCode: BPBM
- **Type status:** Other material. **Occurrence:** catalogNumber: 2006005160; recordedBy: DA Polhemus; lifeStage: adult; **Taxon:** kingdom: Animalia; phylum: Arthropoda; class: Insecta; order: Diptera; family: Canacidae; genus: Procanace; specificEpithet: Procanacenigroviridis; scientificNameAuthorship: Cresson, 1926; **Location:** islandGroup: Hawaiian Islands; island: Kauai; verbatimLocality: Makaleha Stream, at Makaleha Springs; minimumElevationInMeters: 787; **Identification:** identifiedBy: WN Mathis; dateIdentified: 1992; **Event:** verbatimEventDate: 08.xi.1990; **Record Level:** institutionCode: BPBM
- **Type status:** Other material. **Occurrence:** catalogNumber: 2006005162; recordedBy: DA Polhemus; lifeStage: adult; **Taxon:** kingdom: Animalia; phylum: Arthropoda; class: Insecta; order: Diptera; family: Canacidae; genus: Procanace; specificEpithet: Procanacenigroviridis; scientificNameAuthorship: Cresson, 1926; **Location:** islandGroup: Hawaiian Islands; island: Kauai; verbatimLocality: Makaleha Stream, at Makaleha Springs; minimumElevationInMeters: 787; **Identification:** identifiedBy: WN Mathis; dateIdentified: 1992; **Event:** verbatimEventDate: 08.xi.1990; **Record Level:** institutionCode: BPBM
- **Type status:** Other material. **Occurrence:** catalogNumber: 2006005153; recordedBy: DA Polhemus; lifeStage: adult; **Taxon:** kingdom: Animalia; phylum: Arthropoda; class: Insecta; order: Diptera; family: Canacidae; genus: Procanace; specificEpithet: Procanacenigroviridis; scientificNameAuthorship: Cresson, 1926; **Location:** islandGroup: Hawaiian Islands; island: Kauai; verbatimLocality: Makaleha Stream, at Makaleha Springs; minimumElevationInMeters: 787; **Identification:** identifiedBy: WN Mathis; dateIdentified: 1992; **Event:** verbatimEventDate: 08.xi.1990; **Record Level:** institutionCode: BPBM
- **Type status:** Other material. **Occurrence:** catalogNumber: 2006005164; recordedBy: DA Polhemus; lifeStage: adult; **Taxon:** kingdom: Animalia; phylum: Arthropoda; class: Insecta; order: Diptera; family: Canacidae; genus: Procanace; specificEpithet: Procanacenigroviridis; scientificNameAuthorship: Cresson, 1926; **Location:** islandGroup: Hawaiian Islands; island: Kauai; verbatimLocality: Makaleha Stream, at Makaleha Springs; minimumElevationInMeters: 787; **Identification:** identifiedBy: WN Mathis; dateIdentified: 1992; **Event:** verbatimEventDate: 08.xi.1990; **Record Level:** institutionCode: BPBM
- **Type status:** Other material. **Occurrence:** catalogNumber: 2006005165; recordedBy: DA Polhemus; lifeStage: adult; **Taxon:** kingdom: Animalia; phylum: Arthropoda; class: Insecta; order: Diptera; family: Canacidae; genus: Procanace; specificEpithet: Procanacenigroviridis; scientificNameAuthorship: Cresson, 1926; **Location:** islandGroup: Hawaiian Islands; island: Kauai; verbatimLocality: Makaleha Stream, at Makaleha Springs; minimumElevationInMeters: 787; **Identification:** identifiedBy: WN Mathis; dateIdentified: 1992; **Event:** verbatimEventDate: 08.xi.1990; **Record Level:** institutionCode: BPBM
- **Type status:** Other material. **Occurrence:** catalogNumber: 2006005161; recordedBy: DA Polhemus; lifeStage: adult; **Taxon:** kingdom: Animalia; phylum: Arthropoda; class: Insecta; order: Diptera; family: Canacidae; genus: Procanace; specificEpithet: Procanacenigroviridis; scientificNameAuthorship: Cresson, 1926; **Location:** islandGroup: Hawaiian Islands; island: Kauai; verbatimLocality: Makaleha Stream, at Makaleha Springs; minimumElevationInMeters: 787; **Identification:** identifiedBy: WN Mathis; dateIdentified: 1992; **Event:** verbatimEventDate: 08.xi.1990; **Record Level:** institutionCode: BPBM
- **Type status:** Other material. **Occurrence:** catalogNumber: 2006005155; recordedBy: DA Polhemus; lifeStage: adult; **Taxon:** kingdom: Animalia; phylum: Arthropoda; class: Insecta; order: Diptera; family: Canacidae; genus: Procanace; specificEpithet: Procanacenigroviridis; scientificNameAuthorship: Cresson, 1926; **Location:** islandGroup: Hawaiian Islands; island: Kauai; verbatimLocality: Makaleha Stream, at Makaleha Springs; minimumElevationInMeters: 787; **Identification:** identifiedBy: WN Mathis; dateIdentified: 1992; **Event:** verbatimEventDate: 08.xi.1990; **Record Level:** institutionCode: BPBM
- **Type status:** Other material. **Occurrence:** catalogNumber: 2006005154; recordedBy: DA Polhemus; lifeStage: adult; **Taxon:** kingdom: Animalia; phylum: Arthropoda; class: Insecta; order: Diptera; family: Canacidae; genus: Procanace; specificEpithet: Procanacenigroviridis; scientificNameAuthorship: Cresson, 1926; **Location:** islandGroup: Hawaiian Islands; island: Kauai; verbatimLocality: Makaleha Stream, at Makaleha Springs; minimumElevationInMeters: 787; **Identification:** identifiedBy: WN Mathis; dateIdentified: 1992; **Event:** verbatimEventDate: 08.xi.1990; **Record Level:** institutionCode: BPBM
- **Type status:** Other material. **Occurrence:** recordedBy: PM O'Grady, RT Lapoint, GM Bennett, B Ort, NA Pantoja; lifeStage: adult; **Taxon:** kingdom: Animalia; phylum: Arthropoda; class: Insecta; order: Diptera; family: Canacidae; genus: Procanace; specificEpithet: Procanacenigroviridis; scientificNameAuthorship: Cresson, 1926; **Location:** islandGroup: Hawaiian Islands; island: Kauai; verbatimLocality: Kokee Stream near Canyon Trail; **Identification:** identifiedBy: PM O'Grady; dateIdentified: 2014; **Event:** verbatimEventDate: 10.i.2010; **Record Level:** institutionCode: EMEC; collectionCode: 205648
- **Type status:** Other material. **Occurrence:** recordedBy: PM O'Grady, BS Ort, RT Lapoint, GM Bennett; lifeStage: adult; **Taxon:** kingdom: Animalia; phylum: Arthropoda; class: Insecta; order: Diptera; family: Canacidae; genus: Procanace; specificEpithet: Procanacenigroviridis; scientificNameAuthorship: Cresson, 1926; **Location:** islandGroup: Hawaiian Islands; island: Kauai; verbatimLocality: Stream #1, Trail along North Coast; **Identification:** identifiedBy: PM O'Grady; dateIdentified: 2014; **Event:** verbatimEventDate: 11.i.2010; **Record Level:** institutionCode: EMEC; collectionCode: 205647
- **Type status:** Other material. **Occurrence:** recordedBy: PM O'Grady, BS Ort, RT Lapoint, GM Bennett; lifeStage: adult; **Taxon:** kingdom: Animalia; phylum: Arthropoda; class: Insecta; order: Diptera; family: Canacidae; genus: Procanace; specificEpithet: Procanacenigroviridis; scientificNameAuthorship: Cresson, 1926; **Location:** islandGroup: Hawaiian Islands; island: Kauai; verbatimLocality: Hanapakai Stream; **Identification:** identifiedBy: PM O'Grady; dateIdentified: 2014; **Event:** verbatimEventDate: 11.i.2010; **Record Level:** institutionCode: EMEC; collectionCode: 205650

##### Ecological interactions

###### Native status

endemic

##### Distribution

HAWAIIAN ISLANDS: Kauai (Fig. 10​).

##### Notes

Cresson (1926), [original description]; Wirth (1951), [family revision; male genitalia (ventral and lateral), female genitalia (lateral); Hardy and Delfinado (1980), [redescription and revision of Hawaiian taxa; head (front and lateral), female terminalia (dorsal and ventral), spermathecae, wing, surstylus; cephalopharyngael skeleton (larval and pupal, puparium, third instar larvae]; Mathis (1992), [World Catalog]; Nishida (2002), [Hawaiian Arthropod Checklist].

#### Procanace quadrisetosa

Hardy and Delfinado, 1980

##### Materials

- **Type status:** Holotype. **Occurrence:** recordedBy: MD Delfinado; individualCount: 1; sex: female; lifeStage: adult; **Taxon:** kingdom: Animalia; phylum: Arthropoda; class: Insecta; order: Diptera; family: Canacidae; genus: Procanace; specificEpithet: Procanacequadrisetosa; scientificNameAuthorship: Hardy & Delfinado, 1980; **Location:** islandGroup: Hawaiian Islands; island: Kauai; verbatimLocality: Waipoo Falls, Waimea Canyon, Kokee, on wet rocks; **Identification:** identifiedBy: DE Hardy & MD Delfinado; dateIdentified: 1980; **Event:** verbatimEventDate: 2.iv.1970; **Record Level:** institutionCode: BPBM
- **Type status:** Paratype. **Occurrence:** recordedBy: MD Delfinado; individualCount: 1; sex: female; lifeStage: adult; **Taxon:** kingdom: Animalia; phylum: Arthropoda; class: Insecta; order: Diptera; family: Canacidae; genus: Procanace; specificEpithet: Procanacequadrisetosa; scientificNameAuthorship: Hardy & Delfinado, 1980; **Location:** islandGroup: Hawaiian Islands; island: Kauai; verbatimLocality: Waipoo Falls, Waimea Canyon, Kokee, on wet rocks; **Identification:** identifiedBy: DE Hardy & MD Delfinado; dateIdentified: 1980; **Event:** verbatimEventDate: 2.iv.1970; **Record Level:** institutionCode: UHM
- **Type status:** Paratype. **Occurrence:** recordedBy: MD Delfinado; individualCount: 2; lifeStage: adult; **Taxon:** kingdom: Animalia; phylum: Arthropoda; class: Insecta; order: Diptera; family: Canacidae; genus: Procanace; specificEpithet: Procanacequadrisetosa; scientificNameAuthorship: Hardy & Delfinado, 1980; **Location:** islandGroup: Hawaiian Islands; island: Kauai; verbatimLocality: Wailua Falls; **Identification:** identifiedBy: DE Hardy & MD Delfinado; dateIdentified: 1980; **Event:** verbatimEventDate: 4.iv.1970; **Record Level:** institutionCode: UHM
- **Type status:** Paratype. **Occurrence:** recordedBy: MD Delfinado; individualCount: 1; sex: female; lifeStage: adult; **Taxon:** kingdom: Animalia; phylum: Arthropoda; class: Insecta; order: Diptera; family: Canacidae; genus: Procanace; specificEpithet: Procanacequadrisetosa; scientificNameAuthorship: Hardy & Delfinado, 1980; **Location:** islandGroup: Hawaiian Islands; island: Kauai; verbatimLocality: Wailua Falls, on wet rocks; **Identification:** identifiedBy: DE Hardy & MD Delfinado; dateIdentified: 1980; **Event:** verbatimEventDate: 4.iv.1970; **Record Level:** institutionCode: UHM
- **Type status:** Other material. **Occurrence:** recordedBy: PM O'Grady, RT Lapoint, GM Bennett, B Ort, NA Pantoja; lifeStage: adult; **Taxon:** kingdom: Animalia; phylum: Arthropoda; class: Insecta; order: Diptera; family: Canacidae; genus: Procanace; specificEpithet: Procanacequadrisetosa; scientificNameAuthorship: Hardy & Delfinado, 1980; **Location:** islandGroup: Hawaiian Islands; verbatimLocality: Kokee Stream near Canyon Trail; **Identification:** identifiedBy: PM O'Grady; dateIdentified: 2014; **Event:** verbatimEventDate: 10.i.2010; **Record Level:** institutionCode: EMEC; collectionCode: 588.8

##### Ecological interactions

###### Native status

endemic

##### Distribution

HAWAIIAN ISLANDS: Kauai (Fig. 12​).

##### Notes

Hardy and Delfinado (1980), [original description; head (front and lateral), female terminalia (dorsal and ventral), spermathecae, wing, surstylus; cephalopharyngael skeleton (larval and pupal, puparium, third instar larvae]; Mathis (1992), [World Catalog]; Nishida (2002), [Hawaiian Arthropod Checklist].

#### Procanace williamsi

Wirth, 1951

##### Materials

- **Type status:** Holotype. **Occurrence:** recordedBy: WW Wirth; individualCount: 1; sex: male; lifeStage: adult; **Taxon:** kingdom: Animalia; phylum: Arthropoda; class: Insecta; order: Diptera; family: Canacidae; genus: Procanace; specificEpithet: Procanacewilliamsi; scientificNameAuthorship: Wirth, 1951; **Location:** islandGroup: Hawaiian Islands; island: Oahu; verbatimLocality: Kalihi, light trap near shore; **Identification:** identifiedBy: DE Hardy & MD Delfinado; dateIdentified: 1980; **Event:** verbatimEventDate: 11.v.1946; **Record Level:** institutionCode: USNM
- **Type status:** Paratype. **Occurrence:** recordedBy: WW Wirth; individualCount: 1; sex: female; lifeStage: adult; **Taxon:** kingdom: Animalia; phylum: Arthropoda; class: Insecta; order: Diptera; family: Canacidae; genus: Procanace; specificEpithet: Procanacewilliamsi; scientificNameAuthorship: Wirth, 1951; **Location:** islandGroup: Hawaiian Islands; verbatimLocality: from plane; **Identification:** identifiedBy: DE Hardy & MD Delfinado; dateIdentified: 1980; **Event:** verbatimEventDate: 8.xi.1944; **Record Level:** institutionCode: UHM
- **Type status:** Paratype. **Occurrence:** catalogNumber: 59965; recordedBy: WW Wirth; individualCount: 1; sex: male; lifeStage: adult; **Taxon:** kingdom: Animalia; phylum: Arthropoda; class: Insecta; order: Diptera; family: Canacidae; genus: Procanace; specificEpithet: Procanacewilliamsi; scientificNameAuthorship: Wirth, 1951; **Location:** islandGroup: Hawaiian Islands; verbatimLocality: from plane; **Identification:** identifiedBy: DE Hardy & MD Delfinado; dateIdentified: 1980; **Event:** verbatimEventDate: 8.xi.1944; **Record Level:** institutionCode: USNM
- **Type status:** Other material. **Occurrence:** recordedBy: MS Adachi; individualCount: 5; lifeStage: adult; **Taxon:** kingdom: Animalia; phylum: Arthropoda; class: Insecta; order: Diptera; family: Canacidae; genus: Procanace; specificEpithet: Procanacewilliamsi; scientificNameAuthorship: Wirth, 1951; **Location:** islandGroup: Hawaiian Islands; island: Oahu; verbatimLocality: Waikiki; **Event:** verbatimEventDate: 16.iv.1950; **Record Level:** institutionCode: UHM
- **Type status:** Other material. **Occurrence:** recordedBy: MS Adachi; individualCount: 2; sex: 2 males; lifeStage: adult; **Taxon:** kingdom: Animalia; phylum: Arthropoda; class: Insecta; order: Diptera; family: Canacidae; genus: Procanace; specificEpithet: Procanacewilliamsi; scientificNameAuthorship: Wirth, 1951; **Location:** islandGroup: Hawaiian Islands; island: Oahu; verbatimLocality: Waikiki; **Event:** verbatimEventDate: 16.iv.1950; **Record Level:** institutionCode: USNM
- **Type status:** Other material. **Occurrence:** recordedBy: MS Adachi; individualCount: 13; lifeStage: adult; **Taxon:** kingdom: Animalia; phylum: Arthropoda; class: Insecta; order: Diptera; family: Canacidae; genus: Procanace; specificEpithet: Procanacewilliamsi; scientificNameAuthorship: Wirth, 1951; **Location:** islandGroup: Hawaiian Islands; island: Oahu; verbatimLocality: Waikiki; **Event:** verbatimEventDate: i.1952; **Record Level:** institutionCode: UHM
- **Type status:** Other material. **Occurrence:** recordedBy: MS Adachi; individualCount: 22; lifeStage: adult; **Taxon:** kingdom: Animalia; phylum: Arthropoda; class: Insecta; order: Diptera; family: Canacidae; genus: Procanace; specificEpithet: Procanacewilliamsi; scientificNameAuthorship: Wirth, 1951; **Location:** islandGroup: Hawaiian Islands; island: Oahu; verbatimLocality: Ala Wai Canal; **Event:** verbatimEventDate: vi.1952; **Record Level:** institutionCode: UHM
- **Type status:** Other material. **Occurrence:** recordedBy: I Miyagi; individualCount: 2; sex: female; lifeStage: adult; **Taxon:** kingdom: Animalia; phylum: Arthropoda; class: Insecta; order: Diptera; family: Canacidae; genus: Procanace; specificEpithet: Procanacewilliamsi; scientificNameAuthorship: Wirth, 1951; **Location:** islandGroup: Japan; island: Shikoku; verbatimLocality: Ehime Prefecture, Matsuyama; **Event:** verbatimEventDate: 4.x.1961; **Record Level:** institutionCode: EIHU
- **Type status:** Other material. **Occurrence:** recordedBy: I Miyagi; individualCount: 4; sex: male; lifeStage: adult; **Taxon:** kingdom: Animalia; phylum: Arthropoda; class: Insecta; order: Diptera; family: Canacidae; genus: Procanace; specificEpithet: Procanacewilliamsi; scientificNameAuthorship: Wirth, 1951; **Location:** islandGroup: Japan; island: Kyushu; verbatimLocality: Kagoshima Prefecture, Ibusuki; **Event:** verbatimEventDate: 17.x.1961; **Record Level:** institutionCode: EIHU
- **Type status:** Other material. **Occurrence:** recordedBy: I Miyagi; individualCount: 3; sex: female; lifeStage: adult; **Taxon:** kingdom: Animalia; phylum: Arthropoda; class: Insecta; order: Diptera; family: Canacidae; genus: Procanace; specificEpithet: Procanacewilliamsi; scientificNameAuthorship: Wirth, 1951; **Location:** islandGroup: Japan; island: Kyushu; verbatimLocality: Kagoshima Prefecture, Ibusuki; **Event:** verbatimEventDate: 17.x.1961; **Record Level:** institutionCode: EIHU
- **Type status:** Other material. **Occurrence:** recordedBy: JW Beardsley; individualCount: 1; lifeStage: adult; **Taxon:** kingdom: Animalia; phylum: Arthropoda; class: Insecta; order: Diptera; family: Canacidae; genus: Procanace; specificEpithet: Procanacewilliamsi; scientificNameAuthorship: Wirth, 1951; **Location:** islandGroup: Hawaiian Islands; island: Oahu; verbatimLocality: Ewa; verbatimElevation: light trap; **Event:** verbatimEventDate: xi.1962; **Record Level:** institutionCode: UHM
- **Type status:** Other material. **Occurrence:** recordedBy: I Miyagi; individualCount: 5; sex: female; lifeStage: adult; **Taxon:** kingdom: Animalia; phylum: Arthropoda; class: Insecta; order: Diptera; family: Canacidae; genus: Procanace; specificEpithet: Procanacewilliamsi; scientificNameAuthorship: Wirth, 1951; **Location:** islandGroup: Japan; island: Shikoku; verbatimLocality: Ehime Prefecture, Nagahama; **Event:** verbatimEventDate: 8.ix.1962; **Record Level:** institutionCode: EIHU
- **Type status:** Other material. **Occurrence:** recordedBy: I Miyagi; individualCount: 6; sex: male; lifeStage: adult; **Taxon:** kingdom: Animalia; phylum: Arthropoda; class: Insecta; order: Diptera; family: Canacidae; genus: Procanace; specificEpithet: Procanacewilliamsi; scientificNameAuthorship: Wirth, 1951; **Location:** islandGroup: Japan; island: Shikoku; verbatimLocality: Ehime Prefecture, Nagahama; **Event:** verbatimEventDate: 8.ix.1962; **Record Level:** institutionCode: EIHU
- **Type status:** Other material. **Occurrence:** recordedBy: I Miyagi; individualCount: 20; sex: female; lifeStage: adult; **Taxon:** kingdom: Animalia; phylum: Arthropoda; class: Insecta; order: Diptera; family: Canacidae; genus: Procanace; specificEpithet: Procanacewilliamsi; scientificNameAuthorship: Wirth, 1951; **Location:** islandGroup: Japan; island: Shikoku; verbatimLocality: Ehime Prefecture, Uwajima; **Event:** verbatimEventDate: 9.ix.1962; **Record Level:** institutionCode: EIHU
- **Type status:** Other material. **Occurrence:** recordedBy: I Miyagi; individualCount: 20; sex: male; lifeStage: adult; **Taxon:** kingdom: Animalia; phylum: Arthropoda; class: Insecta; order: Diptera; family: Canacidae; genus: Procanace; specificEpithet: Procanacewilliamsi; scientificNameAuthorship: Wirth, 1951; **Location:** islandGroup: Japan; island: Shikoku; verbatimLocality: Ehime Prefecture, Uwajima; **Event:** verbatimEventDate: 9.ix.1962; **Record Level:** institutionCode: EIHU
- **Type status:** Other material. **Occurrence:** recordedBy: I Miyagi; individualCount: 10; sex: female; lifeStage: adult; **Taxon:** kingdom: Animalia; phylum: Arthropoda; class: Insecta; order: Diptera; family: Canacidae; genus: Procanace; specificEpithet: Procanacewilliamsi; scientificNameAuthorship: Wirth, 1951; **Location:** islandGroup: Japan; island: Honshu; verbatimLocality: Sizuoka Prefecture, Izu; **Event:** verbatimEventDate: 20.vii.1963; **Record Level:** institutionCode: EIHU
- **Type status:** Other material. **Occurrence:** recordedBy: I Miyagi; individualCount: 5; sex: male; lifeStage: adult; **Taxon:** kingdom: Animalia; phylum: Arthropoda; class: Insecta; order: Diptera; family: Canacidae; genus: Procanace; specificEpithet: Procanacewilliamsi; scientificNameAuthorship: Wirth, 1951; **Location:** islandGroup: Japan; island: Honshu; verbatimLocality: Sizuoka Prefecture, Izu; **Event:** verbatimEventDate: 20.vii.1963; **Record Level:** institutionCode: EIHU
- **Type status:** Other material. **Occurrence:** recordedBy: I Miyagi; individualCount: 10; sex: female; lifeStage: adult; **Taxon:** kingdom: Animalia; phylum: Arthropoda; class: Insecta; order: Diptera; family: Canacidae; genus: Procanace; specificEpithet: Procanacewilliamsi; scientificNameAuthorship: Wirth, 1951; **Location:** islandGroup: Japan; island: Honshu; verbatimLocality: Sizuoka Prefecture, Omaezaki; **Event:** verbatimEventDate: 22.vii.1963; **Record Level:** institutionCode: EIHU
- **Type status:** Other material. **Occurrence:** recordedBy: I Miyagi; individualCount: 5; sex: male; lifeStage: adult; **Taxon:** kingdom: Animalia; phylum: Arthropoda; class: Insecta; order: Diptera; family: Canacidae; genus: Procanace; specificEpithet: Procanacewilliamsi; scientificNameAuthorship: Wirth, 1951; **Location:** islandGroup: Japan; island: Honshu; verbatimLocality: Sizuoka Prefecture, Omaezaki; **Event:** verbatimEventDate: 22.vii.1963; **Record Level:** institutionCode: EIHU
- **Type status:** Other material. **Occurrence:** recordedBy: I Miyagi; individualCount: 7; sex: female; lifeStage: adult; **Taxon:** kingdom: Animalia; phylum: Arthropoda; class: Insecta; order: Diptera; family: Canacidae; genus: Procanace; specificEpithet: Procanacewilliamsi; scientificNameAuthorship: Wirth, 1951; **Location:** islandGroup: Japan; island: Kyushu; verbatimLocality: Tsushima; **Event:** verbatimEventDate: 6.viii.1963; **Record Level:** institutionCode: EIHU
- **Type status:** Other material. **Occurrence:** recordedBy: I Miyagi; individualCount: 12; sex: male; lifeStage: adult; **Taxon:** kingdom: Animalia; phylum: Arthropoda; class: Insecta; order: Diptera; family: Canacidae; genus: Procanace; specificEpithet: Procanacewilliamsi; scientificNameAuthorship: Wirth, 1951; **Location:** islandGroup: Japan; island: Kyushu; verbatimLocality: Tsushima; **Event:** verbatimEventDate: 6.viii.1963; **Record Level:** institutionCode: EIHU
- **Type status:** Other material. **Occurrence:** recordedBy: I Miyagi; individualCount: 20; sex: female; lifeStage: adult; **Taxon:** kingdom: Animalia; phylum: Arthropoda; class: Insecta; order: Diptera; family: Canacidae; genus: Procanace; specificEpithet: Procanacewilliamsi; scientificNameAuthorship: Wirth, 1951; **Location:** islandGroup: Japan; island: Kyushu; verbatimLocality: Yakushima; **Event:** verbatimEventDate: 13.viii.1963; **Record Level:** institutionCode: EIHU
- **Type status:** Other material. **Occurrence:** recordedBy: I Miyagi; individualCount: 13; sex: male; lifeStage: adult; **Taxon:** kingdom: Animalia; phylum: Arthropoda; class: Insecta; order: Diptera; family: Canacidae; genus: Procanace; specificEpithet: Procanacewilliamsi; scientificNameAuthorship: Wirth, 1951; **Location:** islandGroup: Japan; island: Kyushu; verbatimLocality: Yakushima; **Event:** verbatimEventDate: 13.viii.1963; **Record Level:** institutionCode: EIHU
- **Type status:** Other material. **Occurrence:** recordedBy: no collector given; individualCount: 3; lifeStage: adult; **Taxon:** kingdom: Animalia; phylum: Arthropoda; class: Insecta; order: Diptera; family: Canacidae; genus: Procanace; specificEpithet: Procanacewilliamsi; scientificNameAuthorship: Wirth, 1951; **Location:** islandGroup: Hawaiian Islands; island: Oahu; verbatimLocality: Kaneohe Fire Station; **Event:** verbatimEventDate: 28.iii.1966; **Record Level:** institutionCode: UHM
- **Type status:** Other material. **Occurrence:** recordedBy: JR Vockeroth; individualCount: 4; sex: 2 males, 2 females; lifeStage: adult; **Taxon:** kingdom: Animalia; phylum: Arthropoda; class: Insecta; order: Diptera; family: Canacidae; genus: Procanace; specificEpithet: Procanacewilliamsi; scientificNameAuthorship: Wirth, 1951; **Location:** islandGroup: Hawaiian Islands; island: Oahu; verbatimLocality: Honolulu, brackish pond on coral Ala Moana; **Identification:** identifiedBy: W Mathis; **Event:** eventDate: 25.ix.1996; verbatimEventDate: 25.iv.1966; **Record Level:** institutionCode: USNM
- **Type status:** Other material. **Occurrence:** recordedBy: JR Vockeroth; individualCount: 4; sex: 4 females; lifeStage: adult; **Taxon:** kingdom: Animalia; phylum: Arthropoda; class: Insecta; order: Diptera; family: Canacidae; genus: Procanace; specificEpithet: Procanacewilliamsi; scientificNameAuthorship: Wirth, 1951; **Location:** islandGroup: Hawaiian Islands; island: Oahu; verbatimLocality: Honolulu, brackish pond on coral Ala Moana; **Identification:** identifiedBy: W Mathis; **Event:** eventDate: 8.i.1996; verbatimEventDate: 25.iv.1966; **Record Level:** institutionCode: USNM
- **Type status:** Other material. **Occurrence:** recordedBy: JA Tenorio, MD Dedlinado; individualCount: 33; lifeStage: adult; **Taxon:** kingdom: Animalia; phylum: Arthropoda; class: Insecta; order: Diptera; family: Canacidae; genus: Procanace; specificEpithet: Procanacewilliamsi; scientificNameAuthorship: Wirth, 1951; **Location:** islandGroup: Hawaiian Islands; island: Oahu; verbatimLocality: Ala Wai Canal; **Event:** verbatimEventDate: 22.v.1970; **Record Level:** institutionCode: UHM
- **Type status:** Other material. **Occurrence:** recordedBy: SL Montgomery; individualCount: 45; lifeStage: adult; **Taxon:** kingdom: Animalia; phylum: Arthropoda; class: Insecta; order: Diptera; family: Canacidae; genus: Procanace; specificEpithet: Procanacewilliamsi; scientificNameAuthorship: Wirth, 1951; **Location:** islandGroup: Hawaiian Islands; island: Oahu; verbatimLocality: Kahana Stream Estuary; **Event:** verbatimEventDate: 26.v.1970; **Record Level:** institutionCode: UHM
- **Type status:** Other material. **Occurrence:** recordedBy: L Teremoto, L Uyenishi; individualCount: 39; lifeStage: adult; **Taxon:** kingdom: Animalia; phylum: Arthropoda; class: Insecta; order: Diptera; family: Canacidae; genus: Procanace; specificEpithet: Procanacewilliamsi; scientificNameAuthorship: Wirth, 1951; **Location:** islandGroup: Hawaiian Islands; island: Oahu; verbatimLocality: Laiemaloo Bridge, Laie; **Event:** verbatimEventDate: 26.v.1970; **Record Level:** institutionCode: UHM
- **Type status:** Other material. **Occurrence:** recordedBy: JA Tenorio; individualCount: 1; lifeStage: adult; **Taxon:** kingdom: Animalia; phylum: Arthropoda; class: Insecta; order: Diptera; family: Canacidae; genus: Procanace; specificEpithet: Procanacewilliamsi; scientificNameAuthorship: Wirth, 1951; **Location:** islandGroup: Hawaiian Islands; island: Oahu; verbatimLocality: Waimea Bay, under bridge; **Event:** verbatimEventDate: 26.v.1970; **Record Level:** institutionCode: UHM
- **Type status:** Other material. **Occurrence:** recordedBy: JA Tenorio; individualCount: 53; lifeStage: adult; **Taxon:** kingdom: Animalia; phylum: Arthropoda; class: Insecta; order: Diptera; family: Canacidae; genus: Procanace; specificEpithet: Procanacewilliamsi; scientificNameAuthorship: Wirth, 1951; **Location:** islandGroup: Hawaiian Islands; island: Oahu; verbatimLocality: Oneawa Canal, Kailua; **Event:** verbatimEventDate: 31.v.1970; **Record Level:** institutionCode: UHM
- **Type status:** Other material. **Occurrence:** catalogNumber: 2008000695; recordedBy: DJ Preston; lifeStage: adult; **Taxon:** kingdom: Animalia; phylum: Arthropoda; class: Insecta; order: Diptera; family: Canacidae; genus: Procanace; specificEpithet: Procanacewilliamsi; scientificNameAuthorship: Wirth, 1951; **Location:** islandGroup: Hawaiian Islands; island: Oahu; verbatimLocality: Pearl Harbor, Honouliuli Stream, near pipeline bridge; **Identification:** identifiedBy: K Arakaki; **Event:** verbatimEventDate: 19.xi.1997; eventRemarks: Pearl Harbor Survey; **Record Level:** institutionCode: BPBM
- **Type status:** Other material. **Occurrence:** catalogNumber: 2008000693; recordedBy: DJ Preston, R Englund, R Wolfe; lifeStage: adult; **Taxon:** kingdom: Animalia; phylum: Arthropoda; class: Insecta; order: Diptera; family: Canacidae; genus: Procanace; specificEpithet: Procanacewilliamsi; scientificNameAuthorship: Wirth, 1951; **Location:** islandGroup: Hawaiian Islands; island: Oahu; verbatimLocality: Pearl Harbor, Honouliuli Stream, near pipeline bridge; **Identification:** identifiedBy: K Arakaki; **Event:** verbatimEventDate: 19.xi.1997; eventRemarks: Pearl Harbor Survey; **Record Level:** institutionCode: BPBM
- **Type status:** Other material. **Occurrence:** catalogNumber: 2008000694; recordedBy: DJ Preston, R Englund, R Wolfe; lifeStage: adult; **Taxon:** kingdom: Animalia; phylum: Arthropoda; class: Insecta; order: Diptera; family: Canacidae; genus: Procanace; specificEpithet: Procanacewilliamsi; scientificNameAuthorship: Wirth, 1951; **Location:** islandGroup: Hawaiian Islands; island: Oahu; verbatimLocality: Pearl Harbor, Honouliuli Stream, near pipeline bridge; **Identification:** identifiedBy: K Arakaki; **Event:** verbatimEventDate: 19.xi.1997; eventRemarks: Pearl Harbor Survey; **Record Level:** institutionCode: BPBM
- **Type status:** Other material. **Occurrence:** catalogNumber: 2008000548; recordedBy: DJ Preston, R Englund, R Wolfe; lifeStage: adult; **Taxon:** kingdom: Animalia; phylum: Arthropoda; class: Insecta; order: Diptera; family: Canacidae; genus: Procanace; specificEpithet: Procanacewilliamsi; scientificNameAuthorship: Wirth, 1951; **Location:** islandGroup: Hawaiian Islands; island: Oahu; verbatimLocality: Pearl Harbor, Honouliuli Stream, near pipeline bridge; **Identification:** identifiedBy: K Arakaki; **Event:** verbatimEventDate: 19.xi.1997; eventRemarks: Pearl Harbor Survey; **Record Level:** institutionCode: BPBM
- **Type status:** Other material. **Occurrence:** catalogNumber: 2008000708; recordedBy: DJ Preston; lifeStage: adult; **Taxon:** kingdom: Animalia; phylum: Arthropoda; class: Insecta; order: Diptera; family: Canacidae; genus: Procanace; specificEpithet: Procanacewilliamsi; scientificNameAuthorship: Wirth, 1951; **Location:** islandGroup: Hawaiian Islands; island: Oahu; verbatimLocality: Pearl Harbor, Halawa Stream, at Salt Lake Blvd.; **Identification:** identifiedBy: K Arakaki; **Event:** verbatimEventDate: 08.xii.1997; eventRemarks: Pearl Harbor Survey; **Record Level:** institutionCode: BPBM
- **Type status:** Other material. **Occurrence:** catalogNumber: 2008000709; recordedBy: DJ Preston, R Englund, R Wolfe; lifeStage: adult; **Taxon:** kingdom: Animalia; phylum: Arthropoda; class: Insecta; order: Diptera; family: Canacidae; genus: Procanace; specificEpithet: Procanacewilliamsi; scientificNameAuthorship: Wirth, 1951; **Location:** islandGroup: Hawaiian Islands; island: Oahu; verbatimLocality: Koolau Mountains, Halawa Stream, South of Salt Lake Blvd Bridge; minimumElevationInMeters: 0; maximumElevationInMeters: 16; **Identification:** identifiedBy: K Arakaki; **Event:** verbatimEventDate: 23.iii.1998; eventRemarks: Pearl Harbor Survey; **Record Level:** institutionCode: BPBM
- **Type status:** Other material. **Occurrence:** catalogNumber: 2008000760; recordedBy: K Arakaki; lifeStage: adult; **Taxon:** kingdom: Animalia; phylum: Arthropoda; class: Insecta; order: Diptera; family: Canacidae; genus: Procanace; specificEpithet: Procanacewilliamsi; scientificNameAuthorship: Wirth, 1951; **Location:** islandGroup: Hawaiian Islands; island: Oahu; verbatimLocality: Pearl Harbor, Middle Loch, near Waiawa Springs along shore; **Identification:** identifiedBy: K Arakaki; dateIdentified: 1998; **Event:** verbatimEventDate: 06.v.1998; eventRemarks: Pearl Harbor Survey; **Record Level:** institutionCode: BPBM
- **Type status:** Other material. **Occurrence:** catalogNumber: 2008000761; recordedBy: K Arakaki; lifeStage: adult; **Taxon:** kingdom: Animalia; phylum: Arthropoda; class: Insecta; order: Diptera; family: Canacidae; genus: Procanace; specificEpithet: Procanacewilliamsi; scientificNameAuthorship: Wirth, 1951; **Location:** islandGroup: Hawaiian Islands; island: Oahu; verbatimLocality: Pearl Harbor, Middle Loch, near Waiawa Springs along shore; **Identification:** identifiedBy: K Arakaki; dateIdentified: 1998; **Event:** verbatimEventDate: 06.v.1998; eventRemarks: Pearl Harbor Survey; **Record Level:** institutionCode: BPBM
- **Type status:** Other material. **Occurrence:** catalogNumber: 2008000764; recordedBy: K Arakaki; lifeStage: adult; **Taxon:** kingdom: Animalia; phylum: Arthropoda; class: Insecta; order: Diptera; family: Canacidae; genus: Procanace; specificEpithet: Procanacewilliamsi; scientificNameAuthorship: Wirth, 1951; **Location:** islandGroup: Hawaiian Islands; island: Oahu; verbatimLocality: Pearl Harbor, Middle Loch, near Waiawa Springs along shore; **Identification:** identifiedBy: K Arakaki; dateIdentified: 1998; **Event:** verbatimEventDate: 06.v.1998; eventRemarks: Pearl Harbor Survey; **Record Level:** institutionCode: BPBM
- **Type status:** Other material. **Occurrence:** catalogNumber: 2008000763; recordedBy: K Arakaki; lifeStage: adult; **Taxon:** kingdom: Animalia; phylum: Arthropoda; class: Insecta; order: Diptera; family: Canacidae; genus: Procanace; specificEpithet: Procanacewilliamsi; scientificNameAuthorship: Wirth, 1951; **Location:** islandGroup: Hawaiian Islands; island: Oahu; verbatimLocality: Pearl Harbor, Middle Loch, near Waiawa Springs along shore; **Identification:** identifiedBy: K Arakaki; dateIdentified: 1998; **Event:** verbatimEventDate: 06.v.1998; eventRemarks: Pearl Harbor Survey; **Record Level:** institutionCode: BPBM
- **Type status:** Other material. **Occurrence:** catalogNumber: 2008000765; recordedBy: K Arakaki; lifeStage: adult; **Taxon:** kingdom: Animalia; phylum: Arthropoda; class: Insecta; order: Diptera; family: Canacidae; genus: Procanace; specificEpithet: Procanacewilliamsi; scientificNameAuthorship: Wirth, 1951; **Location:** islandGroup: Hawaiian Islands; island: Oahu; verbatimLocality: Pearl Harbor, Middle Loch, near Waiawa Springs along shore; **Identification:** identifiedBy: K Arakaki; dateIdentified: 1998; **Event:** verbatimEventDate: 06.v.1998; eventRemarks: Pearl Harbor Survey; **Record Level:** institutionCode: BPBM
- **Type status:** Other material. **Occurrence:** catalogNumber: 2008000766; recordedBy: K Arakaki; lifeStage: adult; **Taxon:** kingdom: Animalia; phylum: Arthropoda; class: Insecta; order: Diptera; family: Canacidae; genus: Procanace; specificEpithet: Procanacewilliamsi; scientificNameAuthorship: Wirth, 1951; **Location:** islandGroup: Hawaiian Islands; island: Oahu; verbatimLocality: Pearl Harbor, Middle Loch, near Waiawa Springs along shore; **Identification:** identifiedBy: K Arakaki; dateIdentified: 1998; **Event:** verbatimEventDate: 06.v.1998; eventRemarks: Pearl Harbor Survey; **Record Level:** institutionCode: BPBM
- **Type status:** Other material. **Occurrence:** catalogNumber: 2008000767; recordedBy: K Arakaki; lifeStage: adult; **Taxon:** kingdom: Animalia; phylum: Arthropoda; class: Insecta; order: Diptera; family: Canacidae; genus: Procanace; specificEpithet: Procanacewilliamsi; scientificNameAuthorship: Wirth, 1951; **Location:** islandGroup: Hawaiian Islands; island: Oahu; verbatimLocality: Pearl Harbor, Middle Loch, near Waiawa Springs along shore; **Identification:** identifiedBy: K Arakaki; dateIdentified: 1998; **Event:** verbatimEventDate: 06.v.1998; eventRemarks: Pearl Harbor Survey; **Record Level:** institutionCode: BPBM
- **Type status:** Other material. **Occurrence:** catalogNumber: 2008000769; recordedBy: K Arakaki; lifeStage: adult; **Taxon:** kingdom: Animalia; phylum: Arthropoda; class: Insecta; order: Diptera; family: Canacidae; genus: Procanace; specificEpithet: Procanacewilliamsi; scientificNameAuthorship: Wirth, 1951; **Location:** islandGroup: Hawaiian Islands; island: Oahu; verbatimLocality: Pearl Harbor, Middle Loch, near Waiawa Springs along shore; **Identification:** identifiedBy: K Arakaki; dateIdentified: 1998; **Event:** verbatimEventDate: 06.v.1998; eventRemarks: Pearl Harbor Survey; **Record Level:** institutionCode: BPBM
- **Type status:** Other material. **Occurrence:** catalogNumber: 2008000768; recordedBy: K Arakaki; lifeStage: adult; **Taxon:** kingdom: Animalia; phylum: Arthropoda; class: Insecta; order: Diptera; family: Canacidae; genus: Procanace; specificEpithet: Procanacewilliamsi; scientificNameAuthorship: Wirth, 1951; **Location:** islandGroup: Hawaiian Islands; island: Oahu; verbatimLocality: Pearl Harbor, Middle Loch, near Waiawa Springs along shore; **Identification:** identifiedBy: K Arakaki; dateIdentified: 1998; **Event:** verbatimEventDate: 06.v.1998; eventRemarks: Pearl Harbor Survey; **Record Level:** institutionCode: BPBM
- **Type status:** Other material. **Occurrence:** catalogNumber: 2008000762; recordedBy: K Arakaki; lifeStage: adult; **Taxon:** kingdom: Animalia; phylum: Arthropoda; class: Insecta; order: Diptera; family: Canacidae; genus: Procanace; specificEpithet: Procanacewilliamsi; scientificNameAuthorship: Wirth, 1951; **Location:** islandGroup: Hawaiian Islands; island: Oahu; verbatimLocality: Pearl Harbor, Middle Loch, near Waiawa Springs along shore; **Identification:** identifiedBy: K Arakaki; dateIdentified: 1998; **Event:** verbatimEventDate: 06.v.1998; eventRemarks: Pearl Harbor Survey; **Record Level:** institutionCode: BPBM
- **Type status:** Other material. **Occurrence:** catalogNumber: 2008000770; recordedBy: K Arakaki; lifeStage: adult; **Taxon:** kingdom: Animalia; phylum: Arthropoda; class: Insecta; order: Diptera; family: Canacidae; genus: Procanace; specificEpithet: Procanacewilliamsi; scientificNameAuthorship: Wirth, 1951; **Location:** islandGroup: Hawaiian Islands; island: Oahu; verbatimLocality: Pearl Harbor, Middle Loch, near Waiawa Springs along shore; **Identification:** identifiedBy: K Arakaki; dateIdentified: 1998; **Event:** verbatimEventDate: 06.v.1998; eventRemarks: Pearl Harbor Survey; **Record Level:** institutionCode: BPBM
- **Type status:** Other material. **Occurrence:** catalogNumber: 2008000773; recordedBy: GA Samuelson; lifeStage: adult; **Taxon:** kingdom: Animalia; phylum: Arthropoda; class: Insecta; order: Diptera; family: Canacidae; genus: Procanace; specificEpithet: Procanacewilliamsi; scientificNameAuthorship: Wirth, 1951; **Location:** islandGroup: Hawaiian Islands; island: Oahu; verbatimLocality: Pearl Harbor, Middle Loch, near Waiawa Springs along shore; **Identification:** identifiedBy: K Arakaki; dateIdentified: 1998; **Event:** verbatimEventDate: 06.v.1998; eventRemarks: Pearl Harbor Survey; **Record Level:** institutionCode: BPBM
- **Type status:** Other material. **Occurrence:** catalogNumber: 2008000772; recordedBy: K Arakaki; lifeStage: adult; **Taxon:** kingdom: Animalia; phylum: Arthropoda; class: Insecta; order: Diptera; family: Canacidae; genus: Procanace; specificEpithet: Procanacewilliamsi; scientificNameAuthorship: Wirth, 1951; **Location:** islandGroup: Hawaiian Islands; island: Oahu; verbatimLocality: Pearl Harbor, Middle Loch, near Waiawa Springs along shore; **Identification:** identifiedBy: K Arakaki; dateIdentified: 1998; **Event:** verbatimEventDate: 06.v.1998; eventRemarks: Pearl Harbor Survey; **Record Level:** institutionCode: BPBM
- **Type status:** Other material. **Occurrence:** catalogNumber: 2008000771; recordedBy: K Arakaki; lifeStage: adult; **Taxon:** kingdom: Animalia; phylum: Arthropoda; class: Insecta; order: Diptera; family: Canacidae; genus: Procanace; specificEpithet: Procanacewilliamsi; scientificNameAuthorship: Wirth, 1951; **Location:** islandGroup: Hawaiian Islands; island: Oahu; verbatimLocality: Pearl Harbor, Middle Loch, near Waiawa Springs along shore; **Identification:** identifiedBy: K Arakaki; dateIdentified: 1998; **Event:** verbatimEventDate: 06.v.1998; eventRemarks: Pearl Harbor Survey; **Record Level:** institutionCode: BPBM
- **Type status:** Other material. **Occurrence:** catalogNumber: 2008005104; recordedBy: K Arakaki; lifeStage: adult; **Taxon:** kingdom: Animalia; phylum: Arthropoda; class: Insecta; order: Diptera; family: Canacidae; genus: Procanace; specificEpithet: Procanacewilliamsi; scientificNameAuthorship: Wirth, 1951; **Location:** islandGroup: Hawaiian Islands; island: Oahu; verbatimLocality: Pearl Harbor, Blaisdell Park, shoreline; **Identification:** identifiedBy: K Arakaki; **Event:** verbatimEventDate: 28.v.1998; eventRemarks: Pearl Harbor Survey; **Record Level:** institutionCode: BPBM
- **Type status:** Other material. **Occurrence:** catalogNumber: 2008005103; recordedBy: K Arakaki; lifeStage: adult; **Taxon:** kingdom: Animalia; phylum: Arthropoda; class: Insecta; order: Diptera; family: Canacidae; genus: Procanace; specificEpithet: Procanacewilliamsi; scientificNameAuthorship: Wirth, 1951; **Location:** islandGroup: Hawaiian Islands; island: Oahu; verbatimLocality: Pearl Harbor, Blaisdell Park, shoreline; **Identification:** identifiedBy: K Arakaki; **Event:** verbatimEventDate: 28.v.1998; eventRemarks: Pearl Harbor Survey; **Record Level:** institutionCode: BPBM
- **Type status:** Other material. **Occurrence:** catalogNumber: 2008000716; recordedBy: GA Samuelson; lifeStage: adult; **Taxon:** kingdom: Animalia; phylum: Arthropoda; class: Insecta; order: Diptera; family: Canacidae; genus: Procanace; specificEpithet: Procanacewilliamsi; scientificNameAuthorship: Wirth, 1951; **Location:** islandGroup: Hawaiian Islands; island: Oahu; verbatimLocality: Waimalu Stream, Blaisdell Park; **Identification:** identifiedBy: K Arakaki; **Event:** verbatimEventDate: 28.v.1998; eventRemarks: Pearl Harbor Survey; **Record Level:** institutionCode: BPBM
- **Type status:** Other material. **Occurrence:** catalogNumber: 2008000715; recordedBy: K Arakaki; lifeStage: adult; **Taxon:** kingdom: Animalia; phylum: Arthropoda; class: Insecta; order: Diptera; family: Canacidae; genus: Procanace; specificEpithet: Procanacewilliamsi; scientificNameAuthorship: Wirth, 1951; **Location:** islandGroup: Hawaiian Islands; island: Oahu; verbatimLocality: Waimalu Stream, Blaisdell Park; **Identification:** identifiedBy: K Arakaki; **Event:** verbatimEventDate: 28.v.1998; eventRemarks: Pearl Harbor Survey; **Record Level:** institutionCode: BPBM
- **Type status:** Other material. **Occurrence:** catalogNumber: 2008000714; recordedBy: K Arakaki; lifeStage: adult; **Taxon:** kingdom: Animalia; phylum: Arthropoda; class: Insecta; order: Diptera; family: Canacidae; genus: Procanace; specificEpithet: Procanacewilliamsi; scientificNameAuthorship: Wirth, 1951; **Location:** islandGroup: Hawaiian Islands; island: Oahu; verbatimLocality: Blaisdell Park, shoreline; **Identification:** identifiedBy: K Arakaki; **Event:** verbatimEventDate: 28.v.1998; eventRemarks: Pearl Harbor Survey; **Record Level:** institutionCode: BPBM
- **Type status:** Other material. **Occurrence:** catalogNumber: 2008000751; recordedBy: GA Samuelson, K Arakaki, K Kami; lifeStage: adult; **Taxon:** kingdom: Animalia; phylum: Arthropoda; class: Insecta; order: Diptera; family: Canacidae; genus: Procanace; specificEpithet: Procanacewilliamsi; scientificNameAuthorship: Wirth, 1951; **Location:** islandGroup: Hawaiian Islands; island: Oahu; verbatimLocality: Koolau Mountains, Halawa Stream, bridge area along stream; minimumElevationInMeters: 0; maximumElevationInMeters: 3; **Identification:** identifiedBy: K Arakaki; **Event:** verbatimEventDate: 02.vi.1998; eventRemarks: Pearl Harbor Survey; **Record Level:** institutionCode: BPBM
- **Type status:** Other material. **Occurrence:** catalogNumber: 2008000752; recordedBy: GA Samuelson, K Arakaki, K Kami; lifeStage: adult; **Taxon:** kingdom: Animalia; phylum: Arthropoda; class: Insecta; order: Diptera; family: Canacidae; genus: Procanace; specificEpithet: Procanacewilliamsi; scientificNameAuthorship: Wirth, 1951; **Location:** islandGroup: Hawaiian Islands; island: Oahu; verbatimLocality: Koolau Mountains, Halawa Stream, bridge area along stream; minimumElevationInMeters: 0; maximumElevationInMeters: 3; **Identification:** identifiedBy: K Arakaki; **Event:** verbatimEventDate: 02.vi.1998; eventRemarks: Pearl Harbor Survey; **Record Level:** institutionCode: BPBM
- **Type status:** Other material. **Occurrence:** catalogNumber: 2008000755; recordedBy: GA Samuelson, K Arakaki, K Kami; lifeStage: adult; **Taxon:** kingdom: Animalia; phylum: Arthropoda; class: Insecta; order: Diptera; family: Canacidae; genus: Procanace; specificEpithet: Procanacewilliamsi; scientificNameAuthorship: Wirth, 1951; **Location:** islandGroup: Hawaiian Islands; island: Oahu; verbatimLocality: Koolau Mountains, Halawa Stream, McDonald's Area; minimumElevationInMeters: 0; **Identification:** identifiedBy: K Arakaki; **Event:** verbatimEventDate: 02.vi.1998; eventRemarks: Pearl Harbor Survey; **Record Level:** institutionCode: BPBM
- **Type status:** Other material. **Occurrence:** catalogNumber: 2008000759; recordedBy: GA Samuelson, K Arakaki, K Kami; lifeStage: adult; **Taxon:** kingdom: Animalia; phylum: Arthropoda; class: Insecta; order: Diptera; family: Canacidae; genus: Procanace; specificEpithet: Procanacewilliamsi; scientificNameAuthorship: Wirth, 1951; **Location:** islandGroup: Hawaiian Islands; island: Oahu; verbatimLocality: Koolau Mountains, Halawa Stream, McDonald's Area; minimumElevationInMeters: 0; **Identification:** identifiedBy: K Arakaki; **Event:** verbatimEventDate: 02.vi.1998; eventRemarks: Pearl Harbor Survey; **Record Level:** institutionCode: BPBM
- **Type status:** Other material. **Occurrence:** catalogNumber: 2008000756; recordedBy: GA Samuelson, K Arakaki, K Kami; lifeStage: adult; **Taxon:** kingdom: Animalia; phylum: Arthropoda; class: Insecta; order: Diptera; family: Canacidae; genus: Procanace; specificEpithet: Procanacewilliamsi; scientificNameAuthorship: Wirth, 1951; **Location:** islandGroup: Hawaiian Islands; island: Oahu; verbatimLocality: Koolau Mountains, Halawa Stream, McDonald's Area; minimumElevationInMeters: 0; **Identification:** identifiedBy: K Arakaki; **Event:** verbatimEventDate: 02.vi.1998; eventRemarks: Pearl Harbor Survey; **Record Level:** institutionCode: BPBM
- **Type status:** Other material. **Occurrence:** catalogNumber: 2008000758; recordedBy: GA Samuelson, K Arakaki, K Kami; lifeStage: adult; **Taxon:** kingdom: Animalia; phylum: Arthropoda; class: Insecta; order: Diptera; family: Canacidae; genus: Procanace; specificEpithet: Procanacewilliamsi; scientificNameAuthorship: Wirth, 1951; **Location:** islandGroup: Hawaiian Islands; island: Oahu; verbatimLocality: Koolau Mountains, Halawa Stream, McDonald's Area; minimumElevationInMeters: 0; **Identification:** identifiedBy: K Arakaki; **Event:** verbatimEventDate: 02.vi.1998; eventRemarks: Pearl Harbor Survey; **Record Level:** institutionCode: BPBM
- **Type status:** Other material. **Occurrence:** catalogNumber: 2008000753; recordedBy: GA Samuelson, K Arakaki, K Kami; lifeStage: adult; **Taxon:** kingdom: Animalia; phylum: Arthropoda; class: Insecta; order: Diptera; family: Canacidae; genus: Procanace; specificEpithet: Procanacewilliamsi; scientificNameAuthorship: Wirth, 1951; **Location:** islandGroup: Hawaiian Islands; island: Oahu; verbatimLocality: Koolau Mountains, Halawa Stream (lower), under bridge; minimumElevationInMeters: 0; **Identification:** identifiedBy: K Arakaki; **Event:** verbatimEventDate: 02.vi.1998; eventRemarks: Pearl Harbor Survey; **Record Level:** institutionCode: BPBM
- **Type status:** Other material. **Occurrence:** catalogNumber: 2008000754; recordedBy: GA Samuelson, K Arakaki, K Kami; lifeStage: adult; **Taxon:** kingdom: Animalia; phylum: Arthropoda; class: Insecta; order: Diptera; family: Canacidae; genus: Procanace; specificEpithet: Procanacewilliamsi; scientificNameAuthorship: Wirth, 1951; **Location:** islandGroup: Hawaiian Islands; island: Oahu; verbatimLocality: Koolau Mountains, Halawa Stream (upper); minimumElevationInMeters: 0; **Identification:** identifiedBy: K Arakaki; **Event:** verbatimEventDate: 02.vi.1998; eventRemarks: Pearl Harbor Survey; **Record Level:** institutionCode: BPBM
- **Type status:** Other material. **Occurrence:** catalogNumber: 2008000757; recordedBy: GA Samuelson, K Arakaki, K Kami; lifeStage: adult; **Taxon:** kingdom: Animalia; phylum: Arthropoda; class: Insecta; order: Diptera; family: Canacidae; genus: Procanace; specificEpithet: Procanacewilliamsi; scientificNameAuthorship: Wirth, 1951; **Location:** islandGroup: Hawaiian Islands; island: Oahu; verbatimLocality: Koolau Mountains, Halawa Stream, McDonald's Area; minimumElevationInMeters: 0; **Identification:** identifiedBy: K Arakaki; **Event:** verbatimEventDate: 02.vi.1998; eventRemarks: Pearl Harbor Survey; **Record Level:** institutionCode: BPBM
- **Type status:** Other material. **Occurrence:** catalogNumber: 2008000717; recordedBy: K Arakaki, GA Samuelson, K Kami; lifeStage: adult; **Taxon:** kingdom: Animalia; phylum: Arthropoda; class: Insecta; order: Diptera; family: Canacidae; genus: Procanace; specificEpithet: Procanacewilliamsi; scientificNameAuthorship: Wirth, 1951; **Location:** islandGroup: Hawaiian Islands; island: Oahu; verbatimLocality: Pearl Harbor, Waiawa Springs, stream bank; minimumElevationInMeters: 0; **Identification:** identifiedBy: K Arakaki; **Event:** verbatimEventDate: 03.vi.1998; eventRemarks: Pearl Harbor Survey; **Record Level:** institutionCode: BPBM
- **Type status:** Other material. **Occurrence:** catalogNumber: 2008000718; recordedBy: K Arakaki, GA Samuelson, K Kami; lifeStage: adult; **Taxon:** kingdom: Animalia; phylum: Arthropoda; class: Insecta; order: Diptera; family: Canacidae; genus: Procanace; specificEpithet: Procanacewilliamsi; scientificNameAuthorship: Wirth, 1951; **Location:** islandGroup: Hawaiian Islands; island: Oahu; verbatimLocality: Pearl Harbor, Waiawa Springs, stream bank; minimumElevationInMeters: 0; **Identification:** identifiedBy: K Arakaki; **Event:** verbatimEventDate: 03.vi.1998; eventRemarks: Pearl Harbor Survey; **Record Level:** institutionCode: BPBM
- **Type status:** Other material. **Occurrence:** catalogNumber: 2008000719; recordedBy: K Arakaki, GA Samuelson, K Kami; lifeStage: adult; **Taxon:** kingdom: Animalia; phylum: Arthropoda; class: Insecta; order: Diptera; family: Canacidae; genus: Procanace; specificEpithet: Procanacewilliamsi; scientificNameAuthorship: Wirth, 1951; **Location:** islandGroup: Hawaiian Islands; island: Oahu; verbatimLocality: Pearl Harbor, Waiawa Springs, stream bank; minimumElevationInMeters: 0; **Identification:** identifiedBy: K Arakaki; **Event:** verbatimEventDate: 03.vi.1998; eventRemarks: Pearl Harbor Survey; **Record Level:** institutionCode: BPBM
- **Type status:** Other material. **Occurrence:** catalogNumber: 2008000720; recordedBy: K Arakaki, GA Samuelson, K Kami; lifeStage: adult; **Taxon:** kingdom: Animalia; phylum: Arthropoda; class: Insecta; order: Diptera; family: Canacidae; genus: Procanace; specificEpithet: Procanacewilliamsi; scientificNameAuthorship: Wirth, 1951; **Location:** islandGroup: Hawaiian Islands; island: Oahu; verbatimLocality: Pearl Harbor, Waiawa Springs, stream bank; minimumElevationInMeters: 0; **Identification:** identifiedBy: K Arakaki; **Event:** verbatimEventDate: 03.vi.1998; eventRemarks: Pearl Harbor Survey; **Record Level:** institutionCode: BPBM
- **Type status:** Other material. **Occurrence:** catalogNumber: 2008000706; recordedBy: GA Samuelson, K Arakaki, K Kami; lifeStage: adult; **Taxon:** kingdom: Animalia; phylum: Arthropoda; class: Insecta; order: Diptera; family: Canacidae; genus: Procanace; specificEpithet: Procanacewilliamsi; scientificNameAuthorship: Wirth, 1951; **Location:** islandGroup: Hawaiian Islands; island: Oahu; verbatimLocality: Waikele Stream, near Cultural Garden; **Identification:** identifiedBy: K Arakaki; **Event:** verbatimEventDate: 09.vi.1998; eventRemarks: Pearl Harbor Survey; **Record Level:** institutionCode: BPBM
- **Type status:** Other material. **Occurrence:** catalogNumber: 2008000704; recordedBy: GA Samuelson, K Arakaki, K Kami; lifeStage: adult; **Taxon:** kingdom: Animalia; phylum: Arthropoda; class: Insecta; order: Diptera; family: Canacidae; genus: Procanace; specificEpithet: Procanacewilliamsi; scientificNameAuthorship: Wirth, 1951; **Location:** islandGroup: Hawaiian Islands; island: Oahu; verbatimLocality: Waikele Stream, near Cultural Garden; **Identification:** identifiedBy: K Arakaki; **Event:** verbatimEventDate: 09.vi.1998; eventRemarks: Pearl Harbor Survey; **Record Level:** institutionCode: BPBM
- **Type status:** Other material. **Occurrence:** catalogNumber: 2008000703; recordedBy: GA Samuelson, K Arakaki, K Kami; lifeStage: adult; **Taxon:** kingdom: Animalia; phylum: Arthropoda; class: Insecta; order: Diptera; family: Canacidae; genus: Procanace; specificEpithet: Procanacewilliamsi; scientificNameAuthorship: Wirth, 1951; **Location:** islandGroup: Hawaiian Islands; island: Oahu; verbatimLocality: Waikele Stream, near Cultural Garden; **Identification:** identifiedBy: K Arakaki; **Event:** verbatimEventDate: 09.vi.1998; eventRemarks: Pearl Harbor Survey; **Record Level:** institutionCode: BPBM
- **Type status:** Other material. **Occurrence:** catalogNumber: 2008000707; recordedBy: GA Samuelson, K Arakaki, K Kami; lifeStage: adult; **Taxon:** kingdom: Animalia; phylum: Arthropoda; class: Insecta; order: Diptera; family: Canacidae; genus: Procanace; specificEpithet: Procanacewilliamsi; scientificNameAuthorship: Wirth, 1951; **Location:** islandGroup: Hawaiian Islands; island: Oahu; verbatimLocality: Waikele Stream, near Cultural Garden; **Identification:** identifiedBy: K Arakaki; **Event:** verbatimEventDate: 09.vi.1998; eventRemarks: Pearl Harbor Survey; **Record Level:** institutionCode: BPBM
- **Type status:** Other material. **Occurrence:** catalogNumber: 2008000705; recordedBy: GA Samuelson, K Arakaki, K Kami; lifeStage: adult; **Taxon:** kingdom: Animalia; phylum: Arthropoda; class: Insecta; order: Diptera; family: Canacidae; genus: Procanace; specificEpithet: Procanacewilliamsi; scientificNameAuthorship: Wirth, 1951; **Location:** islandGroup: Hawaiian Islands; island: Oahu; verbatimLocality: Waikele Stream, near Cultural Garden; **Identification:** identifiedBy: K Arakaki; **Event:** verbatimEventDate: 09.vi.1998; eventRemarks: Pearl Harbor Survey; **Record Level:** institutionCode: BPBM
- **Type status:** Other material. **Occurrence:** catalogNumber: 2008000635; recordedBy: GA Samuelson, K Arakaki, K Kami; lifeStage: adult; **Taxon:** kingdom: Animalia; phylum: Arthropoda; class: Insecta; order: Diptera; family: Canacidae; genus: Procanace; specificEpithet: Procanacewilliamsi; scientificNameAuthorship: Wirth, 1951; **Location:** islandGroup: Hawaiian Islands; island: Oahu; verbatimLocality: Pearl Harbor, Honouliuli, shore; minimumElevationInMeters: 0; **Identification:** identifiedBy: K Arakaki; **Event:** verbatimEventDate: 17.vi.1998; eventRemarks: Pearl Harbor Survey; **Record Level:** institutionCode: BPBM
- **Type status:** Other material. **Occurrence:** catalogNumber: 2008000636; recordedBy: GA Samuelson, K Arakaki, K Kami; lifeStage: adult; **Taxon:** kingdom: Animalia; phylum: Arthropoda; class: Insecta; order: Diptera; family: Canacidae; genus: Procanace; specificEpithet: Procanacewilliamsi; scientificNameAuthorship: Wirth, 1951; **Location:** islandGroup: Hawaiian Islands; island: Oahu; verbatimLocality: Pearl Harbor, Honouliuli, shore; minimumElevationInMeters: 0; **Identification:** identifiedBy: K Arakaki; **Event:** verbatimEventDate: 17.vi.1998; eventRemarks: Pearl Harbor Survey; **Record Level:** institutionCode: BPBM
- **Type status:** Other material. **Occurrence:** catalogNumber: 2008000637; recordedBy: GA Samuelson, K Arakaki, K Kami; lifeStage: adult; **Taxon:** kingdom: Animalia; phylum: Arthropoda; class: Insecta; order: Diptera; family: Canacidae; genus: Procanace; specificEpithet: Procanacewilliamsi; scientificNameAuthorship: Wirth, 1951; **Location:** islandGroup: Hawaiian Islands; island: Oahu; verbatimLocality: Pearl Harbor, Honouliuli, shore; minimumElevationInMeters: 0; **Identification:** identifiedBy: K Arakaki; **Event:** verbatimEventDate: 17.vi.1998; eventRemarks: Pearl Harbor Survey; **Record Level:** institutionCode: BPBM
- **Type status:** Other material. **Occurrence:** catalogNumber: 2008000638; recordedBy: GA Samuelson, K Arakaki, K Kami; lifeStage: adult; **Taxon:** kingdom: Animalia; phylum: Arthropoda; class: Insecta; order: Diptera; family: Canacidae; genus: Procanace; specificEpithet: Procanacewilliamsi; scientificNameAuthorship: Wirth, 1951; **Location:** islandGroup: Hawaiian Islands; island: Oahu; verbatimLocality: Pearl Harbor, Honouliuli, shore; minimumElevationInMeters: 0; **Identification:** identifiedBy: K Arakaki; **Event:** verbatimEventDate: 17.vi.1998; eventRemarks: Pearl Harbor Survey; **Record Level:** institutionCode: BPBM
- **Type status:** Other material. **Occurrence:** catalogNumber: 2008000639; recordedBy: GA Samuelson, K Arakaki, K Kami; lifeStage: adult; **Taxon:** kingdom: Animalia; phylum: Arthropoda; class: Insecta; order: Diptera; family: Canacidae; genus: Procanace; specificEpithet: Procanacewilliamsi; scientificNameAuthorship: Wirth, 1951; **Location:** islandGroup: Hawaiian Islands; island: Oahu; verbatimLocality: Pearl Harbor, Honouliuli, shore; minimumElevationInMeters: 0; **Identification:** identifiedBy: K Arakaki; **Event:** verbatimEventDate: 17.vi.1998; eventRemarks: Pearl Harbor Survey; **Record Level:** institutionCode: BPBM
- **Type status:** Other material. **Occurrence:** catalogNumber: 2008000642; recordedBy: GA Samuelson, K Arakaki, K Kami; lifeStage: adult; **Taxon:** kingdom: Animalia; phylum: Arthropoda; class: Insecta; order: Diptera; family: Canacidae; genus: Procanace; specificEpithet: Procanacewilliamsi; scientificNameAuthorship: Wirth, 1951; **Location:** islandGroup: Hawaiian Islands; island: Oahu; verbatimLocality: Pearl Harbor, Honouliuli, shore; minimumElevationInMeters: 0; **Identification:** identifiedBy: K Arakaki; **Event:** verbatimEventDate: 17.vi.1998; eventRemarks: Pearl Harbor Survey; **Record Level:** institutionCode: BPBM
- **Type status:** Other material. **Occurrence:** catalogNumber: 2008000666; recordedBy: GA Samuelson, K Arakaki, K Kami; lifeStage: adult; **Taxon:** kingdom: Animalia; phylum: Arthropoda; class: Insecta; order: Diptera; family: Canacidae; genus: Procanace; specificEpithet: Procanacewilliamsi; scientificNameAuthorship: Wirth, 1951; **Location:** islandGroup: Hawaiian Islands; island: Oahu; verbatimLocality: Pearl Harbor, Honouliuli, shore; minimumElevationInMeters: 0; **Identification:** identifiedBy: K Arakaki; **Event:** verbatimEventDate: 17.vi.1998; eventRemarks: Pearl Harbor Survey; **Record Level:** institutionCode: BPBM
- **Type status:** Other material. **Occurrence:** catalogNumber: 2008000659; recordedBy: GA Samuelson, K Arakaki, K Kami; lifeStage: adult; **Taxon:** kingdom: Animalia; phylum: Arthropoda; class: Insecta; order: Diptera; family: Canacidae; genus: Procanace; specificEpithet: Procanacewilliamsi; scientificNameAuthorship: Wirth, 1951; **Location:** islandGroup: Hawaiian Islands; island: Oahu; verbatimLocality: Pearl Harbor, Honouliuli, shore; minimumElevationInMeters: 0; **Identification:** identifiedBy: K Arakaki; **Event:** verbatimEventDate: 17.vi.1998; eventRemarks: Pearl Harbor Survey; **Record Level:** institutionCode: BPBM
- **Type status:** Other material. **Occurrence:** catalogNumber: 2008000660; recordedBy: GA Samuelson, K Arakaki, K Kami; lifeStage: adult; **Taxon:** kingdom: Animalia; phylum: Arthropoda; class: Insecta; order: Diptera; family: Canacidae; genus: Procanace; specificEpithet: Procanacewilliamsi; scientificNameAuthorship: Wirth, 1951; **Location:** islandGroup: Hawaiian Islands; island: Oahu; verbatimLocality: Pearl Harbor, Honouliuli, shore; minimumElevationInMeters: 0; **Identification:** identifiedBy: K Arakaki; **Event:** verbatimEventDate: 17.vi.1998; eventRemarks: Pearl Harbor Survey; **Record Level:** institutionCode: BPBM
- **Type status:** Other material. **Occurrence:** catalogNumber: 2008000661; recordedBy: GA Samuelson, K Arakaki, K Kami; lifeStage: adult; **Taxon:** kingdom: Animalia; phylum: Arthropoda; class: Insecta; order: Diptera; family: Canacidae; genus: Procanace; specificEpithet: Procanacewilliamsi; scientificNameAuthorship: Wirth, 1951; **Location:** islandGroup: Hawaiian Islands; island: Oahu; verbatimLocality: Pearl Harbor, Honouliuli, shore; minimumElevationInMeters: 0; **Identification:** identifiedBy: K Arakaki; **Event:** verbatimEventDate: 17.vi.1998; eventRemarks: Pearl Harbor Survey; **Record Level:** institutionCode: BPBM
- **Type status:** Other material. **Occurrence:** catalogNumber: 2008000662; recordedBy: GA Samuelson, K Arakaki, K Kami; lifeStage: adult; **Taxon:** kingdom: Animalia; phylum: Arthropoda; class: Insecta; order: Diptera; family: Canacidae; genus: Procanace; specificEpithet: Procanacewilliamsi; scientificNameAuthorship: Wirth, 1951; **Location:** islandGroup: Hawaiian Islands; island: Oahu; verbatimLocality: Pearl Harbor, Honouliuli, shore; minimumElevationInMeters: 0; **Identification:** identifiedBy: K Arakaki; **Event:** verbatimEventDate: 17.vi.1998; eventRemarks: Pearl Harbor Survey; **Record Level:** institutionCode: BPBM
- **Type status:** Other material. **Occurrence:** catalogNumber: 2008000663; recordedBy: GA Samuelson, K Arakaki, K Kami; lifeStage: adult; **Taxon:** kingdom: Animalia; phylum: Arthropoda; class: Insecta; order: Diptera; family: Canacidae; genus: Procanace; specificEpithet: Procanacewilliamsi; scientificNameAuthorship: Wirth, 1951; **Location:** islandGroup: Hawaiian Islands; island: Oahu; verbatimLocality: Pearl Harbor, Honouliuli, shore; minimumElevationInMeters: 0; **Identification:** identifiedBy: K Arakaki; **Event:** verbatimEventDate: 17.vi.1998; eventRemarks: Pearl Harbor Survey; **Record Level:** institutionCode: BPBM
- **Type status:** Other material. **Occurrence:** catalogNumber: 2008000640; recordedBy: GA Samuelson, K Arakaki, K Kami; lifeStage: adult; **Taxon:** kingdom: Animalia; phylum: Arthropoda; class: Insecta; order: Diptera; family: Canacidae; genus: Procanace; specificEpithet: Procanacewilliamsi; scientificNameAuthorship: Wirth, 1951; **Location:** islandGroup: Hawaiian Islands; island: Oahu; verbatimLocality: Pearl Harbor, Honouliuli, shore; minimumElevationInMeters: 0; **Identification:** identifiedBy: K Arakaki; **Event:** verbatimEventDate: 17.vi.1998; eventRemarks: Pearl Harbor Survey; **Record Level:** institutionCode: BPBM
- **Type status:** Other material. **Occurrence:** catalogNumber: 2008000656; recordedBy: GA Samuelson, K Arakaki, K Kami; lifeStage: adult; **Taxon:** kingdom: Animalia; phylum: Arthropoda; class: Insecta; order: Diptera; family: Canacidae; genus: Procanace; specificEpithet: Procanacewilliamsi; scientificNameAuthorship: Wirth, 1951; **Location:** islandGroup: Hawaiian Islands; island: Oahu; verbatimLocality: Pearl Harbor, Honouliuli, shore; minimumElevationInMeters: 0; **Identification:** identifiedBy: K Arakaki; **Event:** verbatimEventDate: 17.vi.1998; eventRemarks: Pearl Harbor Survey; **Record Level:** institutionCode: BPBM
- **Type status:** Other material. **Occurrence:** catalogNumber: 2008000665; recordedBy: GA Samuelson, K Arakaki, K Kami; lifeStage: adult; **Taxon:** kingdom: Animalia; phylum: Arthropoda; class: Insecta; order: Diptera; family: Canacidae; genus: Procanace; specificEpithet: Procanacewilliamsi; scientificNameAuthorship: Wirth, 1951; **Location:** islandGroup: Hawaiian Islands; island: Oahu; verbatimLocality: Pearl Harbor, Honouliuli, shore; minimumElevationInMeters: 0; **Identification:** identifiedBy: K Arakaki; **Event:** verbatimEventDate: 17.vi.1998; eventRemarks: Pearl Harbor Survey; **Record Level:** institutionCode: BPBM
- **Type status:** Other material. **Occurrence:** catalogNumber: 2008000667; recordedBy: GA Samuelson, K Arakaki, K Kami; lifeStage: adult; **Taxon:** kingdom: Animalia; phylum: Arthropoda; class: Insecta; order: Diptera; family: Canacidae; genus: Procanace; specificEpithet: Procanacewilliamsi; scientificNameAuthorship: Wirth, 1951; **Location:** islandGroup: Hawaiian Islands; island: Oahu; verbatimLocality: Pearl Harbor, Honouliuli, shore; minimumElevationInMeters: 0; **Identification:** identifiedBy: K Arakaki; **Event:** verbatimEventDate: 17.vi.1998; eventRemarks: Pearl Harbor Survey; **Record Level:** institutionCode: BPBM
- **Type status:** Other material. **Occurrence:** catalogNumber: 2008000668; recordedBy: GA Samuelson, K Arakaki, K Kami; lifeStage: adult; **Taxon:** kingdom: Animalia; phylum: Arthropoda; class: Insecta; order: Diptera; family: Canacidae; genus: Procanace; specificEpithet: Procanacewilliamsi; scientificNameAuthorship: Wirth, 1951; **Location:** islandGroup: Hawaiian Islands; island: Oahu; verbatimLocality: Pearl Harbor, Honouliuli, shore; minimumElevationInMeters: 0; **Identification:** identifiedBy: K Arakaki; **Event:** verbatimEventDate: 17.vi.1998; eventRemarks: Pearl Harbor Survey; **Record Level:** institutionCode: BPBM
- **Type status:** Other material. **Occurrence:** catalogNumber: 2008000669; recordedBy: GA Samuelson, K Arakaki, K Kami; lifeStage: adult; **Taxon:** kingdom: Animalia; phylum: Arthropoda; class: Insecta; order: Diptera; family: Canacidae; genus: Procanace; specificEpithet: Procanacewilliamsi; scientificNameAuthorship: Wirth, 1951; **Location:** islandGroup: Hawaiian Islands; island: Oahu; verbatimLocality: Pearl Harbor, Honouliuli, shore; minimumElevationInMeters: 0; **Identification:** identifiedBy: K Arakaki; **Event:** verbatimEventDate: 17.vi.1998; eventRemarks: Pearl Harbor Survey; **Record Level:** institutionCode: BPBM
- **Type status:** Other material. **Occurrence:** catalogNumber: 2008000670; recordedBy: GA Samuelson, K Arakaki, K Kami; lifeStage: adult; **Taxon:** kingdom: Animalia; phylum: Arthropoda; class: Insecta; order: Diptera; family: Canacidae; genus: Procanace; specificEpithet: Procanacewilliamsi; scientificNameAuthorship: Wirth, 1951; **Location:** islandGroup: Hawaiian Islands; island: Oahu; verbatimLocality: Pearl Harbor, Honouliuli, shore; minimumElevationInMeters: 0; **Identification:** identifiedBy: K Arakaki; **Event:** verbatimEventDate: 17.vi.1998; eventRemarks: Pearl Harbor Survey; **Record Level:** institutionCode: BPBM
- **Type status:** Other material. **Occurrence:** catalogNumber: 2008000671; recordedBy: GA Samuelson, K Arakaki, K Kami; lifeStage: adult; **Taxon:** kingdom: Animalia; phylum: Arthropoda; class: Insecta; order: Diptera; family: Canacidae; genus: Procanace; specificEpithet: Procanacewilliamsi; scientificNameAuthorship: Wirth, 1951; **Location:** islandGroup: Hawaiian Islands; island: Oahu; verbatimLocality: Pearl Harbor, Honouliuli, shore; minimumElevationInMeters: 0; **Identification:** identifiedBy: K Arakaki; **Event:** verbatimEventDate: 17.vi.1998; eventRemarks: Pearl Harbor Survey; **Record Level:** institutionCode: BPBM
- **Type status:** Other material. **Occurrence:** catalogNumber: 2008000672; recordedBy: GA Samuelson, K Arakaki, K Kami; lifeStage: adult; **Taxon:** kingdom: Animalia; phylum: Arthropoda; class: Insecta; order: Diptera; family: Canacidae; genus: Procanace; specificEpithet: Procanacewilliamsi; scientificNameAuthorship: Wirth, 1951; **Location:** islandGroup: Hawaiian Islands; island: Oahu; verbatimLocality: Pearl Harbor, Honouliuli, shore; minimumElevationInMeters: 0; **Identification:** identifiedBy: K Arakaki; **Event:** verbatimEventDate: 17.vi.1998; eventRemarks: Pearl Harbor Survey; **Record Level:** institutionCode: BPBM
- **Type status:** Other material. **Occurrence:** catalogNumber: 2008000664; recordedBy: GA Samuelson, K Arakaki, K Kami; lifeStage: adult; **Taxon:** kingdom: Animalia; phylum: Arthropoda; class: Insecta; order: Diptera; family: Canacidae; genus: Procanace; specificEpithet: Procanacewilliamsi; scientificNameAuthorship: Wirth, 1951; **Location:** islandGroup: Hawaiian Islands; island: Oahu; verbatimLocality: Pearl Harbor, Honouliuli, shore; minimumElevationInMeters: 0; **Identification:** identifiedBy: K Arakaki; **Event:** verbatimEventDate: 17.vi.1998; eventRemarks: Pearl Harbor Survey; **Record Level:** institutionCode: BPBM
- **Type status:** Other material. **Occurrence:** catalogNumber: 2008000650; recordedBy: GA Samuelson, K Arakaki, K Kami; lifeStage: adult; **Taxon:** kingdom: Animalia; phylum: Arthropoda; class: Insecta; order: Diptera; family: Canacidae; genus: Procanace; specificEpithet: Procanacewilliamsi; scientificNameAuthorship: Wirth, 1951; **Location:** islandGroup: Hawaiian Islands; island: Oahu; verbatimLocality: Pearl Harbor, Honouliuli, shore; minimumElevationInMeters: 0; **Identification:** identifiedBy: K Arakaki; **Event:** verbatimEventDate: 17.vi.1998; eventRemarks: Pearl Harbor Survey; **Record Level:** institutionCode: BPBM
- **Type status:** Other material. **Occurrence:** catalogNumber: 2008000643; recordedBy: GA Samuelson, K Arakaki, K Kami; lifeStage: adult; **Taxon:** kingdom: Animalia; phylum: Arthropoda; class: Insecta; order: Diptera; family: Canacidae; genus: Procanace; specificEpithet: Procanacewilliamsi; scientificNameAuthorship: Wirth, 1951; **Location:** islandGroup: Hawaiian Islands; island: Oahu; verbatimLocality: Pearl Harbor, Honouliuli, shore; minimumElevationInMeters: 0; **Identification:** identifiedBy: K Arakaki; **Event:** verbatimEventDate: 17.vi.1998; eventRemarks: Pearl Harbor Survey; **Record Level:** institutionCode: BPBM
- **Type status:** Other material. **Occurrence:** catalogNumber: 2008000644; recordedBy: GA Samuelson, K Arakaki, K Kami; lifeStage: adult; **Taxon:** kingdom: Animalia; phylum: Arthropoda; class: Insecta; order: Diptera; family: Canacidae; genus: Procanace; specificEpithet: Procanacewilliamsi; scientificNameAuthorship: Wirth, 1951; **Location:** islandGroup: Hawaiian Islands; island: Oahu; verbatimLocality: Pearl Harbor, Honouliuli, shore; minimumElevationInMeters: 0; **Identification:** identifiedBy: K Arakaki; **Event:** verbatimEventDate: 17.vi.1998; eventRemarks: Pearl Harbor Survey; **Record Level:** institutionCode: BPBM
- **Type status:** Other material. **Occurrence:** catalogNumber: 2008000645; recordedBy: GA Samuelson, K Arakaki, K Kami; lifeStage: adult; **Taxon:** kingdom: Animalia; phylum: Arthropoda; class: Insecta; order: Diptera; family: Canacidae; genus: Procanace; specificEpithet: Procanacewilliamsi; scientificNameAuthorship: Wirth, 1951; **Location:** islandGroup: Hawaiian Islands; island: Oahu; verbatimLocality: Pearl Harbor, Honouliuli, shore; minimumElevationInMeters: 0; **Identification:** identifiedBy: K Arakaki; **Event:** verbatimEventDate: 17.vi.1998; eventRemarks: Pearl Harbor Survey; **Record Level:** institutionCode: BPBM
- **Type status:** Other material. **Occurrence:** catalogNumber: 2008000646; recordedBy: GA Samuelson, K Arakaki, K Kami; lifeStage: adult; **Taxon:** kingdom: Animalia; phylum: Arthropoda; class: Insecta; order: Diptera; family: Canacidae; genus: Procanace; specificEpithet: Procanacewilliamsi; scientificNameAuthorship: Wirth, 1951; **Location:** islandGroup: Hawaiian Islands; island: Oahu; verbatimLocality: Pearl Harbor, Honouliuli, shore; minimumElevationInMeters: 0; **Identification:** identifiedBy: K Arakaki; **Event:** verbatimEventDate: 17.vi.1998; eventRemarks: Pearl Harbor Survey; **Record Level:** institutionCode: BPBM
- **Type status:** Other material. **Occurrence:** catalogNumber: 2008000647; recordedBy: GA Samuelson, K Arakaki, K Kami; lifeStage: adult; **Taxon:** kingdom: Animalia; phylum: Arthropoda; class: Insecta; order: Diptera; family: Canacidae; genus: Procanace; specificEpithet: Procanacewilliamsi; scientificNameAuthorship: Wirth, 1951; **Location:** islandGroup: Hawaiian Islands; island: Oahu; verbatimLocality: Pearl Harbor, Honouliuli, shore; minimumElevationInMeters: 0; **Identification:** identifiedBy: K Arakaki; **Event:** verbatimEventDate: 17.vi.1998; eventRemarks: Pearl Harbor Survey; **Record Level:** institutionCode: BPBM
- **Type status:** Other material. **Occurrence:** catalogNumber: 2008000658; recordedBy: GA Samuelson, K Arakaki, K Kami; lifeStage: adult; **Taxon:** kingdom: Animalia; phylum: Arthropoda; class: Insecta; order: Diptera; family: Canacidae; genus: Procanace; specificEpithet: Procanacewilliamsi; scientificNameAuthorship: Wirth, 1951; **Location:** islandGroup: Hawaiian Islands; island: Oahu; verbatimLocality: Pearl Harbor, Honouliuli, shore; minimumElevationInMeters: 0; **Identification:** identifiedBy: K Arakaki; **Event:** verbatimEventDate: 17.vi.1998; eventRemarks: Pearl Harbor Survey; **Record Level:** institutionCode: BPBM
- **Type status:** Other material. **Occurrence:** catalogNumber: 2008000649; recordedBy: GA Samuelson, K Arakaki, K Kami; lifeStage: adult; **Taxon:** kingdom: Animalia; phylum: Arthropoda; class: Insecta; order: Diptera; family: Canacidae; genus: Procanace; specificEpithet: Procanacewilliamsi; scientificNameAuthorship: Wirth, 1951; **Location:** islandGroup: Hawaiian Islands; island: Oahu; verbatimLocality: Pearl Harbor, Honouliuli, shore; minimumElevationInMeters: 0; **Identification:** identifiedBy: K Arakaki; **Event:** verbatimEventDate: 17.vi.1998; eventRemarks: Pearl Harbor Survey; **Record Level:** institutionCode: BPBM
- **Type status:** Other material. **Occurrence:** catalogNumber: 2008000657; recordedBy: GA Samuelson, K Arakaki, K Kami; lifeStage: adult; **Taxon:** kingdom: Animalia; phylum: Arthropoda; class: Insecta; order: Diptera; family: Canacidae; genus: Procanace; specificEpithet: Procanacewilliamsi; scientificNameAuthorship: Wirth, 1951; **Location:** islandGroup: Hawaiian Islands; island: Oahu; verbatimLocality: Pearl Harbor, Honouliuli, shore; minimumElevationInMeters: 0; **Identification:** identifiedBy: K Arakaki; **Event:** verbatimEventDate: 17.vi.1998; eventRemarks: Pearl Harbor Survey; **Record Level:** institutionCode: BPBM
- **Type status:** Other material. **Occurrence:** catalogNumber: 2008000651; recordedBy: GA Samuelson, K Arakaki, K Kami; lifeStage: adult; **Taxon:** kingdom: Animalia; phylum: Arthropoda; class: Insecta; order: Diptera; family: Canacidae; genus: Procanace; specificEpithet: Procanacewilliamsi; scientificNameAuthorship: Wirth, 1951; **Location:** islandGroup: Hawaiian Islands; island: Oahu; verbatimLocality: Pearl Harbor, Honouliuli, shore; minimumElevationInMeters: 0; **Identification:** identifiedBy: K Arakaki; **Event:** verbatimEventDate: 17.vi.1998; eventRemarks: Pearl Harbor Survey; **Record Level:** institutionCode: BPBM
- **Type status:** Other material. **Occurrence:** catalogNumber: 2008000652; recordedBy: GA Samuelson, K Arakaki, K Kami; lifeStage: adult; **Taxon:** kingdom: Animalia; phylum: Arthropoda; class: Insecta; order: Diptera; family: Canacidae; genus: Procanace; specificEpithet: Procanacewilliamsi; scientificNameAuthorship: Wirth, 1951; **Location:** islandGroup: Hawaiian Islands; island: Oahu; verbatimLocality: Pearl Harbor, Honouliuli, shore; minimumElevationInMeters: 0; **Identification:** identifiedBy: K Arakaki; **Event:** verbatimEventDate: 17.vi.1998; eventRemarks: Pearl Harbor Survey; **Record Level:** institutionCode: BPBM
- **Type status:** Other material. **Occurrence:** catalogNumber: 2008000653; recordedBy: GA Samuelson, K Arakaki, K Kami; lifeStage: adult; **Taxon:** kingdom: Animalia; phylum: Arthropoda; class: Insecta; order: Diptera; family: Canacidae; genus: Procanace; specificEpithet: Procanacewilliamsi; scientificNameAuthorship: Wirth, 1951; **Location:** islandGroup: Hawaiian Islands; island: Oahu; verbatimLocality: Pearl Harbor, Honouliuli, shore; minimumElevationInMeters: 0; **Identification:** identifiedBy: K Arakaki; **Event:** verbatimEventDate: 17.vi.1998; eventRemarks: Pearl Harbor Survey; **Record Level:** institutionCode: BPBM
- **Type status:** Other material. **Occurrence:** catalogNumber: 2008000654; recordedBy: GA Samuelson, K Arakaki, K Kami; lifeStage: adult; **Taxon:** kingdom: Animalia; phylum: Arthropoda; class: Insecta; order: Diptera; family: Canacidae; genus: Procanace; specificEpithet: Procanacewilliamsi; scientificNameAuthorship: Wirth, 1951; **Location:** islandGroup: Hawaiian Islands; island: Oahu; verbatimLocality: Pearl Harbor, Honouliuli, shore; minimumElevationInMeters: 0; **Identification:** identifiedBy: K Arakaki; **Event:** verbatimEventDate: 17.vi.1998; eventRemarks: Pearl Harbor Survey; **Record Level:** institutionCode: BPBM
- **Type status:** Other material. **Occurrence:** catalogNumber: 2008000655; recordedBy: GA Samuelson, K Arakaki, K Kami; lifeStage: adult; **Taxon:** kingdom: Animalia; phylum: Arthropoda; class: Insecta; order: Diptera; family: Canacidae; genus: Procanace; specificEpithet: Procanacewilliamsi; scientificNameAuthorship: Wirth, 1951; **Location:** islandGroup: Hawaiian Islands; island: Oahu; verbatimLocality: Pearl Harbor, Honouliuli, shore; minimumElevationInMeters: 0; **Identification:** identifiedBy: K Arakaki; **Event:** verbatimEventDate: 17.vi.1998; eventRemarks: Pearl Harbor Survey; **Record Level:** institutionCode: BPBM
- **Type status:** Other material. **Occurrence:** catalogNumber: 2008000641; recordedBy: GA Samuelson, K Arakaki, K Kami; lifeStage: adult; **Taxon:** kingdom: Animalia; phylum: Arthropoda; class: Insecta; order: Diptera; family: Canacidae; genus: Procanace; specificEpithet: Procanacewilliamsi; scientificNameAuthorship: Wirth, 1951; **Location:** islandGroup: Hawaiian Islands; island: Oahu; verbatimLocality: Pearl Harbor, Honouliuli, shore; minimumElevationInMeters: 0; **Identification:** identifiedBy: K Arakaki; **Event:** verbatimEventDate: 17.vi.1998; eventRemarks: Pearl Harbor Survey; **Record Level:** institutionCode: BPBM
- **Type status:** Other material. **Occurrence:** catalogNumber: 2008000648; recordedBy: GA Samuelson, K Arakaki, K Kami; lifeStage: adult; **Taxon:** kingdom: Animalia; phylum: Arthropoda; class: Insecta; order: Diptera; family: Canacidae; genus: Procanace; specificEpithet: Procanacewilliamsi; scientificNameAuthorship: Wirth, 1951; **Location:** islandGroup: Hawaiian Islands; island: Oahu; verbatimLocality: Pearl Harbor, Honouliuli, shore; minimumElevationInMeters: 0; **Identification:** identifiedBy: K Arakaki; **Event:** verbatimEventDate: 17.vi.1998; eventRemarks: Pearl Harbor Survey; **Record Level:** institutionCode: BPBM
- **Type status:** Other material. **Occurrence:** catalogNumber: 2008000675; recordedBy: GA Samuelson, K Arakaki, K Kami; lifeStage: adult; **Taxon:** kingdom: Animalia; phylum: Arthropoda; class: Insecta; order: Diptera; family: Canacidae; genus: Procanace; specificEpithet: Procanacewilliamsi; scientificNameAuthorship: Wirth, 1951; **Location:** islandGroup: Hawaiian Islands; island: Oahu; verbatimLocality: Pearl Harbor, Honouliuli, shore; minimumElevationInMeters: 0; **Identification:** identifiedBy: K Arakaki; **Event:** verbatimEventDate: 17.vi.1998; eventRemarks: Pearl Harbor Survey; **Record Level:** institutionCode: BPBM
- **Type status:** Other material. **Occurrence:** catalogNumber: 2008000673; recordedBy: GA Samuelson, K Arakaki, K Kami; lifeStage: adult; **Taxon:** kingdom: Animalia; phylum: Arthropoda; class: Insecta; order: Diptera; family: Canacidae; genus: Procanace; specificEpithet: Procanacewilliamsi; scientificNameAuthorship: Wirth, 1951; **Location:** islandGroup: Hawaiian Islands; island: Oahu; verbatimLocality: Pearl Harbor, Honouliuli, shore; minimumElevationInMeters: 0; **Identification:** identifiedBy: K Arakaki; **Event:** verbatimEventDate: 17.vi.1998; eventRemarks: Pearl Harbor Survey; **Record Level:** institutionCode: BPBM
- **Type status:** Other material. **Occurrence:** catalogNumber: 2008000692; recordedBy: K Arakaki, GA Samuelson, K Kami; lifeStage: adult; **Taxon:** kingdom: Animalia; phylum: Arthropoda; class: Insecta; order: Diptera; family: Canacidae; genus: Procanace; specificEpithet: Procanacewilliamsi; scientificNameAuthorship: Wirth, 1951; **Location:** islandGroup: Hawaiian Islands; island: Oahu; verbatimLocality: Pearl Harbor, Honouliuli, rocky mud flats; **Identification:** identifiedBy: K Arakaki; **Event:** verbatimEventDate: 17.vi.1998; eventRemarks: Pearl Harbor Survey; **Record Level:** institutionCode: BPBM
- **Type status:** Other material. **Occurrence:** catalogNumber: 2008000676; recordedBy: GA Samuelson, K Arakaki, K Kami; lifeStage: adult; **Taxon:** kingdom: Animalia; phylum: Arthropoda; class: Insecta; order: Diptera; family: Canacidae; genus: Procanace; specificEpithet: Procanacewilliamsi; scientificNameAuthorship: Wirth, 1951; **Location:** islandGroup: Hawaiian Islands; island: Oahu; verbatimLocality: Pearl Harbor, Honouliuli, shore; minimumElevationInMeters: 0; **Identification:** identifiedBy: K Arakaki; **Event:** verbatimEventDate: 17.vi.1998; eventRemarks: Pearl Harbor Survey; **Record Level:** institutionCode: BPBM
- **Type status:** Other material. **Occurrence:** catalogNumber: 2008000683; recordedBy: GA Samuelson, K Arakaki, K Kami; lifeStage: adult; **Taxon:** kingdom: Animalia; phylum: Arthropoda; class: Insecta; order: Diptera; family: Canacidae; genus: Procanace; specificEpithet: Procanacewilliamsi; scientificNameAuthorship: Wirth, 1951; **Location:** islandGroup: Hawaiian Islands; island: Oahu; verbatimLocality: Pearl Harbor, Honouliuli, shore; minimumElevationInMeters: 0; **Identification:** identifiedBy: K Arakaki; **Event:** verbatimEventDate: 17.vi.1998; eventRemarks: Pearl Harbor Survey; **Record Level:** institutionCode: BPBM
- **Type status:** Other material. **Occurrence:** catalogNumber: 2008000689; recordedBy: K Arakaki, GA Samuelson, K Kami; lifeStage: adult; **Taxon:** kingdom: Animalia; phylum: Arthropoda; class: Insecta; order: Diptera; family: Canacidae; genus: Procanace; specificEpithet: Procanacewilliamsi; scientificNameAuthorship: Wirth, 1951; **Location:** islandGroup: Hawaiian Islands; island: Oahu; verbatimLocality: Pearl Harbor, Honouliuli, rocky mud flats; **Identification:** identifiedBy: K Arakaki; **Event:** verbatimEventDate: 17.vi.1998; eventRemarks: Pearl Harbor Survey; **Record Level:** institutionCode: BPBM
- **Type status:** Other material. **Occurrence:** catalogNumber: 2008000677; recordedBy: GA Samuelson, K Arakaki, K Kami; lifeStage: adult; **Taxon:** kingdom: Animalia; phylum: Arthropoda; class: Insecta; order: Diptera; family: Canacidae; genus: Procanace; specificEpithet: Procanacewilliamsi; scientificNameAuthorship: Wirth, 1951; **Location:** islandGroup: Hawaiian Islands; island: Oahu; verbatimLocality: Pearl Harbor, Honouliuli, shore; minimumElevationInMeters: 0; **Identification:** identifiedBy: K Arakaki; **Event:** verbatimEventDate: 17.vi.1998; eventRemarks: Pearl Harbor Survey; **Record Level:** institutionCode: BPBM
- **Type status:** Other material. **Occurrence:** catalogNumber: 2008000679; recordedBy: GA Samuelson, K Arakaki, K Kami; lifeStage: adult; **Taxon:** kingdom: Animalia; phylum: Arthropoda; class: Insecta; order: Diptera; family: Canacidae; genus: Procanace; specificEpithet: Procanacewilliamsi; scientificNameAuthorship: Wirth, 1951; **Location:** islandGroup: Hawaiian Islands; island: Oahu; verbatimLocality: Pearl Harbor, Honouliuli, shore; minimumElevationInMeters: 0; **Identification:** identifiedBy: K Arakaki; **Event:** verbatimEventDate: 17.vi.1998; eventRemarks: Pearl Harbor Survey; **Record Level:** institutionCode: BPBM
- **Type status:** Other material. **Occurrence:** catalogNumber: 2008000678; recordedBy: GA Samuelson, K Arakaki, K Kami; lifeStage: adult; **Taxon:** kingdom: Animalia; phylum: Arthropoda; class: Insecta; order: Diptera; family: Canacidae; genus: Procanace; specificEpithet: Procanacewilliamsi; scientificNameAuthorship: Wirth, 1951; **Location:** islandGroup: Hawaiian Islands; island: Oahu; verbatimLocality: Pearl Harbor, Honouliuli, shore; minimumElevationInMeters: 0; **Identification:** identifiedBy: K Arakaki; **Event:** verbatimEventDate: 17.vi.1998; eventRemarks: Pearl Harbor Survey; **Record Level:** institutionCode: BPBM
- **Type status:** Other material. **Occurrence:** catalogNumber: 2008000680; recordedBy: GA Samuelson, K Arakaki, K Kami; lifeStage: adult; **Taxon:** kingdom: Animalia; phylum: Arthropoda; class: Insecta; order: Diptera; family: Canacidae; genus: Procanace; specificEpithet: Procanacewilliamsi; scientificNameAuthorship: Wirth, 1951; **Location:** islandGroup: Hawaiian Islands; island: Oahu; verbatimLocality: Pearl Harbor, Honouliuli, shore; minimumElevationInMeters: 0; **Identification:** identifiedBy: K Arakaki; **Event:** verbatimEventDate: 17.vi.1998; eventRemarks: Pearl Harbor Survey; **Record Level:** institutionCode: BPBM
- **Type status:** Other material. **Occurrence:** catalogNumber: 2008000691; recordedBy: K Arakaki, GA Samuelson, K Kami; lifeStage: adult; **Taxon:** kingdom: Animalia; phylum: Arthropoda; class: Insecta; order: Diptera; family: Canacidae; genus: Procanace; specificEpithet: Procanacewilliamsi; scientificNameAuthorship: Wirth, 1951; **Location:** islandGroup: Hawaiian Islands; island: Oahu; verbatimLocality: Pearl Harbor, Honouliuli, rocky mud flats; **Identification:** identifiedBy: K Arakaki; **Event:** verbatimEventDate: 17.vi.1998; eventRemarks: Pearl Harbor Survey; **Record Level:** institutionCode: BPBM
- **Type status:** Other material. **Occurrence:** catalogNumber: 2008000682; recordedBy: GA Samuelson, K Arakaki, K Kami; lifeStage: adult; **Taxon:** kingdom: Animalia; phylum: Arthropoda; class: Insecta; order: Diptera; family: Canacidae; genus: Procanace; specificEpithet: Procanacewilliamsi; scientificNameAuthorship: Wirth, 1951; **Location:** islandGroup: Hawaiian Islands; island: Oahu; verbatimLocality: Pearl Harbor, Honouliuli, shore; minimumElevationInMeters: 0; **Identification:** identifiedBy: K Arakaki; **Event:** verbatimEventDate: 17.vi.1998; eventRemarks: Pearl Harbor Survey; **Record Level:** institutionCode: BPBM
- **Type status:** Other material. **Occurrence:** catalogNumber: 2008000684; recordedBy: GA Samuelson, K Arakaki, K Kami; lifeStage: adult; **Taxon:** kingdom: Animalia; phylum: Arthropoda; class: Insecta; order: Diptera; family: Canacidae; genus: Procanace; specificEpithet: Procanacewilliamsi; scientificNameAuthorship: Wirth, 1951; **Location:** islandGroup: Hawaiian Islands; island: Oahu; verbatimLocality: Pearl Harbor, Honouliuli, shore; minimumElevationInMeters: 0; **Identification:** identifiedBy: K Arakaki; **Event:** verbatimEventDate: 17.vi.1998; eventRemarks: Pearl Harbor Survey; **Record Level:** institutionCode: BPBM
- **Type status:** Other material. **Occurrence:** catalogNumber: 2008000685; recordedBy: K Arakaki, GA Samuelson, K Kami; lifeStage: adult; **Taxon:** kingdom: Animalia; phylum: Arthropoda; class: Insecta; order: Diptera; family: Canacidae; genus: Procanace; specificEpithet: Procanacewilliamsi; scientificNameAuthorship: Wirth, 1951; **Location:** islandGroup: Hawaiian Islands; island: Oahu; verbatimLocality: Pearl Harbor, Honouliuli, pong along golf course, opposite pier; minimumElevationInMeters: 0; **Identification:** identifiedBy: K Arakaki; **Event:** verbatimEventDate: 17.vi.1998; eventRemarks: Pearl Harbor Survey; **Record Level:** institutionCode: BPBM
- **Type status:** Other material. **Occurrence:** catalogNumber: 2008000690; recordedBy: K Arakaki, GA Samuelson, K Kami; lifeStage: adult; **Taxon:** kingdom: Animalia; phylum: Arthropoda; class: Insecta; order: Diptera; family: Canacidae; genus: Procanace; specificEpithet: Procanacewilliamsi; scientificNameAuthorship: Wirth, 1951; **Location:** islandGroup: Hawaiian Islands; island: Oahu; verbatimLocality: Pearl Harbor, Honouliuli, rocky mud flats; **Identification:** identifiedBy: K Arakaki; **Event:** verbatimEventDate: 17.vi.1998; eventRemarks: Pearl Harbor Survey; **Record Level:** institutionCode: BPBM
- **Type status:** Other material. **Occurrence:** catalogNumber: 2008000686; recordedBy: K Arakaki, GA Samuelson, K Kami; lifeStage: adult; **Taxon:** kingdom: Animalia; phylum: Arthropoda; class: Insecta; order: Diptera; family: Canacidae; genus: Procanace; specificEpithet: Procanacewilliamsi; scientificNameAuthorship: Wirth, 1951; **Location:** islandGroup: Hawaiian Islands; island: Oahu; verbatimLocality: Pearl Harbor, Honouliuli, rocky mud flats; **Identification:** identifiedBy: K Arakaki; **Event:** verbatimEventDate: 17.vi.1998; eventRemarks: Pearl Harbor Survey; **Record Level:** institutionCode: BPBM
- **Type status:** Other material. **Occurrence:** catalogNumber: 2008000688; recordedBy: K Arakaki, GA Samuelson, K Kami; lifeStage: adult; **Taxon:** kingdom: Animalia; phylum: Arthropoda; class: Insecta; order: Diptera; family: Canacidae; genus: Procanace; specificEpithet: Procanacewilliamsi; scientificNameAuthorship: Wirth, 1951; **Location:** islandGroup: Hawaiian Islands; island: Oahu; verbatimLocality: Pearl Harbor, Honouliuli, rocky mud flats; **Identification:** identifiedBy: K Arakaki; **Event:** verbatimEventDate: 17.vi.1998; eventRemarks: Pearl Harbor Survey; **Record Level:** institutionCode: BPBM
- **Type status:** Other material. **Occurrence:** catalogNumber: 2008000681; recordedBy: GA Samuelson, K Arakaki, K Kami; lifeStage: adult; **Taxon:** kingdom: Animalia; phylum: Arthropoda; class: Insecta; order: Diptera; family: Canacidae; genus: Procanace; specificEpithet: Procanacewilliamsi; scientificNameAuthorship: Wirth, 1951; **Location:** islandGroup: Hawaiian Islands; island: Oahu; verbatimLocality: Pearl Harbor, Honouliuli, shore; minimumElevationInMeters: 0; **Identification:** identifiedBy: K Arakaki; **Event:** verbatimEventDate: 17.vi.1998; eventRemarks: Pearl Harbor Survey; **Record Level:** institutionCode: BPBM
- **Type status:** Other material. **Occurrence:** catalogNumber: 2008000687; recordedBy: K Arakaki, GA Samuelson, K Kami; lifeStage: adult; **Taxon:** kingdom: Animalia; phylum: Arthropoda; class: Insecta; order: Diptera; family: Canacidae; genus: Procanace; specificEpithet: Procanacewilliamsi; scientificNameAuthorship: Wirth, 1951; **Location:** islandGroup: Hawaiian Islands; island: Oahu; verbatimLocality: Pearl Harbor, Honouliuli edge of mangrove; **Identification:** identifiedBy: K Arakaki; **Event:** verbatimEventDate: 17.vi.1998; eventRemarks: Pearl Harbor Survey; **Record Level:** institutionCode: BPBM
- **Type status:** Other material. **Occurrence:** catalogNumber: 2008000674; recordedBy: GA Samuelson, K Arakaki, K Kami; lifeStage: adult; **Taxon:** kingdom: Animalia; phylum: Arthropoda; class: Insecta; order: Diptera; family: Canacidae; genus: Procanace; specificEpithet: Procanacewilliamsi; scientificNameAuthorship: Wirth, 1951; **Location:** islandGroup: Hawaiian Islands; island: Oahu; verbatimLocality: Pearl Harbor, Honouliuli, shore; minimumElevationInMeters: 0; **Identification:** identifiedBy: K Arakaki; **Event:** verbatimEventDate: 17.vi.1998; eventRemarks: Pearl Harbor Survey; **Record Level:** institutionCode: BPBM
- **Type status:** Other material. **Occurrence:** catalogNumber: 2008000699; recordedBy: K Arakaki, K Kami; lifeStage: adult; **Taxon:** kingdom: Animalia; phylum: Arthropoda; class: Insecta; order: Diptera; family: Canacidae; genus: Procanace; specificEpithet: Procanacewilliamsi; scientificNameAuthorship: Wirth, 1951; **Location:** islandGroup: Hawaiian Islands; island: Oahu; verbatimLocality: Pearl Harbor, E'o Stream, waterline, near bridge foundation; **Identification:** identifiedBy: K Arakaki; **Event:** verbatimEventDate: 19.vi.1998; eventRemarks: Pearl Harbor Survey; **Record Level:** institutionCode: BPBM
- **Type status:** Other material. **Occurrence:** catalogNumber: 2008000698; recordedBy: K Arakaki, K Kami; lifeStage: adult; **Taxon:** kingdom: Animalia; phylum: Arthropoda; class: Insecta; order: Diptera; family: Canacidae; genus: Procanace; specificEpithet: Procanacewilliamsi; scientificNameAuthorship: Wirth, 1951; **Location:** islandGroup: Hawaiian Islands; island: Oahu; verbatimLocality: Pearl Harbor, E'o Stream, waterline, near bridge foundation; **Identification:** identifiedBy: K Arakaki; **Event:** verbatimEventDate: 19.vi.1998; eventRemarks: Pearl Harbor Survey; **Record Level:** institutionCode: BPBM
- **Type status:** Other material. **Occurrence:** catalogNumber: 2008000700; recordedBy: K Arakaki, K Kami; lifeStage: adult; **Taxon:** kingdom: Animalia; phylum: Arthropoda; class: Insecta; order: Diptera; family: Canacidae; genus: Procanace; specificEpithet: Procanacewilliamsi; scientificNameAuthorship: Wirth, 1951; **Location:** islandGroup: Hawaiian Islands; island: Oahu; verbatimLocality: E'o Stream, in canal; **Identification:** identifiedBy: K Arakaki; **Event:** verbatimEventDate: 19.vi.1998; eventRemarks: Pearl Harbor Survey; **Record Level:** institutionCode: BPBM
- **Type status:** Other material. **Occurrence:** catalogNumber: 2008000696; recordedBy: K Arakaki, K Kami; lifeStage: adult; **Taxon:** kingdom: Animalia; phylum: Arthropoda; class: Insecta; order: Diptera; family: Canacidae; genus: Procanace; specificEpithet: Procanacewilliamsi; scientificNameAuthorship: Wirth, 1951; **Location:** islandGroup: Hawaiian Islands; island: Oahu; verbatimLocality: Pearl Harbor, E'o Stream, waterline, near bridge foundation; **Identification:** identifiedBy: K Arakaki; **Event:** verbatimEventDate: 19.vi.1998; eventRemarks: Pearl Harbor Survey; **Record Level:** institutionCode: BPBM
- **Type status:** Other material. **Occurrence:** catalogNumber: 2008000697; recordedBy: K Arakaki, K Kami; lifeStage: adult; **Taxon:** kingdom: Animalia; phylum: Arthropoda; class: Insecta; order: Diptera; family: Canacidae; genus: Procanace; specificEpithet: Procanacewilliamsi; scientificNameAuthorship: Wirth, 1951; **Location:** islandGroup: Hawaiian Islands; island: Oahu; verbatimLocality: Pearl Harbor, E'o Stream, waterline, near bridge foundation; **Identification:** identifiedBy: K Arakaki; **Event:** verbatimEventDate: 19.vi.1998; eventRemarks: Pearl Harbor Survey; **Record Level:** institutionCode: BPBM
- **Type status:** Other material. **Occurrence:** catalogNumber: 2008000748; recordedBy: K Arakaki, K Kami; lifeStage: adult; **Taxon:** kingdom: Animalia; phylum: Arthropoda; class: Insecta; order: Diptera; family: Canacidae; genus: Procanace; specificEpithet: Procanacewilliamsi; scientificNameAuthorship: Wirth, 1951; **Location:** islandGroup: Hawaiian Islands; island: Oahu; verbatimLocality: Aiea Bay, rocky shoreline; **Identification:** identifiedBy: K Arakaki; **Event:** verbatimEventDate: 24.vi.1998; eventRemarks: Pearl Harbor Survey; **Record Level:** institutionCode: BPBM
- **Type status:** Other material. **Occurrence:** catalogNumber: 2008000744; recordedBy: K Arakaki, K Kami; sex: female; lifeStage: adult; **Taxon:** kingdom: Animalia; phylum: Arthropoda; class: Insecta; order: Diptera; family: Canacidae; genus: Procanace; specificEpithet: Procanacewilliamsi; scientificNameAuthorship: Wirth, 1951; **Location:** islandGroup: Hawaiian Islands; island: Oahu; verbatimLocality: Aiea Stream; **Identification:** identifiedBy: K Arakaki; **Event:** verbatimEventDate: 24.vi.1998; eventRemarks: Pearl Harbor Survey; **Record Level:** institutionCode: BPBM
- **Type status:** Other material. **Occurrence:** catalogNumber: 2008000745; recordedBy: K Arakaki, K Kami; lifeStage: adult; **Taxon:** kingdom: Animalia; phylum: Arthropoda; class: Insecta; order: Diptera; family: Canacidae; genus: Procanace; specificEpithet: Procanacewilliamsi; scientificNameAuthorship: Wirth, 1951; **Location:** islandGroup: Hawaiian Islands; island: Oahu; verbatimLocality: Aiea Bay, rocky shoreline; **Identification:** identifiedBy: K Arakaki; **Event:** verbatimEventDate: 24.vi.1998; eventRemarks: Pearl Harbor Survey; **Record Level:** institutionCode: BPBM
- **Type status:** Other material. **Occurrence:** catalogNumber: 2008000747; recordedBy: K Arakaki, K Kami; lifeStage: adult; **Taxon:** kingdom: Animalia; phylum: Arthropoda; class: Insecta; order: Diptera; family: Canacidae; genus: Procanace; specificEpithet: Procanacewilliamsi; scientificNameAuthorship: Wirth, 1951; **Location:** islandGroup: Hawaiian Islands; island: Oahu; verbatimLocality: Aiea Bay, rocky shoreline; **Identification:** identifiedBy: K Arakaki; **Event:** verbatimEventDate: 24.vi.1998; eventRemarks: Pearl Harbor Survey; **Record Level:** institutionCode: BPBM
- **Type status:** Other material. **Occurrence:** catalogNumber: 2008000749; recordedBy: K Arakaki, K Kami; lifeStage: adult; **Taxon:** kingdom: Animalia; phylum: Arthropoda; class: Insecta; order: Diptera; family: Canacidae; genus: Procanace; specificEpithet: Procanacewilliamsi; scientificNameAuthorship: Wirth, 1951; **Location:** islandGroup: Hawaiian Islands; island: Oahu; verbatimLocality: Aiea Bay, rocky shoreline; **Identification:** identifiedBy: K Arakaki; **Event:** verbatimEventDate: 24.vi.1998; eventRemarks: Pearl Harbor Survey; **Record Level:** institutionCode: BPBM
- **Type status:** Other material. **Occurrence:** catalogNumber: 2008000750; recordedBy: K Arakaki, K Kami; lifeStage: adult; **Taxon:** kingdom: Animalia; phylum: Arthropoda; class: Insecta; order: Diptera; family: Canacidae; genus: Procanace; specificEpithet: Procanacewilliamsi; scientificNameAuthorship: Wirth, 1951; **Location:** islandGroup: Hawaiian Islands; island: Oahu; verbatimLocality: Aiea Bay, rocky shoreline; **Identification:** identifiedBy: K Arakaki; **Event:** verbatimEventDate: 24.vi.1998; eventRemarks: Pearl Harbor Survey; **Record Level:** institutionCode: BPBM
- **Type status:** Other material. **Occurrence:** catalogNumber: 2008000746; recordedBy: K Arakaki, K Kami; lifeStage: adult; **Taxon:** kingdom: Animalia; phylum: Arthropoda; class: Insecta; order: Diptera; family: Canacidae; genus: Procanace; specificEpithet: Procanacewilliamsi; scientificNameAuthorship: Wirth, 1951; **Location:** islandGroup: Hawaiian Islands; island: Oahu; verbatimLocality: Aiea Bay, rocky shoreline; **Identification:** identifiedBy: K Arakaki; **Event:** verbatimEventDate: 24.vi.1998; eventRemarks: Pearl Harbor Survey; **Record Level:** institutionCode: BPBM
- **Type status:** Other material. **Occurrence:** catalogNumber: 2008000741; recordedBy: K Arakaki, GA Samuelson, K Kami; lifeStage: adult; **Taxon:** kingdom: Animalia; phylum: Arthropoda; class: Insecta; order: Diptera; family: Canacidae; genus: Procanace; specificEpithet: Procanacewilliamsi; scientificNameAuthorship: Wirth, 1951; **Location:** islandGroup: Hawaiian Islands; island: Oahu; verbatimLocality: Kalauao Ponds, Ewa Stream; minimumElevationInMeters: 0; **Identification:** identifiedBy: K Arakaki; **Event:** verbatimEventDate: 29.vi.1998; eventRemarks: Pearl Harbor Survey; **Record Level:** institutionCode: BPBM
- **Type status:** Other material. **Occurrence:** catalogNumber: 2008000742; recordedBy: K Arakaki, GA Samuelson, K Kami; lifeStage: adult; **Taxon:** kingdom: Animalia; phylum: Arthropoda; class: Insecta; order: Diptera; family: Canacidae; genus: Procanace; specificEpithet: Procanacewilliamsi; scientificNameAuthorship: Wirth, 1951; **Location:** islandGroup: Hawaiian Islands; island: Oahu; verbatimLocality: Kalauao Ponds, stream from watercress farm; minimumElevationInMeters: 0; maximumElevationInMeters: 3; **Identification:** identifiedBy: K Arakaki; **Event:** verbatimEventDate: 29.vi.1998; eventRemarks: Pearl Harbor Survey; **Record Level:** institutionCode: BPBM
- **Type status:** Other material. **Occurrence:** catalogNumber: 2008000738; recordedBy: K Arakaki, GA Samuelson, K Kami; lifeStage: adult; **Taxon:** kingdom: Animalia; phylum: Arthropoda; class: Insecta; order: Diptera; family: Canacidae; genus: Procanace; specificEpithet: Procanacewilliamsi; scientificNameAuthorship: Wirth, 1951; **Location:** islandGroup: Hawaiian Islands; island: Oahu; verbatimLocality: Kalauao Ponds, Ewa Stream; minimumElevationInMeters: 0; **Identification:** identifiedBy: K Arakaki; **Event:** verbatimEventDate: 29.vi.1998; eventRemarks: Pearl Harbor Survey; **Record Level:** institutionCode: BPBM
- **Type status:** Other material. **Occurrence:** catalogNumber: 2008000743; recordedBy: K Arakaki, GA Samuelson, K Kami; lifeStage: adult; **Taxon:** kingdom: Animalia; phylum: Arthropoda; class: Insecta; order: Diptera; family: Canacidae; genus: Procanace; specificEpithet: Procanacewilliamsi; scientificNameAuthorship: Wirth, 1951; **Location:** islandGroup: Hawaiian Islands; island: Oahu; verbatimLocality: Kalauao Ponds, stream from watercress farm; minimumElevationInMeters: 0; maximumElevationInMeters: 3; **Identification:** identifiedBy: K Arakaki; **Event:** verbatimEventDate: 29.vi.1998; eventRemarks: Pearl Harbor Survey; **Record Level:** institutionCode: BPBM
- **Type status:** Other material. **Occurrence:** catalogNumber: 2008000740; recordedBy: K Arakaki, GA Samuelson, K Kami; lifeStage: adult; **Taxon:** kingdom: Animalia; phylum: Arthropoda; class: Insecta; order: Diptera; family: Canacidae; genus: Procanace; specificEpithet: Procanacewilliamsi; scientificNameAuthorship: Wirth, 1951; **Location:** islandGroup: Hawaiian Islands; island: Oahu; verbatimLocality: Kalauao Ponds, Ewa Stream; minimumElevationInMeters: 0; **Identification:** identifiedBy: K Arakaki; **Event:** verbatimEventDate: 29.vi.1998; eventRemarks: Pearl Harbor Survey; **Record Level:** institutionCode: BPBM
- **Type status:** Other material. **Occurrence:** catalogNumber: 2008000732; recordedBy: K Arakaki, GA Samuelson, K Kami; lifeStage: adult; **Taxon:** kingdom: Animalia; phylum: Arthropoda; class: Insecta; order: Diptera; family: Canacidae; genus: Procanace; specificEpithet: Procanacewilliamsi; scientificNameAuthorship: Wirth, 1951; **Location:** islandGroup: Hawaiian Islands; island: Oahu; verbatimLocality: Kalauao Ponds, Ewa Stream; minimumElevationInMeters: 0; **Identification:** identifiedBy: K Arakaki; **Event:** verbatimEventDate: 29.vi.1998; eventRemarks: Pearl Harbor Survey; **Record Level:** institutionCode: BPBM
- **Type status:** Other material. **Occurrence:** catalogNumber: 2008000725; recordedBy: K Arakaki, GA Samuelson, K Kami; lifeStage: adult; **Taxon:** kingdom: Animalia; phylum: Arthropoda; class: Insecta; order: Diptera; family: Canacidae; genus: Procanace; specificEpithet: Procanacewilliamsi; scientificNameAuthorship: Wirth, 1951; **Location:** islandGroup: Hawaiian Islands; island: Oahu; verbatimLocality: Kalauao stream; minimumElevationInMeters: 0; **Identification:** identifiedBy: K Arakaki; **Event:** verbatimEventDate: 29.vi.1998; eventRemarks: Pearl Harbor Survey; **Record Level:** institutionCode: BPBM
- **Type status:** Other material. **Occurrence:** catalogNumber: 2008000726; recordedBy: K Arakaki, GA Samuelson, K Kami; lifeStage: adult; **Taxon:** kingdom: Animalia; phylum: Arthropoda; class: Insecta; order: Diptera; family: Canacidae; genus: Procanace; specificEpithet: Procanacewilliamsi; scientificNameAuthorship: Wirth, 1951; **Location:** islandGroup: Hawaiian Islands; island: Oahu; verbatimLocality: Kalauao Ponds, Ewa Stream; minimumElevationInMeters: 0; **Identification:** identifiedBy: K Arakaki; **Event:** verbatimEventDate: 29.vi.1998; eventRemarks: Pearl Harbor Survey; **Record Level:** institutionCode: BPBM
- **Type status:** Other material. **Occurrence:** catalogNumber: 2008000727; recordedBy: K Arakaki, GA Samuelson, K Kami; lifeStage: adult; **Taxon:** kingdom: Animalia; phylum: Arthropoda; class: Insecta; order: Diptera; family: Canacidae; genus: Procanace; specificEpithet: Procanacewilliamsi; scientificNameAuthorship: Wirth, 1951; **Location:** islandGroup: Hawaiian Islands; island: Oahu; verbatimLocality: Kalauao Ponds, Ewa Stream; minimumElevationInMeters: 0; **Identification:** identifiedBy: K Arakaki; **Event:** verbatimEventDate: 29.vi.1998; eventRemarks: Pearl Harbor Survey; **Record Level:** institutionCode: BPBM
- **Type status:** Other material. **Occurrence:** catalogNumber: 2008000728; recordedBy: K Arakaki, GA Samuelson, K Kami; lifeStage: adult; **Taxon:** kingdom: Animalia; phylum: Arthropoda; class: Insecta; order: Diptera; family: Canacidae; genus: Procanace; specificEpithet: Procanacewilliamsi; scientificNameAuthorship: Wirth, 1951; **Location:** islandGroup: Hawaiian Islands; island: Oahu; verbatimLocality: Kalauao Ponds, Ewa Stream; minimumElevationInMeters: 0; **Identification:** identifiedBy: K Arakaki; **Event:** verbatimEventDate: 29.vi.1998; eventRemarks: Pearl Harbor Survey; **Record Level:** institutionCode: BPBM
- **Type status:** Other material. **Occurrence:** catalogNumber: 2008000729; recordedBy: K Arakaki, GA Samuelson, K Kami; lifeStage: adult; **Taxon:** kingdom: Animalia; phylum: Arthropoda; class: Insecta; order: Diptera; family: Canacidae; genus: Procanace; specificEpithet: Procanacewilliamsi; scientificNameAuthorship: Wirth, 1951; **Location:** islandGroup: Hawaiian Islands; island: Oahu; verbatimLocality: Kalauao Ponds, Ewa Stream; minimumElevationInMeters: 0; **Identification:** identifiedBy: K Arakaki; **Event:** verbatimEventDate: 29.vi.1998; eventRemarks: Pearl Harbor Survey; **Record Level:** institutionCode: BPBM
- **Type status:** Other material. **Occurrence:** catalogNumber: 2008000731; recordedBy: K Arakaki, GA Samuelson, K Kami; lifeStage: adult; **Taxon:** kingdom: Animalia; phylum: Arthropoda; class: Insecta; order: Diptera; family: Canacidae; genus: Procanace; specificEpithet: Procanacewilliamsi; scientificNameAuthorship: Wirth, 1951; **Location:** islandGroup: Hawaiian Islands; island: Oahu; verbatimLocality: Kalauao Ponds, Ewa Stream; minimumElevationInMeters: 0; **Identification:** identifiedBy: K Arakaki; **Event:** verbatimEventDate: 29.vi.1998; eventRemarks: Pearl Harbor Survey; **Record Level:** institutionCode: BPBM
- **Type status:** Other material. **Occurrence:** catalogNumber: 2008000722; recordedBy: K Arakaki, GA Samuelson, K Kami; lifeStage: adult; **Taxon:** kingdom: Animalia; phylum: Arthropoda; class: Insecta; order: Diptera; family: Canacidae; genus: Procanace; specificEpithet: Procanacewilliamsi; scientificNameAuthorship: Wirth, 1951; **Location:** islandGroup: Hawaiian Islands; island: Oahu; verbatimLocality: Kalauao stream; minimumElevationInMeters: 0; **Identification:** identifiedBy: K Arakaki; **Event:** verbatimEventDate: 29.vi.1998; eventRemarks: Pearl Harbor Survey; **Record Level:** institutionCode: BPBM
- **Type status:** Other material. **Occurrence:** catalogNumber: 2008000734; recordedBy: K Arakaki, GA Samuelson, K Kami; lifeStage: adult; **Taxon:** kingdom: Animalia; phylum: Arthropoda; class: Insecta; order: Diptera; family: Canacidae; genus: Procanace; specificEpithet: Procanacewilliamsi; scientificNameAuthorship: Wirth, 1951; **Location:** islandGroup: Hawaiian Islands; island: Oahu; verbatimLocality: Kalauao Ponds, Ewa Stream; minimumElevationInMeters: 0; **Identification:** identifiedBy: K Arakaki; **Event:** verbatimEventDate: 29.vi.1998; eventRemarks: Pearl Harbor Survey; **Record Level:** institutionCode: BPBM
- **Type status:** Other material. **Occurrence:** catalogNumber: 2008000733; recordedBy: K Arakaki, GA Samuelson, K Kami; lifeStage: adult; **Taxon:** kingdom: Animalia; phylum: Arthropoda; class: Insecta; order: Diptera; family: Canacidae; genus: Procanace; specificEpithet: Procanacewilliamsi; scientificNameAuthorship: Wirth, 1951; **Location:** islandGroup: Hawaiian Islands; island: Oahu; verbatimLocality: Kalauao Ponds, Ewa Stream; minimumElevationInMeters: 0; **Identification:** identifiedBy: K Arakaki; **Event:** verbatimEventDate: 29.vi.1998; eventRemarks: Pearl Harbor Survey; **Record Level:** institutionCode: BPBM
- **Type status:** Other material. **Occurrence:** catalogNumber: 2008000735; recordedBy: K Arakaki, GA Samuelson, K Kami; lifeStage: adult; **Taxon:** kingdom: Animalia; phylum: Arthropoda; class: Insecta; order: Diptera; family: Canacidae; genus: Procanace; specificEpithet: Procanacewilliamsi; scientificNameAuthorship: Wirth, 1951; **Location:** islandGroup: Hawaiian Islands; island: Oahu; verbatimLocality: Kalauao Ponds, Ewa Stream; minimumElevationInMeters: 0; **Identification:** identifiedBy: K Arakaki; **Event:** verbatimEventDate: 29.vi.1998; eventRemarks: Pearl Harbor Survey; **Record Level:** institutionCode: BPBM
- **Type status:** Other material. **Occurrence:** catalogNumber: 2008000736; recordedBy: K Arakaki, GA Samuelson, K Kami; lifeStage: adult; **Taxon:** kingdom: Animalia; phylum: Arthropoda; class: Insecta; order: Diptera; family: Canacidae; genus: Procanace; specificEpithet: Procanacewilliamsi; scientificNameAuthorship: Wirth, 1951; **Location:** islandGroup: Hawaiian Islands; island: Oahu; verbatimLocality: Kalauao Ponds, Ewa Stream; minimumElevationInMeters: 0; **Identification:** identifiedBy: K Arakaki; **Event:** verbatimEventDate: 29.vi.1998; eventRemarks: Pearl Harbor Survey; **Record Level:** institutionCode: BPBM
- **Type status:** Other material. **Occurrence:** catalogNumber: 2008000737; recordedBy: K Arakaki, GA Samuelson, K Kami; lifeStage: adult; **Taxon:** kingdom: Animalia; phylum: Arthropoda; class: Insecta; order: Diptera; family: Canacidae; genus: Procanace; specificEpithet: Procanacewilliamsi; scientificNameAuthorship: Wirth, 1951; **Location:** islandGroup: Hawaiian Islands; island: Oahu; verbatimLocality: Kalauao Ponds, Ewa Stream; minimumElevationInMeters: 0; **Identification:** identifiedBy: K Arakaki; **Event:** verbatimEventDate: 29.vi.1998; eventRemarks: Pearl Harbor Survey; **Record Level:** institutionCode: BPBM
- **Type status:** Other material. **Occurrence:** catalogNumber: 2008000730; recordedBy: K Arakaki, GA Samuelson, K Kami; lifeStage: adult; **Taxon:** kingdom: Animalia; phylum: Arthropoda; class: Insecta; order: Diptera; family: Canacidae; genus: Procanace; specificEpithet: Procanacewilliamsi; scientificNameAuthorship: Wirth, 1951; **Location:** islandGroup: Hawaiian Islands; island: Oahu; verbatimLocality: Kalauao Ponds, Ewa Stream; minimumElevationInMeters: 0; **Identification:** identifiedBy: K Arakaki; **Event:** verbatimEventDate: 29.vi.1998; eventRemarks: Pearl Harbor Survey; **Record Level:** institutionCode: BPBM
- **Type status:** Other material. **Occurrence:** catalogNumber: 2008000724; recordedBy: K Arakaki, GA Samuelson, K Kami; lifeStage: adult; **Taxon:** kingdom: Animalia; phylum: Arthropoda; class: Insecta; order: Diptera; family: Canacidae; genus: Procanace; specificEpithet: Procanacewilliamsi; scientificNameAuthorship: Wirth, 1951; **Location:** islandGroup: Hawaiian Islands; island: Oahu; verbatimLocality: Kalauao stream; minimumElevationInMeters: 0; **Identification:** identifiedBy: K Arakaki; **Event:** verbatimEventDate: 29.vi.1998; eventRemarks: Pearl Harbor Survey; **Record Level:** institutionCode: BPBM
- **Type status:** Other material. **Occurrence:** catalogNumber: 2008000723; recordedBy: K Arakaki, GA Samuelson, K Kami; lifeStage: adult; **Taxon:** kingdom: Animalia; phylum: Arthropoda; class: Insecta; order: Diptera; family: Canacidae; genus: Procanace; specificEpithet: Procanacewilliamsi; scientificNameAuthorship: Wirth, 1951; **Location:** islandGroup: Hawaiian Islands; island: Oahu; verbatimLocality: Kalauao stream; minimumElevationInMeters: 0; **Identification:** identifiedBy: K Arakaki; **Event:** verbatimEventDate: 29.vi.1998; eventRemarks: Pearl Harbor Survey; **Record Level:** institutionCode: BPBM
- **Type status:** Other material. **Occurrence:** catalogNumber: 2008000721; recordedBy: K Arakaki, GA Samuelson, K Kami; lifeStage: adult; **Taxon:** kingdom: Animalia; phylum: Arthropoda; class: Insecta; order: Diptera; family: Canacidae; genus: Procanace; specificEpithet: Procanacewilliamsi; scientificNameAuthorship: Wirth, 1951; **Location:** islandGroup: Hawaiian Islands; island: Oahu; verbatimLocality: Kalauao stream; minimumElevationInMeters: 0; **Identification:** identifiedBy: K Arakaki; **Event:** verbatimEventDate: 29.vi.1998; eventRemarks: Pearl Harbor Survey; **Record Level:** institutionCode: BPBM
- **Type status:** Other material. **Occurrence:** catalogNumber: 2008000739; recordedBy: K Arakaki, GA Samuelson, K Kami; lifeStage: adult; **Taxon:** kingdom: Animalia; phylum: Arthropoda; class: Insecta; order: Diptera; family: Canacidae; genus: Procanace; specificEpithet: Procanacewilliamsi; scientificNameAuthorship: Wirth, 1951; **Location:** islandGroup: Hawaiian Islands; island: Oahu; verbatimLocality: Kalauao Ponds, Ewa Stream; minimumElevationInMeters: 0; **Identification:** identifiedBy: K Arakaki; **Event:** verbatimEventDate: 29.vi.1998; eventRemarks: Pearl Harbor Survey; **Record Level:** institutionCode: BPBM
- **Type status:** Other material. **Occurrence:** catalogNumber: 2008000710; recordedBy: K Arakaki, K Kami; lifeStage: adult; **Taxon:** kingdom: Animalia; phylum: Arthropoda; class: Insecta; order: Diptera; family: Canacidae; genus: Procanace; specificEpithet: Procanacewilliamsi; scientificNameAuthorship: Wirth, 1951; **Location:** islandGroup: Hawaiian Islands; island: Oahu; verbatimLocality: Waiau Hawaiian Electric Power Plant, at ewa end; minimumElevationInMeters: 0; **Identification:** identifiedBy: K Arakaki; **Event:** verbatimEventDate: 30.vi.1998; eventRemarks: Pearl Harbor Survey; **Record Level:** institutionCode: BPBM
- **Type status:** Other material. **Occurrence:** catalogNumber: 2008000711; recordedBy: K Arakaki, K Kami; lifeStage: adult; **Taxon:** kingdom: Animalia; phylum: Arthropoda; class: Insecta; order: Diptera; family: Canacidae; genus: Procanace; specificEpithet: Procanacewilliamsi; scientificNameAuthorship: Wirth, 1951; **Location:** islandGroup: Hawaiian Islands; island: Oahu; verbatimLocality: Waiau Hawaiian Electric Power Plant, at ewa end; minimumElevationInMeters: 0; **Identification:** identifiedBy: K Arakaki; **Event:** verbatimEventDate: 30.vi.1998; eventRemarks: Pearl Harbor Survey; **Record Level:** institutionCode: BPBM
- **Type status:** Other material. **Occurrence:** catalogNumber: 2008000712; recordedBy: K Arakaki, K Kami; lifeStage: adult; **Taxon:** kingdom: Animalia; phylum: Arthropoda; class: Insecta; order: Diptera; family: Canacidae; genus: Procanace; specificEpithet: Procanacewilliamsi; scientificNameAuthorship: Wirth, 1951; **Location:** islandGroup: Hawaiian Islands; island: Oahu; verbatimLocality: Waiau Hawaiian Electric Power Plant, at ewa end; minimumElevationInMeters: 0; **Identification:** identifiedBy: K Arakaki; **Event:** verbatimEventDate: 30.vi.1998; eventRemarks: Pearl Harbor Survey; **Record Level:** institutionCode: BPBM
- **Type status:** Other material. **Occurrence:** catalogNumber: 2008000713; recordedBy: K Arakaki, K Kami; lifeStage: adult; **Taxon:** kingdom: Animalia; phylum: Arthropoda; class: Insecta; order: Diptera; family: Canacidae; genus: Procanace; specificEpithet: Procanacewilliamsi; scientificNameAuthorship: Wirth, 1951; **Location:** islandGroup: Hawaiian Islands; island: Oahu; verbatimLocality: Waiau Hawaiian Electric Power Plant, at ewa end; minimumElevationInMeters: 0; **Identification:** identifiedBy: K Arakaki; **Event:** verbatimEventDate: 30.vi.1998; eventRemarks: Pearl Harbor Survey; **Record Level:** institutionCode: BPBM
- **Type status:** Other material. **Occurrence:** catalogNumber: 2008000701; recordedBy: K Arakaki, GA Samuelson, K Kami; lifeStage: adult; **Taxon:** kingdom: Animalia; phylum: Arthropoda; class: Insecta; order: Diptera; family: Canacidae; genus: Procanace; specificEpithet: Procanacewilliamsi; scientificNameAuthorship: Wirth, 1951; **Location:** islandGroup: Hawaiian Islands; island: Oahu; verbatimLocality: E'o Canal, Ted Makalena Golf Course, at shore line; **Identification:** identifiedBy: K Arakaki; **Event:** verbatimEventDate: 27.vii.1998; eventRemarks: Pearl Harbor Survey; **Record Level:** institutionCode: BPBM
- **Type status:** Other material. **Occurrence:** catalogNumber: 2008000702; recordedBy: K Arakaki, GA Samuelson, K Kami; lifeStage: adult; **Taxon:** kingdom: Animalia; phylum: Arthropoda; class: Insecta; order: Diptera; family: Canacidae; genus: Procanace; specificEpithet: Procanacewilliamsi; scientificNameAuthorship: Wirth, 1951; **Location:** islandGroup: Hawaiian Islands; island: Oahu; verbatimLocality: E'o Canal, Ted Makalena Golf Course, at shore line; **Identification:** identifiedBy: K Arakaki; **Event:** verbatimEventDate: 27.vii.1998; eventRemarks: Pearl Harbor Survey; **Record Level:** institutionCode: BPBM

##### Ecological interactions

###### Native status

adventive

##### Distribution

HAWAIIAN ISLANDS: Oahu (Fig. 13​); JAPAN.

##### Notes

Wirth (1951), [original description; male genitalia (ventral and lateral)]; Hardy and Delfinado (1980), [redescription and revision of Hawaiian taxa; head (front and lateral), female terminalia (dorsal and ventral), spermathecae, wing, surstylus; cephalopharyngael skeleton (larval and pupal, puparium, third instar larvae]; Mathis (1992), [World Catalog]; Nishida (2002), [Hawaiian Arthropod Checklist].

#### Procanace wirthi

Hardy and Delfinado, 1980

##### Materials

- **Type status:** Holotype. **Occurrence:** recordedBy: MD Delfinado; individualCount: 1; sex: male; lifeStage: adult; **Taxon:** kingdom: Animalia; phylum: Arthropoda; class: Insecta; order: Diptera; family: Canacidae; genus: Procanace; specificEpithet: Procanacewirthi; scientificNameAuthorship: Hardy & Delfinado, 1980; **Location:** islandGroup: Hawaiian Islands; island: Oahu; verbatimLocality: Maunawili Stream, on wet rocks; verbatimElevation: 800 ft.; **Identification:** identifiedBy: DE Hardy & MD Delfinado; dateIdentified: 1980; **Event:** verbatimEventDate: 15.iv.1970; **Record Level:** institutionCode: BPBM
- **Type status:** Paratype. **Occurrence:** recordedBy: FX Williams; lifeStage: adult; **Taxon:** kingdom: Animalia; phylum: Arthropoda; class: Insecta; order: Diptera; family: Canacidae; genus: Procanace; specificEpithet: Procanacewirthi; scientificNameAuthorship: Hardy & Delfinado, 1980; **Location:** islandGroup: Hawaiian Islands; island: Oahu; verbatimLocality: Hering Valley, Tantalus, wet rocks near waterfall; **Identification:** identifiedBy: DE Hardy & MD Delfinado; dateIdentified: 1980; **Event:** verbatimEventDate: 30.vii.1933
- **Type status:** Paratype. **Occurrence:** catalogNumber: 2006005169; recordedBy: FX Williams; lifeStage: adult; **Taxon:** kingdom: Animalia; phylum: Arthropoda; class: Insecta; order: Diptera; family: Canacidae; genus: Procanace; specificEpithet: Procanacewirthi; scientificNameAuthorship: Hardy & Delfinado, 1980; **Location:** islandGroup: Hawaiian Islands; island: Oahu; verbatimLocality: Honolulu, Hering Valley, Tantalus; **Identification:** identifiedBy: DE Hardy & MD Delfinado; dateIdentified: 1980; **Event:** verbatimEventDate: 30.vii.1933; **Record Level:** institutionCode: BPBM
- **Type status:** Paratype. **Occurrence:** recordedBy: FX Williams; lifeStage: adult; **Taxon:** kingdom: Animalia; phylum: Arthropoda; class: Insecta; order: Diptera; family: Canacidae; genus: Procanace; specificEpithet: Procanacewirthi; scientificNameAuthorship: Hardy & Delfinado, 1980; **Location:** islandGroup: Hawaiian Islands; island: Oahu; verbatimLocality: Konahuinui, on stream rocks; **Identification:** identifiedBy: DE Hardy & MD Delfinado; dateIdentified: 1980; **Event:** verbatimEventDate: 12.v.1935
- **Type status:** Paratype. **Occurrence:** catalogNumber: 2006005167; recordedBy: FX Williams; lifeStage: adult; **Taxon:** kingdom: Animalia; phylum: Arthropoda; class: Insecta; order: Diptera; family: Canacidae; genus: Procanace; specificEpithet: Procanacewirthi; scientificNameAuthorship: Hardy & Delfinado, 1980; **Location:** islandGroup: Hawaiian Islands; island: Oahu; verbatimLocality: Konahuanui; **Identification:** identifiedBy: DE Hardy & MD Delfinado; dateIdentified: 1980; **Event:** verbatimEventDate: 12.v.1935; **Record Level:** institutionCode: BPBM
- **Type status:** Paratype. **Occurrence:** recordedBy: FX Williams; lifeStage: adult; **Taxon:** kingdom: Animalia; phylum: Arthropoda; class: Insecta; order: Diptera; family: Canacidae; genus: Procanace; specificEpithet: Procanacewirthi; scientificNameAuthorship: Hardy & Delfinado, 1980; **Location:** islandGroup: Hawaiian Islands; island: Oahu; verbatimLocality: Waihi-iki, Manoa Valley; **Identification:** identifiedBy: DE Hardy & MD Delfinado; dateIdentified: 1980; **Event:** verbatimEventDate: 22.iii.1936
- **Type status:** Paratype. **Occurrence:** catalogNumber: 2006005166; recordedBy: FX Williams; sex: female; lifeStage: adult; **Taxon:** kingdom: Animalia; phylum: Arthropoda; class: Insecta; order: Diptera; family: Canacidae; genus: Procanace; specificEpithet: Procanacewirthi; scientificNameAuthorship: Hardy & Delfinado, 1980; **Location:** islandGroup: Hawaiian Islands; island: Oahu; verbatimLocality: Manoa Valley, Waihi-iki; **Identification:** identifiedBy: DE Hardy & MD Delfinado; dateIdentified: 1980; **Event:** verbatimEventDate: 22.iii.1936; **Record Level:** institutionCode: BPBM
- **Type status:** Paratype. **Occurrence:** catalogNumber: 2006005171; recordedBy: FX Williams; sex: female; lifeStage: adult; **Taxon:** kingdom: Animalia; phylum: Arthropoda; class: Insecta; order: Diptera; family: Canacidae; genus: Procanace; specificEpithet: Procanacewirthi; scientificNameAuthorship: Hardy & Delfinado, 1980; **Location:** islandGroup: Hawaiian Islands; island: Oahu; verbatimLocality: Manoa Valley, Waihi-iki; **Identification:** identifiedBy: DE Hardy & MD Delfinado; dateIdentified: 1980; **Event:** verbatimEventDate: 22.iii.1936; **Record Level:** institutionCode: BPBM
- **Type status:** Paratype. **Occurrence:** catalogNumber: 2006005176; recordedBy: FX Williams; lifeStage: adult; **Taxon:** kingdom: Animalia; phylum: Arthropoda; class: Insecta; order: Diptera; family: Canacidae; genus: Procanace; specificEpithet: Procanacewirthi; scientificNameAuthorship: Hardy & Delfinado, 1980; **Location:** islandGroup: Hawaiian Islands; island: Oahu; verbatimLocality: Waianae; **Identification:** identifiedBy: DE Hardy & MD Delfinado; dateIdentified: 1980; **Event:** verbatimEventDate: 19.iv.1936; **Record Level:** institutionCode: BPBM
- **Type status:** Paratype. **Occurrence:** recordedBy: FX Williams; lifeStage: adult; **Taxon:** kingdom: Animalia; phylum: Arthropoda; class: Insecta; order: Diptera; family: Canacidae; genus: Procanace; specificEpithet: Procanacewirthi; scientificNameAuthorship: Hardy & Delfinado, 1980; **Location:** islandGroup: Hawaiian Islands; island: Oahu; verbatimLocality: Waianae, swift water stone ditch; **Identification:** identifiedBy: DE Hardy & MD Delfinado; dateIdentified: 1980; **Event:** verbatimEventDate: vii.1936
- **Type status:** Paratype. **Occurrence:** catalogNumber: 2006005168; recordedBy: FX Williams; lifeStage: adult; **Taxon:** kingdom: Animalia; phylum: Arthropoda; class: Insecta; order: Diptera; family: Canacidae; genus: Procanace; specificEpithet: Procanacewirthi; scientificNameAuthorship: Hardy & Delfinado, 1980; **Location:** islandGroup: Hawaiian Islands; island: Oahu; verbatimLocality: Waianae; **Identification:** identifiedBy: DE Hardy & MD Delfinado; dateIdentified: 1980; **Event:** verbatimEventDate: 19.vii.1936; **Record Level:** institutionCode: BPBM
- **Type status:** Paratype. **Occurrence:** catalogNumber: 2006005175; recordedBy: FX Williams; lifeStage: adult; **Taxon:** kingdom: Animalia; phylum: Arthropoda; class: Insecta; order: Diptera; family: Canacidae; genus: Procanace; specificEpithet: Procanacewirthi; scientificNameAuthorship: Hardy & Delfinado, 1980; **Location:** islandGroup: Hawaiian Islands; island: Oahu; verbatimLocality: Waianae; **Identification:** identifiedBy: DE Hardy & MD Delfinado; dateIdentified: 1980; **Event:** verbatimEventDate: 19.vii.1936; **Record Level:** institutionCode: BPBM
- **Type status:** Paratype. **Occurrence:** recordedBy: FX Williams; lifeStage: adult; **Taxon:** kingdom: Animalia; phylum: Arthropoda; class: Insecta; order: Diptera; family: Canacidae; genus: Procanace; specificEpithet: Procanacewirthi; scientificNameAuthorship: Hardy & Delfinado, 1980; **Location:** islandGroup: Hawaiian Islands; island: Oahu; verbatimLocality: Kaluanui Stream; verbatimElevation: 2000 ft.; **Identification:** identifiedBy: DE Hardy & MD Delfinado; dateIdentified: 1980; **Event:** verbatimEventDate: 18.x.1936
- **Type status:** Paratype. **Occurrence:** catalogNumber: 2006005170; recordedBy: FX Williams; individualCount: 2; lifeStage: adult; **Taxon:** kingdom: Animalia; phylum: Arthropoda; class: Insecta; order: Diptera; family: Canacidae; genus: Procanace; specificEpithet: Procanacewirthi; scientificNameAuthorship: Hardy & Delfinado, 1980; **Location:** islandGroup: Hawaiian Islands; island: Oahu; verbatimLocality: Kaluanui Stream; minimumElevationInMeters: 2000; **Identification:** identifiedBy: DE Hardy & MD Delfinado; dateIdentified: 1980; **Event:** verbatimEventDate: 18.x.1936; **Record Level:** institutionCode: BPBM
- **Type status:** Paratype. **Occurrence:** catalogNumber: 2006005174; recordedBy: FX Williams; sex: male; lifeStage: adult; **Taxon:** kingdom: Animalia; phylum: Arthropoda; class: Insecta; order: Diptera; family: Canacidae; genus: Procanace; specificEpithet: Procanacewirthi; scientificNameAuthorship: Hardy & Delfinado, 1980; **Location:** islandGroup: Hawaiian Islands; island: Oahu; verbatimLocality: Kaluanui Stream; minimumElevationInMeters: 2000; **Identification:** identifiedBy: DE Hardy & MD Delfinado; dateIdentified: 1980; **Event:** verbatimEventDate: 18.x.1936; **Record Level:** institutionCode: BPBM
- **Type status:** Paratype. **Occurrence:** catalogNumber: 2006005172; recordedBy: FX Williams; lifeStage: adult; **Taxon:** kingdom: Animalia; phylum: Arthropoda; class: Insecta; order: Diptera; family: Canacidae; genus: Procanace; specificEpithet: Procanacewirthi; scientificNameAuthorship: Hardy & Delfinado, 1980; **Location:** islandGroup: Hawaiian Islands; island: Oahu; verbatimLocality: Honolulu Kalihi Stream; **Identification:** identifiedBy: DE Hardy & MD Delfinado; dateIdentified: 1980; **Event:** verbatimEventDate: 13.iii.1937; **Record Level:** institutionCode: BPBM
- **Type status:** Paratype. **Occurrence:** recordedBy: FX Williams; lifeStage: adult; **Taxon:** kingdom: Animalia; phylum: Arthropoda; class: Insecta; order: Diptera; family: Canacidae; genus: Procanace; specificEpithet: Procanacewirthi; scientificNameAuthorship: Hardy & Delfinado, 1980; **Location:** islandGroup: Hawaiian Islands; island: Oahu; verbatimLocality: Punaluu Stream; **Identification:** identifiedBy: DE Hardy & MD Delfinado; dateIdentified: 1980; **Event:** verbatimEventDate: 28.xi.1937
- **Type status:** Paratype. **Occurrence:** catalogNumber: 2006005173; recordedBy: FX Williams; sex: male; lifeStage: adult; **Taxon:** kingdom: Animalia; phylum: Arthropoda; class: Insecta; order: Diptera; family: Canacidae; genus: Procanace; specificEpithet: Procanacewirthi; scientificNameAuthorship: Hardy & Delfinado, 1980; **Location:** islandGroup: Hawaiian Islands; island: Oahu; verbatimLocality: Punaluu; minimumElevationInMeters: 800; **Identification:** identifiedBy: DE Hardy & MD Delfinado; dateIdentified: 1980; **Event:** verbatimEventDate: 28.xi.1937; **Record Level:** institutionCode: BPBM
- **Type status:** Paratype. **Occurrence:** recordedBy: FX Williams, D Ashdow; lifeStage: adult; **Taxon:** kingdom: Animalia; phylum: Arthropoda; class: Insecta; order: Diptera; family: Canacidae; genus: Procanace; specificEpithet: Procanacewirthi; scientificNameAuthorship: Hardy & Delfinado, 1980; **Location:** islandGroup: Hawaiian Islands; island: Oahu; verbatimLocality: Kalihi Stream, wet boulder; **Identification:** identifiedBy: DE Hardy & MD Delfinado; dateIdentified: 1980; **Event:** verbatimEventDate: 13.iii.1957
- **Type status:** Paratype. **Occurrence:** recordedBy: FX Williams, D Ashdown; lifeStage: adult; **Taxon:** kingdom: Animalia; phylum: Arthropoda; class: Insecta; order: Diptera; family: Canacidae; genus: Procanace; specificEpithet: Procanacewirthi; scientificNameAuthorship: Hardy & Delfinado, 1980; **Location:** islandGroup: Hawaiian Islands; island: Oahu; verbatimLocality: Punaluu Stream; **Identification:** identifiedBy: DE Hardy & MD Delfinado; dateIdentified: 1980; **Event:** verbatimEventDate: 12.vi.1968
- **Type status:** Paratype. **Occurrence:** recordedBy: MD Delfinado; individualCount: 12; lifeStage: adult; **Taxon:** kingdom: Animalia; phylum: Arthropoda; class: Insecta; order: Diptera; family: Canacidae; genus: Procanace; specificEpithet: Procanacewirthi; scientificNameAuthorship: Hardy & Delfinado, 1980; **Location:** islandGroup: Hawaiian Islands; island: Oahu; verbatimLocality: Maunawili Stream; verbatimElevation: 800 ft., on rocks; **Identification:** identifiedBy: DE Hardy & MD Delfinado; dateIdentified: 1980; **Event:** verbatimEventDate: 15.iv.1970; **Record Level:** institutionCode: UHM
- **Type status:** Paratype. **Occurrence:** catalogNumber: 2006005178; recordedBy: MD Delfinado; lifeStage: adult; **Taxon:** kingdom: Animalia; phylum: Arthropoda; class: Insecta; order: Diptera; family: Canacidae; genus: Procanace; specificEpithet: Procanacewirthi; scientificNameAuthorship: Hardy & Delfinado, 1980; **Location:** islandGroup: Hawaiian Islands; island: Oahu; verbatimLocality: Maunawili Stream; minimumElevationInMeters: 800; **Identification:** identifiedBy: DE Hardy & MD Delfinado; dateIdentified: 1980; **Event:** verbatimEventDate: 15.iv.1970; **Record Level:** institutionCode: BPBM
- **Type status:** Paratype. **Occurrence:** recordedBy: MD Delfinado; individualCount: 1; sex: female; lifeStage: adult; **Taxon:** kingdom: Animalia; phylum: Arthropoda; class: Insecta; order: Diptera; family: Canacidae; genus: Procanace; specificEpithet: Procanacewirthi; scientificNameAuthorship: Hardy & Delfinado, 1980; **Location:** islandGroup: Hawaiian Islands; island: Oahu; verbatimLocality: Maunawili Stream, on wet rocks; verbatimElevation: 800 ft.; **Identification:** identifiedBy: DE Hardy & MD Delfinado; dateIdentified: 1980; **Event:** verbatimEventDate: 15.iv.1970; **Record Level:** institutionCode: BPBM
- **Type status:** Paratype. **Occurrence:** recordedBy: MD Delfinado; lifeStage: adult; **Taxon:** kingdom: Animalia; phylum: Arthropoda; class: Insecta; order: Diptera; family: Canacidae; genus: Procanace; specificEpithet: Procanacewirthi; scientificNameAuthorship: Hardy & Delfinado, 1980; **Location:** islandGroup: Hawaiian Islands; island: Oahu; verbatimLocality: Maunawili Stream, on wet rocks; verbatimElevation: 800 ft.; **Identification:** identifiedBy: DE Hardy & MD Delfinado; dateIdentified: 1980; **Event:** verbatimEventDate: 15.iv.1970
- **Type status:** Paratype. **Occurrence:** catalogNumber: 2006005177; recordedBy: MD Delfinado; lifeStage: adult; **Taxon:** kingdom: Animalia; phylum: Arthropoda; class: Insecta; order: Diptera; family: Canacidae; genus: Procanace; specificEpithet: Procanacewirthi; scientificNameAuthorship: Hardy & Delfinado, 1980; **Location:** islandGroup: Hawaiian Islands; island: Oahu; verbatimLocality: Maunawili Stream; minimumElevationInMeters: 800; **Identification:** identifiedBy: DE Hardy & MD Delfinado; dateIdentified: 1980; **Event:** verbatimEventDate: 15.iv.1970; **Record Level:** institutionCode: BPBM
- **Type status:** Paratype. **Occurrence:** recordedBy: MD Delfinado; individualCount: 3; sex: 2 males, 1 female; lifeStage: adult; **Taxon:** kingdom: Animalia; phylum: Arthropoda; class: Insecta; order: Diptera; family: Canacidae; genus: Procanace; specificEpithet: Procanacewirthi; scientificNameAuthorship: Hardy & Delfinado, 1980; **Location:** islandGroup: Hawaiian Islands; island: Oahu; verbatimLocality: Maunawili Stream; verbatimElevation: 800 ft., on rocks; **Identification:** identifiedBy: DE Hardy & MD Delfinado; dateIdentified: 1980; **Event:** verbatimEventDate: 15.iv.1970; **Record Level:** institutionCode: USNM
- **Type status:** Paratype. **Occurrence:** recordedBy: MD Delfinado; sex: 1 female; lifeStage: adult; **Taxon:** kingdom: Animalia; phylum: Arthropoda; class: Insecta; order: Diptera; family: Canacidae; genus: Procanace; specificEpithet: Procanacewirthi; scientificNameAuthorship: Hardy & Delfinado, 1980; **Location:** islandGroup: Hawaiian Islands; island: Oahu; verbatimLocality: Kaluanui Stream, on wet rocks; minimumElevationInMeters: 800; **Identification:** identifiedBy: DE Hardy & MD Delfinado; dateIdentified: 1980; **Event:** verbatimEventDate: 22.iv.1970; **Record Level:** institutionCode: USNM
- **Type status:** Other material. **Occurrence:** recordedBy: DE Hardy; individualCount: 4; sex: 4 males; lifeStage: adult; **Taxon:** kingdom: Animalia; phylum: Arthropoda; class: Insecta; order: Diptera; family: Canacidae; genus: Procanace; specificEpithet: Procanacewirthi; scientificNameAuthorship: Hardy & Delfinado, 1980; **Location:** islandGroup: Hawaiian Islands; island: Oahu; verbatimLocality: Kahana Stream; verbatimElevation: 180 ft.; **Event:** verbatimEventDate: 27.viii.1970; **Record Level:** institutionCode: USNM
- **Type status:** Other material. **Occurrence:** recordedBy: DE Hardy; individualCount: 5; lifeStage: adult; **Taxon:** kingdom: Animalia; phylum: Arthropoda; class: Insecta; order: Diptera; family: Canacidae; genus: Procanace; specificEpithet: Procanacewirthi; scientificNameAuthorship: Hardy & Delfinado, 1980; **Location:** islandGroup: Hawaiian Islands; island: Oahu; verbatimLocality: Kahana Valley; verbatimElevation: 600 ft.; **Event:** verbatimEventDate: 17.ix.1970; **Record Level:** institutionCode: UHM
- **Type status:** Other material. **Occurrence:** recordedBy: JA Tenorio; individualCount: 3; sex: 2 males, 1 female; lifeStage: adult; **Taxon:** kingdom: Animalia; phylum: Arthropoda; class: Insecta; order: Diptera; family: Canacidae; genus: Procanace; specificEpithet: Procanacewirthi; scientificNameAuthorship: Hardy & Delfinado, 1980; **Location:** islandGroup: Hawaiian Islands; island: Oahu; verbatimLocality: Kahana Stream, slow flowing stream; verbatimElevation: 800 ft.; **Event:** verbatimEventDate: 17.ix.1970; **Record Level:** institutionCode: USNM
- **Type status:** Paratype. **Occurrence:** recordedBy: DE Hardy, MD Delfinado; lifeStage: adult; **Taxon:** kingdom: Animalia; phylum: Arthropoda; class: Insecta; order: Diptera; family: Canacidae; genus: Procanace; specificEpithet: Procanacewirthi; scientificNameAuthorship: Hardy & Delfinado, 1980; **Location:** islandGroup: Hawaiian Islands; island: Kauai; verbatimLocality: Hanakapiai River; verbatimElevation: sea level to 600 ft.; **Identification:** identifiedBy: DE Hardy & MD Delfinado; dateIdentified: 1980; **Event:** verbatimEventDate: 10.viii.1971
- **Type status:** Other material. **Occurrence:** recordedBy: DA Polhemus; individualCount: 2; sex: 1 male, 1 female; lifeStage: adult; **Taxon:** kingdom: Animalia; phylum: Arthropoda; class: Insecta; order: Diptera; family: Canacidae; genus: Procanace; specificEpithet: Procanacewirthi; scientificNameAuthorship: Hardy & Delfinado, 1980; **Location:** islandGroup: Hawaiian Islands; island: Maui; verbatimLocality: Iao Valley, West Maui; **Event:** verbatimEventDate: 30.x.1990; **Record Level:** institutionCode: USNM
- **Type status:** Other material. **Occurrence:** catalogNumber: 2006008034; recordedBy: DA Polhemus; lifeStage: adult; **Taxon:** kingdom: Animalia; phylum: Arthropoda; class: Insecta; order: Diptera; family: Canacidae; genus: Procanace; specificEpithet: Procanacewirthi; scientificNameAuthorship: Hardy & Delfinado, 1980; **Location:** islandGroup: Hawaiian Islands; island: Kauai; verbatimLocality: Makaleha Stream, at Makaleha Springs; minimumElevationInMeters: 787; **Identification:** identifiedBy: WN Mathis; dateIdentified: 1992; **Event:** verbatimEventDate: 08.xi.1990; **Record Level:** institutionCode: BPBM
- **Type status:** Other material. **Occurrence:** recordedBy: DA Polhemus; individualCount: 7; sex: 4 males, 3 females; lifeStage: adult; **Taxon:** kingdom: Animalia; phylum: Arthropoda; class: Insecta; order: Diptera; family: Canacidae; genus: Procanace; specificEpithet: Procanacewirthi; scientificNameAuthorship: Hardy & Delfinado, 1980; **Location:** islandGroup: Hawaiian Islands; island: Kauai; verbatimLocality: Makaleha Stream, at Makaleha Springs; verbatimElevation: 240; **Identification:** identifiedBy: WN Mathis; dateIdentified: 1992; **Event:** verbatimEventDate: 08.xi.1990; **Record Level:** institutionCode: USNM
- **Type status:** Other material. **Occurrence:** catalogNumber: 2006008033; recordedBy: DA Polhemus; lifeStage: adult; **Taxon:** kingdom: Animalia; phylum: Arthropoda; class: Insecta; order: Diptera; family: Canacidae; genus: Procanace; specificEpithet: Procanacewirthi; scientificNameAuthorship: Hardy & Delfinado, 1980; **Location:** islandGroup: Hawaiian Islands; island: Kauai; verbatimLocality: Makaleha Stream, at Makaleha Springs; minimumElevationInMeters: 787; **Identification:** identifiedBy: WN Mathis; dateIdentified: 1992; **Event:** verbatimEventDate: 08.xi.1990; **Record Level:** institutionCode: BPBM
- **Type status:** Other material. **Occurrence:** catalogNumber: 2006008035; recordedBy: DA Polhemus; lifeStage: adult; **Taxon:** kingdom: Animalia; phylum: Arthropoda; class: Insecta; order: Diptera; family: Canacidae; genus: Procanace; specificEpithet: Procanacewirthi; scientificNameAuthorship: Hardy & Delfinado, 1980; **Location:** islandGroup: Hawaiian Islands; island: Kauai; verbatimLocality: Makaleha Stream, at Makaleha Springs; minimumElevationInMeters: 787; **Identification:** identifiedBy: WN Mathis; dateIdentified: 1992; **Event:** verbatimEventDate: 08.xi.1990; **Record Level:** institutionCode: BPBM
- **Type status:** Other material. **Occurrence:** catalogNumber: 2006008026; recordedBy: DA Polhemus; lifeStage: adult; **Taxon:** kingdom: Animalia; phylum: Arthropoda; class: Insecta; order: Diptera; family: Canacidae; genus: Procanace; specificEpithet: Procanacewirthi; scientificNameAuthorship: Hardy & Delfinado, 1980; **Location:** islandGroup: Hawaiian Islands; island: Oahu; verbatimLocality: Kaneohe, near Waihee Stream; minimumElevationInMeters: 705; **Identification:** identifiedBy: WN Mathis; dateIdentified: 1992; **Event:** verbatimEventDate: 22.xi.1991; **Record Level:** institutionCode: BPBM
- **Type status:** Other material. **Occurrence:** catalogNumber: 2006008028; recordedBy: DA Polhemus; lifeStage: adult; **Taxon:** kingdom: Animalia; phylum: Arthropoda; class: Insecta; order: Diptera; family: Canacidae; genus: Procanace; specificEpithet: Procanacewirthi; scientificNameAuthorship: Hardy & Delfinado, 1980; **Location:** islandGroup: Hawaiian Islands; island: Oahu; verbatimLocality: Kaneohe, near Waihee Stream; minimumElevationInMeters: 705; **Identification:** identifiedBy: WN Mathis; dateIdentified: 1992; **Event:** verbatimEventDate: 22.xi.1991; **Record Level:** institutionCode: BPBM
- **Type status:** Other material. **Occurrence:** catalogNumber: 2006008029; recordedBy: DA Polhemus; lifeStage: adult; **Taxon:** kingdom: Animalia; phylum: Arthropoda; class: Insecta; order: Diptera; family: Canacidae; genus: Procanace; specificEpithet: Procanacewirthi; scientificNameAuthorship: Hardy & Delfinado, 1980; **Location:** islandGroup: Hawaiian Islands; island: Oahu; verbatimLocality: Kaneohe, near Waihee Stream; minimumElevationInMeters: 705; **Identification:** identifiedBy: WN Mathis; dateIdentified: 1992; **Event:** verbatimEventDate: 22.xi.1991; **Record Level:** institutionCode: BPBM
- **Type status:** Other material. **Occurrence:** catalogNumber: 2006008030; recordedBy: DA Polhemus; lifeStage: adult; **Taxon:** kingdom: Animalia; phylum: Arthropoda; class: Insecta; order: Diptera; family: Canacidae; genus: Procanace; specificEpithet: Procanacewirthi; scientificNameAuthorship: Hardy & Delfinado, 1980; **Location:** islandGroup: Hawaiian Islands; island: Oahu; verbatimLocality: Kaneohe, near Waihee Stream; minimumElevationInMeters: 705; **Identification:** identifiedBy: WN Mathis; dateIdentified: 1992; **Event:** verbatimEventDate: 22.xi.1991; **Record Level:** institutionCode: BPBM
- **Type status:** Other material. **Occurrence:** catalogNumber: 2006008031; recordedBy: DA Polhemus; lifeStage: adult; **Taxon:** kingdom: Animalia; phylum: Arthropoda; class: Insecta; order: Diptera; family: Canacidae; genus: Procanace; specificEpithet: Procanacewirthi; scientificNameAuthorship: Hardy & Delfinado, 1980; **Location:** islandGroup: Hawaiian Islands; island: Oahu; verbatimLocality: Kaneohe, near Waihee Stream; minimumElevationInMeters: 705; **Identification:** identifiedBy: WN Mathis; dateIdentified: 1992; **Event:** verbatimEventDate: 22.xi.1991; **Record Level:** institutionCode: BPBM
- **Type status:** Other material. **Occurrence:** catalogNumber: 2006008032; recordedBy: DA Polhemus; lifeStage: adult; **Taxon:** kingdom: Animalia; phylum: Arthropoda; class: Insecta; order: Diptera; family: Canacidae; genus: Procanace; specificEpithet: Procanacewirthi; scientificNameAuthorship: Hardy & Delfinado, 1980; **Location:** islandGroup: Hawaiian Islands; island: Oahu; verbatimLocality: Kaneohe, near Waihee Stream; minimumElevationInMeters: 705; **Identification:** identifiedBy: WN Mathis; dateIdentified: 1992; **Event:** verbatimEventDate: 22.xi.1991; **Record Level:** institutionCode: BPBM
- **Type status:** Other material. **Occurrence:** catalogNumber: 2006008027; recordedBy: DA Polhemus; lifeStage: adult; **Taxon:** kingdom: Animalia; phylum: Arthropoda; class: Insecta; order: Diptera; family: Canacidae; genus: Procanace; specificEpithet: Procanacewirthi; scientificNameAuthorship: Hardy & Delfinado, 1980; **Location:** islandGroup: Hawaiian Islands; island: Oahu; verbatimLocality: Kaneohe, near Waihee Stream; minimumElevationInMeters: 705; **Identification:** identifiedBy: WN Mathis; dateIdentified: 1992; **Event:** verbatimEventDate: 22.xi.1991; **Record Level:** institutionCode: BPBM
- **Type status:** Other material. **Occurrence:** catalogNumber: 2006005179; recordedBy: DA Polhemus; lifeStage: adult; **Taxon:** kingdom: Animalia; phylum: Arthropoda; class: Insecta; order: Diptera; family: Canacidae; genus: Procanace; specificEpithet: Procanacewirthi; scientificNameAuthorship: Hardy & Delfinado, 1980; **Location:** islandGroup: Hawaiian Islands; island: Oahu; verbatimLocality: Kaneohe, near Waihee Stream; minimumElevationInMeters: 705; **Identification:** identifiedBy: WN Mathis; dateIdentified: 1992; **Event:** verbatimEventDate: 22.xi.1991; **Record Level:** institutionCode: BPBM
- **Type status:** Other material. **Occurrence:** catalogNumber: 2006005180; recordedBy: DA Polhemus; lifeStage: adult; **Taxon:** kingdom: Animalia; phylum: Arthropoda; class: Insecta; order: Diptera; family: Canacidae; genus: Procanace; specificEpithet: Procanacewirthi; scientificNameAuthorship: Hardy & Delfinado, 1980; **Location:** islandGroup: Hawaiian Islands; island: Oahu; verbatimLocality: Kaneohe, near Waihee Stream; minimumElevationInMeters: 705; **Identification:** identifiedBy: WN Mathis; dateIdentified: 1992; **Event:** verbatimEventDate: 22.xi.1991; **Record Level:** institutionCode: BPBM
- **Type status:** Other material. **Occurrence:** catalogNumber: 2006005181; recordedBy: DA Polhemus; lifeStage: adult; **Taxon:** kingdom: Animalia; phylum: Arthropoda; class: Insecta; order: Diptera; family: Canacidae; genus: Procanace; specificEpithet: Procanacewirthi; scientificNameAuthorship: Hardy & Delfinado, 1980; **Location:** islandGroup: Hawaiian Islands; island: Oahu; verbatimLocality: Kaneohe, near Waihee Stream; minimumElevationInMeters: 705; **Identification:** identifiedBy: WN Mathis; dateIdentified: 1992; **Event:** verbatimEventDate: 22.xi.1991; **Record Level:** institutionCode: BPBM
- **Type status:** Other material. **Occurrence:** catalogNumber: 2006005182; recordedBy: DA Polhemus; lifeStage: adult; **Taxon:** kingdom: Animalia; phylum: Arthropoda; class: Insecta; order: Diptera; family: Canacidae; genus: Procanace; specificEpithet: Procanacewirthi; scientificNameAuthorship: Hardy & Delfinado, 1980; **Location:** islandGroup: Hawaiian Islands; island: Oahu; verbatimLocality: Kaneohe, near Waihee Stream; minimumElevationInMeters: 705; **Identification:** identifiedBy: WN Mathis; dateIdentified: 1992; **Event:** verbatimEventDate: 22.xi.1991; **Record Level:** institutionCode: BPBM
- **Type status:** Other material. **Occurrence:** recordedBy: DA Polhemus; individualCount: 8; sex: 5 males, 3 females; lifeStage: adult; **Taxon:** kingdom: Animalia; phylum: Arthropoda; class: Insecta; order: Diptera; family: Canacidae; genus: Procanace; specificEpithet: Procanacewirthi; scientificNameAuthorship: Hardy & Delfinado, 1980; **Location:** islandGroup: Hawaiian Islands; island: Oahu; verbatimLocality: Waihee Stream, near Kaneohe; verbatimElevation: 215; **Event:** verbatimEventDate: 22.xi.1991; **Record Level:** institutionCode: USNM
- **Type status:** Other material. **Occurrence:** catalogNumber: 2006005184; recordedBy: DA Polhemus; lifeStage: adult; **Taxon:** kingdom: Animalia; phylum: Arthropoda; class: Insecta; order: Diptera; family: Canacidae; genus: Procanace; specificEpithet: Procanacewirthi; scientificNameAuthorship: Hardy & Delfinado, 1980; **Location:** islandGroup: Hawaiian Islands; island: Oahu; verbatimLocality: Kaneohe, near Waihee Stream; minimumElevationInMeters: 705; **Identification:** identifiedBy: WN Mathis; dateIdentified: 1992; **Event:** verbatimEventDate: 22.xi.1991; **Record Level:** institutionCode: BPBM
- **Type status:** Other material. **Occurrence:** catalogNumber: 2006005183; recordedBy: DA Polhemus; lifeStage: adult; **Taxon:** kingdom: Animalia; phylum: Arthropoda; class: Insecta; order: Diptera; family: Canacidae; genus: Procanace; specificEpithet: Procanacewirthi; scientificNameAuthorship: Hardy & Delfinado, 1980; **Location:** islandGroup: Hawaiian Islands; island: Oahu; verbatimLocality: Kaneohe, near Waihee Stream; minimumElevationInMeters: 705; **Identification:** identifiedBy: WN Mathis; dateIdentified: 1992; **Event:** verbatimEventDate: 22.xi.1991; **Record Level:** institutionCode: BPBM

##### Ecological interactions

###### Native status

endemic

##### Distribution

**Figure 14.**
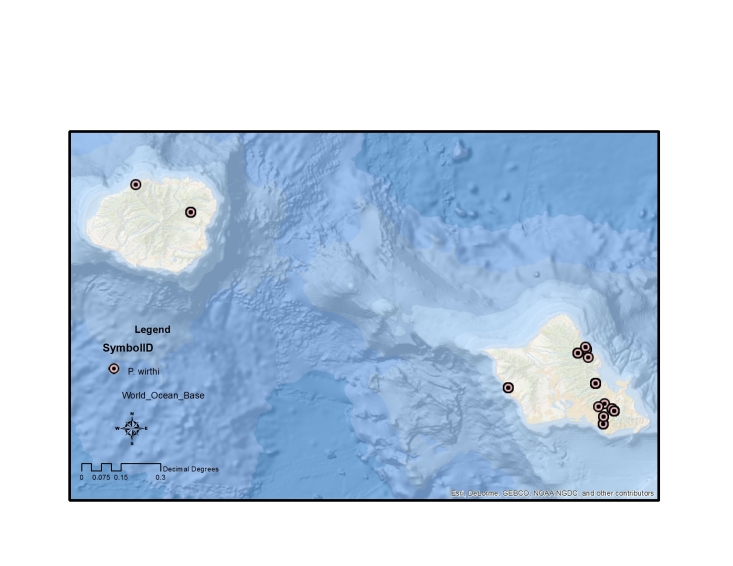
Distribution of *P. wirthi* (Oahu and Kauai)

HAWAIIAN ISLANDS: Kauai, Oahu (Fig. 14​).

##### Notes

Hardy and Delfinado (1980), [redescription and revision of Hawaiian taxa; head (front and lateral), female terminalia (dorsal and ventral), spermathecae, wing, surstylus; cephalopharyngael skeleton (larval and pupal, puparium, third instar larvae]; Mathis (1992), [World Catalog]; Nishida (2002), [Hawaiian Arthropod Checklist].

#### Procanace unnamed species

##### Materials

- **Type status:** Other material. **Occurrence:** recordedBy: JA Tenorio; lifeStage: adult; **Taxon:** kingdom: Animalia; phylum: Arthropoda; class: Insecta; order: Diptera; family: Canacidae; genus: Procanace; specificEpithet: unnamed species; **Location:** islandGroup: Hawaiian Islands; island: Hawaii; verbatimLocality: Wailuku River; verbatimElevation: 50 ft.; **Event:** verbatimEventDate: 23.vi.1971
- **Type status:** Other material. **Occurrence:** recordedBy: PM O'Grady, BS Ort, RT Lapoint, GM Bennett; lifeStage: adult; **Taxon:** kingdom: Animalia; phylum: Arthropoda; class: Insecta; order: Diptera; family: Canacidae; genus: Procanace; specificEpithet: unnamed species; **Location:** islandGroup: Hawaiian Islands; island: Kauai; verbatimLocality: Hanapakai Stream; **Identification:** identifiedBy: PM O'Grady; dateIdentified: 2014; **Event:** verbatimEventDate: 11.i.2010; **Record Level:** institutionCode: EMEC; collectionCode: 205649

##### Ecological interactions

###### Native status

endemic

##### Distribution

HAWAIIAN ISLANDS: Hawaii

## Identification Keys

### Revised Key to the Hawaiian Species of Canacidae

**Table:** 

1	C broken once at terminus of Sc (Fig. 15); lower portion of face not bulging (Fig. 16​).	Canacidae, 2
–	C broken twice, with both humeral and subcostal breaks (Fig. 22​); lower portion of face typically large, extending over oral cavity (Fig. 21).	Ephydridae
2	Single pair of fronto-orbital setae present; frons with only a few scattered setae; gena covered with short setae; oral vibrissae weak; only two incomplete rows of acrostichael setulae; cell M joined with 2nd cell M2.	Pelomyiinae, *Pelomyia occidentalis* Williston, 1893
–	Three to five fronto-orbital setae present; other characters not in the combination above.	3
3	Genal setae lacking.	Tethininae, refer to Munari & Evenhuis (2011)
–	Genal setae present.	Canacinae, 4
4	One pair of strong setae present on front, just anterior and lateral to the ocellar triangle (Fig. 16); mesofrons lacking setae; 4 pairs of strong setae present on genae (Fig. 16​); scutellum with 2-4 strong setae on disc (Fig. 17​).	*Canaceoides*, 5
–	No strong setae on front; at least a few setulae on mesofrons; 3 (or fewer) pairs of genal setae; scutellum lacking strong setae on disc (Fig. 20​).	*Procanace*, 6
5	Femur irregularly setose, with setae present on all posterior surfaces (Fig. 18​). Hawaiian Islands, Ecuador, Mexico, Peru.	*Canaceoides angulatus* Wirth
–	Femur with setae on two distinct rows on posterior and posterodorsal surfaces; no setae on posteroventral surface (Fig. 19​). Hawaiian Islands.	*Canaceoides hawaiiensis* Wirth
6	Predominately dull black, including squamae and halteres, often with brownish pollinosity on mesonotum and scutellum and greenish sheen over front and face; clypeus very large, nearly as long as face; mesofrons with only a few setae on lower portion; actostichal setulae absent; wings dark, smoky gray; usually two strong genal setae. Hawaiian Islands.	*nigroviridis* complex, 7
–	Mostly lighy-gray pollinose, with squamae and halteres yellow; clypeus small, less than ½ length of face; front with numerous setulae over lower ½ - 2/3; acrostichals well developed; 3 strong genal setae. Oahu, Japan.	*Procanace williamsi* Wirth
7	Males.	8
–	Females.	14
8	Surstylus bifurcate, each lobe roughly equal in size; lacking a small basal lobe on outer margin.	9
–	Surstylus not divided, with small basal lobe arising from outer ventral margin.	10
9	Outer lobe of surstylus straight, broad (not tapered and blunt), rounded at apex. Kauai, Oahu, Molokai, Maui.	*Procanace bifurcata* Hardy & Delfinado
–	Outer lobe curved upward, tapered at apex, with apical portion thinner, more translucent than remainder of lobe. Hawaii, Maui.	*Procanace confusa* Hardy & Delfinado
10	Surstylus slender, boomerang shaped. Kauai, Oahu.	*Procanace wirthi* Hardy & Delfinado
–	Surstylus not as above.	11
11	Surstylus comparatively slender, 3-4x longer than wide. Hawaii, Maui, Molokai.	*Procanace acuminata* Hardy & Delfinado
–	Surstylus shorter and broader, ½ longer than wide	12
12	Hypandrium forming a complete ring beneath apices of stustyli; front and face usually with a coppery green sheen; mesonotum and scutellum chocolate-brown pollinose with a faint green sheen. Kauai.	*Procanace nigroviridis* Cresson
–	Hypandrium developed into two upcurved arms on venter; coloration not as above.	13
13	Oahu, Molokai, Maui, Hawaii.	*Procanace constricta* Hardy & Delfinado
–	Kauai	*Procanace hardyi* sp. nov
14	Second tergum greatly elongated, tergites 1+2 longer than remainder of abdomen. Hawaii, Maui, Molokai.	*Procanace acuminata* Hardy & Delfinado
–	Second tergum equal in size of other tergites (or only slightly longer than tergites 1 and 3 in *P. wirthi*).	15
15	Abdomen constricted medially.	16
–	Abdomen not constricted medially.	17
16	Lateral margins of tergite 8 do not extend beyond the apex of the ovipositor; thickened setae on ovipositor long, distinct (Fig. 5). Kauai.	*Procanace hardyi* sp. nov.
–	Lateral margins of tergite 8 extend beyond the apex of the ovipositor; thickened setae on ovipositor short, indistinct (Fig. 6). Oahu, Molokai, Maui, Hawaii.	*Procanace constricta* Hardy & Delfinado
17	Posterior plates of 8th sternum each with 7-8 long setae; 8th tergum with a row long and short setae.	18
–	Characteristics of 8th sterna and terga not as above.	19
18	Kauai, Oahu, Molokai, Maui.	*Procanace bifurcata* Hardy & Delfinado
–	Maui and Hawaii.	*Procanace confusa* Hardy & Delfinado
19	Eighth tergum comparatively narrow, with four long setae on hind margin. Kauai.	*Procanace quadrisetosa* Hardy and Delfinado
–	Eighth tergum with numerous setae of varying lengths.	20
20	Cerci short and broad, as wide as long; eigth tergum cordate, as long as wide; ninth sternum nearly divided in middle of anterior margin; seventh sternum large, at least two times longer than atrial sclerotization and convex. Oahu and Kauai.	*Procanace wirthi* Hardy & Delfinado
–	Cerci longer than wide; eigth tergum about two times wider than long; ninth sternum entire, not concave on anterior margin; seventh sternum scarcely longer than atrial sclerotization and concave on posterior margin. Kauai.	*Procanace nigroviridis* Hardy & Delfinado

This key relies heavily on the couplets in the Hardy and Delfinado (1980) keys to the Tethinidae and the Canacidae. It has been modified to accommodate the inclusion of Tethinidae in the Canacidae and to add the new species *Procanace hardyi*. The first couplet separates Canacidae and Ephydridae. While these families aren't close phylogenetically, they are commonly collected in the same habitats in Hawaii and do look quite similar.

## Supplementary Material

XML Treatment for Procanace hardyi

XML Treatment for Canaceoides angulatus

XML Treatment for Canaceoides hawaiiensis

XML Treatment for Procanace acuminata

XML Treatment for Procanace bifurcata

XML Treatment for Procanace confusa

XML Treatment for Procanace constricta

XML Treatment for Procanace nigroviridis

XML Treatment for Procanace quadrisetosa

XML Treatment for Procanace williamsi

XML Treatment for Procanace wirthi

XML Treatment for Procanace unnamed species

## Figures and Tables

**Figure 15.**
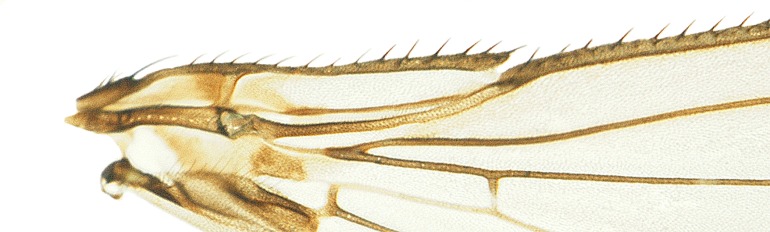
Wing of *Canaceoides hawaiiensis*, showing a single break at the terminus of the subcostal vein. The specimen imaged was from collection 205542.

**Figure 16.**
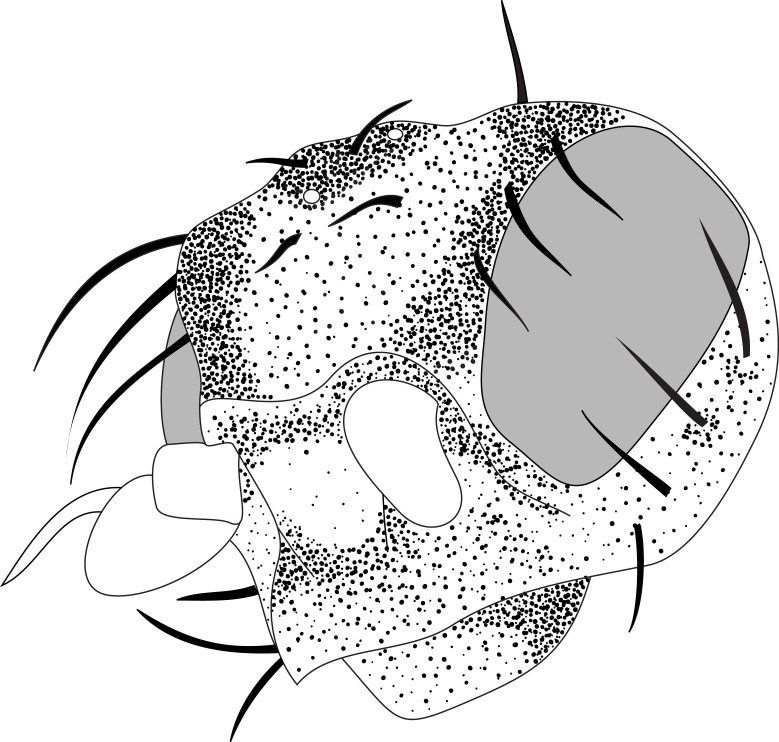
Face of *Canaceoides hawaiiensis*, showing the widely separated antennal bases and swollen upper portion of the face and enlarged clypeus. The specimen imaged was from collection 205542.

**Figure 17.**
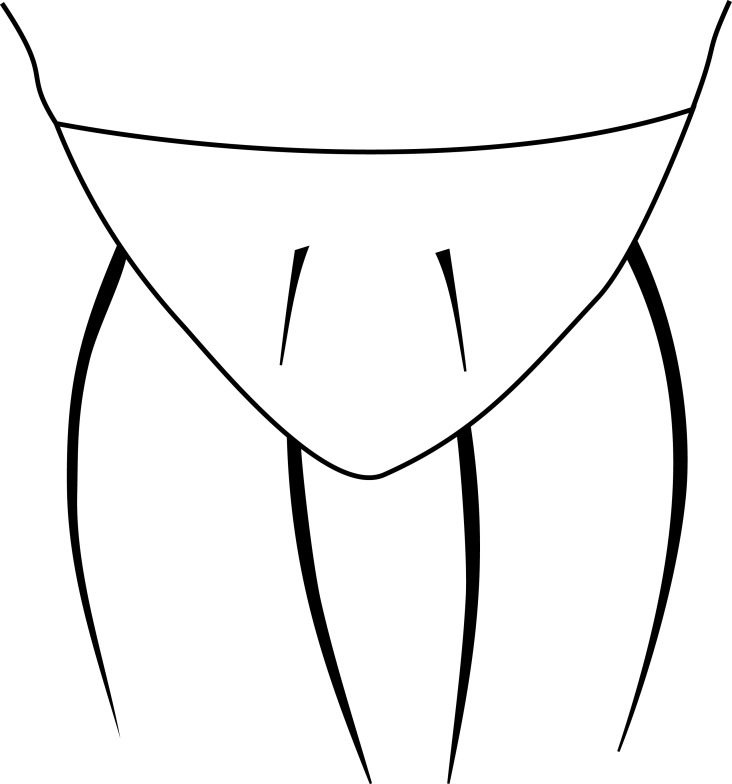
Dorsal view of posterior thorax of *Canaceoides angulatus* showing extranumerary setae on disc of scutellum. The specimen imaged is from collection 205519.

**Figure 18.**
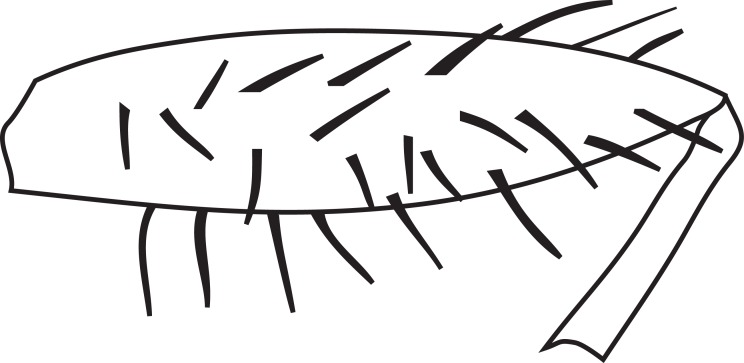
Leg of *Canaceoides angulatus* showing irregularly placed setae on posterior surface of femur. The specimen imaged is from collection 205519.

**Figure 19.**
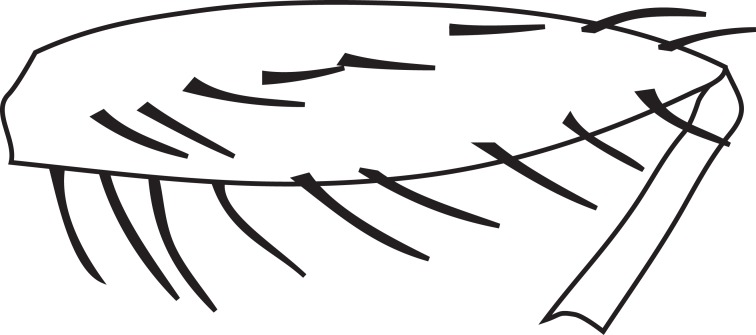
Leg of *Canaceoides hawaiiensis*, showing two regular rows of setae on posterior surface of femur. The specimen imaged was from collection 205542.

**Figure 20.**
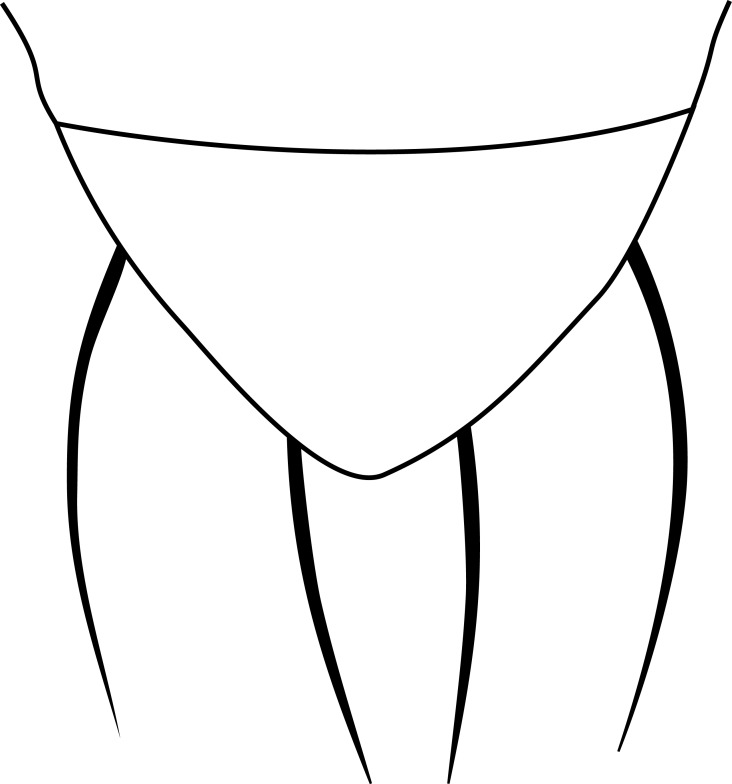
Dorsal view of posterior thorax of *Procanace constricta* showing the lack of supernumerary setae on disc of scutellum. The specimen imaged is from collection 205268.

**Figure 21.**
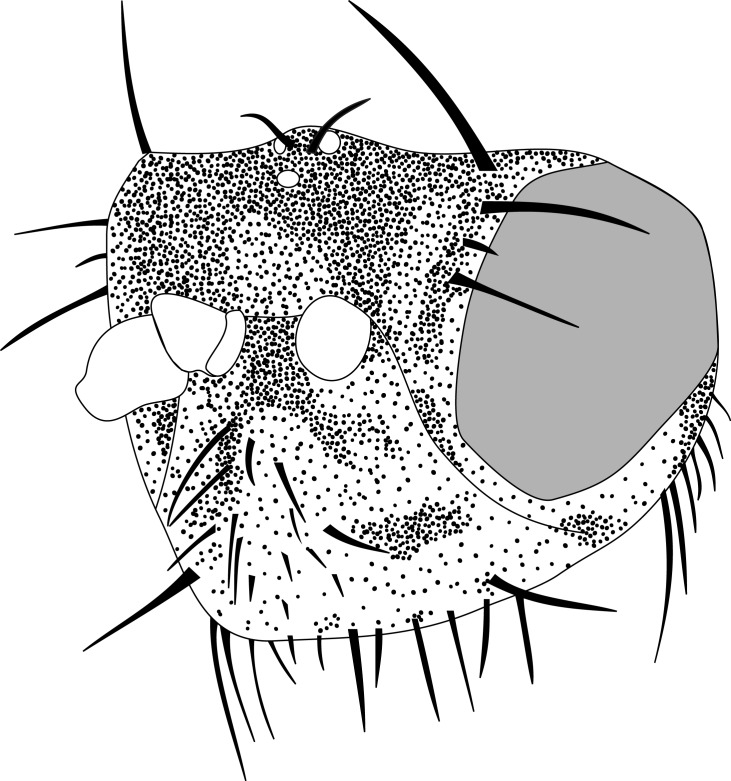
Head of *Scatella kauaiensis*, showing the large face which extends over the oral cavity. The clypeus is normal in size and does not extend over the mouth. The specimen imaged was from collection 205192.

**Figure 22.**
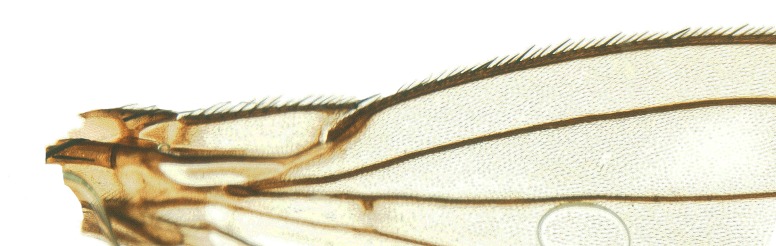
Wing of *Scatella kauaiensis*, showing breaks in the costal vein at the insertion points of the humeral crossvein and the subcostal vein. The specimen imaged was from collection 205192.

**Table 1.** **Distribution of *Canaceoides* and *Procanace* species in Hawaii.** Status indicates whether the species are endemic (E) or adventive (A). Islands sampled are: Kauai (K), Oahu (O), Molokai (Mo), Lanai (L), Kahoolawae (Ka) Maui (Ma), Hawaii (H), and the Northwest Hawaiian Islands (NWHI). Presence of a species on a given island is designated by an X. An asterisk denotes a new island record for the species.

Species	Status	K	O	Mo	L	Ka	Ma	H	NWHI
*C. angulatus*	A	X	X	X		X*	X	X	Laysan, Lisianski, Midway, Nihoa
*C. hawaiiensis*	E	X	X	X			X	X	Nihoa
*P. acuminata*	E			X			X	X	
*P. bifurcata*	E	X	X	X*			X*		
*P. confusa*	E						X	X	
*P. constricta*	E		X*	X			X	X	
*P. hardyi*	E	X							
*P. nigroviridis*	E	X							
*P. quadrisetosa*	E	X							
*P. williamsi*	A		X						
*P. wirthi*	E	X	X						
P. unnamed sp. DEH	E							X	
